# Supplementary material for: Global prevalence of elevated estimated pulmonary artery systolic pressure in clinically stable children and adults with sickle cell disease: A systematic review and meta-analysis
Source: PLoS One. 2025 Feb 13;20(2):e0318751. doi: 10.1371/journal.pone.0318751 (PMC11825009; doi:10.1371/journal.pone.0318751)
Supplement: S2 File — (DOC) [file pone.0318751.s002.doc]

**Supplementary Online Content**

**Search Syntax**

**PubMed**

((pulmonary hypertension[tiab] OR PAP[tiab] OR pulmonary arterial pressure[tiab] OR PASP[tiab] OR pulmonary artery systolic pressure[tiab] OR TRV[tiab] OR Tricuspid regurgitant jet velocity[tiab] OR echocardiography[tiab]) AND (Sickle cell disease[tiab] OR Sickle Cell Anemia[tiab] OR Sickle Cell Trait[tiab] OR Hemoglobin S Disease[tiab]))

N= 751

**Scopus**

((TITLE-ABS(pulmonary hypertension) OR TITLE-ABS(PAP) OR TITLE-ABS(pulmonary arterial pressure) OR TITLE-ABS(PASP) OR TITLE-ABS(pulmonary artery systolic pressure) OR TITLE-ABS(TRV) OR TITLE-ABS(tricuspid regurgitant jet velocity OR TITLE-ABS(echocardiography) AND (TITLE-ABS(Sickle cell disease) OR TITLE-ABS(Sickle Cell Anemia) OR TITLE-ABS(Sickle Cell Trait) OR TITLE-ABS(Hemoglobin S Disease)))

N= 2980

**WOS**

((TS=pulmonary hypertension OR TS=PAP OR TS=pulmonary arterial pressure OR TS=PASP OR TS=pulmonary artery systolic pressure OR TS=TRV OR TS=tricuspid regurgitant jet velocity OR TS= echocardiography) AND (TS=Sickle cell disease OR TS=Sickle Cell Anemia OR TS=Sickle Cell Trait OR TS=Hemoglobin S Disease))

N= 1524

**Embase**

((“pulmonary hypertension”:ti,ab OR PAP:ti,ab OR “pulmonary arterial pressure”:ti,ab OR PASP:ti,ab OR “pulmonary artery systolic pressure”:ti,ab OR TRV:ti,ab OR “Tricuspid regurgitant jet velocity”:ti,ab OR “echocardiography”:ti,ab) AND (Sickle cell disease:ti,ab OR “Sickle Cell Anemia”:ti,ab OR “Sickle Cell Trait”:ti,ab OR “Hemoglobin S Disease”:ti,ab))

N= 1794

**Science Direct**

((title-abs-key(pulmonary hypertension) OR title-abs-key(PAP) OR title-abs-key(pulmonary arterial pressure) OR title-abs-key(PASP) OR title-abs-key(pulmonary artery systolic pressure) OR title-abs-key(TRV) OR title-abs-key(tricuspid regurgitant jet velocity) OR title-abs-key(echocardiography)) AND (title-abs-key(Sickle cell disease) OR title-abs-key(Sickle Cell Anemia) OR title-abs-key(Sickle Cell Trait) OR title-abs-key(Hemoglobin S Disease)))

N= 843

**Google scholar**

allintitle: "pulmonary hypertension" + "sickle cell disease"

N= 215
