# Supplementary material for: Global prevalence of elevated estimated pulmonary artery systolic pressure in clinically stable children and adults with sickle cell disease: A systematic review and meta-analysis
Source: PLoS One. 2025 Feb 13;20(2):e0318751. doi: 10.1371/journal.pone.0318751 (PMC11825009; doi:10.1371/journal.pone.0318751)
Supplement: S3 File — (DOCX) [file pone.0318751.s003.docx]

eTable. List of excluded studies identified during initial screening

| No. | Author | Title | Brief report for excluded studies |
| --- | --- | --- | --- |
|  | No author name available | Synthesis: Sickle cell anemia and the lungs | ABSTRACT |
|  | Dosunmu et al., 2013 | Pattern of chronic lung lesions in adults with sickle cell disease in Lagos, Nigeria | ABSTRACT |
|  | Aalbers et al., 2012 | Recruitment of pulmonary hypertension patients for PATENT trial with RIOCIQUAT completed: Sub-Saharan pulmonary hypertension study launched | TITLE |
|  | Abdennour et al., 2013 | Elevated tricuspid regurgitant Jet velocity in lebanese patients with sickle cell disease is associated with more severe disease, familialy clustered and not responsive to hydroxyurea | ABSTRACT |
|  | Abboud et al., 2009 | Hematopoietic stem-cell transplantation for adults with sickle cell disease | TITLE |
|  | Abboud et al., 2009 | Sickle cell disease at the dawn of the molecular era | TITLE |
|  | Abdennour et al., 2013 | Elevated tricuspid regurgitant jet velocity in lebanese patients with sickle cell disease is associated with severe disease and is clustered in families | ABSTRACT |
|  | Abdel Raheem et al., 2019 | Hydroxyurea and cardiac sequelae in children with sickle cell disease | ABSTRACT |
|  | Abdellatif et al., 2012 | Cardiac tamponade due to umbilical venous catheter in the new born | TITLE |
|  | Abman et al., 2016 | Executive summary of the American Heart Association and American thoracic society joint guidelines for pediatric pulmonary hypertension | TITLE |
|  | Abman et al., 2009 | Towards improving the care of children with pulmonary hypertension: The rationale for developing a Pediatric Pulmonary Hypertension Network | ABSTRACT |
|  | Abu-Hashish et al., 2023 | SICKLE CELL BREAKING HEARTS | TITLE |
|  | Abman et al., 2015 | Pediatric pulmonary hypertension | ABSTRACT |
|  | Adamkiewicz et al., 2020 | ETA Receptor Blockade and Vascular Function in Patients with Sickle Cell Disease | ABSTRACT |
|  | Adjagba et al., 2017 | Impact of sickle cell anaemia on cardiac chamber size in the paediatric population | ABSTRACT |
|  | Adamkiewicz et al. 2018 | Quality of care indicators in patients with sickle cell disease (SCD): Influenza vaccination and routine eye examination are associated with outpatient utilization but not acute hospital care, whereas comorbidity indexes are strongly associated with both | ABSTRACT |
|  | Adams-Graves et al., 2016 | Recent treatment guidelines for managing adult patients with sickle cell disease: challenges in access to care, social issues, and adherence | Title |
|  | Adams-Graves et al., 2009 | Inhaled nitric oxide use for acute chest syndrome | TITLE |
|  | Adebiyi et al., 2010 | Noninvasive estimation of pulmonary artery pressures in patients with sickle cell anemia in Ibadan, Nigeria: An echocardiographic study | ABSTRACT |
|  | Adedeji et al., 2001 | Pulmonary thrombotic arteriopathy in patients with sickle cell disease | TITLE |
|  | Adekile et al., 2011 | Limitations of Hb F as a phenotypic modifier in sickle cell disease: study of Kuwaiti Arab patients | ABSTRACT |
|  | Adekile et al., 2016 | The influence of HBF on the pathophysiology and phenotype of sickle cell disease | ABSTRACT |
|  | Adisa et al., 2013 | Association between plasma free haem and incidence of vaso-occlusive episodes and acute chest syndrome in children with sickle cell disease | ABSTRACT |
|  | Adly et al., 2016 | Soluble fas and Fas legend levels in young patients with sickle cell disease: Role in nephropathy and cardiopulmonary complications | TITLE |
|  | Adsumilli et al., 2013 | Splenectomy Is Not Associated With Higher Tricuspid Regurgitant Jet Velocity In Patients With Sickle Cell Disease | ABSTRACT |
|  | Aduloju et al., 2008 | Mortality in Sickle Cell Patient Transitioning from Pediatric to Adult Program: 10 Years Grady Comprehensive Sickle Cell Center Experience | ABSTRACT |
|  | Aessopos et al., 2007 | Endothelial function and arterial stiffness in sickle-thalassemia patients | ABSTRACT |
|  | Aessopos et al., 2001 | Exercise-induced myocardial perfusion abnormalities in sickle beta-thalassemia: Tc-99m tetrofosmin gated SPECT imaging study | TITLE |
|  | Afifi et al., 2019 | IL-Iβ +3954 C / T polymorphism and its clinical associations in egyptian sickle cell disease patients | ABSTRACT |
|  | Afrin et al., 2016 | Mast cell activation disease and the modern epidemic of chronic inflammatory disease | TITLE |
|  | Afriyie-Mensah et al., 2017 | Pulmonary hypertension and associated risk factors in a high burden sickle cell disease population | ABSTRACT |
|  | Agarwal et al., 2018 | Association between pulmonary hypertension and clinical outcomes in hospitalized patients with sickle cell disease | ABSTRACT |
|  | Agarwal et al., 2019 | Candida albicans induced acute chest syndrome in sickle cell disease | TITLE |
|  | Agbakou et al., 2020 | Outcome of adult sickle cell patients admitted in ICU: National retrospective study in French ICUs | TITLE |
|  | Agha et al., 2013 | Sickle cell anemia: Imaging from head to toe | TITLE |
|  | Agheli et al., 2008 | Changes of BNP Level and Pulmonary Arterial Pressure during An Acute Sickle Cell Crisis, Comparison with Steady State | ABSTRACT |
|  | Agrawal et al., 2018 | Successful pulmonary thromboendarterectomy in a patient with sickle cell disease and associated resolution of a leg ulcer | TITLE |
|  | Ahmed et al., 2004 | Echocardiographic abnormalities in sickle cell disease | ABSTRACT |
|  | Ahner et al., 2022 | Can We Perform the Maximal Treadmill Test on Individuals with Sickle Cell Disease? | TITLE |
|  | Aich et al., 2020 | Microfluidics in Sickle Cell Disease Research: State of the Art and a Perspective Beyond the Flow Problem | TITLE |
|  | Aigbe et al., 2012 | Left ventricular structure and function in black normotensive type 2 diabetes mellitus patients | TITLE |
|  | Akgul et al., 2019 | Decreased Heart Rate Variability in Sickle Cell Anemia as Effect of Pulmonary Arterial Hypertension | ABSTRACT |
|  | Akgül et al., 2007 | Increased QT dispersion in sickle cell disease: Effect of pulmonary hypertension | ABSTRACT |
|  | Ako et al., 2016 | MR-augmented cardiopulmonary exercise testing-a proof of concept in sickle cell disease (SCD) | TITLE |
|  | Akgül et al., 2006 | Right ventricular and pulmonary function in sickle cell disease patients with pulmonary hypertension | ABSTRACT |
|  | Akinsheye et al., 2010 | Sickle cell anemia and vascular dysfunction: the nitric oxide connection | ABSTRACT |
|  | Akkus et al., 2021 | Troponin Elevation in Sickle Cell Disease | ABSTRACT |
|  | Al Kahf et al., 2023 | Pulmonary hypertension in sickle cell disease | ABSTRACT |
|  | Alashkar et al., 2020 | Veno-Venous Extracorporeal Membrane Oxygenation in Adult Patients with Sickle Cell Disease and Acute Chest Syndrome: a Single-Center Experience | ABSTRACT |
|  | Al-Dabbagh et al., 2004 | Association of sickle cell anemia and glycogen storage disease type 1a | TITLE |
|  | Al-Humrani et al., 2004 | Prevalence of mitral valve prolapse among patients with sickle cell anemia | TITLE |
|  | Al-Ali et al., 2009 | Assessment of pulmonary hypertension in patients with sickle cell disease with tricuspid regurgitant jet velocity of greater than or equal to 2.5 m/second on a routine echocardiogram | ABSTRACT |
|  | Al Asoom et al., 2020 | Effects of-3.7α deletion and sickle-cell trait on ventilatory and hemodynamic responses to maximum exercise in young saudi females | ABSTRACT |
|  | Alablani et al., 2022 | Development of a Flow Phantom for Transcranial Doppler Ultrasound Quality Assurance | TITLE |
|  | Alsoufi et al., 2016 | The Effect of Noncardiac and Genetic Abnormalities on Outcomes Following Neonatal Congenital Heart Surgery | TITLE |
|  | Alum et al., 2022 | Factors Associated With In-Hospital Post-Cardiac Arrest Survival in a Referral Level Hospital in Uganda | TITLE |
|  | Alam et al., 2023 | Pyrazole: an emerging privileged scaffold in drug discovery | TITLE |
|  | Alam et al., 2011 | Study of clinical characteristics of sickle cell disease patients developing COR-pulmonale during acute hospitalization | TITLE |
|  | Alameri et al., 2008 | Dyspnea, pulmonary function and exercise capacity in adult Saudi patients with sickle cell disease | ABSTRACT |
|  | Alapan et al., 2016 | Emerging point-of-care technologies for sickle cell disease screening and monitoring | ABSTRACT |
|  | Alayash et al., 2018 | Oxidative pathways in the sickle cell and beyond | TITLE |
|  | Albinni et al., 2020 | Focused update on pulmonary hypertension in children—selected topics of interest for the adult cardiologist | TITLE |
|  | Al-Ebrahim et al., 2008 | Cardiac surgery and sickle cell disease | TITLE |
|  | Aleem et al., 2007 | Echocardiographic abnormalities in adolescent and adult Saudi patients with sickle cell disease | ABSTRACT |
|  | Aleluia et al., 2017 | Association of classical markers and establishment of the dyslipidemic subphenotype of sickle cell anemia | ABSTRACT |
|  | Aliyu et al., 2008 | Sickle cell disease and pulmonary hypertension in Africa: A global perspective and review of epidemiology, pathophysiology, and management | TITLE |
|  | Aliyu et al., 2010 | NT-proBNP as a marker of cardiopulmonary status in sickle cell anaemia in Africa: Research paper | ABSTRACT |
|  | Al-Khoufi et al., 2013 | Prevalence of pulmonary arterial hypertension among sickle cell disease patients in Al Hassa | ABSTRACT |
|  | Alkindi et al., 2015 | Management of sickle cell disease | TITLE |
|  | Alkindi et al., 2018 | Risk factors predisposing development of priapism in sickle cell disease patients | ABSTRACT |
|  | Alkindi et al., 2020 | Clinical and laboratory parameters, risk factors predisposing to the development of priapism in sickle cell patients | ABSTRACT |
|  | Allali et al., 2022 | Tocilizumab for severe acute chest syndrome in a child with sickle cell disease and dramatically high interleukin-6 values in endotracheal and pleural fluids | TITLE |
|  | Allali et al., 2021 | Chronic organ injuries in children with sickle cell disease | ABSTRACT |
|  | Alli et al., 2014 | Recommendations for the management of sickle cell disease in South Africa | TITLE |
|  | Almeida et al., 2021 | Therapist-oriented home rehabilitation for adults with sickle cell anemia: effects on muscle strength, functional capacity, and quality of life | TITLE |
|  | Almeida et al., 2020 | Sickle cell disease subjects and mouse models have elevated nitrite and cGMP levels in blood compartments | TITLE |
|  | Almusally et al., 2023 | Early recognition of pulmonary complications of sickle cell disease | ABSTRACT |
|  | Alobeidi et al., 2015 | Cerebral microhaemorrhages secondary to fat embolus syndrome in sickle cell disease | ABSTRACT |
|  | Al-Qudsi et al., 2023 | Voxelotor as a Treatment of Persistent Hypoxia in the ICU | TITLE |
|  | Alsaghir et al., 2023 | Successful therapeutic plasma exchange for a patient with sickle cell disease and fat embolism syndrome after a failure of a response to red cell exchange transfusion | TITLE |
|  | Alsaied et al., 2018 | Diastolic dysfunction is associated with exercise impairment in patients with sickle cell anemia | ABSTRACT |
|  | Alsaied et al., 2020 | Left atrial dysfunction in sickle cell anemia is associated with diffuse myocardial fibrosis, increased right ventricular pressure and reduced exercise capacity | ABSTRACT |
|  | Al-Samkari et al., 2020 | The variable manifestations of disease in pyruvate kinase deficiency and their management | TITLE |
|  | Alsilmi et al., 2022 | Chest computed tomographic findings in patients with sickle cell disease-related acute chest syndrome: A retrospective study | TITLE |
|  | Al-Suliman et al., 2006 | Patterns of mortality in adult sickle cell disease in Al-Hasa region of Saudi Arabia | ABSTRACT |
|  | Altawashi et al., 2015 | Inhaled nitric oxide for treating pain crises in people with sickle cell disease | ABSTRACT |
|  | Altman et al., 2016 | A treatment algorithm to identify therapeutic approaches for leg ulcers in patients with sickle cell disease | TITLE |
|  | Alvarez-Argote et al., 2023 | Pathophysiological characterization of the Townes mouse model for sickle cell disease | TITLE |
|  | Álvarez-Fernández et al., 2007 | Some more applications of transcranial Doppler in the ICU | TITLE |
|  | Alves Pereira et al., 2009 | Iron chelation in patients with thalassemia intermedia and other non transfusion dependent congenital hemolytic anemias | TITLE |
|  | Ambrusko et al., 2020 | Pulmonary hypertension in children with hemolytic disorders | ABSTRACT |
|  | Ambrusko et al., 2005 | Pulmonary Hypertension in Children with Sickle Cell Disease: Clinical Characteristics and Co-Morbidities | ABSTRACT |
|  | Ambrusko et al., 2006 | Elevation of tricuspid regurgitant jet velocity, a marker for pulmonary hypertension in children with sickle cell disease | ABSTRACT |
|  | Amira et al., 2018 | Pulmonary embolism in patients with sickle cell disease in intensive care unit: A challenging diagnosis | TITLE |
|  | Amoozgar et al., 2015 | Evaluation of cardiac sequelae in patients with sickle cell anemia | ABSTRACT |
|  | Anand et al., 2012 | Enzymatic mechanisms regulating protein s-nitrosylation: Implications in health and disease | TITLE |
|  | Andemariam et al., 2021 | Real-world experience of patients with sickle cell disease treated with voxelotor: A multicenter, retrospective study | TITLE |
|  | Anoop et al., 2009 | Usefulness and limitations of Bayesian network model as a mortality risk assessment tool in sickle cell anemia | TITLE |
|  | Aessopos et al., 2009 | Cardiac involvement in sickle beta-thalassemia | ABSTRACT |
|  | Aessopos et al., 2001 | Exercise-induced myocardial perfusion abnormalities in sickle β-thalassemia: Tc-99m tetrofosmin gated SPECT imaging study | TITLE |
|  | Lancet;Group of authors, 2015 | Global, regional, and national age-sex specific all-cause and cause-specific mortality for 240 causes of death, 1990-2013: a systematic analysis for the Global Burden of Disease Study 2013 | TITLE |
|  | Abba et al., 2023 | Echocardiographic Left Ventricular Hypertrophy and Geometric Patterns in Patients with Sickle Cell Anaemia | ABSTRACT |
|  | AbdelMassih et al., 2020 | Discrepancy in patterns of myocardial involvement in beta-thalassaemia vs. sickle cell anaemia | TITLE |
|  | Abo-Zenah et al., 2009 | Cardiorenal risk prevalence in sickle cell hemoglobinopathy | TITLE |
|  | Acar et al., 2000 | Myocardial perfusion in children with sickle cell anaemia | TITLE |
|  | Achigbu et al., 2015 | Pulmonary function indices in children with sickle cell anemia in Enugu, south-east Nigeria | ABSTRACT |
|  | Adam et al., 2013 | Potential role for statins in sickle cell disease | TITLE |
|  | Adebayo et al., 2002 | Cardiovascular changes in sickle cell anaemia | ABSTRACT |
|  | Adebayo et al., 2002 | The clinical, electrocardiographic and self-paced walking exercise features of Nigerians with sickle cell anaemia presenting at OAUTHC, Ile-Ife | TITLE |
|  | Adekile et al., 2018 | Correlates of Pulmonary Function in Children with Sickle Cell Disease and Elevated Fetal Hemoglobin | ABSTRACT |
|  | Adeniyi et al., 2023 | Blood pressure and electrocardiographic profile of children with sickle cell anaemia in steady-state and vaso-occlusive crisis | ABSTRACT |
|  | Adhikari et al., 2010 | Preoperative exchange transfusion for sickle cell disease patients undergoing open-heart surgery: an exception to the rule | TITLE |
|  | Adoukonou et al., 2010 | Management of stroke in sub-Saharan Africa: current issues | TITLE |
|  | Afzal et al., 2023 | Ultraviolet light oxidation of fresh hemoglobin eliminates aggregate formation seen in commercially sourced hemoglobin | TITLE |
|  | Agu et al., 2023 | Molecular docking as a tool for the discovery of molecular targets of nutraceuticals in diseases management | TITLE |
|  | Ahmad et al., 2012 | Evaluation of myocardial deformation in patients with sickle cell disease and preserved ejection fraction using three-dimensional speckle tracking echocardiography | ABSTRACT |
|  | Ahmed et al., 2005 | Hemoglobin oxygen saturation discrepancy using various methods in patients with sickle cell vaso-occlusive painful crisis | TITLE |
|  | Akinbami et al., 2019 | On artherogenic index of plasma in sickle cell anaemia patients | TITLE |
|  | Al Asoom et al., 2020 | Effects of (-3.7)α Deletion and Sickle-Cell Trait on Ventilatory and Hemodynamic Responses to Maximum Exercise in Young Saudi Females | TITLE |
|  | Al Biltagi et al., 2020 | Pulmonary functions in children and adolescents with sickle cell disease | ABSTRACT |
|  | Ali et al., 2019 | Catastrophic sickling crisis in patient undergoing cardiac transplantation with sickle cell trait | TITLE |
|  | Aliyu et al., 2022 | Effects of pregnancy on cardiac structure and function in women with sickle cell anemia: a longitudinal comparative study | TITLE |
|  | Alkhars et al., 2023 | Endoscopic Excision of Primary Tracheal Schwannoma: A Case Report | TITLE |
|  | Alkhunaizi et al., 2018 | Prevalence of Microalbuminuria in Adult Patients with Sickle Cell Disease in Eastern Saudi Arabia | ABSTRACT |
|  | Almeida et al., 2008 | Abnormal myocardial flow reserve in sickle cell disease: a myocardial contrast echocardiography study | TITLE |
|  | Almeida et al., 2015 | Validation of a method to directly and specifically measure nitrite in biological matrices | TITLE |
|  | Alvarado et al., 2015 | Heart rate recovery is impaired after maximal exercise testing in children with sickle cell anemia | TITLE |
|  | Alsultan et al., 2018 | Utilizing Whole-Exome Sequencing to Characterize the Phenotypic Variability of Sickle Cell Disease | TITLE |
|  | Alzahrani et al., 2021 | Non-myeloablative human leukocyte antigen-matched related donor transplantation in sickle cell disease: outcomes from three independent centres | TITLE |
|  | Angel et al., 2020 | Asthma, allergic sensitization and lung function in sickle cell disease | TITLE |
|  | Animasahun et al., 2010 | Echocardiographic findings among children with sickle cell anaemia at the Lagos University Teaching Hospital | ABSTRACT |
|  | Antonelli Rossi et al., 2023 | Effect of a Physical Exercise Program on the Inflammatory Response, Cardiac Functions, Functional Capacity, and Quality of Life in Patients with Sickle Cell Disease | TITLE |
|  | Antwi-Boasiako et al., 2021 | Exercise-induced haemoglobin oxygen desaturation in patients with SCD | TITLE |
|  | Antwi-Boasiako et al., 2021 | Association between pulmonary function and cardiac enzymes in sickle cell disease | TITLE |
|  | Ariganjoye et al., 2017 | Pediatric Hypovitaminosis D: Molecular Perspectives and Clinical Implications | TITLE |
|  | Arslankoylu et al., 2010 | Assessment of cardiac functions in sickle cell anemia with Doppler myocardial performance index | TITLE |
|  | Arteta et al., 2014 | Abnormal pulmonary function and associated risk factors in children and adolescents with sickle cell anemia | TITLE |
|  | Ashley-Koch et al., 2008 | Identification of genetic polymorphisms associated with risk for pulmonary hypertension in sickle cell disease | TITLE |
|  | Asiedu et al., 2017 | Prevalent Health Concerns Among African American Women Belonging to a National Volunteer Service Organization (The Links, Incorporated) | TITLE |
|  | Ansa et al., 2022 | Cardiovascular Findings in Adult Patients with Sickle Cell Anaemia in Steady State seen in Calabar, Nigeria | ABSTRACT |
|  | Ansari et al., 2019 | Ischemia-Reperfusion Injury in Sickle Cell Disease <i>From Basics to Therapeutics</i> | TITLE |
|  | Ansari et al., 2018 | Sickle cell disease: a malady beyond a hemoglobin defect in cerebrovascular disease | TITLE |
|  | Anthi et al., 2007 | Hemodynamic and functional assessment of patients with sickle cell disease and pulmonary hypertension | Abstract |
|  | Antoniu et al., 2010 | Enhancing the nitric oxide synthesis pathway in asthma: A plausible therapeutic approach? | TITLE |
|  | Anurogo et al., 2021 | Cell and gene therapy for anemia: Hematopoietic stem cells and gene editing | TITLE |
|  | Anusim et al., 2020 | Presentation, management and outcomes of COVID-19 patients with sickle cell disease | TITLE |
|  | Anderson et al., 2008 | The role of endomyocardial biopsy in the management of cardiovascular disease: A scientific statement from the American Heart Association, the American College of Cardiology, and the European Society of Cardiology | TITLE |
|  | Andrade et al., 2015 | Current Concepts in Pediatric Stroke | TITLE |
|  | Anwar et al., 2009 | Real-time three-dimensional echocardiographic assessment of inferior vena caval thrombosis | TITLE |
|  | Appenzeller et al., 2012 | Chorea in primary antiphospholipid syndrome is associated with rheumatic fever | TITLE |
|  | Archer et al., 2015 | 2015 Clinical trials update in sickle cell anemia | TITLE |
|  | Ardiles et al., 2007 | Pulmonary complications of sickle cell disease in adults | ABSTRACT |
|  | Arencibia-Núñez et al., 2012 | Hemolytic index: An approach to clinical subphenotypes in children with sickle cell disease | TITLE |
|  | Arigliani et al., 2020 | Management of chronic respiratory complications in children and adolescents with sickle cell disease | ABSTRACT |
|  | Arlet et al., 2021 | Association between Hospitalised Vaso-Occlusive Crises and Acute/Chronic Complications in Sickle Cell Disease Patients Aged 16 Years and Older Using the French National Health Insurance Database (SNDS) | TITLE |
|  | Armenis et al., 2019 | Reduced peripheral blood superoxide dismutase 2 expression in sickle cell disease | TITLE |
|  | Arteta et al., 2007 | Pulmonary Function Tests and Their Correlation with Tricuspid Regurgitant Jet Velocity in Pediatric Sickle Cell Disease Patients | ABSTRACT |
|  | Artin et al., 2010 | Caffeine-related atrial fibrillation | TITLE |
|  | Arumugam et al., 2015 | Genetic diminution of circulating prothrombin ameliorates multiorgan pathologies in sickle cell disease mice | TITLE |
|  | Asare et al., 2022 | Pattern of Haemolysis During Pregnancy in Women with Sickle Cell Disease (SCD), and its Effects on Feto-maternal Outcome | TITLE |
|  | Asbeutah et al., 2023 | Repeat transcranial Doppler ultrasound imaging in Kuwaiti children with sickle cell disease after a 10-year interval: A prospective, cohort study | Abstract |
|  | Ashley-Koch et al., 2004 | Genetic Polymorphisms Associated with Risk for Pulmonary Hypertension and Proteinuria in Sickle Cell Disease | ABSTRACT |
|  | Ashley-Koch et al., 2008 | Identification of genetic polymorphisms associated with risk for pulmonary hypertension in sickle cell disease | ABSTRACT |
|  | Ashouri et al., 2021 | Critical Role of Hemopexin Mediated Cytoprotection in the Pathophysiology of Sickle Cell Disease | TITLE |
|  | Aslan et al., 2007 | Redox-dependent impairment of vascular function in sickle cell disease | TITLE |
|  | Astadicko et al., 2018 | Cardiac complications of sickle cell disease in children | ABSTRACT |
|  | Ata et al., 2023 | Genotypic and Phenotypic Composition of Sickle Cell Disease in the Arab Population-A Systematic Review | TITLE |
|  | Ataga et al., 2009 | Hypercoagulability and thrombotic complications in hemolytic anemias | TITLE |
|  | Ataga et al., 2010 | Correlates and potential mechanmism of urinary albumin excretion in sickle cell disease | TITLE |
|  | Ataga et al., 2010 | Association of soluble fms-like tyrosine kinase-1 (sFLT-1) with pulmonary hypertension and hemolysis in sickle cell disease | TITLE |
|  | Ataga et al., 2012 | Association of coagulation activation with clinical complications in sickle cell disease | ABSTRACT |
|  | Ataga et al., 2011 | Association of soluble fms-like tyrosine kinase-1 with pulmonary hypertension and haemolysis in sickle cell disease | ABSTRACT |
|  | Ataga et al., 2010 | Urinary albumin excretion is associated with pulmonary hypertension in sickle cell disease: potential role of soluble fms-like tyrosine kinase-1 | TITLE |
|  | Ataga et al., 2014 | The glomerulopathy of sickle cell disease | TITLE |
|  | Ataga et al., 2016 | Albuminuria Is Associated with Endothelial Dysfunction and Elevated Plasma Endothelin-1 in Sickle Cell Anemia | TITLE |
|  | Ataga et al., 2020 | Low hemoglobin increases risk for cerebrovascular disease, kidney disease, pulmonary vasculopathy, and mortality in sickle cell disease: A systematic literature review and meta-analysis | TITLE |
|  | Ataga et al., 2015 | Association of pro-inflammatory high-density lipoprotein cholesterol with clinical and laboratory variables in sickle cell disease | TITLE |
|  | Ataga et al., 2008 | Association of Placenta Growth Factor with Hemolysis and Inflammation in Sickle Cell Disease (SCD) | TITLE |
|  | Ataga et al., 2014 | Pulmonary hypertension in sickle cell disease: Diagnosis and management | TITLE |
|  | Ataga et al., 2005 | Progression of Pulmonary Hypertension in Patients with Sickle Cell Disease | ABSTRACT |
|  | Ataga et al., 2008 | Coagulation activation and inflammation in sickle cell disease-associated pulmonary hypertension | ABSTRACT |
|  | Ataga et al., 2006 | Pulmonary hypertension in patients with sickle cell disease | ABSTRACT |
|  | Ataga et al., 2019 | The Effect of Crizanlizumab Plus Standard of Care (SoC) Ataga Versus Soc Alone on Renal Function in Patients with Sickle Cell Disease and Chronic Kidney Disease: A Randomized, Multicenter, Open-Label, Phase II Study (STEADFAST) | TITLE |
|  | Asnani et al., 2011 | Excess risk of maternal death from sickle cell disease in Jamaica: 1998-2007 | TITLE |
|  | Assanasen et al., 2003 | Acute myocardial infarction in sickle cell anemia | TITLE |
|  | Assari et al., 2014 | Race and Ethnic Differences in the Associations between Cardiovascular Diseases, Anxiety, and Depression in the United States | TITLE |
|  | Ataga et al., 2018 | Advances in new drug therapies for the management of sickle cell disease | TITLE |
|  | Ataga et al., 2015 | The trials and hopes for drug development in sickle cell disease | TITLE |
|  | Ataga et al., 2008 | Efficacy and safety of the Gardos channel blocker, senicapoc (ICA-17043), in patients with sickle cell anemia | TITLE |
|  | Ataga et al., 2004 | Pulmonary hypertension in sickle cell disease | ABSTRACT |
|  | Ataga et al., 2009 | Senicapoc (ICA-17043): A potential therapy for the prevention and treatment of hemolysis-associated complications in sickle cell anemia | TITLE |
|  | Atanda et al., 2017 | Rheumatic heart disease in sickle cell disease in sub-Saharan Africa | TITLE |
|  | Ates et al., 2017 | Laparoscopic splenectomy in pediatric age: Long-term follow-up | TITLE |
|  | Aubry et al., 2020 | Cor pulmonale | TITLE |
|  | Audard et al., 2010 | Acute kidney injury in sickle patients with painful crisis or acute chest syndrome and its relation to pulmonary hypertension | Title |
|  | Aurora et al., 2022 | The successful use of eculizumab for treatment of thrombotic microangiopathy in pediatric acute SARSCoV2 infection and multisystem inflammatory syndrome in children | TITLE |
|  | Austin et al., 2019 | Striking dyserythropoiesis in sickle cell anemia following an aplastic crisis | TITLE |
|  | Audino et al., 2010 | Prognostic value of pro-BNP and VEGF in children with sickle cell disease | ABSTRACT |
|  | Audardet al., 2006 | Successful combined heart and kidney transplantation in a patient with sickle-cell anemia | TITLE |
|  | Awodu et al., 2007 | Haemostatic variables and their relationship to body mass index and blood pressure in adult Nigerians with the sickle cell trait | TITLE |
|  | Augoustides et al., 2001 | Perioperative use of nitric oxide in cardiothoracic anesthesia and intensive care | TITLE |
|  | Aujla et al., 2020 | Cardiovascular Sequelae of Sickle Cell Disease | ABSTRACT |
|  | Austin et al., 2014 | Pulmonary hypertension: NHLBI workshop on the primary prevention of chronic lung diseases | TITLE |
|  | Azar et al., 2017 | Sickle Cell Disease: A Brief Update | TITLE |
|  | Azbell et al., 2021 | Treatment dilemmas: strategies for priapism, chronic leg ulcer disease, and pulmonary hypertension in sickle cell disease | TITLE |
|  | Aworanti et al., 2020 | Acute leukemia in sickle cell disease patients in a tertiary health facility in Nigeria: A case series | TITLE |
|  | Bacopoulou et al., 2010 | Reproductive and contraceptive issues in chronically ill adolescents | TITLE |
|  | Bachir et al., 2009 | Prospective multicentric survey on pulmonary hypertension (PH) in adults with sickle cell disease | ABSTRACT |
|  | Bacon et al., 2013 | Current diagnostic investigation in pulmonary hypertension | Title |
|  | Bacsu et al., 2012 | Priapism in an Infant With Sickle Cell Trait After Cardiac Transplant | TITLE |
|  | Badawy et al., 2016 | Cardiopulmonary fitness and clinical outcomes in adults followed in the cooperative study for sickle cell disease | ABSTRACT |
|  | Bachmeyer et al., 2014 | Unusual cutaneous lesions indicating fat embolism syndrome in homozygous sickle cell disease | TITLE |
|  | Ballas et al., 2014 | Primary stroke in a woman with sickle cell anemia responsive to hydroxyurea therapy | TITLE |
|  | Bala et al., 2023 | Prevalence and risk factors for pulmonary embolism in children with sickle cell disease: an institutional retrospective cohort study | ABSTRACT |
|  | Bardakci et al., 2006 | Surgical management of valvular heart disease associated with sickle cell anemia of HbD Los Angeles-type hemoglobinopathy | Title |
|  | Baritussio et al., 2023 | Transient pericardial constriction: A not so rare entity | TITLE |
|  | Bae et al., 2012 | Meta-analysis of 2040 sickle cell anemia patients: BCL11A and HBS1L-MYB are the major modifiers of HbF in African Americans | TITLE |
|  | Bae et al., 2015 | Association of FOXO3A polymorphisms with hematocrit, LDH and longevity in patients with sickle cell anemia from csscd, walk-phasst, and push clinical trials | TITLE |
|  | Bailey et al., 2019 | Relationship between Vaso-Occlusive Crises and Important Complications in Sickle Cell Disease Patients | ABSTRACT |
|  | Bakeer et al., 2016 | Sickle cell anemia mice develop a unique cardiomyopathy with restrictive physiology | TITLE |
|  | Bakshi et al., 2016 | The role of the arginine metabolome in pain: implications for sickle cell disease | TITLE |
|  | Balbaid et al., 2021 | Prevalence of Pulmonary Hypertension in Sickle Cell Anemiai Patient in KSA | ABSTRACT |
|  | Baldwin et al., 2022 | Medical and Non-medical Costs of Sickle Cell Disease and Treatments from a US Perspective: A Systematic Review and Landscape Analysis | TITLE |
|  | Balfour-Lynn et al., 2009 | Domiciliary Oxygen for Children | TITLE |
|  | Balfour-Lynn et al., 2009 | BTS guidelines for home oxygen in children | TITLE |
|  | Baliga et al., 2012 | Treatment of Heart Failure in Pulmonary Arterial Hypertension-The Urgency of Getting This Right | TITLE |
|  | Ballas et al., 2002 | Sickle cell anaemia - Progress in pathogenesis and treatment | TITLE |
|  | Ballas et al., 2013 | Lactate dehydrogenase and hemolysis in sickle cell disease | ABSTRACT |
|  | Ballas et al., 2015 | Clinical utility of lactate dehydrogenase in determining the severity of hemolysis in sickle cell anemia | TITLE |
|  | Ballas et al., 2012 | Beyond the definitions of the phenotypic complications of sickle cell disease: An update on management | TITLE |
|  | Ballas et al., 2010 | Definitions of the phenotypic manifestations of sickle cell disease | TITLE |
|  | Ballas et al., 2006 | Hyperhemolysis during the evolution of uncomplicated acute painful episodes in patients with sickle cell anemia | ABSTRACT |
|  | Bader et al., 2015 | Clinical Q & A: Translating Therapeutic Temperature Management from Theory to Practice | TITLE |
|  | Bailey et al., 2019 | Integrating Fat Embolism Syndrome Scoring Indices in Sickle Cell Disease: A Practice Management Review | TITLE |
|  | Bardale et al., 2011 | Diagnosis of sickle cell disease at autopsy: A case report | TITLE |
|  | Bartram et al., 2010 | Outcome of children with sickle cell disease admitted to intensive care - A single institution experience | TITLE |
|  | Ballenberger et al., 2023 | ACUTE DECOMPENSATED PULMONARY HYPERTENSION AND RIGHT HEART FAILURE IN ACUTE CHEST SYNDROME IN HEMOGLOBIN SC DISEASE | TITLE |
|  | Banjar et al., 2008 | Diagnosis and management of pulmonary arterial hypertension in the pediatric population (PH) | TITLE |
|  | Banks et al., 2014 | Pulmonary endarterectomy: Part II. Operation, anesthetic management, and postoperative care | TITLE |
|  | Baptista et al., 2021 | The emerging challenge of sickle cell nephropathy | TITLE |
|  | Baranga et al., 2023 | In Situ Pulmonary Arterial Thrombosis: Literature Review and Clinical Significance of a Distinct Entity | TITLE |
|  | Barbosa et al., 2014 | Assessment of Ventricular Function in Adults with Sickle Cell Disease: Role of Two-Dimensional Speckle-Tracking Strain | TITLE |
|  | Badawy et al., 2016 | Assessing cardiac and liver iron overload in chronically transfused patients with sickle cell disease | ABSTRACT |
|  | Badawyt al., 2018 | Exercise capacity and clinical outcomes in adults followed in the Cooperative Study of Sickle Cell Disease (CSSCD) | ABSTRACT |
|  | Bah et al., 2006 | Renal diseases--morbidity and mortality in Nephrology Service, National Hospital Donka | TITLE |
|  | Balanchivadze et al., 2020 | Impact of COVID-19 Infection on 24 Patients with Sickle Cell Disease. One Center Urban Experience, Detroit, MI, USA | TITLE |
|  | Barbosa et al., 2015 | Air pollution and children's health: sickle cell disease | Title |
|  | Barriteau et al., 2022 | Cerebral and skeletal muscle tissue oxygenation during exercise challenge in children and young adults with sickle cell anaemia | TITLE |
|  | Batra et al., 2002 | Cardiac abnormalities in children with sickle cell anemia | ABSTRACT |
|  | Batnyam et al., 2021 | Failure of Complete Endothelialization of a Watchman Device 3 Years Post-Implantation | TITLE |
|  | Bayer et al., 2019 | Etiology and Outcomes of Thrombotic Microangiopathies | TITLE |
|  | Bayramoğlu et al., 2013 | Arterial stiffness and pulse wave reflection in young adult heterozygous sickle cell carriers | TITLE |
|  | Bariola et al., 2021 | Impact of Bamlanivimab Monoclonal Antibody Treatment on Hospitalization and Mortality among Nonhospitalized Adults with Severe Acute Respiratory Syndrome Coronavirus 2 Infection | TITLE |
|  | Barnett et al., 2008 | Pulmonary hypertension: an increasingly recognized complication of hereditary hemolytic anemias and HIV infection | ABSTRACT |
|  | Barnett et al., 2006 | Sildenafil in the treatment of pulmonary hypertension | ABSTRACT |
|  | Barriteau et al., 2022 | Practical Guidance for the Use of Voxelotor in the Management of Sickle Cell Disease | TITLE |
|  | Barst et al., 2010 | Predictors of six-minute walk distance in adults with sickle cell anemia in the walk-PHaSST study | ABSTRACT |
|  | Barst et al., 2010 | Exercise capacity and haemodynamics in patients with sickle cell disease with pulmonary hypertension treated with bosentan: Results of the ASSET studies | ABSTRACT |
|  | Barst et al., 2009 | Conventional and targeted medical therapies | ABSTRACT |
|  | Bartolucci et al., 2023 | Sickle cell disease hemolysis investigations and targeted therapeutics prospects | TITLE |
|  | Bas et al., 2023 | Pulmonary Extramedullary Hematopoiesis Presented as Massive Hemoptysis, A Rare Entity: A Case Report | TITLE |
|  | Bartolucci et al., 2012 | Clinical management of adult sickle-cell disease | TITLE |
|  | Bartolucci et al., 2016 | Six months of hydroxyurea reduces albuminuria in patients with sickle cell disease | TITLE |
|  | Bartolucci et al., 2014 | Chronic complications of sickle cell disease | TITLE |
|  | Baydar et al., 2019 | Riociguat use in a patient with sickle cell anemia related chronic thromboembolic pulmonary hypertension | TITLE |
|  | Bayés et al., 2003 | Gateways to clinical trials | TITLE |
|  | Baysal et al., 2002 | Nitric oxide II: Therapeutic uses and clinical applications | TITLE |
|  | Bebic et al., 2022 | Respiratory physiology at high altitude and considerations for pediatric patients | TITLE |
|  | Bedőcs et al., 2020 | The Critical Choice of Animal Models in Nanomedicine Safety Assessment: A Lesson Learned From Hemoglobin-Based Oxygen Carriers | TITLE |
|  | Beers van et al., 2006 | Glycocalyx Perturbation in Patients with Sickle Cell Disease: Association with Disease Morbidity | TITLE |
|  | Beers van et al., 2010 | Low mortality in sickle cell patient cohort with elevated tricuspid regurgitant jet flow velocity | ABSTRACT |
|  | Belisário et al., 2020 | Association of HIV infection with clinical and laboratory characteristics of sickle cell disease | TITLE |
|  | Belisário et al., 2019 | Sickle cell disease nephropathy: an update on risk factors and potential biomarkers in pediatric patients | TITLE |
|  | Bedirianet al., 2018 | Left ventricular structural and functional changes evaluated by echocardiography and two-dimensional strain in patients with sickle cell disease | ABSTRACT |
|  | Bedricket al., 2022 | Fertility preservation for pediatric patients with hemoglobinopathies: Multidisciplinary counseling needed to optimize outcomes | TITLE |
|  | Belcher et al., 2013 | MP4CO, a pegylated hemoglobin saturated with carbon monoxide, is a modulator of HO-1, inflammation, and vaso-occlusion in transgenic sickle mice | TITLE |
|  | Belcik et al., 2017 | Augmentation of Muscle Blood Flow by Ultrasound Cavitation Is Mediated by ATP and Purinergic Signaling | TITLE |
|  | Belferet al., 2014 | A GCH1 haplotype confers sex-specific susceptibility to pain crises and altered endothelial function in adults with sickle cell anemia | TITLE |
|  | Bellet al., 2016 | Lifetime Risk of Venous Thromboembolism in Two Cohort Studies | TITLE |
|  | Bello et al., 2017 | Sickle cell trait is not associated with an increased risk of heart failure or abnormalities of cardiac structure and function | Abstract |
|  | Berdoukas et al., 2005 | Lack of correlation between iron overload cardiac dysfunction and needle liver biopsy iron concentration | TITLE |
|  | Berg et al., 2005 | Fetal echocardiographic evaluation of atrial morphology and the prediction of laterality in cases of heterotaxy syndromes | ABSTRACT |
|  | Bernard et al., 2010 | Pediatric Arterial Ischemic Stroke | TITLE |
|  | Belisário et al., 2020 | Evidence for interactions between inflammatory markers and renin-angiotensin system molecules in the occurrence of albuminuria in children with sickle cell anemia | TITLE |
|  | Belveyre et al., 2019 | Spontaneous breathing during extracorporeal membrane oxygenation treatment of sickle cell disease acute chest syndrome | TITLE |
|  | Beninati et al., 2019 | The natural progression of pulmonary hypertension and sickle cell disease in pregnancy | TITLE |
|  | Benites et al., 2017 | Echocardiografic Abnormalities in Patients with Sickle Cell/β-Thalassemia Do Not Depend on the β-Thalassemia Phenotype | ABSTRACT |
|  | Benites et al., 2019 | An update on arginine in sickle cell disease | TITLE |
|  | Bennett et al., 2012 | Tobacco smoke exposure and stroke risk in children with sickle cell disease | TITLE |
|  | Bennewitz et al., 2020 | P-selectin-deficient mice to study pathophysiology of sickle cell disease | TITLE |
|  | Benza et al., 2008 | Pulmonary hypertension associated with sickle cell disease: Pathophysiology and rationale for treatment | ABSTRACT |
|  | Berdoukas et al., 2019 | Evaluation and progress of patients with significant hemoglobinopathies in Greek patients who live outside of athens | TITLE |
|  | Bereal-Williams et al., 2012 | Atorvastatin reduces serum cholesterol and triglycerides with limited improvement in vascular function in adults with sickle cell anemia | TITLE |
|  | Bernaudin et al., 2009 | Prevalence and risk factors of elevated tricuspid regurgitant jet velocity in children with sickle cell disease: Association with age, hemolysis, oxygen saturation and CD36 deficiency | ABSTRACT |
|  | Bernaudin et al., 2017 | Allogeneic/Matched Related Transplantation for β-Thalassemia and Sickle Cell Anemia | Title |
|  | Beshir et al., 2022 | ASSESSMENT OF CYSTATIN C IN PEDIATRIC SICKLE CELL DISEASE AND B-THALASSEMIA AS A MARKER OF SUBCLINICAL CARDIOVASCULAR DYSFUNCTION: A CASE-CONTROL STUDY | ABSTRACT |
|  | Bhaskar et al., 2021 | Susceptibility to vascular complications in sickle cell anemia patients is associated with intron 4a/b polymorphism of the NOS3 gene: A meta-analysis | TITLE |
|  | Bhatia et al., 2013 | Reduced toxicity conditioning (RTC) followed by matched sibling allogeneic stem cell transplantation in symptomatic and asymptomatic patients with sickle cell disease results in stable donor engraftment with with minimal complications | TITLE |
|  | Blyden et al., 2018 | Case series of patients with severe sickle cell disease treated with voxelotor (GBT440) by compassionate access | TITLE |
|  | Bhatraju et al., 2015 | Inhaled nitric oxide: Current clinical concepts | TITLE |
|  | Bhatt et al., 2016 | Mediastinal lymphadenopathy in homozygous sickle cell disease: An unusual diagnosis | TITLE |
|  | Bhor et al., 2018 | Clinical burden and management of sickle cell disease among pediatric patients | TITLE |
|  | Bhor et al., 2018 | Relationship between pain crisis and life-threatening complications in sickle cell disease: A retrospective claims based study | TITLE |
|  | Biassi et al., 2018 | MicroRNA and severity of sickle cell anemia | ABSTRACT |
|  | Biassi et al., 2022 | miRNA profile and disease severity in patients with sickle cell anemia | ABSTRACT |
|  | Biemond et al., 2017 | Predictive value of gradual change in renal function on vasculopathy related complications and mortality in patients with sickle cell disease | TITLE |
|  | Bigna et al., 2017 | Prevalence and etiologies of pulmonary hypertension in Africa: A systematic review and meta-analysis | ABSTRACT |
|  | Biller et al., 2017 | Red cell exchange: A concise review of indications and management | TITLE |
|  | Bindu et al., 2022 | Plasma protein precipitation and comparative analysis of Sickle Cell Disease (SCD) samples using SDS-PAGE | TITLE |
|  | Black et al., 2018 | Pilot results from an integrated pediatric pulmonary clinic on outcomes in children with sickle cell disease | Title |
|  | Bliddal et al., 2020 | Validation of a comorbidity index for use in obstetric patients: A nationwide cohort study | TITLE |
|  | Bodmer et al., 2023 | Obstructive Bronchial Fibrin Cast Formation in COVID-19 Severe Respiratory Failure | TITLE |
|  | Borst et al., 2000 | The hammer, the sickle, and the scalpel: A cardiac surgeon's view of Eastern Europe | TITLE |
|  | Bloom et al., 2009 | Lung function tests in sickle cell patients | TITLE |
|  | Bloomfield et al., 2012 | Conditions that predispose to pulmonary hypertension and right heart failure in persons exposed to household air pollution in LMIC | TITLE |
|  | Blum et al., 2011 | Pulmonary hypertension in sickle cell disease | ABSTRACT |
|  | Bluro et al., 2018 | Pulmonary Arterial Hypertension | ABSTRACT |
|  | Ben Ameur et al., 2016 | The emergent use of Cardiopulmonary bypass and extracorporeal membrane oxygenator in a child with sickle cell disease | TITLE |
|  | Bhatia et al., 2014 | Reduced toxicity, myeloablative conditioning with BU, fludarabine, alemtuzumab and SCT from sibling donors in children with sickle cell disease | TITLE |
|  | Bhatt et al., 2007 | Perioperative management of sickle cell disease in paediatric cardiac surgery | TITLE |
|  | Bhattacharya et al., 2022 | Incorporating neglected non-communicable diseases into the national health program-A review | TITLE |
|  | Bhat et al., 2019 | Purulent Pericarditis in Sickle Cell Disease Due to Streptococcus agalactiae; a Unique Case Report and Literature Review | TITLE |
|  | Biko et al., 2021 | Imaging of children with COVID-19: experience from a tertiary children’s hospital in the United States | TITLE |
|  | Bishnoiet al., 2023 | Modifying priming techniques in cardiopulmonary bypass circuit in known case of sickle-cell trait undergoing open heart surgery | TITLE |
|  | Bishburg et al., 2008 | Abiotrophia spp. and Staphylococcus epidermidis endocarditis treated with daptomycin | TITLE |
|  | Biswas et al., 2013 | Two- and three-dimensional speckle tracking echocardiography: Clinical applications and future directions | ABSTRACT |
|  | Blacket al., 2020 | Feasibility and preliminary outcomes of an integrated pediatric sickle cell disease and pulmonary care clinic for children with sickle cell disease | Title |
|  | Blackburnt al., 2018 | Unique Contribution of Haptoglobin and Haptoglobin Genotype in Aneurysmal Subarachnoid Hemorrhage | TITLE |
|  | Blaket al., 2013 | Considerations for Care: Management of Asthma in the Child with Sickle Cell Disease | TITLE |
|  | Blancet al., 2012 | Right ventricular systolic strain is altered in children with sickle cell disease | ABSTRACT |
|  | Blinder et al., 2013 | Age-related treatment patterns in sickle cell disease patients and the associated sickle cell complications and healthcare costs | TITLE |
|  | Boateng et al., 2019 | Red blood cell alloimmunization and minor red blood cell antigen phenotypes in transfused Ghanaian patients with sickle cell disease | TITLE |
|  | Bocchieri et al., 2010 | Exchange transfusion before cardiopulmonary bypass in sickle cell disease | TITLE |
|  | Bogui et al., 2013 | The PhysioFlow thoracic impedancemeter is not valid for the measurements of cardiac hemodynamic parameters in chronic anemic patients | TITLE |
|  | Bokov et al., 2020 | Salbutamol Worsens the Autonomic Nervous System Dysfunction of Children With Sickle Cell Disease | TITLE |
|  | Bollacheet al., 2016 | Abnormalities in aortic properties: a potential link between left ventricular diastolic function and ventricular-aortic coupling in sickle cell disease | TITLE |
|  | Bonnema et al., 2010 | Contraception choices in women with underlying medical conditions | TITLE |
|  | Bomhard et al., 2013 | Hypoxaemia affects male reproduction: A case study of how to differentiate between primary and secondary hypoxic testicular toxicity due to chemical exposure | TITLE |
|  | Boudreaux et al., 2022 | Addressing severe chronic NCDs across Africa: measuring demand for the Package of Essential Non-communicable Disease Interventions-Plus (PEN-Plus) | TITLE |
|  | Bollache et al., 2016 | Abnormalities in aortic properties: a potential link between left ventricular diastolic function and ventricular—aortic coupling in sickle cell disease | TITLE |
|  | Bollache et al., 2015 | Left ventricular-aortic coupling in sickle cell disease underlies diastolic dysfunction | TITLE |
|  | Bortolotti et al., 2022 | A Suspected Case of Cerebral Fat Embolism Triggering a Drug-resistant Status Epilepticus in a HbS/β+-Thalassaemia Patient | TITLE |
|  | Borgna-Pignatti et al., 2007 | Modern treatment of thalassaemia intermedia | TITLE |
|  | Bowers et al., 2021 | Management of Acute Pulmonary Embolism in a Patient with Sickle Cell Anemia Using Catheter-Directed Thrombolysis | TITLE |
|  | Bratis et al., 2013 | Abnormal myocardial perfusion-fibrosis pattern in sickle cell disease assessed by cardiac magnetic resonance imaging | TITLE |
|  | Boyd et al., 2009 | Lower airway obstruction is associated with increased morbidity in children with sickle cell disease | TITLE |
|  | Brousse et al., 2020 | One-Fifth of Children with Sickle Cell Anemia Show Exercise-Induced Hemoglobin Desaturation: Rate of Perceived Exertion and Role of Blood Rheology | TITLE |
|  | Bruzoni et al., 2011 | Single-site umbilical laparoscopic splenectomy | TITLE |
|  | Bryant et al., 2005 | Asthma in the pediatric sickle cell patient with acute chest syndrome | TITLE |
|  | Brewin et al., 2020 | Catheter associated thromboses in patients with sickle cell anaemia and dual lumen Vortex apheresis ports are common and can be clinically asymptomatic | TITLE |
|  | Brewin et al., 2021 | Genome wide association study of silent cerebral infarction in sickle cell disease (HbSS and HbSC) | TITLE |
|  | Brobeck et al., 2005 | Pediatric stroke: The child is not merely a small adult | TITLE |
|  | Buckner et al., 2014 | Does hydroxyurea prevent pulmonary complications of sickle cell disease? | ABSTRACT |
|  | Bodez et al., 2016 | Prognostic value of right ventricular systolic function in cardiac amyloidosis | TITLE |
|  | Böger et al., 2005 | L-arginine improves vascular function by overcoming the deleterious effects of ADMA, a novel cardiovascular risk factor | TITLE |
|  | Boissier et al., 2019 | Extracorporeal Life Support for Severe Acute Chest Syndrome in Adult Sickle Cell Disease: A Preliminary Report | TITLE |
|  | Bokov et al., 2020 | Altered pulmonary capillary blood volume in childhood sickle cell disease | TITLE |
|  | Bonini-Domingos et al., 2016 | Compound heterozygosity for hemoglobin S and D: what do we need to know? | TITLE |
|  | Boonyawat et al., 2014 | Hydroxyurea and colonic ulcers: A case report | TITLE |
|  | Borrelli et al., 2023 | Diagnosis of Paediatric Obstructive Sleep-Disordered Breathing beyond Polysomnography | TITLE |
|  | Bossley et al., 2022 | Sickle Cell Disease-Pulmonary Management | TITLE |
|  | Bourg et al., 2018 | Improvement of the six-minute walk test in children with sickle cell disease over time | ABSTRACT |
|  | Boussaada et al., 2004 | Pulmonary hypertension in sickle cell anemia. A case report | ABSTRACT |
|  | Boussaada et al., 2004 | Pulmonary hypertension in heterozygous sickle hemoglobinopathy about one case | Title |
|  | Bowers et al., 2015 | Interventions for treating pulmonary hypertension in people with sickle cell disease or thalassaemia | TITLE |
|  | Boyd et al., 2006 | The outcomes of sickle cell disease in adulthood are clear, but the origins and progression of sickle cell anemia-induced problems in the heart and lung in childhood are not | TITLE |
|  | Boye-Doe et al., 2020 | The Grndad Registry: Contemporary Natural History Data and an Analysis of Real-World Patterns of Use and Limitations of Disease Modifying Therapy in Adults with SCD | TITLE |
|  | Boyer et al., 2016 | ATS core curriculum 2016: Part III. Pediatric pulmonary medicine | TITLE |
|  | Bradley et al., 2021 | ACHD-Specific Risk Factors for Severe COVID-19 | TITLE |
|  | Brady et al., 2011 | Improving access to pulmonary care for children with sickle cell disease | ABSTRACT |
|  | Brady et al., 2009 | Screening for sickle cell lung disease in a pediatric sickle cell clinic | ABSTRACT |
|  | Braga et al., 2015 | Altered left ventricular twist is associated with clinical severity in adults and adolescents with homozygous sickle cell anemia | ABSTRACT |
|  | Brandow et al., 2009 | Hydroxyurea in Children with Sickle Cell Disease: Practice Patterns and Barriers to Utilization | ABSTRACT |
|  | Brandow et al., 2022 | Advances in the diagnosis and treatment of sickle cell disease | TITLE |
|  | Braun et al., 2010 | Emergence of orphan drugs in the United States: A quantitative assessment of the first 25 years | TITLE |
|  | Brider et al., 2021 | THE HEMATOLOGICAL AMBIGUITY: ACUTE ONSET SEVERE PULMONARY HYPERTENSION MIMICKING ACUTE PULMONARY EMBOLISM | TITLE |
|  | Brill et al., 2021 | Multiple facets of venous thrombosis | TITLE |
|  | Brittain et al., 2014 | Ironing out placenta growth factor | TITLE |
|  | Brittain et al., 2010 | Placenta growth factor in sickle cell disease: association with hemolysis and inflammation | TITLE |
|  | Broderick et al., 2012 | Priapism and Sickle-Cell Anemia: Diagnosis and Nonsurgical Therapy | Title |
|  | Brousse et al., 2014 | The spleen and sickle cell disease: The sick(led) spleen | TITLE |
|  | Brousse et al., 2014 | Management of sickle cell disease in the community | TITLE |
|  | Brown et al., 2017 | Aworld of diagnostic considerations for ulcers in children with sickle cell disease | TITLE |
|  | Brugnara et al., 2010 | ASH 2009 meeting report - Top 10 clinically oriented abstracts in sickle cell disease | TITLE |
|  | Brzoska et al., 2019 | The role of platelets in sickle cell disease | TITLE |
|  | Buchanan et al., 2004 | Sickle cell disease | TITLE |
|  | Buchanan et al., 2005 | Opioid selection during sickle cell pain crisis and its impact on the development of acute chest syndrome | TITLE |
|  | Buehler et al., 2010 | Toxicological consequences of extracellular hemoglobin: Biochemical and physiological perspectives | TITLE |
|  | Buehler et al., 2020 | Haptoglobin Therapeutics and Compartmentalization of Cell-Free Hemoglobin Toxicity | TITLE |
|  | Buehler et al., 2021 | Hemopexin dosing improves cardiopulmonary dysfunction in murine sickle cell disease | TITLE |
|  | Buehler et al., 2021 | Murine models of sickle cell disease and beta-thalassemia demonstrate pulmonary hypertension with distinctive features | TITLE |
|  | Bueno et al., 2013 | Nitrite signaling in pulmonary hypertension: Mechanisms of bioactivation, signaling, and therapeutics | TITLE |
|  | Bunn et al., 2010 | Pulmonary hypertension and nitric oxide depletion in sickle cell disease | ABSTRACT |
|  | Burns et al., 2019 | Autosplenectomy in a Patient with Paroxysmal Nocturnal Hemoglobinuria (PNH) | TITLE |
|  | Butrous et al., 2014 | The role of phosphodiesterase inhibitors in the management of pulmonary vascular diseases | TITLE |
|  | Byrne et al., 2010 | The importance of antibody detection and identification in the chronically transfused patient | TITLE |
|  | Byars et al., 2023 | Saved by point of care echo | TITLE |
|  | Caboot et al., 2008 | Pulmonary complications of sickle cell disease in children | Title |
|  | Cabrita et al., 2009 | Survival of patients with pulmonary hypertension and sickle cell disease in the UK | TITLE |
|  | Cabrita et al., 2012 | Anomalous insertion of the papillary muscle in a patient with sickle cell disease: A normal variant with no left ventricular outflow obstruction | TITLE |
|  | Cai et al., 2022 | Risk and protective factors for severe COVID-19 infection in a cohort of patients with sickle cell disease | TITLE |
|  | Cairo et al., 2018 | Significantly Improved Long Term Health Related Quality of Life (HRQL) and Neurocognition Following Familial Haploidentical Stem Cell Transplantation (HISCT) Utilizing CD34 Enrichment and Mononuclear (CD3) Addback in High Risk Patients with Sickle Cell Disease (SCD) | TITLE |
|  | Callaghan et al., 2022 | Trial in progress: The randomized, doubleblind, placebo-controlled phase 2a crosswalk-c trial evaluating the efficacy of crovalimab as adjunct treatment in the prevention of vasoocclusive episodes (VOEs) in patients (pts) with sickle cell disease (SCD) | TITLE |
|  | Callahan et al., 2002 | What respiratory problems occur in sickle cell anemia? | TITLE |
|  | Cabaret et al., 2013 | Multifocal osteoarticular infection caused by Salmonella non typhi in a child with sickle cell disease | TITLE |
|  | Capoccia et al., 2020 | Delayed repair of aortic dissection in sickle cell anaemia as a combined cardiac and vascular surgical approach | TITLE |
|  | Capoccia et al., 2021 | Cardiac and Vascular Surgeons for the Treatment of Aortic Disease: A Successful Partnership for Decision-Making and Management of Complex Cases | TITLE |
|  | Casa et al., 2013 | The inter-association task force for preventing sudden death in secondary school athletics programs: Best-practices recommendations | TITLE |
|  | Casas et al., 2003 | Sickle-cell disease: A review of the 5-year experience of a single institution | ABSTRACT |
|  | Cancado et al., 2018 | Assessment of liver and cardiac iron overload using MRI in patients with chronic anemias in Latin American countries: results from ASIMILA study* | TITLE |
|  | Carles et al., 2001 | Sicle cell anemia and pregnancy: Considerations on systematic prophylactic transfusion | TITLE |
|  | Callaway et al., 2023 | Personalized Exercise Intervention for Older Adults with Sickle Cell Disease: The Sicklefit Study | TITLE |
|  | Calvert et al., 2009 | Myocardial protection by nitrite | TITLE |
|  | Calvert et al., 2010 | Clinical translation of nitrite therapy for cardiovascular diseases | Title |
|  | Campbell et al., 2020 | The economic burden of end-organ damage among medicaid patients with sickle cell disease in the United States: A population-based longitudinal claims study | TITLE |
|  | Campbell et al., 2009 | Prospective evaluation of haemoglobin oxygen saturation at rest and after exercise in paediatric sickle cell disease patients | TITLE |
|  | Campbell et al., 2007 | Prospective Evaluation of the Prevalence of Elevated Tricuspid Regurgitant Jet Velocity and Associated Clinical and Echocardiographic Factors in Children and Adolescents with Sickle Cell Disease | ABSTRACT |
|  | Campbell et al., 2010 | Evaluation of Transcranial Doppler Velocities (TCD) and it's relationship to tricuspid regurgitation velocity, hemolysis, blood pressure, and other hematologic parameters in non-transfused pediatric sickle cell disease patients | TITLE |
|  | Campbell et al., 2005 | Adverse Events in Sickle Cell Patients with Nocturnal Hypoxia | TITLE |
|  | Cancado et al., 2008 | Pulmonary Hypertension as a Risk Factor for Death in Patients with Sickle Cell Anemia | ABSTRACT |
|  | Cannas et al., 2017 | Hydroxycarbamine: from an Old Drug Used in Malignant Hemopathies to a Current Standard in Sickle Cell Disease | TITLE |
|  | Cao et al., 2022 | Prevalence of the number of pre-gestational diagnoses and trends in the United States in 2006 and 2016 | TITLE |
|  | Caplin et al., 2012 | Endogenous nitric oxide synthase inhibitors in the biology of disease: Markers, mediators, and regulators? | TITLE |
|  | Carrara et al., 2023 | Factors related to the readiness of Brazilian chronic pediatric patients to transition to care in adult clinics | TITLE |
|  | Carroll et al., 2013 | Depressive symptoms and disease management selfefficacy, not catastrophizing, predict pain interference independent of pain severity in sickle cell disease: Results from the baseline IMPORT study | TITLE |
|  | Carter et al., 2014 | Euvolemic automated transfusion of red cells in volume-sensitive patients | TITLE |
|  | Carthan et al., 2011 | Hemolysis-associated pulmonary hypertension | Abstract |
|  | Carvalho et al., 2017 | Evaluation of Alpha-1 Antitrypsin Levels and <i>SERPINA1</i> Gene Polymorphisms in Sickle Cell Disease | TITLE |
|  | Carvalho et al., 2017 | Contraceptive practices in women with sickle-cell disease | TITLE |
|  | Castonguay et al., 2021 | COVID-19 and Sickle Cell Disease in the Province of Quebec: Morbidity and Mortality Rates Derived from the Provincial Registry | TITLE |
|  | Castro et al., 2010 | Pulmonar hypertension in children with sickle cell disease living at high altitude: A single center experience in Bogota-Colombia | ABSTRACT |
|  | Castro et al., 2005 | Pulmonary hypertension in sickle cell disease: Mechanisms, diagnosis, and management | TITLE |
|  | Castro et al., 2003 | Pulmonary hypertension in sickle cell disease: Cardiac catheterization results and survival | ABSTRACT |
|  | Castro et al., 2011 | Pulmonary hypertension in sickle cell disease | ABSTRACT |
|  | Castro et al., 2010 | Serum B12 levels in children with sickle cell disease are lower than in healthy control subjects | TITLE |
|  | Callahan et al., 2002 | Cardiopulmonary responses to exercise in women with sickle cell anemia | ABSTRACT |
|  | Callebaut et al., 2005 | Origin, fate, and function of the components of the avian germ disc region and early blastoderm: role of ooplasmic determinants | TITLE |
|  | Camcıoğluet al., 2015 | The Role of Inspiratory Muscle Training in Sickle Cell Anemia Related Pulmonary Damage due to Recurrent Acute Chest Syndrome Attacks | TITLE |
|  | Carstens et al., 2019 | Clinical relevance of pulmonary vasculature involvement in sickle cell disease | ABSTRACT |
|  | Castro et al., 2005 | Pulmonary hypertension in sickle cell disease: mechanisms, diagnosis, and management | TITLE |
|  | Cecchini et al., 2016 | Pulmonary Vascular Dysfunction and Cor Pulmonale During Acute Respiratory Distress Syndrome in Sicklers | TITLE |
|  | Ceglie et al., 2019 | Gender-Related Differences in Sickle Cell Disease in a Pediatric Cohort: A Single-Center Retrospective Study | ABSTRACT |
|  | Chabott al., 2008 | Mitral valve replacement in a patient with sickle cell disease using perioperative exchange transfusion | TITLE |
|  | Chacon-Portillo et al., 2020 | Sickle Cell-Related Complications in Patients Undergoing Cardiopulmonary Bypass | Title |
|  | Chalacheva et al., 2015 | Autonomic responses to cold face stimulation in sickle cell disease: a time-varying model analysis | TITLE |
|  | Chalacheva et al., 2019 | Sickle Cell Disease Subjects Have a Distinct Abnormal Autonomic Phenotype Characterized by Peripheral Vasoconstriction With Blunted Cardiac Response to Head-Up Tilt | TITLE |
|  | Changet al., 2006 | Correction of the sickle cell mutation in embryonic stem cells | TITLE |
|  | Charlot et al., 2015 | Physical activity level is not a determinant of autonomic nervous system activity and clinical severity in children/adolescents with sickle cell anemia: A pilot study | TITLE |
|  | Charrin et al., 2015 | Oxidative stress is decreased in physically active sickle cell SAD mice | TITLE |
|  | Chaudry et al., 2013 | The impact of sickle cell disease on exercise capacity in children | ABSTRACT |
|  | Chen et al., 2021 | Anesthetic Considerations for Percutaneous and Open Right Atrial Thrombectomy in a Hemoglobin SC Patient | TITLE |
|  | Cheminet et al., 2022 | Acute chest syndrome in adult sickle cell patients | TITLE |
|  | Chen et al., 2020 | Pulmonary disease burden in Hispanic and non-Hispanic children with sickle cell disease | ABSTRACT |
|  | Chenik et al., 2022 | Evaluation of left ventricular systolic function in children with sickle cell anemia: contribution of 2D strain | TITLE |
|  | Castro et al., 2011 | Senicapoc trial results support the existence of different sub-phenotypes of sickle cell disease with possible drug-induced phenotypic shifts | TITLE |
|  | Castro et al., 2009 | Lower ferritin concentrations are associated with decreased hemolysis in sickle cell disease children without iron overload | TITLE |
|  | Caughey et al., 2015 | Albuminuria is associated with endothelial dysfunction in sickle cell disease | TITLE |
|  | Caughey et al., 2016 | Sickle Cardiomyopathy the Missing Forest in the Trees | TITLE |
|  | Caughey et al., 2021 | Editorial commentary: Sickle cell disease: Diagnosing the heart of the matter | Title |
|  | Cazenave et al., 2020 | Tubular Acidification Defect in Adults with Sickle Cell Disease | TITLE |
|  | Cecchini et al., 2015 | Sickle cell disease in the ICU | TITLE |
|  | Ceci et al., 2014 | Multidisciplinary care in haemoglobinopathies | TITLE |
|  | Cela et al., 2017 | National registry of hemoglobinopathies in Spain (REPHem) | TITLE |
|  | Chacko et al., 2013 | Myocardial infarction in sickle cell disease: Use of translational imaging to diagnose an under-recognized problem | TITLE |
|  | Chacko et al., 2023 | Sickle Cell Disease (SCD) Leading to Pulmonary Arterial Hypertension (PAH) and Cholelithiasis (CL) | ABSTRACT |
|  | Chacra et al., 2022 | Sickle Cell Anemia as the First in the Hierarchy for Cardiac Alterations, Drives Attention to the Whole Spectrum of Hemolytic Anemias | TITLE |
|  | Chakravarty et al., 2008 | Pregnancy outcomes in systemic sclerosis, primary pulmonary hypertension, and sickle cell disease | TITLE |
|  | Chakravorty et al., 2015 | Sickle cell disease: a neglected chronic disease of increasing global health importance | TITLE |
|  | Chan et al., 2023 | Pulmonary hypertension screening in children with sickle cell disease | ABSTRACT |
|  | Chan et al., 2022 | Regarding: Pulmonary hypertension is associated with an increased incidence of NAFLD | TITLE |
|  | Chandrakala et al., 2013 | Hydroxycarbamide should be universal therapy for a sickler! | TITLE |
|  | Chang et al., 2018 | Genetic modifiers of severity in sickle cell disease | ABSTRACT |
|  | Chang et al., 2008 | Continuous inhaled nitric oxide therapy in a case of sickle cell disease with multiorgan involvement | TITLE |
|  | Chaouch et al., 2015 | Association between rs267196 and rs267201 of BMP6 gene and osteonecrosis among Sickle Cell Aneamia patients | TITLE |
|  | Chapdelaine et al., 2008 | Cardiopulmonary Testing and Pulmonary Hypertension in Patients with Sickle Cell Anemia | ABSTRACT |
|  | Chapusette et al., 2012 | Reduction of the six-minute walk distance in children with sickle cell disease is correlated with silent infarct: Results from a cross-sectional evaluation in a single center in Belgium | ABSTRACT |
|  | Charbonney et al., 2006 | Acute chest syndrome in sickle cell disease and acute respiratory distress syndrome - From pathophysiology to therapy | TITLE |
|  | Chaturvedi et al., 2021 | A case of sideroblastic anemia masked by underlying sickle cell disease in a young African American patient | TITLE |
|  | Chaturvedi et al., 2016 | Evolution of sickle cell disease from a life-threatening disease of children to a chronic disease of adults: The last 40 years | ABSTRACT |
|  | Chaturvedi et al., 2018 | Clustering of end-organ disease and earlier mortality in adults with sickle cell disease: A retrospective-prospective cohort study | ABSTRACT |
|  | Chaturvedi et al., 2017 | Elevated tricuspid regurgitant jet velocity, reduced forced expiratory volume in 1 second, and mortality in adults with sickle cell disease | ABSTRACT |
|  | Chaudhary et al., 2023 | Fetomaternal Outcomes among Patients with Sickle Cell Disease: A Retrospective Study | TITLE |
|  | Chaurasia et al., 2023 | Prevalence of pulmonary hypertension in children with sickle cell disease | ABSTRACT |
|  | Chawla et al., 2019 | Evolution of the HGB-206 study design for evaluating lentiglobin gene therapy in sickle cell disease | TITLE |
|  | Cheesman et al., 2015 | Sickle cell disease: Symptoms, complications and management | TITLE |
|  | Chen et al., 2009 | Active von willebrand factor (VWF) in plasma of sickle cell patients is an indicator of disease severity | TITLE |
|  | Chen et al., 2008 | Active Von Willebrand Factor in Plasma of Sickle Cell Patients Is An Indicator for Severity of Sickle Cell Anemia | TITLE |
|  | Chen et al., 2023 | Prevalence and Time Trends of Oral Anticoagulant Utilization in Adults with Sickle Cell Disease | TITLE |
|  | Chueamuangphan et al., 2014 | Benefits of chronic blood transfusion in hemoglobin E/β thalassemia with pulmonary arterial hypertension |  |
|  | Chiadika et al., 2018 | Echocardiographic parameters to identify sickle cell patients with cardio-pathology | ABSTRACT |
|  | Chinawa et al., 2021 | The impact of body mass index on left ventricular mass index, right ventricular function, and cardiac dimension of children with sickle cell anemia in Enugu City | Title |
|  | Chirico et al., 2012 | Role of oxidative stress in the pathogenesis of sickle cell disease | TITLE |
|  | Chironi et al., 2009 | Endothelial microparticles in diseases | TITLE |
|  | Cho et al., 2011 | Regular long-term red blood cell transfusions for managing chronic chest complications in sickle cell disease | TITLE |
|  | Chonat et al., 2017 | Current standards of care and long term outcomes for thalassemia and sickle cell disease | TITLE |
|  | Chinawa et al., 2021 | Left ventricular function and cardiac valvar annular dimensions among children with sickle cell anemia compared to those with hemoglobin AA type in Enugu, Nigeria | TITLE |
|  | Chung et al., 2023 | Prevalence of Pediatric Masked Hypertension and Risk of Subclinical Cardiovascular Outcomes: A Systematic Review and Meta-Analysis | TITLE |
|  | Ciapponi et al., 2020 | The quality of clinical practice guidelines for preoperative care using the AGREE II instrument: A systematic review | TITLE |
|  | Chunget al., 2007 | Prodrugs for the treatment of neglected diseases | TITLE |
|  | Chunget al., 2020 | Cardiovascular Risk and Cardiovascular Health Behaviours in the Transition From Childhood to Adulthood | TITLE |
|  | Cipolotti et al., 2001 | Echocardiographic characteristics of patients with sickle cell anaemia in Sergipe, Brazil | ABSTRACT |
|  | Cirelli et al., 2018 | The Burden of Indirect Causes of Maternal Morbidity and Mortality in the Process of Obstetric Transition: A Cross-Sectional Multicenter Study | TITLE |
|  | Clarkeet al., 2022 | Catastrophic Neurological Complications in 2 Patients With Sickle Cell Disease and COVID-19 | TITLE |
|  | Cohen et al., 2015 | Sickle cell disease: wheeze or asthma? | TITLE |
|  | Cohen et al., 2013 | Environmental tobacco smoke and airway obstruction in children with sickle cell anemia | TITLE |
|  | Cohen et al., 2016 | Pattern of Lung Function Is Not Associated with Prior or Future Morbidity in Children with Sickle Cell Anemia | Title |
|  | Coller et al., 2005 | Leukocytosis and ischemic vascular disease morbidity and mortality: is it time to intervene? | TITLE |
|  | Cohen et al., 2022 | Pulmonary embolism in pediatric and adolescent patients with COVID-19 infection during the SARS-CoV-2 delta wave | TITLE |
|  | Constantine et al., 2017 | Recurrent Priapism Gone Wrong: ST-Elevation Myocardial Infarction and Cardiogenic Shock After Penile Corporal Phenylephrine Irrigation | TITLE |
|  | Courbebaisse et al., 2017 | Carboxy-terminal fragment of fibroblast growth factor 23 induces heart hypertrophy in sickle cell disease | TITLE |
|  | Colombatti et al., 2013 | Coagulation activation in children with sickle cell disease is associated with cerebral small vessel vasculopathy | TITLE |
|  | Colombatti et al., 2011 | Tricuspid regurgitant velocity elevation in a three-year old child with sickle cell anemia and recurrent acute chest syndromes reversed not by hydroxyurea but by bone marrow transplantation | ABSTRACT |
|  | Connes et al., 2006 | Oxygen uptake kinetics during heavy submaximal exercise: Effect of sickle cell trait with or without alpha-thalassemia | TITLE |
|  | Cooper et al., 2019 | Translating exercise benefits in sickle cell disease | TITLE |
|  | Chou et al., 2016 | Management of Patients with Sickle Cell Disease Using Transfusion Therapy: Guidelines and Complications | TITLE |
|  | Cintho Ozahata et al., 2019 | Clinical and Genetic Predictors of Priapism in Sickle Cell Disease: Results from the Recipient Epidemiology and Donor Evaluation Study III Brazil Cohort Study | TITLE |
|  | Cisneros et al., 2020 | Recent Advances in the Treatment of Sickle Cell Disease | TITLE |
|  | Cita et al., 2016 | Association of adenylyl cyclase 6 rs3730070 polymorphism and hemolytic level in patients with sickle cell anemia | TITLE |
|  | Claster et al., 2000 | Acute chest syndrome in sickle cell disease: Pathophysiology and management | TITLE |
|  | Claudino et al., 2012 | Sickling cells, cyclic nucleotides, and protein kinases: the pathophysiology of urogenital disorders in sickle cell anemia | Title |
|  | Close et al., 2009 | Effectiveness of imatinib therapy for a patient with sickle cell anemia and chronic myelocytic leukemia | TITLE |
|  | Coghlan et al., 2007 | Cardiac assessment of patients for haematopoietic stem cell transplantation | TITLE |
|  | Cohen et al., 2008 | Association of Pulmonary Hypertension with Sickle Cell Disease Subtypes | ABSTRACT |
|  | Cohen et al., 2010 | Improved access to pulmonary care for children with sickle cell disease | ABSTRACT |
|  | Cohen et al., 2011 | Recurrent, severe wheezing is associated with morbidity and mortality in adults with sickle cell disease | TITLE |
|  | Colella et al., 2012 | Elevation of hypercoagulability markers in hemoglobin SC disease | TITLE |
|  | Colella et al., 2013 | Increased Inflammatory Markers and Their Correlations To Clinical Complications In Hemoglobin SC Disease | TITLE |
|  | Colombatti et al., 2012 | Extensive coagulation profile in children with sickle cell disease and its role in cerebral micro-vasculopathy | TITLE |
|  | Colombatti et al., 2009 | Sickle cell disease affected children: An increasing group of children born in Europe from immigrant families | TITLE |
|  | Colombatti et al., 2010 | Pulmonary hypertension in young children with sickle cell disease: When does it start? Survey among immigrant patients in North-East Italy | ABSTRACT |
|  | Colpo et al., 2016 | Prophylactic red blood cell exchange in allogeneic stem cell transplantation in pediatric patients with sickle cell disease: A single center experience | TITLE |
|  | Cornier et al., 2004 | Phenotype characterization and natural history of spondylothoracic dysplasia syndrome: a series of 27 new cases | TITLE |
|  | Covi et al., 2018 | Changes in Bi-ventricular Function After Hematopoietic Stem Cell Transplant as Assessed by Speckle Tracking Echocardiography | TITLE |
|  | Crook et al., 2019 | Falsely low activated clotting time measured in a patient with sickle cell trait | TITLE |
|  | Connes et al., 2016 | The role of blood rheology in sickle cell disease | Title |
|  | Connes et al., 2013 | Autonomic nervous system dysfunction: Implication in sickle cell disease | TITLE |
|  | Connes et al., 2014 | Haemolysis and abnormal haemorheology in sickle cell anaemia | TITLE |
|  | Connes et al., 2011 | Exercise limitation, exercise testing and exercise recommendations in sickle cell anemia | TITLE |
|  | Connor et al., 2018 | Brief topical sodium nitrite and its impact on the quality of life in patients with sickle leg ulcers | TITLE |
|  | Connor et al., 2008 | Pulmonary hypertension in children with Evans syndrome | TITLE |
|  | Conran et al., 2014 | Intravascular hemolysis: A disease mechanism not to be ignored | TITLE |
|  | Conran et al., 2018 | Inflammation in sickle cell disease | TITLE |
|  | Conran et al., 2009 | Hemoglobin disorders and endothelial cell interactions | TITLE |
|  | Conran et al., 2022 | Sickle Cell Disease and Oxidative Stress: Implications for a Role of Diminished Endothelial SOD2 in Pulmonary Complications | TITLE |
|  | Conran et al., 2009 | Newer aspects of the pathophysiology of sickle cell disease vaso-occlusion | TITLE |
|  | Conran et al., 2019 | cGMP modulation therapeutics for sickle cell disease | TITLE |
|  | Cook et al., 2022 | Combined preoperative plasma exchange and red blood cell exchange transfusion in a renal transplant patient with protein S deficiency and hemoglobin SC disease | TITLE |
|  | Cooper et al., 2019 | Pharmacological interventions for painful sickle cell vaso-occlusive crises in adults | TITLE |
|  | Corns et al., 2009 | Free arginase-I in human pulmonary microvascular endothelial cell culture leads to endothelial dysfunction and increased reactive oxygen species | TITLE |
|  | Correale et al., 2012 | Long-term treatment with high-dose of sildenafil in a thalassemic patient with pulmonary hypertension | TITLE |
|  | Cortese-Krott et al., 2018 | Identification of a soluble guanylate cyclase in RBCs: preserved activity in patients with. coronary artery disease | TITLE |
|  | Costa et al., 2016 | Clinical manifestations and treatment of adult sickle cell disease | Title |
|  | Couronnéet al., 2009 | Hodgkin lymphoma in a sickle cell anaemia child treated with hydroxyurea | TITLE |
|  | Cox et al., 2018 | Ready-to-use food supplement, with or without arginine and citrulline, with daily chloroquine in Tanzanian children with sickle-cell disease: a double-blind, random order crossover trial | TITLE |
|  | Cox et al., 2020 | L-Glutamine in sickle cell disease | TITLE |
|  | Cramer-Bour et al., 2021 | Long-term tolerability of phosphodiesterase-5 inhibitors in pulmonary hypertension of sickle cell disease | ABSTRACT |
|  | Crane et al., 2011 | Priapism in sickle cell anemia: emerging mechanistic understanding and better preventative strategies | TITLE |
|  | Crary et al., 2009 | Vascular complications after splenectomy for hematologic disorders | ABSTRACT |
|  | Crary et al., 2011 | Prevalence of pulmonary hypertension in hereditary spherocytosis | ABSTRACT |
|  | Crawford et al., 2017 | Management of sickle cell disease in patients undergoing cardiac surgery | TITLE |
|  | Creagh-Brown et al., 2009 | Bench-to-bedside review: Inhaled nitric oxide therapy in adults | TITLE |
|  | Cui et al., 2002 | Inhaled nitric oxide: Is systemic host defense at risk? | TITLE |
|  | Cumming et al., 2008 | Venous incompetence, poverty and lactate dehydrogenase in Jamaica are important predictors of leg ulceration in sickle cell anaemia | TITLE |
|  | Cunico et al., 2023 | Initiatives to promote access to medicines after publication of the Brazilian Policy on the Comprehensive Care of People with Rare Diseases | TITLE |
|  | Curran et al., 2020 | Perinatal Management of Bart's Hemoglobinopathy: Paradoxical Effects of Intrauterine, Transplacental, and Partial Exchange Transfusions | TITLE |
|  | Cuttica et al., 2011 | Pulmonary artery thrombosis: Another piece to the acute chest syndrome puzzle | TITLE |
|  | D’Alessandro et al., 2023 | Metabolic signatures of cardiorenal dysfunction in plasma from sickle cell patients as a function of therapeutic transfusion and hydroxyurea treatment | TITLE |
|  | da Guarda et al., 2020 | Investigation of Lipid Profile and Clinical Manifestations in SCA Children | ABSTRACT |
|  | Dagartzikas et al., 2002 | Cerebrovascular event, dilated cardiomyopathy, and pheochromocytoma | TITLE |
|  | Dahhan et al., 2015 | Myocardial infarction after physical exertion in a healthy young patient with coronary artery ectasia and sickle cell trait | TITLE |
|  | da Silva et al., 2023 | Exercise Capacity and Biomarkers Among Children and Adolescents With Sickle Cell Disease | ABSTRACT |
|  | Dabirian et al., 2017 | Cardiac Structural and Functional Changes Evaluated by Transthoracic and Tissue Doppler Echocardiography in Adult Patients with Sickle Cell Disease | ABSTRACT |
|  | Daiber, et al., 2016 | Virtual issue by COST Action BM1203 (EU-ROS) “Emerging concepts in redox biology and oxidative stress” | TITLE |
|  | Dakhallah et al., 2021 | COVID-19 in children and young adults with sickle cell disease in the province of Quebec | TITLE |
|  | Dallas et al., 2013 | Long-term outcome and evaluation of organ function in pediatric patients undergoing haploidentical and matched related hematopoietic cell transplantation for sickle cell disease | TITLE |
|  | Dalle-Donne et al., 2006 | Biomarkers of oxidative damage in human disease | TITLE |
|  | Damanhouri et al., 2015 | Clinical biomarkers in sickle cell disease | TITLE |
|  | Dandekar et al., 2017 | Hematopoietic Stem Cell Transplantation for the Pediatric Hematologist/Oncologist | Title |
|  | Dampier et al., 2010 | Initial experience with the improve trial-a phase III analgesic trial for hospitalized sickle cell painful episodes | TITLE |
|  | Dampier et al., 2010 | Health-related quality of life in children with sickle cell disease: A report from the Comprehensive Sickle Cell Centers Clinical Trial Consortium | TITLE |
|  | Dandara et al., 2016 | Precision medicine 2.0: The next wave of science | TITLE |
|  | Dang et al., 2005 | Myocardial injury or infarction associated with fat embolism in sickle cell disease: A report of three cases with survival | Title |
|  | Dang et al., 2005 | Bone marrow embolism in sickle cell disease: A review | TITLE |
|  | Daniel et al., 2011 | Sickle cell/β 0-thalassemia associated with the 1393 bp deletion can be associated with a severe phenotype | ABSTRACT |
|  | Daniels et al., 2010 | Are intracardiac shunts a risk factor for stroke in sickle cell disease? | TITLE |
|  | Danilov et al., 2011 | A novel ACE mutation (Arg532Trp) is associated with dramatic increases in blood ACE | TITLE |
|  | Darbari et al., 2011 | Markers of severe vaso-occlusive painful episodes in children and adolescents with sickle cell anemia | TITLE |
|  | Darbari et al., 2010 | Epidemiology and risk factors for pain in children and adolescent with sickle cell anemia | Title |
|  | Darbari et al., 2011 | Markers of severe vaso-occlusive painful episodes in children and adolescents with sickle cell anemia | TITLE |
|  | Darbari et al., 2008 | Severe vaso-occlusive episodes associated with use of systemic corticosteroids in patients with sickle cell disease | TITLE |
|  | Darbari et al., 2010 | Epidemiology of pain in sickle cell anemia and its association with a susceptibility marker in the gch1 gene | TITLE |
|  | Darbari et al., 2011 | Health care utilization for painful events is associated with early mortality in a contemporary population of adults with sickle cell anemia | TITLE |
|  | Darbari et al., 2006 | Circumstances of death in adult sickle cell disease patients | TITLE |
|  | Daaboulet al., 2011 | Anesthetic and cardiopulmonary bypass considerations for cardiac surgery in unique pediatric patient populations: sickle cell disease and cold agglutinin disease | TITLE |
|  | Daak et al., 2020 | Biochemical and therapeutic effects of Omega-3 fatty acids in sickle cell disease | TITLE |
|  | D'Alessandro et al., 2023 | In vivo evaluation of the effect of sickle cell hemoglobin S, C and therapeutic transfusion on erythrocyte metabolism and cardiorenal dysfunction | TITLE |
|  | D'Amico et al., 2021 | Definity, an affinity for painful crisis: a case series describing vaso-occlusive pain crises in sickle cell patients undergoing echocardiogram with Definity contrast | Title |
|  | Dast al., 2012 | Utility of impedance cardiography for the detection of hemodynamic changes in stable patients with sickle cell disease | TITLE |
|  | Das et al., 2008 | Cardiopulmonary stress testing in children with sickle cell disease who are on long-term erythrocytapheresis | TITLE |
|  | Dattaniet al., 2017 | The cause of the stroke: a diagnostic uncertainty | TITLE |
|  | Davidopoulou et al., 2021 | Aggravated Dental and Periodontal Status in Patients with Sickle Cell Disease and Its Association with Serum Ferritin | TITLE |
|  | Davran et al., 2014 | Left renal atrophy | TITLE |
|  | Darbari et al., 2012 | Markers of severe vaso-occlusive painful episode frequency in children and adolescents with sickle cell anemia | TITLE |
|  | Darbari et al., 2013 | Severe Painful Vaso-Occlusive Crises and Mortality in a Contemporary Adult Sickle Cell Anemia Cohort Study | TITLE |
|  | Das et al., 2020 | Use of selexipag in a child with pulmonary hypertension associated with sickle cell disease | TITLE |
|  | Dasgupta et al., 2010 | Antisickling property of fetal hemoglobin enhances nitric oxide bioavailability and ameliorates organ oxidative stress in transgenic-knockout sickle mice | TITLE |
|  | De et al., 2018 | Airway inflammation in sickle cell diseaseA translational perspective | TITLE |
|  | de Almeida et al., 2016 | Sickle Cell Anemia: From Basic Science to Clinical Practice | TITLE |
|  | de Almeida et al., 2021 | Therapist-oriented home rehabilitation for adults with sickle cell anemia: effects on muscle strength, functional capacity, and quality of life | TITLE |
|  | de Araújo et al., 2022 | Exercise Testing In Patients with Sickle Cell Disease: Safety, Feasibility and Potential Prognostic Implication | TITLE |
|  | De Castro et al., 2004 | Pulmonary hypertension in SS, SC and so thalassemia: Prevalence, associated clinical syndromes, and mortality | ABSTRACT |
|  | De et al., 2019 | Airway Inflammation and Lung Function in Sickle Cell Disease | Title |
|  | De et al., 2018 | Airway inflammation in sickle cell disease-A translational perspective | TITLE |
|  | de Boer et al., 2020 | Therapeutic Lessons to be Learned From the Role of Complement Regulators as Double-Edged Sword in Health and Disease | TITLE |
|  | DeBois et al., 2005 | Cardiopulmonary bypass in patients with pre-existing coagulopathy | TITLE |
|  | Denq et al., 2023 | Cardiac Emergency in the Athlete | TITLE |
|  | De et al., 2023 | Acute chest syndrome, airway inflammation and lung function in sickle cell disease | TITLE |
|  | de Araujo Junior et al., 2021 | Cardiovascular benefits of a home-based exercise program in patients with sickle cell disease | TITLE |
|  | Dejong et al., 2022 | Multisystem Inflammatory Syndrome in a Previously Vaccinated Adolescent Female with Sickle Cell Disease | TITLE |
|  | Del Sette et al., 2005 | Transcranial doppler in systemic conditions and stroke | TITLE |
|  | de Gheldere et al., 2006 | Orthopaedic complications associated with sickle-cell disease | TITLE |
|  | Delmonte et al., 2013 | An unusual case of sarcoidosis in an adult patient with sickle cell disease: Management with methotrexate and low dose of steroid | Title |
|  | de Montalembert et al., 2017 | Cardiac iron overload in chronically transfused patients with thalassemia, sickle cell anemia, or myelodysplastic syndrome | TITLE |
|  | de Rouxt al., 2020 | Venoarterial Extracorporeal Membrane Oxygenation in Sickle Cell Disease for Urgent Cardiac Surgery | TITLE |
|  | de Souzaet al., 2007 | Coronary flow reserve in sickle cell anemia | TITLE |
|  | de-Graft Aikins et al., 2010 | Tackling Africa's chronic disease burden: from the local to the global | TITLE |
|  | Deonikar et al., 2014 | Computational analysis of nitric oxide biotransport to red blood cell in the presence of free hemoglobin and NO donor | TITLE |
|  | Desaiet al., 2014 | Mechanistic insights and characterization of sickle cell disease-associated cardiomyopathy | TITLE |
|  | Desai et al., 2012 | A novel molecular signature for elevated tricuspid regurgitation velocity in sickle cell disease | TITLE |
|  | Desai et al., 2020 | Nationwide prevalence and trends in acute cardiovascular events and in-hospital mortality among adult African Americans with sickle cell trait | TITLE |
|  | Deymann et al., 2003 | Myocardial infarction and transient ventricular dysfunction in an adolescent with sickle cell disease | ABSTRACT |
|  | De Castro et al., 2008 | Pulmonary hypertension associated with sickle cell disease: clinical and laboratory endpoints and disease outcomes | ABSTRACT |
|  | De Castro et al., 2005 | Left Sided Heart Dysfunction in Sickle Cell Disease: Echocardiographic and Genetic Studies | TITLE |
|  | De Castro et al., 2006 | Current Prevalence of Specific Clinical Outcomes in Adult Patients with Hb SS or Hb Sβ0 Thalassemia | TITLE |
|  | De Castro et al., 2019 | Anatomy of an exchange transfusion program for adults with sickle cell disease | TITLE |
|  | De Franceschi et al., 2015 | Emerging clinical complication in sickle cell disease | TITLE |
|  | De Franceschi et al., 2008 | Protective effects of phosphodiesterase-4 (PDE-4) inhibition in the early phase of pulmonary arterial hypertension in transgenic sickle cell mice | TITLE |
|  | De Jesus-Rojas et al., 2018 | Screening of pulmonary hypertension in a cohort of children with sickle cell disease | ABSTRACT |
|  | De Laura et al., 2023 | POST-THROMBOEMBOLIC PULMONARY HYPERTENSION: A COMPLEX CASE | TITLE |
|  | de Lima-Filho et al., 2016 | Exercise-Induced Abnormal Increase of Systolic Pulmonary Artery Pressure in Adult Patients With Sickle Cell Anemia: An Exercise Stress Echocardiography Study | TITLE |
|  | deLemos et al., 2013 | Systemic Causes of Cholestasis | TITLE |
|  | DePew et al., 2010 | A 49-year-old woman with acute respiratory failure | TITLE |
|  | De Montalembert et al., 2008 | Management of sickle cell disease | TITLE |
|  | De Montalembert et al., 2004 | Myocardial ischaemia in children with sickle cell disease | TITLE |
|  | Desai et al., 2023 | Ventricular arrhythmias in sickle cell anemia | TITLE |
|  | Desai et al., 2019 | Hypoxia in sickle cell disease due to right to left shunting | TITLE |
|  | de Padua et al., 2012 | Sickle cell anemia: A significant potential cause of pulmonary hypertension in Brazil | ABSTRACT |
|  | de Sousa et al., 2020 | Pulmonary thromboendarterectomy in patients with chronic thromboembolic pulmonary hypertension and hemoglobinopathies | Title |
|  | DeBaun et al., 2014 | The challenge of creating an evidence-based guideline for sickle cell disease | TITLE |
|  | DeBaun et al., 2012 | Associated risk factors for silent cerebral infarcts in sickle cell anemia: low baseline hemoglobin, sex, and relative high systolic blood pressure | TITLE |
|  | Debnath et al., 2021 | Impact of Alloimmunization in Patients with Sickle Cell Disease | TITLE |
|  | Dedeken et al., 2014 | Clinical benefit and costs' evaluation of erythocytapheresis compared to manual exchange transfusion for children with sickle cell disease: A single center experience | TITLE |
|  | Deeb et al., 2022 | HEPATOBILIARY DISEASE AND OUTCOMES FOR PATIENTS LIVING WITH SICKLE CELL ANEMIA: A 30 YEAR REVIEW OF 1009 PATIENTS | TITLE |
|  | Delaney et al., 2013 | LEG ULCERS IN SICKLE CELL DISEASE: CURRENT PATTERNS AND PRACTICES | TITLE |
|  | Delicou et al., 2021 | Acute Chest Syndrome in Sickle Cell Disease: Clinical Presentation and Outcomes. The Experience of a Single Thalassemia and Sickle Cell Unit in a University Hospital | TITLE |
|  | Delicou et al., 2019 | Sickle-Cell Disease in Greece: Patient Reported Outcomes Related to Clinical Complications, Treatment Choices and Attitudes, Beliefs and Trends Affecting Potential Participation in Clinical Trials - a Greek National Multicentric Study | TITLE |
|  | Della-Moretta et al., 2021 | Risk Factors for Adverse Maternal and Fetal Outcomes in Pregnant Patients with Sickle Cell Disease | TITLE |
|  | Dellegrottaglie et al., 2014 | Prevalence and severity of ventricular dysfunction in patients with HIV-related pulmonary arterial hypertension | TITLE |
|  | DeLoughery et al., 2021 | Clinician's Corner: Anemia at Altitude-Thalassemia, Sickle Cell Disease, and Other Inherited Anemias | TITLE |
|  | Delville et al., 2016 | Arterio-venous fistula is an applicable vascular access for erythracytapheresis in patients with sickle cell disease | TITLE |
|  | Delville et al., 2017 | Arterio-venous fistula for automated red blood cells exchange in patients with sickle cell disease: Complications and outcomes | Title |
|  | Delville et al., 2016 | Arterio-Venous Fistula Is an Applicable Vascular Access for Erythracytapheresis in Patients with Sickle Cell Disease | TITLE |
|  | Dembinski et al., 2006 | Pulmonary hypertension | TITLE |
|  | Deneuve et al., 2017 | Patient with homozygous sickle cell disease and free flap surgery: Ensuring the success of the procedure | TITLE |
|  | Deneuve et al., 2017 | Drépanocytose homozygote et nécessité d’une réparation par lambeau libre : comment assurer la réussite du projet thérapeutique | TITLE |
|  | Deonikar et al., 2012 | Low micromolar intravascular cell-free hemoglobin concentration affects vascular NO bioavailability in sickle cell disease: A computational analysis | TITLE |
|  | Derchi et al., 2014 | Efficacy and safety of sildenafil for the treatment of severe pulmonary hypertension in patients with hemoglobinopathies: results from a long-term follow up | ABSTRACT |
|  | Derchi et al., 2005 | Incidence of Pulmonary Hypertension in Haemoglobinopathic Patients without Left Ventricular Disfunction | TITLE |
|  | Derchi et al., 2005 | Therapeutic approaches to pulmonary hypertension in hemoglobinopathies: Efficacy and safety of sildenafil in the treatment of severe pulmonary hypertension in patients with hemoglobinopathy | Title |
|  | Derchi et al., 2005 | Therapeutic approaches to pulmonary hypertension in hemoglobinopathies - Efficacy and safety of sildenafil in the treatment of severe pulmonary hypertension in patients with hemoglobinopathy | TITLE |
|  | Derchi et al., 2005 | Efficacy and safety of sildenafil in the treatment of severe pulmonary hypertension in patients with hemoglobinopathies | TITLE |
|  | Derchi et al., 2012 | Estimation of the prevalence of pulmonary artery hypertension in a large group of β-thalassemia patients using right heart catheterization | ABSTRACT |
|  | Desai et al., 2017 | Association of circulating transcriptomic profiles with mortality in sickle cell disease | TITLE |
|  | Desai et al., 2022 | The Cardiopulmonary Complications of Sickle Cell Disease | TITLE |
|  | Desai et al., 2012 | A novel molecular signature for elevated tricuspid regurgitation velocity in sickle cell disease | ABSTRACT |
|  | Desai et al., 2010 | Systemic blood pressure is associated with anemia and placenta growth factor in sickle cell anemia | Title |
|  | Desai et al., 2011 | Systemic blood pressure is associated with anemia and placenta growth factor in sickle cell anemia | TITLE |
|  | Desai et al., 2012 | Decades after the cooperative study: A re-examination of systemic blood pressure in sickle cell disease | TITLE |
|  | Desai et al., 2015 | Alloimmunization is associated with older age of transfused red blood cells in sickle cell disease | TITLE |
|  | Desai et al., 2011 | Longitudinal study of echocardiographically-derived tricuspid regurgitant jet velocity in sickle cell disease | ABSTRACT |
|  | Desai et al., 2013 | Longitudinal study of echocardiography-derived tricuspid regurgitant jet velocity in sickle cell disease | ABSTRACT |
|  | DesJardin et al., 2019 | Successful Use of Pulmonary Vasodilators in Acute Chest Syndrome Complicated by Persistent Right Ventricular Failure | TITLE |
|  | Dessap et al., 2011 | Pulmonary artery thrombosis during acute chest syndrome in sickle cell disease | TITLE |
|  | Dessap et al., 2008 | Pulmonary hypertension and cor pulmonale during severe acute chest syndrome in sickle cell disease | TITLE |
|  | Detterich et al., 2010 | Elevated tricuspid regurgitation jet correlates with decreased brachial artery relaxivity in sickle cell anemia patients on chronic transfusion therapy | ABSTRACT |
|  | Detterich et al., 2011 | Acute cardiovascular and hematologic changes after a single transfusion demonstrate sex differences in chronically transfused sickle cell anemia patients | TITLE |
|  | Detterich et al., 2018 | Simple chronic transfusion therapy, a crucial therapeutic option for sickle cell disease, improves but does not normalize blood rheology: What should be our goals for transfusion therapy? | TITLE |
|  | Detterich et al., 2015 | Chronic transfusion therapy improves but does not normalize systemic and pulmonary vasculopathy in sickle cell disease | ABSTRACT |
|  | Detterich et al., 2012 | Male patients with sickle cell anemia on simple chronic transfusion protocol show increased hemolysis and reticulocyte production as well as increased systemic and pulmonary resistance compared to female patients | Title |
|  | Detterich et al., 2013 | Patients with sickle cell anemia on simple chronic transfusion protocol show sex differences for hemodynamic and hematologic responses to transfusion | ABSTRACT |
|  | Detterich et al., 2009 | Pulmonary hypertension and vascular disease in patients with sickle cell anemia on chronic transfusion therapy | ABSTRACT |
|  | Deveci et al., 2022 | Increased Circulating Copeptin Levels Are Associated with Vaso-Occlusive Crisis and Right Ventricular Dysfunction in Sickle Cell Anemia | ABSTRACT |
|  | Dhabangi et al., 2017 | B-type natriuretic peptide and plasma hemoglobin levels following transfusion of shorter-storage versus longer-storage red blood cells: Results from the TOTAL randomized trial | TITLE |
|  | Dham et al., 2009 | Prospective echocardiography assessment of pulmonary hypertension and its potential etiologies in children with sickle cell disease | ABSTRACT |
|  | Dhar et al., 2021 | Longitudinal analysis of cardiac abnormalities in pediatric patients with sickle cell anemia and effect of hydroxyurea therapy | TITLE |
|  | Dhayhi et al., 2021 | Rare presentation of infective endocarditis due to Salmonella entrica subspecies salamae (subgroup ll) in a sickle cell anemia girl | ABSTRACT |
|  | Dhédin et al., 2020 | Allogeneic hematopoietic stem cell transplant in children and adults with sickle cell disease: Indications and modalities | TITLE |
|  | d'Humières et al., 2023 | Determinants of ventricular arrhythmias in sickle cell anemia: toward better prevention of sudden cardiac death | TITLE |
|  | Di Liberto et al., 2016 | Dense red blood cell and oxygen desaturation in sickle-cell disease | TITLE |
|  | Di Maggio et al., 2019 | Sickle related events following cardiac catheterisation: risk implication for other invasive procedures | TITLE |
|  | Di Maggio et al., 2014 | Chronic administration of hydroxyurea (HU) and outcomes in patients with sickle cell disease (SCD) at a single referral institution | TITLE |
|  | Di Matteo et al., 2012 | Recent developments in transposon-mediated gene therapy | TITLE |
|  | Diamantidis et al., 2023 | Clinical significance of mutational variants in beta and alpha genes in patients with hemoglobinopathies from two large Greek centers: a complex interplay between genotype and phenotype | TITLE |
|  | Di Maggio et al., 2018 | Chronic administration of hydroxyurea (HU) benefits caucasian patients with sickle-beta thalassemia | TITLE |
|  | Di Maria et al., 2015 | Left ventricular rotational mechanics in Tanzanian children with sickle cell disease | TITLE |
|  | Dimova et al., 2019 | Diastolic dysfunction and cardiomyopathy in patients with sickle cell anemia | TITLE |
|  | Dickerhoff et al., 2015 | Hemoglobin disorders | TITLE |
|  | Dickerson et al., 2012 | Young adults with SCD in US children's hospitals: Are they different from adolescents? | TITLE |
|  | Dickert et al., 2009 | Ancillary care obligations of medical researchers | TITLE |
|  | Didichenko et al., 2022 | CSL112 Increases Plasma Cholesterol Efflux Capacity and Reduces Erythrocyte Membrane Cholesterol Content in Blood Samples from Patients with Sickle Cell Disease | TITLE |
|  | Dierick et al., 2018 | The burden of sickle cell disease | TITLE |
|  | Dimopoulou et al., 2022 | COVID-19 in patients with thalassemia and sickle cell disease: A single center experience | TITLE |
|  | Dimova et al., 2019 | Incidence, diagnosis and treatment of pulmonary hypertension in patients with sickle cell anemia | TITLE |
|  | Dinan et al., 2013 | The current state of imaging pediatric hemoglobinopathies | TITLE |
|  | Ding et al., 2009 | Limbal stem cell deficiency arising from systemic chemotherapy with hydroxycarbamide | TITLE |
|  | Dingle et al., 2023 | Healthcare Utilization and Cost of Care Analysis of Patients with Sickle Cell Disease with High Vs. Low Biomarkers: A Retrospective Chart Review | TITLE |
|  | Diagneet al., 2000 | Infections in Senegalese children and adolescents with sickle cell anemia: epidemiological aspects | TITLE |
|  | Dobie et al., 2023 | Sickle Cell Disease and Thromboembolism: New Insights on the Pathophysiology, Diagnosis, and Treatment | TITLE |
|  | Doiron et al., 2022 | Pregnancy-Related Stroke: A Review | TITLE |
|  | Do et al., 2012 | Successful management of plastic bronchitis in a child post fontan: Case report and literature review | TITLE |
|  | Dobre et al., 2022 | Acidosis in renal disease: should we be concerned? | TITLE |
|  | Dowling et al., 2011 | Intracardiac shunting and stroke in children: A systematic review | TITLE |
|  | Domanski et al., 2023 | The criminalisation of women’s healthcare in the post-Dobbs era: an analysis of the anti-abortion trigger law statutes | TITLE |
|  | Dong et al., 2023 | EE310 Relationships Among Sickle Cell Disease Complications and Their Implications for Cost-Effectiveness Modeling for Therapies with Curative Intent | TITLE |
|  | Dooms et al., 2013 | Do we need authorized orphan drugs when compounded medications are available? | TITLE |
|  | D'Orléans-Juste et al., 2008 | Forefronts in endothelin | TITLE |
|  | Doss et al., 2016 | Phase 1 Study of a Sulforaphane-Containing Broccoli Sprout Homogenate for Sickle Cell Disease | TITLE |
|  | Diaw et al., 2015 | Blood viscosity is lower in trained than in sedentary sickle cell trait carriers | TITLE |
|  | Diaw et al., 2015 | Sickle Cell Trait Worsens Oxidative Stress, Abnormal Blood Rheology, and Vascular Dysfunction in Type 2 Diabetes | TITLE |
|  | Díaz-Pache et al., 2011 | Treatment of a female patient with sickle-cell anemia during cardiac surgery with cardiopulmonary bypass | TITLE |
|  | Doshi et al., 2013 | Increased left main coronary artery dimensions in children with sickle cell disease | TITLE |
|  | Douce et al., 2019 | Association of sickle cell trait with atrial fibrillation: The REGARDS cohort | TITLE |
|  | Doukkyt al., 2012 | A novel expression of exercise induced pulmonary hypertension in human immunodeficiency virus patients: a pilot study | TITLE |
|  | Dowling et al., 2010 | Prevalence of intracardiac shunting in children with sickle cell disease and stroke | TITLE |
|  | Dowling et al., 2017 | Increased prevalence of potential right-to-left shunting in children with sickle cell anaemia and stroke | Title |
|  | Dowling et al., 2009 | Stroke in Sickle Cell Anemia: Alternative Etiologies | TITLE |
|  | Drossner et al., 2011 | Cardiac disease in pediatric patients presenting to a pediatric ED with chest pain | TITLE |
|  | Du et al., 2022 | Case Report: Report of Infective Endocarditis Caused by Abiotrophia defectiva and Literature Review | TITLE |
|  | Duarte et al., 2016 | Genome-Wide Analysis Identifies IL-18 and FUCA2 as Novel Genes Associated with Diastolic Function in African Americans with Sickle Cell Disease | TITLE |
|  | Duckworth et al., 2020 | Spirometry use in Patients with Sickle Cell Disease with and without asthma and acute chest syndrome: A Multicenter Study | TITLE |
|  | Durgin et al., 2022 | Smooth muscle cell CYB5R3 preserves cardiac and vascular function under chronic hypoxic stress | TITLE |
|  | Dosunmu-Ogunbi et al., 2022 | SOD2 V16A amplifies vascular dysfunction in sickle cell patients by curtailing mitochondria complex IV activity | TITLE |
|  | Dosunmu-Ogunbi et al., 2022 | Endothelial superoxide dismutase 2 is decreased in sickle cell disease and regulates fibronectin processing | TITLE |
|  | Dosunmu-Ogunbi et al., 2019 | Decoding the role of SOD2 in sickle cell disease | TITLE |
|  | Doyle et al., 2021 | Paraganglioma in an adolescent awaiting a cardiac transplant due to unrepaired congenital cyanotic heart disease and a univentricular heart: The first reported case | TITLE |
|  | Duarte-Neto et al., 2021 | An autopsy study of the spectrum of severe COVID-19 in children: From SARS to different phenotypes of MIS-C | TITLE |
|  | Duke et al., 2023 | Randomised controlled trials in child and adolescent health in 2023 | TITLE |
|  | Dutta et al., 2019 | Effects of rifaximin on circulating aged neutrophils in sickle cell disease | TITLE |
|  | Drasar et al., 2014 | Sickle cell disease in an urban environment in the United Kingdom: Description of an adult cohort | TITLE |
|  | Drawz et al., 2016 | Kidney disease among patients with sickle cell disease, hemoglobin SS and SC | TITLE |
|  | Driscoll, M. C.  et al., 2007 | Sickle cell disease | TITLE |
|  | Driss et al., 2011 | Regular automated erythrocytapheresis in sickle cell patients | TITLE |
|  | Dubert et al., 2017 | Degree of anemia, indirect markers of hemolysis, and vascular complications of sickle cell disease in Africa | ABSTRACT |
|  | Dudek et al., 2010 | A single nucleotide polymorphism in the human cortactin gene associates with sickle cell disease and alters protein function | TITLE |
|  | Durante et al., 2019 | The emerging role of l-glutamine in cardiovascular health and disease | TITLE |
|  | Duru et al., 2021 | Variations and characteristics of the various clinical phenotypes in a cohort of Nigerian sickle cell patients | ABSTRACT |
|  | Dworkis et al., 2009 | Intravascular hemolysis in sickle cell disease: Discovery of associated genomic copy number variations | TITLE |
|  | Dwyer et al., 2011 | Bosentan for the treatment of adult pulmonary hypertension | TITLE |
|  | Dzik et al., 2008 | Nitric Oxide: An Introductory Primer | TITLE |
|  | Dzudie et al., 2020 | Pulmonary hypertension in low- And middle-income countries with focus on sub-Saharan Africa | ABSTRACT |
|  | Eapen et al., 2019 | A phase II trial to compare allogeneic transplant vs. standard of care for severe sickle cell disease: Blood and marrow transplant clinical trials network (BMT CTN) protocol 1503 | TITLE |
|  | Ebert et al., 2016 | The NHLBI SMARTT Program: A Novel Approach to Facilitate Translational Research for Heart, Lung and Blood Diseases | TITLE |
|  | Echaniz-Aviles et al., 2012 | Epidemiology of pneumococcal diseases in Mexico in patients older than 50 years old | TITLE |
|  | Echeverría et al., 2008 | Patología respiratoria en pacientes con enfermedad de células falciformes. Protocolo de actuación (I) | TITLE |
|  | Eckman et al., 2001 | Techniques for blood administration in sickle cell patients | TITLE |
|  | Eckmanet al., 2017 | Standard measures for sickle cell disease research: the PhenX Toolkit sickle cell disease collections | TITLE |
|  | Eckman et al., 2011 | Sickle cell anemia pathophysiology: Back to the data | TITLE |
|  | Edwards, et al., 2017 | Repeated critical illness and unplanned readmissions within 1 year to PICUs | TITLE |
|  | Edwin et al., 2010 | eComment: Cardiopulmonary bypass without exchange transfusion in sickle cell disease - An update | Title |
|  | Edwin et al., 2011 | The development of cardiac surgery in West Africa - The case of Ghana | TITLE |
|  | Egrie et al., 2007 | A Child With Sickle Cell Disease and Anomalous Right Coronary Artery | TITLE |
|  | El Missiry et al., 2011 | Impaired arginine-nitric oxide oathway and oulmonary hypertension in thalassemia and sickle cell hemoglobinopathies-(Review article) | TITLE |
|  | Elalfy et al., 2018 | Left Ventricular Structural and Functional Changes in Children With β-Thalassemia and Sickle Cell Disease: Relationship to Sleep-disordered Breathing | TITLE |
|  | Elbahlawan et al., 2021 | Pulmonary Manifestations of Hematologic and Oncologic Diseases in Children | TITLE |
|  | Edwin et al., 2010 | eComment: Cardiopulmonary bypass without exchange transfusion in sickle cell disease--an update | TITLE |
|  | Edwinet al., 2014 | Hypothermic cardiopulmonary bypass without exchange transfusion in sickle-cell patients: a matched-pair analysis | TITLE |
|  | Elenga et al., 2014 | Associated factors of acute chest syndrome in children with sickle cell disease in French Guiana | ABSTRACT |
|  | El-Maghraby et al., 2004 | Clinical relevance of left ventricular volumes and function assessed by gated SPECT in paediatric patients | Title |
|  | El-Beshlawy et al., 2006 | Diastolic dysfunction and pulmonary hypertension in sickle cell anemia: is there a role for L-carnitine treatment? | ABSTRACT |
|  | El-Beshlawy et al., 2008 | Hydroxyurea in the Management of Pediatric Sickle Cell Patients | TITLE |
|  | El-Din Farghal et al., 2022 | SLEEP DISORDERED BREATHING IN SICKLE CELL DISEASE: RELATION TO PULMONARY HYPERTENSION AND STROKE | Title |
|  | Eid et al., 2021 | Infective endocarditis with embolic renal infarct presenting as acute abdomen | TITLE |
|  | Ejim et al., 2016 | Left ventricular systolic function in sickle cell anaemia: An echocardiographic evaluation in adult Nigerian patients | ABSTRACT |
|  | Elenga et al., 2022 | Real-World data on efficacy of L-glutamine in preventing sickle cell disease-related complications in pediatric and adult patients | TITLE |
|  | Eliyahu et al., 2023 | Effects of Hormone Replacement Therapy on Women's Lung Health and Disease | TITLE |
|  | Ellithy et al., 2015 | Relation between glutathione s transferase genes (GSTM1, GSTT1 and GSTP1) polymorphisms and clinical manifestations of sickle cell disease in Egyptian patients | TITLE |
|  | Elmariah et al., 2012 | Clinical characteristics associated with survival in adult sickle cell disease | ABSTRACT |
|  | Elia et al., 2021 | Acute chest syndrome and COVID-19 in sickle cell disease pediatric patients | TITLE |
|  | Enriquez-Sarano et al., 2022 | Transcontinental Cooperation of Cardiologists: Caring for Difficult Valvular Cases Supported by “La-Chaine-de-l'espoir” and “United-Surgeons-For-Children” | TITLE |
|  | Español et al., 2022 | Multisystem Inflammatory Syndrome in a Pediatric Patient with Sickle Cell Disease and COVID-19: A Case Report | TITLE |
|  | El Missiri et al., 2014 | Echocardiographic assessment of left ventricular mechanical dyssynchrony - A practical approach | TITLE |
|  | Elazhary et al., 2022 | Bradycardia associated with antithymocyte globulin treatment of a pediatric patient with sickle cell disease: a case report and literature review | TITLE |
|  | Elbers et al., 2015 | The pediatric stroke code: Early management of the child with stroke | TITLE |
|  | El-Maghrabyt al., 2002 | Value of myocardial SPECT in early detection of ischaemia in children with sickle cell anaemia | TITLE |
|  | Elmariah et al., 2014 | Factors associated with survival in a contemporary adult sickle cell disease cohort | Abstract |
|  | Emokpae et al., 2012 | Association of oxidative stress markers with atherogenic index of plasma in adult sickle cell nephropathy | Title |
|  | Elsharawy et al., 2009 | Atherosclerosis in sickle cell disease - A review | TITLE |
|  | Elshinawy et al., 2016 | Adenotonsillectomy in sickle cell disease: Is transfusion needed? | TITLE |
|  | El-Beshlawy et al., 2019 | International sentinel site surveillance of patients with transfusional hemosiderosis treated with deferasirox in actual practice setting | TITLE |
|  | El-Hawy et al., 2022 | Assessment of serum neopterin and kynurenine levels in Egyptian children with sickle cell disease: a single center study | TITLE |
|  | Emeline et al., 2021 | Cardiac rhabdomyoma with hydrops fetalis: Prenatal management by abdominal drainage | TITLE |
|  | Enakpene et al., 2014 | Non-invasive estimation of pulmonary artery pressures in patients with sickle cell anaemia in Ibadan, Nigeria: An echocardiographic study | ABSTRACT |
|  | Engmannet al., 2021 | The contribution of family physicians to chronic disease management through continuity of care in Ghana | TITLE |
|  | Enayatollahi et al., 2015 | In-Hospital Morbidity and Mortality Following Total Joint Arthroplasty in Patients with Hemoglobinopathies | Title |
|  | Ephraim et al., 2016 | Normal Non-HDL Cholesterol, Low Total Cholesterol, and HDL Cholesterol Levels in Sickle Cell Disease Patients in the Steady State: A Case-Control Study of Tema Metropolis | TITLE |
|  | Epis et al., 2022 | Simple open-heart surgery protocol for sickle-cell disease patients: a retrospective cohort study comparing patients undergoing mitral valve surgery | TITLE |
|  | Epstein et al., 2022 | Considerations for the future: current and future treatment paradigms with mineralocorticoid receptor antagonists-unmet needs and underserved patient cohorts | TITLE |
|  | Ershler et al., 2023 | Hemoglobin and End-Organ Damage in Individuals with Sickle Cell Disease | TITLE |
|  | Erdbruegger et al., 2010 | Circulating endothelial cells: Markers and mediators of vascular damage | Title |
|  | Espinosa Martínez et al., 2010 | Pulmonary hypertension in adults with sickle cell anemia. Preliminary results | ABSTRACT |
|  | Esse et al., 2019 | The contribution of homocysteine metabolism disruption to endothelial dysfunction: State-of-the-art | TITLE |
|  | Estcourt et al., 2016 | Preoperative blood transfusions for sickle cell disease | Title |
|  | Estcourt et al., 2016 | Regular long-term red blood cell transfusions for managing chronic chest complications in sickle cell disease | Title |
|  | Estrada Del Cueto et al., 2010 | Analysis of some hematological, biochemical and clinical variables in cases of sickle cell anemia. Preliminary results | TITLE |
|  | Etienne-Julan et al., 2018 | Characteristics of episodes of acute chest syndrome in adults patients with sickle cell disease in guadeloupe and predictive factors of severity: A retrospective study | ABSTRACT |
|  | Everett et al., 2014 | Paediatrics and proteomics: Back to the beginning | TITLE |
|  | Falamaki et al., 2016 | The emergent body imaging findings of sickle cell anemia: What every emergency room radiologist needs to know | TITLE |
|  | Falkner et al., 2020 | Blood pressure unknowns of sickle cell disease in children | TITLE |
|  | Farber et al., 2004 | Mechanisms of disease: Pulmonary arterial hypertension | TITLE |
|  | Faria et al., 2013 | Pulmonary capillary hemangiomatosis: An uncommon cause of pulmonary hypertension | TITLE |
|  | Farmakis et al., 2011 | Pulmonary hypertension associated with hemoglobinopathies: Prevalent but overlooked | TITLE |
|  | Farmakis et al., 2012 | Letter by Farmakis and aessopos regarding article, "echocardiographic Markers of elevated pulmonary pressure and left ventricular diastolic dysfunction are associated with exercise intolerance in adults and adolescents with homozygous sickle cell anemia in the United States and United Kingdom" | TITLE |
|  | Fares et al., 2011 | Pulmonary hypertension: Clinical presentation, diagnosis, treatment, and dana point world symposium highlights | TITLE |
|  | Farawela et al., 2016 | Association between Duffy antigen receptor expression and disease severity in sickle cell disease patients | TITLE |
|  | Farmakis et al., 2017 | Heart failure in haemoglobinopathies: pathophysiology, clinical phenotypes, and management | TITLE |
|  | Farooqui et al., 2016 | CASE 10—2016: Hemoglobinopathies and Cardiac Surgery | TITLE |
|  | Farooqui et al., 2023 | VENOUS THROMBOEMBOLISM INCIDENCE IN FEMALE SICKLE CELL DISEASE PATIENTS ON HYDROXYUREA | TITLE |
|  | Faro et al., 2015 | Left ventricular hypertrophy in children, adolescents and young adults with sickle cell anemia | Title |
|  | Faulkner et al., 2014 | Hematopietic stem cell transplantation for thalassemia, price and prejudice | Title |
|  | Farzaeiet al., 2023 | Regulatory effects of trimetazidine in cardiac ischemia/reperfusion injury | TITLE |
|  | Fattori et al., 2015 | Sickle-cell Anemia and Latent Diastolic Dysfunction: Echocardiographic Alterations | TITLE |
|  | Fawibe et al., 2012 | Advanced sickle cell associated interstitial lung disease presenting with cor pulmonale in a Nigerian | TITLE |
|  | Farrell et al., 2019 | End points for sickle cell disease clinical trials: Renal and cardiopulmonary, cure, and low-resource settings | TITLE |
|  | Fartoukh et al., 2009 | Resuscitation | TITLE |
|  | Federici et al., 2011 | Atypical thrombosis in hematology | TITLE |
|  | Feitet al., 2023 | Long-term clinical outcomes and healthcare utilization of sickle cell disease patients with COVID-19: A 2.5-year follow-up study | TITLE |
|  | Federti et al., 2023 | In Humanized Sickle Cell Mice, Imatinib Protects Against Sickle Cell-Related Injury | TITLE |
|  | Feray et al., 2017 | Plastic bronchitis: An unusual complication of acute chest syndrome in adult | TITLE |
|  | Ferdinande et al., 2023 | A remarkable presentation of a massive Budd-Chiari syndrome | TITLE |
|  | Feld et al., 2015 | Liver injury is associated with mortality in sickle cell disease | TITLE |
|  | Fergusonet al., 2019 | Effects of living at moderate altitude on pulmonary vascular function and exercise capacity in mice with sickle cell anaemia | TITLE |
|  | Ferguson et al., 2020 | The effect of dietary nitrate supplementation on the speed-duration relationship in mice with sickle cell disease | TITLE |
|  | Ferriero et al., 2019 | Management of Stroke in Neonates and Children: A Scientific Statement From the American Heart Association/American Stroke Association | TITLE |
|  | Field et al., 2008 | Growth of lung function in children with sickle cell anemia | TITLE |
|  | Field et al., 2008 | Longitudinal analysis of pulmonary function in adults with sickle cell disease | TITLE |
|  | Field et al., 2012 | The role of fibrocytes in sickle cell lung disease | Title |
|  | Field et al., 2020 | Phase 2 trial of montelukast for prevention of pain in sickle cell disease | Title |
|  | Feldet al., 2021 | NT-proBNP levels and cardiopulmonary function in children with sickle cell disease | ABSTRACT |
|  | Fitzhugh et al., 2022 | Knowledge to date on secondary malignancy following hematopoietic cell transplantation for sickle cell disease | TITLE |
|  | Fucharoen et al., 2012 | The hemoglobin E thalassemias | TITLE |
|  | Fermaglich et al., 2023 | A comprehensive study of the rare diseases and conditions targeted by orphan drug designations and approvals over the forty years of the Orphan Drug Act | TITLE |
|  | Fernández Águila et al., 2022 | Chronic organ dysfunction in patients with sickle cell anemia. Part I: cardiorespiratory manifestations | TITLE |
|  | Ferreira De Melo et al., 2014 | What are the most promising emerging therapies for sickle cell disease? | TITLE |
|  | Fertrin et al., 2012 | Monocyte shift to a non-classical cd14dim/cd16+ phenotype correlates with fetal hemoglobin levels in sickle cell anemia patients treated with hydroxyurea | TITLE |
|  | Fertrin et al., 2010 | Genomic polymorphisms in sickle cell disease: implications for clinical diversity and treatment | TITLE |
|  | Field et al., 2008 | Enuresis is a common and persistent problem among children and young adults with sickle cell anemia | TITLE |
|  | Field et al., 2010 | Author response letter to: "Pulmonary thrombi are not detected by 3D magnetic resonance angiography in adults with sickle cell anemia and an elevated tricuspid regurgitant jet velocity" | TITLE |
|  | Field et al., 2014 | Advances in sickle cell therapies in the hydroxyurea era | TITLE |
|  | Fieldet al., 2011 | Airway hyperresponsiveness in children with sickle cell anemia | TITLE |
|  | Fields et al., 2020 | Chronic Disease Management in Sickle Cell Trait Patients in the Primary Care Setting: A Case Report | TITLE |
|  | Filho et al., 2015 | Raman spectroscopy for a rapid diagnosis of sickle cell disease in human blood samples: a preliminary study | TITLE |
|  | Fitzgerald et al., 2012 | Misclassification of pulmonary hypertension in adults with sickle hemoglobinopathies using Doppler echocardiography | ABSTRACT |
|  | Fitzhugh et al., 2012 | Infusion of hemolyzed red blood cells within peripheral blood stem cell grafts in patients with and without sickle cell disease | Title |
|  | Field et al., 2011 | Targeting iNKT cells for the treatment of sickle cell disease | TITLE |
|  | Finkelhor et al., 2015 | Limitations and strengths of doppler/echo pulmonary artery systolic pressure-right heart catheterization correlations: A systematic literature review | TITLE |
|  | Fiore et al., 2010 | Altitude illness: Risk factors, prevention, presentation, and treatment | TITLE |
|  | Fioretto et al., 2003 | Inhaled nitric oxide in pediatrics | TITLE |
|  | Fitzhugh et al., 2016 | Post-transplant cyclophosphamide improves engraftment in patients with sickle cell disease (SCD) and severe organ damage who undergo haploidentical peripheral blood stem cell transplantation (PBSCT) | TITLE |
|  | Fitzhugh et al., 2005 | Morbidity and Associated Sudden Death in Sickle Cell Disease | TITLE |
|  | Fitzhugh et al., 2015 | Hydroxyurea-Increased Fetal Hemoglobin Is Associated with Less Organ Damage and Longer Survival in Adults with Sickle Cell Anemia | TITLE |
|  | Fitzhugh et al., 2010 | Cardiopulmonary complications leading to premature deaths in adult patients with sickle cell disease | ABSTRACT |
|  | Fitzhugh et al., 2022 | Long-Term Health Effects of Curative Therapies on Heart, Lungs, and Kidneys for Individuals with Sickle Cell Disease Compared to Those with Hematologic Malignancies | TITLE |
|  | Fitzhugh et al., 2017 | The case for HLA-identical sibling hematopoietic stem cell transplantation in children with symptomatic sickle cell anemia | TITLE |
|  | Föll et al., 2020 | A phase ii stratified trial to assess haploidentical tdepleted stem cell transplantation in patients with sickle cell disease with no available sibling donor | TITLE |
|  | Fonseca et al., 2015 | Pulmonary hypertension in sickle cell disease | ABSTRACT |
|  | Fonseca et al., 2010 | Diagnosis of pulmonary hypertension in adults with sickle cell disease | ABSTRACT |
|  | Ford et al., 2022 | Secondary Prevention of Ischemic Stroke: Updated Guidelines From AHA/ASA | TITLE |
|  | Francisco et al., 2023 | Recovering from a renal vascular catastrophe: Case report | TITLE |
|  | Forni et al., 2008 | Manual Erythro-Exchange (MEEX) to Prevent Complications of Sickle Cell Disease in Patients Unresponsive to Hydroxyurea: A Long-Term Follow-up | TITLE |
|  | Forrest et al., 2011 | Pulmonary hypertension and proteinuria in children with sickle cell disease | ABSTRACT |
|  | Fonseca et al., 2021 | Cerebrovascular Complications of Anemia | TITLE |
|  | Fontaine et al., 2008 | Clinical assessment of the risk for sudden cardiac death in patients with sickle cell anemia | TITLE |
|  | Fox et al., 2012 | Raised erythrocyte creatine in patients with pulmonary arterial hypertension--evidence for subclinical hemolysis | TITLE |
|  | Friedmanet al., 2021 | Stable to improved cardiac and pulmonary function in children with high-risk sickle cell disease following haploidentical stem cell transplantation | TITLE |
|  | Fort et al., 2019 | Recommendations for the use of red blood cell exchange in sickle cell disease | TITLE |
|  | Foster et al., 2009 | Protein S-nitrosylation in health and disease: a current perspective | TITLE |
|  | Foster et al., 2003 | S-nitrosylation in health and disease | TITLE |
|  | Foxet al., 2016 | Endothelin receptor antagonists in sickle cell disease: A promising new therapeutic approach | TITLE |
|  | Fujikura et al., 2022 | Myocardial Iron Overload Causes Subclinical Myocardial Dysfunction in Sickle Cell Disease | TITLE |
|  | Fujikura et al., 2018 | Increased Iron Deposition Is Directly Associated With Myocardial Dysfunction in Patients With Sickle Cell Disease | TITLE |
|  | Fung et al., 2008 | Disparity in the management of iron overload between patients with sickle cell disease and thalassemia who received transfusions | TITLE |
|  | Frantz et al., 2012 | Diagnostic Dilemmas in Pulmonary Hypertension | Title |
|  | Freed et al., 2012 | Allogeneic cellular and autologous stem cell therapy for sickle cell disease: Whom, when and how | TITLE |
|  | Freeman et al., 2015 | Pulmonary endarterectomy as treatment for chronic thromboembolic pulmonary hypertension in sickle cell disease | TITLE |
|  | Friebeet al., 2022 | NO-sensitive guanylyl cyclase in the lung | TITLE |
|  | Friedman et al., 2016 | Hospital delivery volume, severe obstetrical morbidity, and failure to rescue | TITLE |
|  | Furlow et al., 2014 | Pulmonary hypertension of sickle cell disease: new guidelines | ABSTRACT |
|  | Gaartman et al., 2021 | Fluid overload due to intravenous fluid therapy for vaso-occlusive crisis in sickle cell disease: incidence and risk factors | TITLE |
|  | Gaber et al., 2014 | Prevalence of pulmonary hypertension in patients attending echocardiolgy clinic in the eastern part of libya | ABSTRACT |
|  | Gacon et al., 2001 | Cardiac disorders in sickle-cell anemia | TITLE |
|  | Garde et al., 2022 | Recent advances in paediatric sleep disordered breathing | TITLE |
|  | Gardner et al., 2022 | Pregnancy in Patients with Shone Complex: A Single-Center Case Series | TITLE |
|  | Garg et al., 2005 | Fat embolism | TITLE |
|  | Galactéros et al., 2023 | Le globule rouge drépanocytaire. Données génétiques et structurelles | TITLE |
|  | Gati et al., 2013 | Increased left ventricular trabeculation in individuals with sickle cell anaemia: Physiology or pathology? | TITLE |
|  | Gayanilo et al., 2014 | Acute myocardial ischemia in association with acute respiratory acidosis in hemoglobin SC disease: A case report | TITLE |
|  | Galactéros et al., 2023 | The sickle cell. Genetic and structural data | TITLE |
|  | Galacteros et al., 2023 | Modeling the public health impact of voxelotor in the management of sickle cell disease in France | TITLE |
|  | Galadanci et al., 2021 | Prevalence and Factors Associated with Echocardiographic Abnormalities in Children with Sickle Cell Disease; Results from the Displace Study | ABSTRACT |
|  | Gallagher et al., 2015 | Transporting down the road to dehydration | TITLE |
|  | Garg et al., 2021 | Long and short interpregnancy intervals increase severe maternal morbidity | TITLE |
|  | Garrido et al., 2012 | Elevated plasma levels and platelet-associated expression of the pro-thrombotic and pro-inflammatory protein, TNFSF14 (LIGHT), in sickle cell disease | ABSTRACT |
|  | Gacon et al., 2001 | Cardiac manifestations of sickle cell anemia | TITLE |
|  | Galadancit al., 2021 | Association Between Patent Foramen Ovale and Overt Ischemic Stroke in Children With Sickle Cell Disease | TITLE |
|  | Galadanci et al., 2022 | Factors associated with left ventricular hypertrophy in children with sickle cell disease: results from the DISPLACE study | TITLE |
|  | Garadah et al., 2016 | Hormonal and echocardiographic abnormalities in adult patients with sickle-cell anemia in Bahrain | TITLE |
|  | Gauthier et al., 2020 | Goal-Oriented Monitoring of Cyclosporine Is Effective for Graft-versus-Host Disease Prevention after Hematopoietic Stem Cell Transplantation in Sickle Cell Disease and Thalassemia Major | TITLE |
|  | Gassner et al., 2021 | GBT440 Increases Hematocrit and Improves Biventricular Function in Berkeley Sickle Cell Disease Mice | TITLE |
|  | Gazza et al., 2023 | Correlation between Disease Biomarkers and Hemoglobin F Levels in Sickle Cell Patients | TITLE |
|  | Gbotoshoet al., 2017 | Free heme induces a heme importer, heme catabolism and an angiogenic factor in multiple organs in mice: Implications for organ-specific response to intravascular hemolysis | TITLE |
|  | Gbotosho et al., 2023 | The Role of Inflammation in The Cellular and Molecular Mechanisms of Cardiopulmonary Complications of Sickle Cell Disease | TITLE |
|  | Gbotosho et al., 2020 | Heme Induces IL-6 and Cardiac Hypertrophy Genes Transcripts in Sickle Cell Mice | TITLE |
|  | Gbotosho et al., 2021 | The Worst Things in Life are Free: The Role of Free Heme in Sickle Cell Disease | TITLE |
|  | Gbotosho et al., 2021 | Cardiac pathophysiology in sickle cell disease | Title |
|  | Geard et al., 2016 | Genetics of sickle cell-associated cardiovascular disease: An expert review with lessons learned in Africa | TITLE |
|  | Ge et al., 2021 | Application of the RCOG risk assessment model for evaluating postpartum venous thromboembolism in chinese women: A case-control study | TITLE |
|  | Geevasinga et al., 2014 | Sickle cell disease and posterior reversible leukoencephalopathy | TITLE |
|  | Gendreau et al., 2020 | Cerebral fat embolism in sickle cell disease | TITLE |
|  | Georgeet al., 2022 | Whole blood versus red cell concentrates for children with severe anaemia: a secondary analysis of the Transfusion and Treatment of African Children (TRACT) trial | TITLE |
|  | Giannakikou et al., 2015 | Anaesthesia for a patient with B-thalassaemia | TITLE |
|  | Geiszler et al., 2021 | Hyperhemolysis syndrome complicated by acute pulmonary hypertension and right ventricular failure in a patient with sickle cell disease | TITLE |
|  | Geletu et al., 2023 | A Case of Right Ventricular Failure Secondary to Acute Chest Syndrome Managed With Early Red Cell Exchange Transfusion | TITLE |
|  | Gellen et al., 2018 | Moderate-intensity endurance-exercise training in patients with sickle-cell disease without severe chronic complications (EXDRE): an open-label randomised controlled trial | TITLE |
|  | Ginwalla et al., 2018 | Cardiovascular evaluation and management of iron overload cardiomyopathy in sickle cell disease | TITLE |
|  | Ghaderian et al., 2012 | Tissue Doppler echocardiographic findings of left ventricle in children with sickle-cell anemia | ABSTRACT |
|  | Ghanta et al., 2018 | Cyclic guanosine monophosphate-dependent protein kinase i stimulators and activators are therapeutic alternatives for sickle cell disease | TITLE |
|  | Ghosh et al., 2016 | Nonhematopoietic Nrf2 dominantly impedes adult progression of sickle cell anemia in mice | TITLE |
|  | Ghunney et al., 2021 | In Africa, a High Proportion of Adults with HbSC Meet American Society of Hematology's Eligibility Criteria for Severe Sickle Cell Disease and Starting Hydroxyurea Therapy in a Clinical Trial Setting | TITLE |
|  | Gileles-Hillel et al., 2015 | Hemoglobinopathies and sleep - The road less traveled | TITLE |
|  | Gillespie et al., 2023 | The interplay of sleep disordered breathing, nocturnal hypoxemia, and endothelial dysfunction in sickle cell disease | TITLE |
|  | Gilli et al., 2016 | LDH and age are associated with hemolysis-endothelial dysfunction in HbSC patients | ABSTRACT |
|  | Gilyard et al., 2021 | Imaging review of sickle cell disease for the emergency radiologist | TITLE |
|  | Giray et al., 2023 | Longitudinal Changes in Cardiac Function Based on Serial Tissue Doppler and Doppler Imaging for Patients With Sickle Cell Anemia | TITLE |
|  | Giray Ersöz et al., 2021 | Vitrectomy due to vitreous hemorrhage and tractional retinal detachment secondary to eales’ disease | TITLE |
|  | Girgis et al., 2003 | Decreased exhaled nitric oxide in sickle cell disease: Relationship with chronic lung involvement | TITLE |
|  | Giri et al., 2023 | Ineffective Erythropoiesis with Aging in Adults with Sickle Cell Disease | TITLE |
|  | Giron Et al., 2020 | Description of potential presymptomatic markers for early detection of cardiopulmonary complications in pediatric patients with sickle cell disease | ABSTRACT |
|  | Girot et al., 2008 | New issues in adult sickle sell disease | TITLE |
|  | Gladwin et al., 2005 | Unraveling the hemolytic subphenotype of sickle cell disease | TITLE |
|  | Gladwin et al., 2004 | Haldane, hot dogs, halitosis, and hypoxic vasodilation: The emerging biology of the nitrite anion | TITLE |
|  | Gladwin et al., 2021 | Identifying adolescent and young adult patients with sickle cell disease at highest risk of death | TITLE |
|  | Gladwin et al., 2011 | Prevalence, risk factors and mortality of pulmonary hypertension defined by right heart catheterization in patients with sickle cell disease | ABSTRACT |
|  | Gladwin et al., 2015 | Revisiting the hyperhemolysis paradigm | TITLE |
|  | Gladwin et al., 2016 | Cardiovascular complications and risk of death in sickle-cell disease | ABSTRACT |
|  | Gladwinet al., 2017 | Cardiovascular complications in patients with sickle cell disease | ABSTRACT |
|  | Gladwin et al., 2004 | The biochemistry of nitric oxide, nitrite, and hemoglobin: role in blood flow regulation | Title |
|  | Glassberg et al., 2020 | Publication of data collection forms from NHLBI funded sickle cell disease implementation consortium (SCDIC) registry | TITLE |
|  | Glew et al., 2015 | Pulmonic Valve Repair in a Patient with Isolated Pulmonic Valve Endocarditis and Sickle Cell Disease | TITLE |
|  | Gladwin et al., 2010 | Pulmonary hypertension and NO in sickle cell | TITLE |
|  | Gladwin et al., 2014 | Risk factors for death in 632 patients with sickle cell disease in the United States and United Kingdom | ABSTRACT |
|  | Gladwin et al., 2005 | Cardiopulmonary complications of sickle cell disease: role of nitric oxide and hemolytic anemia | ABSTRACT |
|  | Gladwin et al., 2008 | Hemolysis-associated hypercoagulability in sickle cell disease: The plot (and blood) thickens! | ABSTRACT |
|  | Gladwin et al., 2011 | Pulmonary hypertension in sickle cell disease | ABSTRACT |
|  | Gladwin et al., 2012 | Cardiovascular abnormalities in sickle cell disease | ABSTRACT |
|  | Gladwin et al., 2004 | Pulmonary Hypertension as a Risk Factor for Death in Patients with Sickle Cell Disease | ABSTRACT |
|  | Gladwin et al., 2020 | Potential contribution of pulmonary thromboembolic disease in pulmonary hypertension in sickle cell disease | ABSTRACT |
|  | Gladwin et al., 2001 | Nitric oxide therapy in sickle cell disease | TITLE |
|  | Gladwin et al., 2008 | Pulmonary complications of sickle cell disease | ABSTRACT |
|  | Godbole et al., 2022 | Assessment of Cardio-Pulmonary and Renal Dysfunction As Well As Overall Outcomes in Sickle Cell Patients: A Community Hospital-Based Experience | ABSTRACT |
|  | Goel et al., 2010 | Cigarette Smoking Is An Independent Predictor of Chronic Pain In Sickle Cell Patients: Results From the Walk-PHaSST Study | TITLE |
|  | Goel et al., 2011 | Non-cardiopulmonary factors affecting the six-minute walk distance in patients with sickle cell disease: Results from the walk-PHaSST study | ABSTRACT |
|  | Goel et al., 2009 | Current epidemiology and hospitalization characteristics for acute chest syndrome: A nationally representative survey | ABSTRACT |
|  | Gomez et al., 2013 | Asthma Management in Sickle Cell Disease | Title |
|  | Gonsalves et al., 2010 | Endothelin-1-Induced Macrophage Inflammatory Protein-1β Expression in Monocytic Cells Involves Hypoxia-Inducible Factor-1α and AP-1 and Is Negatively Regulated by microRNA-195 | TITLE |
|  | Gokula et al., 2002 | Scleroderma with cardiac tamponade, hyperthyroidism and incidental papillary thyroid carcinoma | TITLE |
|  | Goligorsky et al., 2020 | The Cell “Coat of Many Colors” | TITLE |
|  | Gomez-Arteaga et al., 2023 | Haploidentical allogeneic stem cell transplantation with post-transplant cyclophosphamide and subsequent kidney transplant for patients with severe sickle cell disease with end-stage kidney disease (ESKD) | TITLE |
|  | Gonsalves et al., 2015 | Peroxisome proliferator-activated receptor-α-mediated transcription of miR-301a and miR-454 and their host gene SKA2 regulates endothelin-1 and PAI-1 expression in sickle cell disease | TITLE |
|  | Gonsalves et al., 2015 | Erythropoietin-mediated expression of placenta growth factor is regulated via activation of hypoxia-inducible factor-1α and post-transcriptionally by miR-214 in sickle cell disease | TITLE |
|  | Gonzales et al., 2023 | Caught in a vicious cycle: Cell-free hemoglobin and the pathogenesis of pulmonary hypertension | TITLE |
|  | Gonzales et al., 2022 | Hemin-Induced Endothelial Dysfunction and Endothelial to Mesenchymal Transition in the Pathogenesis of Pulmonary Hypertension Due to Chronic Hemolysis | TITLE |
|  | Gonzales et al., 2023 | TOLL-LIKE RECEPTOR 4 MEDIATES HEMIN-INDUCED PULMONARY ARTERY ENDOTHELIAL DYSFUNCTION AND EDEMA IN MURINE LUNGS | TITLE |
|  | González-Alonso et al., 2012 | A novel role for the red blood cell in the regulation of tissue O 2 supply | TITLE |
|  | Gorbach et al., 2012 | Infrared imaging of nitric oxide-mediated blood flow in human sickle cell disease | TITLE |
|  | Gordeuk et al., 2016 | Pathophysiology and treatment of pulmonary hypertension in sickle cell disease | ABSTRACT |
|  | Gordeuk et al., 2005 | Prevalence of Pulmonary Hypertension and Renal Dysfunction by Systemic Blood Pressure Categories in Sickle Cell Disease | ABSTRACT |
|  | Gordeuk et al., 2008 | Relative systemic hypertension in patients with sickle cell disease is associated with risk of pulmonary hypertension and renal insufficiency | TITLE |
|  | Gordeuk, et al., 2008 | Relative systemic hypertension in associated with risk of pulmonary patients with sickle cell disease is hypertension and renal insufficiency | TITLE |
|  | Goldsteinet al., 2006 | Primary prevention of ischemic stroke: a guideline from the American Heart Association/American Stroke Association Stroke Council: cosponsored by the Atherosclerotic Peripheral Vascular Disease Interdisciplinary Working Group; Cardiovascular Nursing Council; Clinical Cardiology Council; Nutrition, Physical Activity, and Metabolism Council; and the Quality of Care and Outcomes Research Interdisciplinary Working Group | TITLE |
|  | Gonsalves et al., 2010 | Hypoxia-mediated expression of 5-lipoxygenase-activating protein involves HIF-1alpha and NF-kappaB and microRNAs 135a and 199a-5p | TITLE |
|  | Gordeuk et al., 2011 | Elevated tricuspid regurgitation velocity and decline in exercise capacity over 22 months of follow up in children and adolescents with sickle cell anemia | ABSTRACT |
|  | Gosavi et al., 2010 | Atrial septal defect closure on cardiopulmonary bypass in a sickle cell anemia: role of hydroxyurea and partial exchange transfusion | TITLE |
|  | Gouraud et al., 2021 | Is Skeletal Muscle Dysfunction a Limiting Factor of Exercise Functional Capacity in Patients with Sickle Cell Disease? | TITLE |
|  | Gozdzik et al., 2019 | Perioperative Endocarditis Management in a Patient with Homozygous Sickle Cell Disease | TITLE |
|  | Greco et al., 2010 | Umbilical cord blood stem cells for myocardial repair and regeneration | TITLE |
|  | Gorgoneet al., 2022 | Point of care ultrasound detection of thrombus straddling a patent foramen ovale in a patient with acute chest syndrome | TITLE |
|  | Goswami et al., 2022 | LIPID PROFILE IN CHILDREN WITH SICKLE CELL DISEASE IN TWO URBAN COMMUNITY HOSPITALS | TITLE |
|  | Gotardo et al., 2019 | Anti-Inflammatory Effects of Hydroxyurea in a Murine Model of Chronic Intravascular Hemolysis | TITLE |
|  | Gotardo et al., 2023 | Molecular and cellular effects of in vivo chronic intravascular hemolysis and anti-inflammatory therapeutic approaches | TITLE |
|  | Gotardo et al., 2018 | Acute hemolysis induces pro-angiogenic molecule production and neovascularization in vivo | TITLE |
|  | Graham et al., 2007 | Sickle cell lung disease and sudden death: A retrospective/prospective study of 21 autopsy cases and literature review | TITLETitle |
|  | Graham Jr et al., 2004 | The effect of sickle cell disease on the lung | TITLE |
|  | Graham, L.  Et al., 2010 | Interview with the expert: LeRoy Graham, M.D | TITLE |
|  | Graham et al., 2004 | Sickle Cell Disease: Pulmonary Management Options | TITLE |
|  | Green et al., 2022 | Primary myelofibrosis in a patient with sickle cell disease | TITLE |
|  | Graffin et al., 2004 | The false..., reads the sizes | TITLE |
|  | Gravlee et al., 2015 | Cardiopulmonary bypass and mechanical support: Principles and practice: Fourth edition | TITLE |
|  | Grünberg et al., 2012 | Pulmonary vascular pathology | TITLE |
|  | Gualandro et al., 2007 | I guidelines for perioperative evaluation | TITLE |
|  | Greene et al., 2020 | Chapter 24 - Hemoglobin variant detection | TITLE |
|  | Greenough et al., 2004 | Sickle Cell Disease - Pulmonary Complications and a Proinflammatory State? | TITLE |
|  | Greenough et al., 2019 | The Lung in Sickle Cell Disease | TITLE |
|  | Greenoughet al., 2006 | SYSTEMIC DISEASE \| Sickle Cell Disease | TITLE |
|  | Griffiths et al., 2005 | Inhaled nitric oxide therapy in adults | TITLE |
|  | Grohmann et al., 2018 | Non-contraceptive benefits of LNGintrauterine system in anemia | TITLE |
|  | Gualandro et al., 2007 | Cardiopulmonary complications of sickle cell disease | ABSTRACT |
|  | Guedeney et al., 2015 | Cardiac involvement in hemoglobin SC disease compared to homozygous sickle-cell anemia | ABSTRACT |
|  | Guedeney et al., 2016 | Cardiac involvement in hemoglobin SC disease compared to homozygous sickle-cell anemia | ABSTRACT |
|  | Guilliams et al., 2019 | Arteriopathy Influences Pediatric Ischemic Stroke Presentation, but Sickle Cell Disease Influences Stroke Management | Title |
|  | Guiner et al., 2021 | Idiopathic effusive-constrictive pericarditis in a teenager | TITLE |
|  | Guedeney et al., 2016 | Cardiac involvement in the two main genotypes of sickle cell disease: Specificities of hemoglobin sickle cell compared to sickle cell anemia | ABSTRACT |
|  | Guillaumeet al., 2018 | Acute pulmonary hypertension during acute chest syndrome in adult patients with sickle cell disease: Is there a hypercoagulability state? | ABSTRACT |
|  | Gujja et al., 2010 | Iron overload cardiomyopathy: Better understanding of an increasing disorder | TITLE |
|  | Gulati et al., 2022 | 2021 AHA/ACC/ASE/CHEST/SAEM/SCCT/SCMR Guideline for the Evaluation and Diagnosis of Chest Pain: A Report of the American College of Cardiology/American Heart Association Joint Committee on Clinical Practice Guidelines | TITLE |
|  | Güvenç et al., 2005 | Sickle cell anemia patient with sarcoidosis-associated inguinal lymph node and lung infiltration | TITLE |
|  | Gueguenet al., 2014 | Sickle-cell disease stroke throughout life: a retrospective study in an adult referral center | TITLE |
|  | Gupta et al., 2021 | IL-18 mediates sickle cell cardiomyopathy and ventricular arrhythmias | TITLE |
|  | Gutsaevaet al., 2011 | Inhibition of cell adhesion by anti-P-selectin aptamer: a new potential therapeutic agent for sickle cell disease | TITLE |
|  | Gupta et al., 2012 | Breast cancer in sickle cell disease | TITLE |
|  | Guptaet al., 2015 | Morphine for the treatment of pain in sickle cell disease | TITLE |
|  | Gutiérrez-Díaz et al., 2012 | Splenectomy in sickle cell disease | TITLE |
|  | Habara et al., 2016 | Minireview: Genetic basis of heterogeneity and severity in sickle cell disease | TITLE |
|  | Habara et al., 2016 | Genetic basis of heterogeneity and severity in sickle cell disease | TITLE |
|  | Habibiet al., 2004 | Acute complications in sickle cell disease | TITLE |
|  | Habibi et al., 2016 | Delayed hemolytic transfusion reaction in adult sickle-cell disease: presentations, outcomes, and treatments of 99 referral center episodes | TITLE |
|  | Habibiet al., 2021 | Causes and Circumstances of Death: Analysis in 266 Sickle Cell Adult Patients | Title |
|  | Hackett et al., 2002 | Medical advisors for commercial adventures: Coming of age? | TITLE |
|  | Hagar et al., 2015 | Teasing Apart Pain in Sickle Cell Disease | TITLE |
|  | Hage et al., 2018 | Disseminated Intravascular Coagulation and Acute Liver Injury from Ethanol Embolization of an Arteriovenous Malformation | TITLE |
|  | Hambley et al., 2019 | Intracardiac or intrapulmonary shunts were present in at least 35% of adults with homozygous sickle cell disease followed in an outpatient clinic | Title |
|  | Hammoudi et al., 2014 | Subclinical left ventricular systolic impairment in steady state young adult patients with sickle-cell anemia | Title |
|  | Hammoudi et al., 2023 | Heart failure in SCA: still challenging | TITLE |
|  | Hagar et al., 2008 | Clinical differences between children and adults with pulmonary hypertension and sickle cell disease | ABSTRACT |
|  | Hagar et al., 2008 | Advances in clinical research in sickle cell disease | ABSTRACT |
|  | Haider et al., 2009 | Prevalence and predictors of chronic organ damage in adults with sickle cell disease | ABSTRACT |
|  | Hajouj et al., 2022 | S‐Nitrosylation of Paraxonase 1 (PON1) Elevates Its Hydrolytic and Antioxidant Activities | TITLE |
|  | Halabi-Tawil et al., 2008 | Sickle cell leg ulcers: a frequently disabling complication and a marker of severity | TITLE |
|  | Hallmark et al., 2021 | Nitric oxide and sickle cell disease-Is there a painful connection? | TITLE |
|  | Halphen et al., 2014 | Severe Nocturnal and Postexercise Hypoxia in Children and Adolescents with Sickle Cell Disease | TITLE |
|  | Halphen et al., 2012 | Children with sickle cell anemia experience severe oxygen desaturation during night and after six-minute walk distance test | ABSTRACT |
|  | Hamideh et al., 2013 | Sickle cell disease related mortality in the United States (1999-2009) | TITLE |
|  | Ha et al., 2023 | Association of Sickle Cell Disease With Severe Maternal Morbidity | TITLE |
|  | Hadeedet al., 2015 | Endothelial Function and Vascular Properties in Children with Sickle Cell Disease | Title |
|  | Hajoujet al., 2022 | S-Nitrosylation of Paraxonase 1 (PON1) Elevates Its Hydrolytic and Antioxidant Activities | TITLE |
|  | Hallioglu et al., 2011 | Gated myocardial perfusion scintigraphy in children with sickle cell anemia: correlation with echocardiography | ABSTRACT |
|  | Hammoudi et al., 2022 | Altered cardiac reserve is a determinant of exercise intolerance in sickle cell anaemia patients | TITLE |
|  | Hammoudiet al., 2015 | Left atrial volume is not an index of left ventricular diastolic dysfunction in patients with sickle cell anaemia | ABSTRACT |
|  | Han et al., 2017 | Use of anti-inflammatory analgesics in sickle-cell disease | TITLE |
|  | Hankins et al., 2010 | Ventricular diastolic dysfunction in sickle cell anemia is common but not associated with myocardial iron deposition | TITLE |
|  | Harban et al., 2008 | Cardiopulmonary bypass for surgical correction of congenital heart disease in children with sickle cell disease: a case series | TITLE |
|  | Harmon et al., 2014 | Pathogeneses of sudden cardiac death in national collegiate athletic association athletes | TITLE |
|  | Hawley et al., 2009 | Sickle cell disease: two fatalities due to bone marrow emboli in patients with acute chest syndrome | TITLE |
|  | Hammoudi et al., 2020 | Cardiovascular manifestations of sickle cell disease | ABSTRACT |
|  | Hamzaoui et al., 2023 | Lung manifestations of sickle-cell disease | ABSTRACT |
|  | Hamzaoui et al., 2023 | Manifestations respiratoires de la drépanocytose | TITLE |
|  | Han et al., 2016 | Impact of a Clinical Pharmacy Service on the Management of Patients in a Sickle Cell Disease Outpatient Center | TITLE |
|  | Hanes et al., 2012 | Pruritus in sickle cell disease patients: Preliminary data | TITLE |
|  | Hanafy et al., 2018 | Posterior reversible encephalopathy syndrome secondary to asymptomatic poststreptococcal glomerulonephritis in a child with sickle cell anemia: A case report | TITLE |
|  | Hanes et al., 2013 | Assessment and prediction of pruritus in sickle cell disease patients: a preliminary study | TITLE |
|  | Hankins et al., 2009 | Pharmacotherapy in sickle cell disease - state of the art and future prospects | TITLE |
|  | Hannoushet al., 2013 | Ventricular mechanics in sickle cell disease | TITLE |
|  | Hansell et al., 2002 | Small-vessel diseases of the lung: CT-pathologic correlates | TITLE |
|  | Hao et al., 2023 | Multifaceted functions of Drp1 in hypoxia/ischemia-induced mitochondrial quality imbalance: from regulatory mechanism to targeted therapeutic strategy | TITLE |
|  | Haque et al., 2002 | Pulmonary hypertension in sickle cell hemoglobinopathy: a clinicopathologic study of 20 cases | ABSTRACT |
|  | Hardit et al., 2022 | Evolving Strategies in the Management of Sickle Cell Disease in the 21st Century and the Role of the Pediatrician | TITLE |
|  | Harrington et al., 2017 | Longitudinal Analysis of Echocardiographic Abnormalities in Children With Sickle Cell Disease | ABSTRACT |
|  | Harmon et al., 2007 | Update on sideline and event preparation for management of sudden cardiac arrest in athletes | TITLE |
|  | Harrington et al., 2019 | Pulmonary Hypertension in Children with Sickle Cell Disease: a Review of the Current Literature | ABSTRACT |
|  | Hashemzehi et al., 2021 | Diverse manifestations of a sickle cell crisis | TITLE |
|  | Hassell et al., 2011 | Pulmonary hypertension, tricuspid regurgitant velocity screening, and the nitric oxide pathway | ABSTRACT |
|  | Hassell et al., 2014 | Practice guideline for pulmonary hypertension in sickle cell: Direct evidence needed before universal adoption | TITLE |
|  | Hassell et al., 2000 | Transient severe pulmonary hypertension during acute chest syndrome (ACS) in adults with sickle cell disease | ABSTRACT |
|  | Hassinger et al., 2023 | Introduction to the pediatric sleep medicine special issue | TITLE |
|  | Hatzipantelis et al., 2013 | Endothelial activation and inflammation biomarkers in children and adolescents with sickle cell disease | TITLE |
|  | Haw et al., 2018 | Pulmonary hypertension in chronic hemolytic anemias: Pathophysiology and treatment | TITLE |
|  | Haworth et al., 2006 | Role of the endothelium in pulmonary arterial hypertension | TITLE |
|  | Hayeset al., 2019 | Home oxygen therapy for children an official American Thoracic Society clinical practice guideline | TITLE |
|  | Hayes et al., 2014 | Pulmonary hypertension in sickle cell disease | ABSTRACT |
|  | Haymann et al., 2017 | Renin-angiotensin system blockade promotes a cardio-renal protection in albuminuric homozygous sickle cell patients | TITLE |
|  | Hay et al., 2017 | Global, regional, and national disability-adjusted life-years (DALYs) for 333 diseases and injuries and healthy life expectancy (HALE) for 195 countries and territories, 1990-2016: A systematic analysis for the Global Burden of Disease Study 2016 | TITLE |
|  | Haywood et al., 2009 | The burden of hospital readmissions for patients with sickle cell disease in California and North Carolina: 2004 to 2007 | TITLE |
|  | Haywood et al., 2009 | Cardiovascular function and dysfunction in sickle cell anemia | TITLE |
|  | Hazin-Costa et al., 2019 | Chemokines in pregnant women with sickle cell disease | TITLE |
|  | Hebbel et al., 2011 | Reconstructing sickle cell disease: A data-based analysis of the "hyperhemolysis paradigm" for pulmonary hypertension from the perspective of evidence-based medicine | TITLE |
|  | Hebbel et al., 2020 | The multifaceted role of ischemia/reperfusion in sickle cell anemia | TITLE |
|  | Hebbel et al., 2016 | Microparticles in sickle cell anaemia: promise and pitfalls | TITLE |
|  | Hebbel et al., 2021 | Multiple inducers of endothelial NOS (eNOS) dysfunction in sickle cell disease | TITLE |
|  | Heeneyet al., 2014 | Clinical outcomes among patients with sickle cell disease: 24-month follow-up on a 3-year, prospective, noninterventional registry | ABSTRACT |
|  | Hebertet al., 2022 | Outcomes of kidney donors with sickle cell trait: A preliminary analysis | TITLE |
|  | Hedreville et al., 2009 | Central retinal vein occlusion in a sickle cell trait carrier after a cycling race | TITLE |
|  | Heredia-Gutiérrez et al., 2021 | Cerebral aneurysms in pediatrics: a case report and review of the literature | TITLE |
|  | Herron et al., 2023 | Right atrial thrombus removal with use of the ŌNŌ retrieval device | Title |
|  | Heeney et al., 2015 | Comparison of clinical outcomes between adult and pediatric patients with sickle cell disease (SCD): 3-year follow-up in a prospective, longitudinal, noninterventional registry trial | ABSTRACT |
|  | Heeney et al., 2012 | 12-Month follow-up for patients with sickle cell disease in an ongoing 3-year, prospective, non-interventional registry trial | ABSTRACT |
|  | Heeneyet al., 2013 | Clinical Outcomes For Patients With Sickle Cell Disease: 24-Month Follow-Up In An Ongoing 3-Year, Prospective, Non-Interventional Registry Trial | ABSTRACT |
|  | Heeneyet al., 2011 | Baseline characteristics of patients with sickle cell disease in an ongoing 5-year, prospective, noninterventional registry trial | ABSTRACT |
|  | Heeneyet al., 2021 | Association of Hospitalization Due to Vaso-Occlusive Crisis with Subsequent Sickle Cell Disease-Related Organ Damage Hospitalization: Retrospective Analysis of 3-Year Observational Study Data | ABSTRACT |
|  | Helvaciet al., 2014 | Acute chest syndrome in severity of sickle cell diseases | TITLE |
|  | Helvaci et al., 2014 | Autosplenectomy in severity of sickle cell diseases | TITLE |
|  | Helvaci et al., 2022 | Acute chest syndrome and pulmonary hypertension in sickle cell diseases | TITLE |
|  | Helvaci et al., 2014 | Sickle cell diseases and ileus | TITLE |
|  | Helvaci et al., 2013 | Gender differences in severity of sickle cell diseases in non-smokers | TITLE |
|  | Helvaci et al., 2015 | Tonsilectomy in sickle cell diseases | TITLE |
|  | Helvaci et al., 2015 | Mortal quintet of sickle cell diseases | TITLE |
|  | Helvaci et al., 2014 | Smoking and sickle cell diseases | TITLE |
|  | Hemming et al., 2004 | Pro: Exchange transfusion is required for sickle cell trait patients undergoing cardiopulmonary bypass | TITLE |
|  | Herlihy et al., 2010 | Improvement in right ventricular function after using inhaled nitric oxide in patients with sickle cell disease and severe acute chest syndrome | ABSTRACT |
|  | Herbst et al., 2019 | Diffuse Ischemic Strokes and Sickle Cell Crisis Induced by Disseminated Anaplasmosis: A Case Report | Title |
|  | Hervé et al., 2010 | Characterization of a murine model of acute chest syndrome | TITLE |
|  | Higuera et al., 2015 | Hemostasis alterations associated to complications in sickle cell syndrome | TITLE |
|  | Higuera et al., 2014 | Hemostasis alterations in sickle cell syndrome | TITLE |
|  | Hildreth et al., 2008 | Sickle cell vasculopathy | TITLE |
|  | Hill et al., 2005 | Nitric Oxide Consumption and Pulmonary Hypertension in Patients with Paroxysmal Nocturnal Hemoglobinuria | TITLE |
|  | Hill et al., 2002 | A nitric oxide overview | TITLE |
|  | Hirani et al., 2011 | Acute chest syndrome and other pulmonary manifestations of sickle cell disease | TITLE |
|  | Hijaziet al., 2005 | Pulmonary function studies in Kuwaiti children with sickle cell disease and elevated Hb F | ABSTRACT |
|  | Hladunet al., 2013 | Results of hematopoietic stem cell transplantation in hemoglobinopathies: thalassemia major and sickle cell disease | TITLE |
|  | Hicks et al., 2023 | More Than Meets the Eye: Orbital Swelling in an Adolescent with Sickle Cell Disease | TITLE |
|  | Hill et al., 2006 | Increased cerebral blood flow velocity in children with mild sleep-disordered breathing: A possible association with abnormal neuropsychological function | TITLE |
|  | Holmes et al., 2012 | Novel cause of sudden cardiac death: IgG4-related disease | TITLE |
|  | Homsy et al., 2019 | An unusual case of low hemoglobin oxygen saturation | TITLE |
|  | Honsel et al., 2019 | Comparison between adult patients with sickle cell disease of sub-saharan African origin born in metropolitan France and in sub-saharan Africa | TITLE |
|  | Hosseini et al., 2010 | Right ventricular approach for securing the aortic homograft to the interventricular septum in bacterial endocarditis | TITLE |
|  | Howard et al., 2018 | Overnight auto-adjusting continuous airway pressure+standard care compared with standard care alone in the prevention of morbidity in sickle cell disease phase II (POMS2b): Study protocol for a randomised controlled trial | TITLE |
|  | Hoet al., 2023 | Diaphragm excursion correlates with performance and ventilation on the 6-min walk test in children with sickle cell disease | TITLE |
|  | Hoppe et al., 2011 | A pilot study of the short-term use of simvastatin in sickle cell disease: effects on markers of vascular dysfunction | TITLE |
|  | Hostyn et al., 2013 | Evaluation of functional capacity for exercise in children and adolescents with sickle-cell disease through the six-minute walk test | ABSTRACT |
|  | Howardet al., 2019 | Prevention of Morbidity in Sickle Cell Disease (POMS2a)-overnight auto-adjusting continuous positive airway pressure compared with nocturnal oxygen therapy: a randomised crossover pilot study examining patient preference and safety in adults and children | TITLE |
|  | Hoang et al., 2021 | Metabolic Intersection of Cancer and Cardiovascular Diseases: Opportunities for Cancer Therapy | TITLE |
|  | Hoffman et al., 2011 | Pulmonary hypertension in sickle cell disease | ABSTRACT |
|  | Hoffman et al., 2011 | Pulmonary vascular resistance and viscosity: The forgotten factor | TITLE |
|  | Holding et al., 2008 | Do orphan medicines benefit patients? | TITLE |
|  | Hoogenboom et al., 2022 | Clinical outcomes of COVID-19 in patients with sickle cell disease and sickle cell trait: A critical appraisal of the literature | TITLE |
|  | Howardet al., 2018 | Overnight auto-adjusting continuous airway pressure + standard care compared with standard care alone in the prevention of morbidity in sickle cell disease phase II (POMS2b): study protocol for a randomised controlled trial | TITLE |
|  | Hsiehet al., 2014 | Nonmyeloablative HLA-matched sibling allogeneic hematopoietic stem cell transplantation for severe sickle cell phenotype | TITLE |
|  | Hulbert et al., 2022 | Organ function indications and potential improvements following curative therapy for sickle cell disease | TITLE |
|  | Hoppe et al., 2019 | Sickle Cell Disease: Monitoring, Current Treatment, and Therapeutics Under Development | TITLE |
|  | Hoppe et al., 2011 | NOVEL THERAPIES TARGETING THE ENDOTHELIUM IN SICKLE CELL DISEASE | TITLE |
|  | Hopper et al., 2018 | Where is the clinical breakthrough of heme oxygenase-1/carbon therapeutics? | TITLE |
|  | Hopper et al., 2022 | Revisiting Arginine Therapy for Sickle Cell Acute Vasoocclusive Painful Crisis | TITLE |
|  | Houde et al., 2012 | GPCR models of pain in cardiovascular diseases: Contributions of kinins and endothelins | TITLE |
|  | Houwing et al., 2019 | Sickle cell disease: Clinical presentation and management of a global health challenge | TITLE |
|  | Howard et al., 2013 | The role of blood transfusion in sickle cell disease | Title |
|  | Howard et al., 2015 | Transfusion therapy in sickle cell disease | TITLE |
|  | Howard et al., 2007 | Sickle cell disease in North Europe | TITLE |
|  | Howard et al., 2007 | Haemoglobinopathies | TITLE |
|  | Howard et al., 2012 | The obstetric management of sickle cell disease | TITLE |
|  | Howell et al., 2023 | Burden of Aging: Health Outcomes Among Adolescents and Young Adults With Sickle Cell Disease | TITLE |
|  | Howland et al., 2018 | Estimating the hospital delivery costs associated with severe maternal morbidity in New York City, 2008-2012 | TITLE |
|  | Hritani et al., 2023 | An unusual left atrial Rosai-Dorfman involvement in sickle cell disease patient | TITLE |
|  | Hsieh et al., 2015 | Sickle cell disease is not so benign | TITLE |
|  | Hsiehet al., 2011 | Non-Myeloablative Allogeneic Hematopoietic Stem Cell Transplantation (allo-HSCT) for patients with Severe Sickle Cell Disease (SCD) | TITLE |
|  | Hsieh et al., 2006 | A Novel Allogeneic Transplant Conditioning Regimen Designed for Tolerance Induction in Patients with Severe Sickle Cell Disease | TITLE |
|  | Hsieh et al., 2007 | Improvement in Hemolytic Parameters and Tricuspid Regurgitant Jet Velocity (TRV) Following Non-Myeloablative Allogeneic Stem Cell Transplantation (allo-SCT) in Adults with Severe Sickle Cell Disease (SCD) | ABSTRACT |
|  | Hsu et al., 2006 | Hemolysis in Sickle Cell Mice Causes Pulmonary Hypertension Due to Global Impairment in Nitric Oxide Bioavailability | TITLE |
|  | Hsue et al., 2008 | Pulmonary arterial hypertension and HIV infection: Authors' reply | TITLE |
|  | Huang et al., 2006 | Lack of allosterically controlled intramolecular transfer of nitric oxide from the heme to cysteine in the β subunit of hemoglobin | TITLE |
|  | Humbert et al., 2009 | Update in pulmonary hypertension 2008 | TITLE |
|  | Humbert et al., 2012 | Early detection and management of pulmonary arterial hypertension | ABSTRACT |
|  | Humbert et al., 2006 | The need for national registries in rare diseases | TITLE |
|  | Hundekar et al., 2012 | Level of nitric oxide and antioxidant vitamins in sickle cell anaemia patients | Title |
|  | Hunter et al., 2016 | Hemgolobin S-C Disease at the Emory University Georgia Comprehensive Sickle Cell Center at Grady Health System | ABSTRACT |
|  | Hummadi et al., 2019 | Cardiac Arrhythmia in a Patient with Sickle Cell Anemia and Falciparum Malaria Treated with Intravenous Artesunate | TITLE |
|  | Hyacinthet al., 2021 | Association of Sickle Cell Trait With Incidence of Coronary Heart Disease Among African American Individuals | TITLE |
|  | Ibanez et al., 2016 | Anti-HI can cause a severe delayed hemolytic transfusion reaction with hyperhemolysis in sickle cell disease patients | TITLE |
|  | Ibemere et al., 2023 | Characterising the prevalence of overweight and obese status among adults with sickle cell disease | TITLE |
|  | Ichinose et al., 2017 | Inhaled Nitric Oxide-Current Practice and Future Potential Uses and Development | TITLE |
|  | Idrees et al., 2008 | Saudi guidelines on diagnosis and treatment of pulmonary arterial hypertension | TITLE |
|  | Idris et al., 2022 | Sickle cell disease as an accelerated aging syndrome | TITLE |
|  | Ikeda et al., 2012 | Laser speckle contrast imaging characterizes delayed reperfusion after transient brachial artery occlusion in patients with sickle cell diseas | TITLE |
|  | Ilonze et al., 2023 | Cardiovascular complications of sickle cell disease: A primer for the general clinician | ABSTRACT |
|  | Imran et al., 2018 | An optimist approach for recognition of thrombotic haemoglobinopathy. A threat to survival an experience from developing country Pakistan | TITLE |
|  | Imtiaz et al.,2021 | Clinical and physiological characteristics of, medically treated, chronic thromboembolic pulmonary hypertension patients in Saudi Arabia: A single center experience | TITLE |
|  | Inam et al., 2023 | Outcomes and long-term effects of hematopoietic stem cell transplant in sickle cell disease | TITLE |
|  | Iannucciet al., 2016 | Sickle Cell Disease with Cyanotic Congenital Heart Disease: Long-Term Outcomes in 5 Children | TITLE |
|  | Ibrahim el et al., 2012 | Assessment of cardiac iron deposition in sickle cell disease using 3.0 Tesla cardiovascular magnetic resonance | TITLE |
|  | Ikobo et al., 2019 | Evaluation of the Follow-Up and State of Adolescents with Sickle-Cell Disease in Brazzaville (Congo) | TITLE |
|  | Igbokwe et al., 2022 | Kidney transplantation in sickle cell disease patients: Case series and experience from a Nigerian kidney transplant center | TITLE |
|  | Ilodibia et al., 2022 | Electrocardiographic findings in Nigerian athletes with the sickle cell trait | ABSTRACT |
|  | Imran et al., 2021 | Eosinophilic Myocarditis in a Patient With Sickle Cell Disease | TITLE |
|  | Inusa et al., 2014 | Subarachnoid haemorrhage and cerebral vasculopathy in a child with sickle cell anaemia | TITLE |
|  | Isaac et al., 2022 | Systemic thrombolysis for acute central retinal artery occlusion in sickle cell disease: Case report | TITLE |
|  | Intzes et al., 2013 | Pulmonary function abnormalities and asthma are prevalent in children with sickle cell disease and are associated with acute chest syndrome | TITLE |
|  | Inamoet al., 2009 | Pulmonary hypertension does not affect the autonomic nervous system dysfunction of sickle cell disease | ABSTRACT |
|  | Inati et al., 2008 | Childhood anemias: A success story of basic science and clinical care | TITLE |
|  | Inati et al., 2019 | Sickle Cell Disease Burden in North Lebanon | TITLE |
|  | Inati et al., 2008 | Sickle cell disease: New insights into pathophysiology and treatment | TITLE |
|  | Inati et al., 2009 | Magnetic resonance imaging T2∗ in the evaluation of cardiac Iron overload in patients with sickle cell disease | TITLE |
|  | Inati et al., 2009 | Absence of cardiac siderosis by MRI T2* despite transfusion burden, hepatic and serum iron overload in Lebanese patients with sickle cell disease | TITLE |
|  | Indik et al., 2016 | Associations of prolonged QTc in sickle cell disease | TITLE |
|  | Isaza-López et al., 2020 | Characterization of kidney complications in patients with sickle cell anemia | TITLE |
|  | Isgro et al., 2017 | Spirometric Evaluation of Pulmonary Function in Nigerian Children underwent Bone Marrow Transplantation for Sickle Cell Anemia | TITLE |
|  | Ishola et al., 2016 | Risk factors and co-morbidities in adolescent thromboembolism are different than those in younger children | ABSTRACT |
|  | Inusa et al., 2017 | Sickle Cell Disease | TITLE |
|  | Inusa et al., 2023 | Global burden of transfusion in sickle cell disease | TITLE |
|  | Iolascon et al., 2017 | Recommendations regarding splenectomy in hereditary hemolytic anemias | TITLE |
|  | Iovino et al., 2013 | Signalling or binding: The role of the platelet-activating factor receptor in invasive pneumococcal disease | TITLE |
|  | Ipson et al., 2023 | CLINICAL FACTORS AFFECTING 6-MINUTE WALK TEST PERFORMANCE IN CHILDREN WITH SICKLE CELL DISEASE | ABSTRACT |
|  | Irwin et al., 2010 | Low dose chronically infused hemoglobin induces pulmonary hypertension or excerbates hypoxia-induced pulmonary hypertension | TITLE |
|  | Irwin et al., 2015 | Hemoglobin-induced lung vascular oxidation, inflammation, and remodeling contribute to the progression of hypoxic pulmonary hypertension and is attenuated in rats with repeated-dose haptoglobin administration | TITLE |
|  | Isa et al., 2020 | Sickle cell disease clinical phenotypes in Nigeria: A preliminary analysis of the Sickle Pan Africa Research Consortium Nigeria database | ABSTRACT |
|  | Isenberget al., 2020 | Atlas of Genetics and Cytogenetics in Oncology and Haematology | TITLE |
|  | Ishak Gabra et al., 2019 | Pulmonary Arterial Hypertension and Therapeutic Interventions | TITLE |
|  | Isma'eel et al., 2006 | Screening for inherited thrombophilia might be warranted among Eastern Mediterranean sickle-βeta-0 thalassemia patients | TITLE |
|  | Isma'eel et al., 2008 | Chronic transfusion, iron overload and cardiac dysfunction: A multi-dimensional perspective | TITLE |
|  | Ivy et al., 2009 | Non-congenital heart disease associated pediatric pulmonary arterial hypertension | ABSTRACT |
|  | Iyamu et al., 2007 | In vitro evidence of the inhibitory capacity of chloroquine on arginase activity in sickle erythrocytes | TITLE |
|  | Jackson et al., 2023 | RBC Exchange Transfusion As an Adjunct Therapy to Control Iron Overload in Patients with Transfusion-Dependent Thalassemia | TITLE |
|  | Jabbar et al., 2023 | Impact of Vitamin D in the improvement of respiratory function in sickle cell disease adult patients | TITLE |
|  | Jackson et al., 2023 | 8-Aminopurines in the Cardiovascular and Renal Systems and beyond | TITLE |
|  | Jacob et al., 2015 | Thrombospondin-1 gene polymorphism is associated with estimated pulmonary artery pressure in patients with sickle cell anemia | ABSTRACT |
|  | Jain et al., 2014 | Acute pancreatitis complicating severe dengue | TITLE |
|  | Jalaly et al., 2016 | A 30-year-old patient who refuses to be drug tested | TITLE |
|  | Jasinski et al., 2019 | Catastrophic Delayed Hemolytic Transfusion Reaction in a Patient with Sickle Cell Disease Without Alloantibodies: Case Report and Review of Literature | TITLE |
|  | Jacobs et al., 2013 | Inflammatory Biomarkers and Cardiovascular Complications in Sickle Cell Disease: A Review | TITLE |
|  | Jagadeeswaran et al., 2017 | Evolving treatment paradigms in sickle cell disease | TITLE |
|  | Jain et al., 2020 | Current practices in the management of beta-hemoglobinopathies | TITLE |
|  | Jain et al., 2018 | National trends of sickle cell disease related adult hospitalizations | TITLE |
|  | Jain et al., 2017 | Acute Chest Syndrome in Children with Sickle Cell Disease | TITLE |
|  | Jain et al., 2010 | Arginine metabolism and nitric oxide bioavailability in sickle cell disease | TITLE |
|  | Jain et al., 2013 | Unraveling restrictive chronic lung disease in sickle cell disease | TITLE |
|  | Jacksonet al., 2012 | Vitamin D deficiency and comorbidities in children with sickle cell anemia | TITLE |
|  | Jajaet al., 2008 | Cardiac and autonomic responses to change in posture or vitamin C supplementation in sickle cell anemia subjects | TITLE |
|  | Jakubik et al., 2000 | Care of the child with sickle cell disease: acute complications | TITLE |
|  | Jana et al., 2018 | Hemoglobin oxidation-dependent reactions promote interactions with band 3 and oxidative changes in sickle cell-derived microparticles | TITLE |
|  | Janka et al., 2010 | Increased pulmonary pressures and myocardial wall stress in children with severe malaria | ABSTRACT |
|  | Jariwalat al., 2019 | Comparative study of alloimmunization against red cell antigens in sickle cell disease & thalassaemia major patients on regular red cell transfusion | TITLE |
|  | Jain et al., 2018 | Role of automated red cell exchange in acute and chronic complications of sickle cell disease | ABSTRACT |
|  | Jawadet al., 2018 | Dynamic blood flow phantom with negative and positive photoacoustic contrasts | TITLE |
|  | Jawad et al., 2022 | Towards rainbow portable Cytophone with laser diodes for global disease diagnostics | TITLE |
|  | Jhaveri et al., 2021 | Association of Anemia and Blood Pressure With Novel Markers of Diastolic Function in Pediatric Sickle Cell Disease | TITLE |
|  | Ji et al., 2014 | Anion exchange HPLC isolation of high-density lipoprotein (HDL) and on-line estimation of proinflammatory HDL | TITLE |
|  | Jaïs et al.,2003 | An extreme consequence of splenectomy in dehydrated hereditary stomatocytosis: gradual thrombo-embolic pulmonary hypertension and lung-heart transplantation | Title |
|  | James et al., 2019 | Management Strategies and Satisfaction Levels in Patients with Sickle Cell Disease: Interim Results from the International Sickle Cell World Assessment Survey (SWAY) | TITLE |
|  | Jamoussi et al., 2019 | Pulmonary hypertension in sickle cell disease: Acute or chronic complication? | ABSTRACT |
|  | Jana, et al., 2017 | Oxidized Mutant Human Hemoglobins S and E Induce Oxidative Stress and Bioenergetic Dysfunction in Human Pulmonary Endothelial Cells | TITLE |
|  | Jang et al., 2021 | PPARg increases HUWE1 to attenuate NF-kB/p65 and sickle cell disease with pulmonary hypertension | TITLE |
|  | Janz et al., 2015 | The role of red blood cells and cell-free hemoglobin in the pathogenesis of ARDS | TITLE |
|  | Jerathet al., 2011 | Pulmonary endarterectomy in sickle cell haemoglobin C disease | TITLE |
|  | Jehan et al., 2014 | To determine the efficacy of inhaled corticosteroids compared to montelukast in reducing exacerbation in uncontrolled asthma in children 6 months to 5 years | Title |
|  | Jiao et al., 2022 | Health State Utilities for Sickle Cell Disease: A Catalog Prepared From a Systematic Review | TITLE |
|  | Jison et al., 2003 | Hemolytic anemia-associated pulmonary hypertension of sickle cell disease and the nitric oxide/arginine pathway | ABSTRACT |
|  | Jindal et al., 2011 | Acute chest pain | TITLE |
|  | Johnet al., 2020 | Implications of covid-19 infections in sickle cell disease | TITLE |
|  | Johnso et al., 2015 | Options in the management of sickle cell disease | TITLE |
|  | Johnson et al., 2005 | The acute chest syndrome | TITLE |
|  | Johnson et al., 2008 | Sickle-cell disease | TITLE |
|  | Johnson et al., 2016 | Sickle-Cell Disease | TITLE |
|  | Johnson et al., 2021 | Exercise-induced changes of vital signs in adults with sickle cell disease | TITLE |
|  | Jonassaint et al., 2011 | How is patient socioeconomic status related to health care utilization? | TITLE |
|  | Jonassaint et al., 2011 | Low socioeconomic status is associated with increased frequency of hospitalizations and acute care visits for treatment of vaso-occlusive pain crises among adult patients with sickle cell disease | Abstract |
|  | Jordan et al., 2011 | Sickle cell day hospital at memorial regional hospital - A solution that works | TITLE |
|  | Josephson et al., 2007 | Transfusion in the Patient With Sickle Cell Disease: A Critical Review of the Literature and Transfusion Guidelines | TITLE |
|  | Joshi et al., 2010 | Uric Acid as a potential biomarker of pulmonary arterial hypertension in patients with sickle cell disease | TITLE |
|  | Joye et al., 2023 | Dual-energy computed tomography to detect early pulmonary vascular changes in children with sickle cell disease: a pilot study | TITLE |
|  | Johnsonet al., 2015 | Coronary artery dilation and left ventricular hypertrophy do not predict morbidity in children with sickle cell disease | TITLE |
|  | Johnsont et al., 2015 | A triad of linezolid toxicity: hypoglycemia, lactic acidosis, and acute pancreatitis | TITLE |
|  | Journeycake et al., 2003 | Thrombotic complications of central venous catheters in children | Title |
|  | Jube et al., 2017 | Significance of lower airway obstruction and vitamin d deficiency in sickle cell disease | TITLE |
|  | Judy et al., 2020 | RNA Seq Profiles and Bioinformatics Validation in a Large Sample of Sickle Cell Disease Patients | TITLE |
|  | Junagade et al., 2013 | Sickle cell disease and pregnancy | TITLE |
|  | Junqueira et al., 2013 | Right and left ventricular function and myocardial scarring in adult patients with sickle cell disease: a comprehensive magnetic resonance assessment of hepatic and myocardial iron overload | ABSTRACT |
|  | Jutant et al., 2021 | Endothelial dysfunction and hypercoagulability in severe sickle-cell acute chest syndrome | TITLE |
|  | Junior et al., 2021 | Cardiovascular benefits of a home-based exercise program in patients with sickle celldisease | TITLE |
|  | Kaddah et al., 2017 | Plasma chitotriosidase and carotid intima–media thickness in children with sickle cell disease | TITLE |
|  | Kaddam et al., 2019 | Acacia Senegal (Gum Arabic) Supplementation Modulate Lipid Profile and Ameliorated Dyslipidemia among Sickle Cell Anemia Patients | Title |
|  | Kalantari et al., 2016 | Group 5 Pulmonary Hypertension: The Orphan's Orphan Disease | TITLE |
|  | Kalff et al., 2010 | The impact of a regular erythrocytapheresis programme on the acute and chronic complications of sickle cell disease in adults | TITLE |
|  | Kalpatthi et al., 2009 | Low prevalence of pulmonary artery hypertension in children with sickle cell anemia | ABSTRACT |
|  | Kalpatthi et al., 2018 | Measuring success: Utility of biomarkers in sickle cell disease clinical trials and care | TITLE |
|  | Kalra et al., 2018 | Placenta growth factor mediated gene regulation in sickle cell disease | TITLE |
|  | Kamdar et al., 2021 | COVID-19 outcomes in a large pediatric hematology-oncology center in Houston, Texas | TITLE |
|  | Kamdar et al., 2011 | Sudden death in adult sickle cell patients: A single institutional experience | TITLE |
|  | Kane et al., 2001 | Echocardiographic aspects in pediatric patients with sickle cell disease | ABSTRACT |
|  | Kang et al., 2017 | Peroxisome proliferator-activated receptor g regulates the v-ets avian erythroblastosis virus E26 oncogene homolog 1/microrna-27a axis to reduce endothelin-1 and endothelial dysfunction in the sickle cell mouse lung | TITLE |
|  | Kalish et al., 2015 | Dietary ω-3 fatty acids protect against vasculopathy in a transgenic mouse model of sickle cell disease | TITLE |
|  | Kang et al., 2011 | Regional and systemic hemodynamic responses following the creation of a murine arteriovenous fistula | TITLE |
|  | Karan et al., 2023 | Two cases of refractory methicillin-susceptible staphylococcus aureus endocarditis responsive to ertapenem | TITLE |
|  | Karavassilis et al., 2021 | Multiple thromboembolic events associated with bilateral superior vena cava and anomalous drainage into the left atrium | TITLE |
|  | Kaiafa et al., 2017 | Anemia and stroke: Where do we stand? | TITLE |
|  | Kanadaşi et al., 2005 | Frequency of diastolic dysfunction in patients with sickle cell anaemia: a tissue Doppler imaging study | ABSTRACT |
|  | Karafint al., 2016 | NHLBI state of the science symposium in therapeutic apheresis: Knowledge gaps and research opportunities in the area of hematology-oncology | TITLE |
|  | Karakaşt et al., 2013 | Left ventricular dyssynchrony is an early manifestation of heart involvement in sickle cell anemia | Title |
|  | Kassabet al., 2007 | Transcranial Doppler: an introduction for primary care physicians | TITLE |
|  | Kassimet al., 2015 | Low forced expiratory volume is associated with earlier death in sickle cell anemia | Title |
|  | Kato et al., 2010 | Diastolic dysfunction in sickle cell | Abstract |
|  | Katzet al., 2018 | Comorbid obstructive sleep apnea and increased risk for sickle cell disease morbidity |  |
|  | Kanter et al., 2018 | Recent progress in gene therapy for severe sickle cell disease: Updated interim results from a phase 1 clinical study of lentiglobin gene therapy | ABSTRACT |
|  | Kanter et al., 2016 | Interim results from a phase 1/2 clinical study of lentiglobin gene therapy for severe sickle cell disease | TITLE |
|  | Kanter et al., 2022 | Biologic and Clinical Efficacy of LentiGlobin for Sickle Cell Disease | TITLE |
|  | Kapetanaki et al., 2019 | Free heme regulates placenta growth factor through NRF2-antioxidant response signaling | TITLE |
|  | Kapoor et al., 2020 | The Prevalence and Impact of Arrhythmias in Hospitalized Patients with Sickle Cell Disorders: A Large Database Analysis | TITLE |
|  | Kapoor et al., 2015 | Multiple neurological and systemic complications in a pregnant patient with sickle cell anemia | TITLE |
|  | Kapoor et al., 2005 | Altitude related disorders and their management | TITLE |
|  | Karacaoglu et al., 2016 | East Mediterranean region sickle cell disease mortality trial: retrospective multicenter cohort analysis of 735 patients | TITLE |
|  | Karafin et al., 2016 | Increased circulating fibrocytes are associated with higher reticulocyte percent in children with sickle cell anemia | ABSTRACT |
|  | Karafin et al., 2021 | Chronic Pain Does Not Impact Baseline Circulating Cytokine Levels in Adults with Sickle Cell Disease | ABSTRACT |
|  | Karakaş et al., 2012 | Assessment of atrial and ventricular mechanics in patients with sickle cell disease without overt pulmonary hypertension: A two-dimensional deformation imaging study | TITLE |
|  | Karayaylali et al., 2002 | Low blood pressure, decreased incidence of hypertension, and renal cardiac, and autonomic nervous system functions in patients with sickle cell syndromes | ABSTRACT |
|  | Karoor et al., 2021 | Evidence supporting a role for circulating macrophages in the regression of vascular remodeling following sub-chronic exposure to hemoglobin plus hypoxia | Title |
|  | Karimi et al., 2020 | Neglected pulmonary arterial hypertension in sickle cell anaemia during prenatal care | TITLE |
|  | Karkoska et al., 2020 | A pilot study to screen for poor academic performance in children with sickle cell disease in the outpatient setting | ABSTRACT |
|  | Karyofyllis et al., 2022 | Sickle cell disease related chronic thromboembolic pulmonary hypertension: challenging clinical scenario | ABSTRACT |
|  | Kasar et al., 2014 | Clinical significance of circulating blood and endothelial cell microparticles in sickle-cell disease | ABSTRACT |
|  | Kassim et al., 2013 | Sickle cell disease, vasculopathy, and therapeutics | TITLE |
|  | Kassim et al., 2023 | Reduced Intensity Haploidentical Bone Marrow Transplantation in Adults with Severe Sickle Cell Disease: BMT CTN 1507 | TITLE |
|  | Kasai et al., 2022 | Overview of current progress and challenges in diagnosis, and management of pediatric sickle cell disease in Democratic Republic of the Congo | TITLE |
|  | Kato et al., 2012 | Erratum: Cardiac rehabilitation participant with sickle cell trait and statin-related hepatotoxicity: A case report (Journal of Cardiopulmonary Rehabilitation and Prevention | TITLE |
|  | Kaur et al., 2021 | Myocardial injury and coronary microvascular disease in sickle cell disease | TITLE |
|  | Kato et al., 2008 | Novel small molecule therapeutics for sickle cell disease: nitric oxide, carbon monoxide, nitrite, and apolipoprotein A-I | TITLE |
|  | Kato et al., 2010 | Risk factors for echocardiography-determined cardiopulmonary abnormalities in sickle cell anemia in the walk-PHaSST study | TITLE |
|  | Kato et al., 2012 | TRV: A physiological biomarker in sickle cell disease | ABSTRACT |
|  | Kato et al., 2012 | Priapism in Sickle-Cell Disease: A Hematologist's Perspective | TITLE |
|  | Kato et al., 2018 | Exercise training: a prescription for sickle-cell disease? | TITLE |
|  | Kato et al., 2019 | Sickle cell vasculopathy: vascular phenotype on fire! | Title |
|  | Kato et al., 2008 | Evolution of novel small-molecule therapeutics targeting sickle cell vasculopathy | TITLE |
|  | Kato et al., 2009 | Mechanisms and clinical complications of hemolysis in sickle cell disease and thalassemia | TITLE |
|  | Kato et al., 2007 | Deconstructing sickle cell disease: reappraisal of the role of hemolysis in the development of clinical subphenotypes | TITLE |
|  | Kato et al., 2009 | Vasculopathy in sickle cell disease: Biology, pathophysiology, genetics, translational medicine, and new research directions | TITLE |
|  | Kato et al., 2006 | Cerebrovascular disease associated with sickle cell pulmonary hypertension | TITLE |
|  | Kato et al., 2005 | Levels of soluble endothelium-derived adhesion molecules in patients with sickle cell disease are associated with pulmonary hypertension, organ dysfunction, and mortality | ABSTRACT |
|  | Kato et al., 2007 | Pulmonary hypertension in sickle cell disease: relevance to children | ABSTRACT |
|  | Kato et al., 2018 | Sickle cell disease | ABSTRACT |
|  | Kato et al., 2017 | Intravascular hemolysis and the pathophysiology of sickle cell disease | TITLE |
|  | Kato et al., 2010 | Pleiotropic effects of intravascular haemolysis on vascular homeostasis | TITLE |
|  | Kato et al., 2009 | Endogenous nitric oxide synthase inhibitors in sickle cell disease: Abnormal levels and correlations with pulmonary hypertension, desaturation, haemolysis, organ dysfunction and death | TITLE |
|  | Kato et al., 2006 | Arginine Metabolite Profiling in Sickle Cell Disease: Abnormal Levels and Correlations with Pulmonary Hypertension, Desaturation, Hemolysis and Organ Dysfunction | TITLE |
|  | Katusicet al., 2009 | Vascular protection by tetrahydrobiopterin: progress and therapeutic prospects | TITLE |
|  | Kaur et al., 2020 | Cardiomyopathy in Sickle Cell Disease | Title |
|  | Kaur et al., 2000 | Prostacyclin for secondary pulmonary hypertension | TITLE |
|  | Kaur et al., 2022 | Causes of Mortality in Sickle Cell Disease Patients: A Longitudinal Review of Deceased Sickle Cell Patients | TITLE |
|  | Kaushik et al., 2012 | Chronically transfused pediatric sickle cell patients are protected from cardiac iron overload | TITLE |
|  | Kawaharaet al., 2019 | Successful Treatment of Acute Chest Syndrome with Manual Exchange Transfusion in a Patient with Sickle Beta(+)-thalassemia | TITLE |
|  | Kengne Fotsing al., 2022 | Relation between haptoglobin polymorphism and oxidative stress status, lipid profile, and cardiovascular risk in sickle cell anemia patients | TITLE |
|  | Khamseekaew et al., 2016 | Effects of Iron Overload on Cardiac Calcium Regulation: Translational Insights Into Mechanisms and Management of a Global Epidemic | TITLE |
|  | Khanet al., 2023 | Potential inflammatory targets in the integrative health care of patients with sickle cell disease | TITLE |
|  | Kaur et al., 2023 | Crizanlizumab in sickle cell disease | TITLE |
|  | Kaur et al., 2013 | An overview on sickle cell disease profile | TITLE |
|  | Kavanagh et al., 2022 | Sickle Cell Disease: A Review | TITLE |
|  | Kayle, et al., 2019 | Transition to adult care in sickle cell disease: A longitudinal study of clinical characteristics and disease severity | TITLE |
|  | Keesari et al., 2023 | Comparing Racial Disparities of Cardiovascular Manifestations in Sickle Cell Trait | TITLE |
|  | Kelaidiet al., 2018 | PlGF and sFlt-1 levels in patients with non-transfusion-dependent thalassemia: Correlations with markers of iron burden and endothelial dysfunction | TITLE |
|  | Kelly et al., 2016 | Erythrocytapheresis for chronic transfusion therapy in sickle cell disease: survey of current practices and review of the literature | TITLE |
|  | Kevil et al., 2011 | Inorganic nitrite therapy: Historical perspective and future directions | TITLE |
|  | Kendel et al., 2021 | Stroke Following Acute Chest Syndrome in a Child With Sickle Cell Disease: A Possible Novel Mechanism | TITLE |
|  | Kerchberger et al., 2020 | The Role of Circulating Cell-Free Hemoglobin in Sepsis-Associated Acute Kidney Injury | TITLE |
|  | Kervan et al., 2011 | Implantable cardioverter defibrillator lead endocarditis causing diffuse right atrial abscess and pulmonary artery embolism | Title |
|  | Khadija Khay et al., 2018 | Heart failure associated to the hemoglobinopathy | TITLE |
|  | Khamees et al., 2021 | Manifestations of HbSE sickle cell disease: a systematic review | TITLE |
|  | Khan e al., 2022 | Sickle Cell Disease and Its Respiratory Complications | TITLE |
|  | Khan et al., 2005 | Detection of circulating endothelial cells and endothelial progenitor cells by flow cytometry | TITLE |
|  | Khemphet et al., 2022 | Prevalence and Association between Obesity and Iron Deficiency in Children | TITLE |
|  | Khin et al., 2019 | PULMONARY HYPERTENSION IN SICKLE CELL DISEASE: LOOKING BEYOND THE USUAL SUSPECT | TITLE |
|  | Khondakar et al., 2020 | EFFECT OF SICKLE CELL GENOTYPE ON COMPONENTS OF METABOLIC SYNDROME | TITLE |
|  | Khorshied et al., 2018 | Protein Z and Endothelin-1 genetic polymorphisms in pediatric Egyptian sickle cell disease patients | TITLE |
|  | Khafaja et al., 2023 | Multisystem inflammatory syndrome in children (MIS-C) and “Near MIS-C”: A continuum? | TITLE |
|  | Khamees et al., 2022 | Single-center experience of LVAD implantation in patients with sickle-cell trait: A retrospective analysis | TITLE |
|  | Khan et al., 2016 | Patent foramen ovale and stroke in childhood: A systematic review of the literature | TITLE |
|  | Khandeparkar et al., 2013 | Cardiac surgery in patients with sickle cell disease | TITLE |
|  | Khoury et al., 2011 | Pulmonary complications of sickle cell disease | TITLE |
|  | Khatamiet al., 2020 | Deceptology in cancer and vaccine sciences: Seeds of immune destruction-mini electric shocks in mitochondria: Neuroplasticity-electrobiology of response profiles and increased induced diseases in four generations - A hypothesis | ABSTRACT |
|  | Khurshid et al., 2002 | Sickle cell disease, extreme hyperbilirubinemia, and pericardial tamponade: Case report and review of the literature | TITLE |
|  | Kim et al., 2022 | Liver Transplant in Hemoglobin SC Disease and Autoimmune Hepatitis: A Case Report | TITLE |
|  | Khurmi al., 2017 | Perioperative considerations for patients with sickle cell disease: a narrative review | TITLE |
|  | Kimt et al., 2009 | Dynamic cerebral autoregulation in homozygous Sickle cell disease | TITLE |
|  | Kincaidet al., 2008 | Transcranial Doppler ultrasonography: a diagnostic tool of increasing utility | TITLE |
|  | Kingt al., 2015 | Successful matched sibling donor marrow transplantation following reduced intensity conditioning in children with hemoglobinopathies | TITLE |
|  | Kiani et al., 2020 | Prenatal genetic diagnosis: Fetal therapy as a possible solution to a positive test | TITLE |
|  | Kielstein et al., 2005 | Arginine metabolism, pulmonary hypertension, and sickle cell disease | TITLE |
|  | Kingué et al., 2000 | Diastolic function of the left ventricle in a North-African patient with homozygous sickle-cell anemia | TITLE |
|  | Klingset al., 2006 | Abnormal pulmonary function in adults with sickle cell anemia | ABSTRACT |
|  | Knight-Maddenet al., 2013 | Mortality, asthma, smoking and acute chest syndrome in young adults with sickle cell disease | TITLE |
|  | Kiley et al., 2012 | American Journal of Physiology - Lung Cellular and Molecular Physiology | TITLE |
|  | Kimet al., 2022 | Diagnosis and treatment of transfusion-related iron overload | TITLE |
|  | Kim et al., 2014 | Red cell exchange: special focus on sickle cell disease | TITLE |
|  | Kimmig et al., 2016 | Review of the Association between Splenectomy and Chronic Thromboembolic Pulmonary Hypertension | TITLE |
|  | Kimyon et al., 2022 | Is the treatment of inflammatory arthritis different in sickle cell disease? | TITLE |
|  | King et al., 2014 | Management of right heart failure in the critically ill | TITLE |
|  | Kinger et al., 2021 | Abdominal Manifestations of Sickle Cell Disease | TITLE |
|  | Kingue et al., 2000 | A study of left ventricular diastolic function in a homozygous Black African population with sickle-cell anemia | TITLE |
|  | Kirkham et al., 2004 | Arterial ischaemic stroke in children. Review of the literature and strategies for future stroke studies | ABSTRACT |
|  | Kirkham et al., 2003 | Stroke and cerebrovascular disease in childhood | TITLE |
|  | Kitenge et al., 2018 | Diagnostic tools and follow-up of sickle-cell anemia in Central Africa | Title |
|  | Kirkham et al., 2007 | Therapy insight: Stroke risk and its management in patients with sickle cell disease | TITLE |
|  | Kladny et al., 2009 | Western Pennsylvania sickle cell network: Accomplishments through integration, education, and product development | TITLE |
|  | Klings et al., 2010 | Alterations in HLA-DR expression in peripheral blood mononuclear cells are associated with an elevated tricuspid regurgitant jet velocity and pulmonary hypertension of sickle cell disease | TITLE |
|  | Klings et al., 2008 | Pulmonary hypertension of sickle cell disease: More than just another lung disease | TITLE |
|  | Klings et al., 2008 | Pulmonary arterial hypertension and left-sided heart disease in sickle cell disease: clinical characteristics and association with soluble adhesion molecule expression | ABSTRACT |
|  | Klings et al., 2001 | Increased f2 isoprostanes in the acute chest syndrome of sickle cell disease as a marker of oxidative stress | ABSTRACT |
|  | Klings et al., 2004 | Pulmonary hypertension as a risk factor for death in patients with sickle cell disease | TITLE |
|  | Klings et al., 2014 | Response to "Efficacy and safety of sildenafil for the treatment of severe pulmonary hypertension in patients with hemoglobinopathies: Results from a long-term follow up " Haematologica 2014;99(2): e17-18 | ABSTRACT |
|  | Klingset al., 2011 | Altered inflammatory gene expression occurs in peripheral blood mononuclear cells of sickle cell disease patients with an elevated tricuspid regurgitant jet velocity and pulmonary hypertension | TITLE |
|  | Klings et al., 2009 | Genetic polymorphisms in NEDD4L are associated with pulmonary hypertension of sickle cell anemia | TITLE |
|  | Klingset al., 2015 | Management of patients with sickle cell disease | ABSTRACT |
|  | Klings et al., 2014 | An official american thoracic society clinical practice guideline: Diagnosis, risk stratification, and management of pulmonary hypertension of sickle cell disease | TITLE |
|  | Klings et al., 2012 | Making it personal: Using genomics to predict pulmonary hypertension in sickle cell disease | TITLE |
|  | Klings et al., 2014 | Pulmonary hypertension of sickle cell disease beyond classification constraints | TITLE |
|  | Klings et al., 2006 | Identification of oxidative post-translational modifications on plasma albumin in patients with pulmonary hypertension of sickle cell anemia | ABSTRACT |
|  | Klings et al., 2005 | Abnormal Pulmonary Function in Adults with Sickle Cell Disease: Association of Decreased DLCO with Systemic Disease | ABSTRACT |
|  | Knight et al., 2020 | Dyspnea in a splenectomized 12-year-old male with hereditary spherocytosis | TITLE |
|  | Knorr et al., 2014 | A massive intestinal vaso-occlusive crisis or girdle syndrome in a 6-year-old boy observed as a first manifestation of sickle cell disease | TITLE |
|  | Koduri et al., 2006 | Acute splenic sequestration crisis in adults with hemoglobin S-C disease: A report of nine cases | TITLE |
|  | Kogler et al., 2019 | Endocarditis caused by Stenotrophomonas maltophilia—A rare presentation of an emerging opportunistic pathogen | TITLE |
|  | Koch et al., 2015 | Intensive management of high-utilizing adults with sickle cell disease lowers admissions | TITLE |
|  | Koehl et al., 2022 | High risk and low prevalence diseases: Acute chest syndrome in sickle cell disease | TITLE |
|  | Koh et al., 2013 | Liver stiffness increases acutely during sickle cell vaso-occlusive crisis | TITLE |
|  | Koh et al., 2013 | Managing haematological disorders during pregnancy | TITLE |
|  | Kohanzadeh et al., 2023 | Association and risk factors of pediatric pulmonary hypertension with obstructive sleep apnea: A national study utilizing the Kids' Inpatient Database (KID) | TITLE |
|  | Kone et al., 2006 | S-nitrosylation: Targets, controls and outcomes | TITLE |
|  | Koneti et al., 2014 | Catheter interventions for double steal from isolation of the subclavian artery associated with patent arterial duct | TITLE |
|  | Konstantinos et al., 2009 | Phosphodiesterase-5 inhibitors: Future perspectives | TITLE |
|  | Kort et al., 2022 | Diffuse cystic lung disease in sickle cell anaemia: A series of 22 cases and a case-control study | TITLE |
|  | Kossorotoff et al., 2014 | Cerebral vasculopathy in pediatric sickle-cell anemia | Title |
|  | Kotiah et al., 2009 | Investigational drugs in sickle cell anemia | TITLE |
|  | Koyuncu et al., 2021 | Cardiac Chamber Quantification by Echocardiography in Adults With Sickle Cell Disease: Need Attention to Eccentric Hypertrophy | TITLE |
|  | Kredietet al., 2010 | To resuscitate or not? Discuss timely with all chronically ill patients | TITLE |
|  | Krishnamurti et al., 2007 | Life threatening parvovirus B19 and herpes simplex virus associated acute myocardial dysfunction in a child with homozygous sickle cell disease | TITLE |
|  | Krishnamurtiet al., 2019 | Bone marrow transplantation for adolescents and young adults with sickle cell disease: Results of a prospective multicenter pilot study | TITLE |
|  | Koutsouka et al., 2011 | A study of chronic hydroxyurea administration in sickle cell patients | TITLE |
|  | Kogler et al., 2019 | Endocarditis caused by Stenotrophomonas maltophilia-A rare presentation of an emerging opportunistic pathogen | TITLE |
|  | Koloet al., 2013 | Cardiac autonomic dysfunction in sickle cell anaemia and its correlation with QT parameters | TITLE |
|  | Kontoghiorgheset al., 2013 | A record number of fatalities in many categories of patients treated with deferasirox: loopholes in regulatory and marketing procedures undermine patient safety and misguide public funds? | TITLE |
|  | Kotwal et al., 2022 | Spirometric Changes After Initiation of Hydroxyurea in Children With Sickle Cell Anemia | TITLE |
|  | Koumbourlis et al., 2014 | Lung function in sickle cell disease | TITLE |
|  | Konieczka et al., 2015 | Cilioretinal artery occlusion in a young patient with flammer syndrome and increased retinal venous pressure | ABSTRACT |
|  | Koren et al., 2010 | Non-transferrin bound labile plasma iron and iron overload in sickle cell disease: A comparative study between sickle cell disease and β thalassemic patients | TITLE |
|  | Kossorotoff et al., 2018 | CD34+ Hematopoietic Stem Cell Count Is Predictive of Vascular Event Occurrence in Children with Sickle Cell Disease | TITLE |
|  | Kozman et al., 2021 | Pediatric Right Ventricular Cardiac Steatosis following Immunosuppressive Treatment | TITLE |
|  | Krajewski et al., 2008 | The proverbial chicken or the egg? Dissection of the role of cell-free hemoglobin versus reactive oxygen species in sickle cell pathophysiology | TITLE |
|  | Krishnamurti et al., 2007 | Hematopoietic cell transplantation: A curative option for sickle cell disease | Title |
|  | Krishnamurti et al., 2007 | Hematopoietic cell transplantation for sickle cell disease: State of the art | TITLE |
|  | Krishnamurti et al., 2021 | Hematopoietic cell transplantation for sickle cell dis ease: Updates and future directions | TITLE |
|  | Krishnamurti et al., 2008 | Hematopoietic Cell Transplantation for Hemoglobinopathies | TITLE |
|  | Kumar et al., 2018 | Applicability of and potential barriers preventing allogeneic stem cell transplant in sickle cell patients treated outside a sickle cell program | TITLE |
|  | Kumar et al., 2020 | Renal papillary necrosis as the first presenting clinical feature in a sickle beta and thalassemic child | TITLE |
|  | Kuriyan et al., 2006 | Commentary on TRALI in leukemia | TITLE |
|  | Lagas et al., 2011 | Lethal morphine intoxication in a patient with a sickle cell crisis and renal impairment: Case report and a review of the literature | TITLE |
|  | Kruzel-Davila et al., 2017 | APOL1 Nephropathy: A Population Genetics and Evolutionary Medicine Detective Story | TITLE |
|  | Krishnan et al., 2010 | Increased levels of the inflammatory biomarker C-reactive protein at baseline are associated with childhood sickle cell vasocclusive crises | TITLE |
|  | Kuanr et al., 2023 | AN OBSERVATIONAL STUDY TO DETERMINE THE PREVALENCE OF PULMONARY HYPERTENSION IN CHILDREN WITH SICKLE CELL DISEASE | TITLE |
|  | Kuentz et al., 2011 | Myeloablative BMT in young adults with sickle-cell disease: the French experience | ABSTRACT |
|  | Kuma et al., 2018 | Prevalence of relative systemic hypertension in adults with sickle cell disease in Ghana | TITLE |
|  | Kubong et al., 2020 | Relationship between Higher Atherogenic Index of Plasma and Oxidative Stress of a Group of Patients Living with Sickle Cell Anemia in Cameroon | TITLE |
|  | Kucukal et al., 2018 | Red Blood Cell Adhesion to Heme-Activated Endothelial Cells Reflects Clinical Phenotype in Sickle Cell Disease | TITLE |
|  | Kuoet al., 2013 | The use of extracorporeal membrane oxygenation in pediatric patients with sickle cell disease | TITLE |
|  | Kupfermant al., 2022 | Blood pressure in children with sickle cell disease is higher than in the general pediatric population | TITLE |
|  | Kurowski et al., 2000 | The preparticipation athletic evaluation | TITLE |
|  | Kuo et al., 2011 | The effect of comprehensive care on maternal and fetal outcomes in sickle cell disease pregnancies | TITLE |
|  | Kupesiz et al., 2012 | The effect of hemolysis on plasma oxidation and nitration in patients with sickle cell disease | TITLE |
|  | Kurnick et al., 2023 | Reversible severe pulmonary hypertension and right heart failure with cardiogenic shock due to scurvy: a case report | TITLE |
|  | Kvamet al., 2021 | Life-Threatening Acute Chest Syndrome in a Patient With Sickle Cell Disease After Switching From Hydroxyurea Therapy to Partial Exchange Transfusions: A Case Report | ABSTRACT |
|  | Kwanashie et al., 2009 | Pharmacological studies on siculine syrup. II: effects on smooth, skeletal and cardiovascular muscle preparations | TITLE |
|  | Lakkakula et al., 2019 | Association between MTHFR 677C>T polymorphism and vascular complications in sickle cell disease: A meta-analysis | TITLE |
|  | Lam et al., 2011 | The lymphocyte potassium channels Kv1.3 and KCa3.1 as targets for immunosuppression | ABSTRACT |
|  | Lama et al., 2011 | Screening and diagnosis of pulmonary hypertension in sickle cell disease patients | TITLE |
|  | Landburg et al., 2008 | Elevated Circulating Stromal Derived Factor-1 Levels in Sickle Cell Disease | ABSTRACT |
|  | Landburg et al., 2010 | Plasma asymmetric dimethylarginine concentrations in sickle cell disease are related to the hemolytic phenotype | TITLE |
|  | Landburg et al., 2009 | Elevated circulating stromal-derived factor-1 levels in sickle cell disease | ABSTRACT |
|  | Landburg et al., 2008 | Association of asymmetric dimethylarginine with sickle cell disease-related pulmonary hypertension | Abstract |
|  | Laires et al., 2004 | Role of cellular magnesium in health and human disease | ABSTRACT |
|  | Larissi et al., 2019 | The Growth Differentiation Factor-15 (GDF-15) levels are increased in patients with compound heterozygous sickle cell and beta-thalassemia (HbS/β(thal)), correlate with markers of hemolysis, iron burden, coagulation, endothelial dysfunction and pulmonary hypertension | TITLE |
|  | Law et al., 2007 | Staged single-ventricle palliation in an infant with hemoglobin SC disease | Title |
|  | Lazopoulos et al., 2008 | Mitral valve replacement and tricuspid valve repair in a patient with sickle cell disease | TITLE |
|  | Lang et al., 2023 | Clinical Outcomes of Acute Chest Syndrome in Patients with Sickle Cell Disease and Pulmonary Hypertension | TITLE |
|  | Lal et al., 2023 | Pragmatic anesthetic approach for extracranial to intracranial bypass surgery in a patient with moyamoya disease and sickle cell disease: a case report | TITLE |
|  | Lamers et al., 2006 | Evaluation of Left Ventricular Systolic Function in Pediatric Sickle Cell Anemia Patients Using the End-Systolic Wall Stress-Velocity of Circumferential Fiber Shortening Relationship | TITLE |
|  | Langer et al., 2013 | Right-To-Left Shunts (Extra-Cardiac Arterial-Venous Malformations) Are Highly Common In Adults With Sickle Cell Disease | TITLE |
|  | Lanzkron et al., 2013 | Mortality rates and age at death from sickle cell disease: U.S., 1979-2005 | TITLE |
|  | Lanzkron et al., 2003 | Platelet count and prevalence of acute chest syndrome, pulmonary hypertension and thrombotic stroke in sickle cell disease | TITLE |
|  | Lappas et al., 2017 | Successful exchange transfusions in a patient with sickle-beta thalassemia having recurrent episodes of liver failure from intrahepatic cholestasis | ABSTRACT |
|  | Larkin et al., 2005 | Elevated Plasma Arginase Levels in Hemoglobinopathies | TITLE |
|  | Latronica et al., 2012 | Tricuspid regurgitant jet velocity (TRV), biomarkers of hemolysis, and impact of oxygen therapy in children with sickle cell disease (SCD) and vaso-occlusive pain episodes (VOE) | TITLE |
|  | Lau et al., 2015 | Early detection of pulmonary arterial hypertension | ABSTRACT |
|  | Lauridsen et al., 2022 | Doing more with less: Patient blood management meets sickle cell disease management | ABSTRACT |
|  | Laurin et al., 2014 | Hydroxyurea is associated with lower prevalence of albuminuria in adults with sickle cell disease | TITLE |
|  | Leão et al., 2005 | Anesthesia in obstetric patient with sickle cell anemia and thalassemic trait after plasmapheresis. Case report | TITLE |
|  | Le Guennec et al., 2019 | Reversible splenial lesion syndrome during venoarterial extracorporeal membrane oxygenation |  |
|  | Lawrence et al., 2016 | Sickle Cell Disease and Stroke: Diagnosis and Management | Title |
|  | Lebensburger et al., 2015 | Systematic review of interventional sickle cell trials registered in ClinicalTrials.gov | TITLE |
|  | Lee et al., 2021 | COVID-19 and Hemoglobinopathies: A Systematic Review of Clinical Presentations, Investigations, and Outcomes | TITLE |
|  | Lee et al., 2011 | Anemia in Pregnancy | TITLE |
|  | Lee et al., 2022 | A Suspected Case of Multisystem Inflammatory Disease in Children Following COVID-19 Vaccination: A Case Report and Systematic Literature Review | TITLE |
|  | Lerman et al., 2011 | Coagulation and hematology in children: An update | TITLE |
|  | Lee et al., 2020 | Editorial to “Sickle-Cell Disease-associated Arrhythmias and In-hospital Outcomes: Insights from the National Inpatient Sample” | TITLE |
|  | Lee et al., 2007 | Pulmonary hypertension in sickle cell disease | TITLE |
|  | Lee et al., 2008 | Pulmonary Hypertension Is Not Associated with An Increased Risk of Death in Children with Sickle-Cell Disease Followed for a Mean of 3 Years | ABSTRACT |
|  | Lee et al., 2009 | Doppler-defined pulmonary hypertension and the risk of death in children with sickle cell disease followed for a mean of three years | ABSTRACT |
|  | Lehmann et al., 2008 | Sickle cell disease, pulmonary hypertension, and sarcoidosis | ABSTRACT |
|  | Le Guernet al., 2021 | Indirect causes of maternal deaths (except stroke, cardiovascular diseases and infections) in France 2013-2015 | ABSTRACT |
|  | Lee et al., 2012 | Adults with sickle cell disease: an interdisciplinary approach to home care and self-care management with a case study | TITLE |
|  | L'Esperance et al., 2016 | Nocturnal haemoglobin oxygen desaturation in urban and rural East African paediatric cohorts with and without sickle cell anaemia: a cross-sectional study | TITLE |
|  | Liem et al., 2020 | The Sickle Cell Pro-Inflammatory Response to Interval Testing Study (SPRINTS) in children and young adults with sickle cell anemia - Study design and methodological strategies | TITLE |
|  | Lemogoum et al., 2008 | Vascular aspects of sickle cell disease | TITLE |
|  | Lemon et al., 2022 | Acute venous thromboembolism after initiation of voxelotor for treatment of sickle cell disease | Title |
|  | Lemonne et al., 2014 | Association between relative systemic hypertension and otologic disorders in patients with sickle cell-hemoglobin C disorder | TITLE |
|  | Lemos et al., 2023 | Biventricular systolic and diastolic function in adolescents with sickle cell anemia and sickle cell traits – Systematic review | TITLE |
|  | Leonardo et al., 2022 | Individual Red Blood Cell Fetal Hemoglobin Concentrations Determined By Imaging Flow Cytometry and the Number of F Cells Are Very Good Predictors of Clinical Response to Increased Level of HbF and Are Associated with Hemolysis, Tricuspid Regurgitant Jet Velocity, and Sickle Retinopathy in Adult Sickle Cell Disease Patients | TITLE |
|  | Leveziel et al., 2011 | Clinical and laboratory factors associated with the severity of proliferative sickle cell retinopathy in patients with sickle cell hemoglobin C (SC) and homozygous sickle cell (SS) disease | TITLE |
|  | Levine et al., 2006 | Diagnosis and management of pulmonary arterial hypertension: Implications for respiratory care | ABSTRACT |
|  | Leveque et al., 2023 | A 24-Year-Old Woman With Cough, Arthralgia, and Skin Ulcerations | ABSTRACT |
|  | Levy et al., 2014 | Clinical use of the activated partial thromboplastin time and prothrombin time for screening: A review of the literature and current guidelines for testing | TITLE |
|  | Liem et al., 2018 | Balancing exercise risk and benefits: Lessons learned from sickle cell trait and sickle cell anemia | TITLE |
|  | Li et al., 2015 | MicroRNA 648 Targets ET-1 mRNA and Is Cotranscriptionally Regulated with <i>MICAL3</i> by PAX5 | TITLE |
|  | Li et al., 2014 | Peroxisome proliferator-activated receptor-α-mediated transcription of miR-199a2 attenuates endothelin-1 Expression via Hypoxia-inducible Factor-1α | TITLE |
|  | Li et al., 2022 | Pyrazole-containing pharmaceuticals: target, pharmacological activity, and their SAR studies | TITLE |
|  | Li et al., 2023 | Macitentan treatment of portopulmonary hypertension with hepatopulmonary syndrome: a case report and literature review | TITLE |
|  | Li et al., 2014 | Emerging microengineered tools for functional analysis and phenotyping of blood cells | Title |
|  | Liem et al., 2019 | American Society of Hematology 2019 guidelines for sickle cell disease: Cardiopulmonary and kidney disease | TITLE |
|  | Liem et al., 2020 | The Sickle Cell Pro-Inflammatory Response to Interval Testing Study (SPRINTS) in children and young adults with sickle cell anemia – Study design and methodological strategies | TITLE |
|  | Liem et al., 2015 | The acute phase inflammatory response to maximal exercise testing in children and young adults with sickle cell anaemia | TITLE |
|  | Liem et al., 2009 | Tricuspid regurgitant jet velocity elevation and its relationship to lung function in pediatric sickle cell disease | TITLE |
|  | Liem et al., 2009 | Functional capacity in children and young adults with sickle cell disease undergoing evaluation for cardiopulmonary disease | ABSTRACT |
|  | Liem et al., 2009 | Reliability of tricuspid regurgitant jet velocity measurements in children and young adults with sickle cell disease | ABSTRACT |
|  | Liem et al., 2006 | Tricuspid Regurgitant Jet Velocity Is Significantly Associated with Hemolysis in the Evaluation of Pulmonary Hypertension in Children and Young Adults with Sickle Cell Disease | ABSTRACT |
|  | Liem et al., 2010 | Reproducibility of tricuspid regurgitant jet velocity measurements in children and young adults with sickle cell disease undergoing screening for pulmonary hypertension | ABSTRACT |
|  | Liem et al., 2007 | Tricuspid regurgitant jet velocity is associated with hemolysis in children and young adults with sickle cell disease evaluated for pulmonary hypertension | ABSTRACT |
|  | Liem et al., 2007 | Corrected QT Interval Is Related to Markers of Hemolysis and Tricuspid Regurgitant Jet Velocity in a Young Cohort with Sickle Cell Disease | ABSTRACT |
|  | Liemet al., 2017 | Association among sickle cell trait, fitness, and cardiovascular risk factors in CARDIA | TITLE |
|  | Liemet al., 2015 | Reduced fitness and abnormal cardiopulmonary responses to maximal exercise testing in children and young adults with sickle cell anemia | TITLE |
|  | Limerick et al., 2022 | Pentostatin Plus Cyclophosphamide Decrease Graft Rejection in Nonmyeloablative Haploidentical Hematopoietic Cell Transplantation for Sickle Cell Disease | TITLE |
|  | Lima et al., 2008 | Enalapril therapy and cardiac remodelling in sickle cell disease patients | TITLE |
|  | Lin et al., 2005 | Pulmonary hypertension in patients with hemoglobinopathies: Could a mechanism for dysfunction provide an avenue for novel therapeutics? | TITLE |
|  | Lin et al., 2005 | Hemolytic anemia-associated pulmonary hypertension in sickle cell disease | ABSTRACT |
|  | Linder et al., 2021 | Red cell transfusion and alloimmunization in sickle cell disease | ABSTRACT |
|  | Lindstedt et al., 2008 | Prolonged QTc in Sickle Cell Disease: A Potential Risk Factor for Early Death? | TITLE |
|  | Linguraru et al., 2008 | Pulmonary artery segmentation and quantification in sickle cell associated pulmonary hypertension | TITLE |
|  | Linguraru et al., 2008 | CT and image processing non-invasive indicators of sickle cell secondary pulmonary hypertension | ABSTRACT |
|  | Lionnet et al., 2009 | Guidelines for management of adult sickle cell disease | TITLE |
|  | Lionnet et al., 2008 | Efficacy of the endothelin receptor blocker bosentan for refractory sickle cell leg ulcers | TITLE |
|  | Linguraru al., 2014 | Computed tomography correlates with cardiopulmonary hemodynamics in pulmonary hypertension in adults with sickle cell disease | TITLE |
|  | Lionnet et al., 2012 | Hemoglobin sickle cell disease complications: a clinical study of 179 cases | Title |
|  | Lionnet et al., 2017 | Sickle cell disease | ABSTRACT |
|  | Lisk et al., 2023 | Moderate hypoxia induces metabolic divergence in circulating monocytes and tissue resident macrophages from Berkeley sickle cell anemia mice | TITLE |
|  | Lisk et al., 2023 | Metabolic and Proteomic Divergence Is Present in Circulating Monocytes and Tissue-Resident Macrophages from Berkeley Sickle Cell Anemia and β-Thalassemia Mice | Title |
|  | Lisk et al., 2012 | Hemoglobin, endothelial cells, and TLRS. Common connection? | TITLE |
|  | Lisk et al., 2011 | Evidence for cell free hemoglobin induction of HIF-1A through a toll like receptor- NF-KB signaling pathway | TITLE |
|  | Lishmanov et al., 2017 | A Woman in Her 20s With Cardiopulmonary Failure | TITLE |
|  | Lorch et al., 2011 | An elevated estimated pulmonary arterial systolic pressure, whenever measured, is associated with excess mortality in adults with sickle cell disease | TITLE |
|  | Little et al., 2009 | Hematologic, biochemical, and cardiopulmonary effects of l-arginine supplementation or phosphodiesterase 5 inhibition in patients with sickle cell disease who are on hydroxyurea therapy | ABSTRACT |
|  | Littleet al., 2010 | The plasma microparticle proteome | TITLE |
|  | Liu et al., 2017 | Antiglycation, radical scavenging, and semicarbazide-sensitive amine oxidase inhibitory activities of acetohydroxamic acid in vitro | TITLE |
|  | Little et al., 2006 | Combination erythropoietin-hydroxyurea therapy in sickle cell disease: Experience from the National Institutes of Health and a literature review | TITLE |
|  | Little et al., 2010 | Iron overload in sickle cell disease | TITLE |
|  | Little et al., 2014 | Nocturnal hypoxemia (not sleep apnea) may drive reticulocytosis in sickle cell disease | TITLE |
|  | Liu et al., 2022 | Plasma Free Hemoglobin Is Associated with LDH, AST, Total Bilirubin, and the Hemolytic Component in Patients with Sickle Cell Anemia | TITLE |
|  | Liu et al., 2015 | Plasma FGF23 Is a Biomarker for Left Ventricular Hypertrophy and Mortality in Adults with Sickle Cell Anemia Chronic Kidney Disease | TITLE |
|  | Liu et al., 2021 | Inhaled pulmonary vasodilators: a narrative review | TITLE |
|  | Livesay et al., 2012 | Acute chest syndrome of sickle cell disease | TITLE |
|  | Lizarralde-Iragorri et al., 2020 | Sickle cell disease: A paradigm for venous thrombosis pathophysiology | TITLE |
|  | Loar et al., 2021 | Assessing the atria in pediatric sickle cell disease: Beyond the dilation | TITLE |
|  | Lobo et al., 2011 | Pulmonary hypertension in sickle cell disease: Study of 137 patients randomly selected from a public hematology hospital in Rio de Janeiro, Brazil | TITLE |
|  | Lode et al., 2007 | Pulmonary hypertension in a case of Hb-Mainz hemolytic anemia | ABSTRACT |
|  | Loganathan et al., 2023 | Non-coding RNAs in human health and disease: potential function as biomarkers and therapeutic targets | ABSTRACT |
|  | Lopes et al., 2022 | Prevalence of Cardiovascular Complications in Individuals with Sickle Cell Anemia and Other Hemoglobinopathies: A Systematic Review | TITLE |
|  | Lopes et al., 2015 | Key endothelial cell angiogenic mechanisms are stimulated by the circulating milieu in sickle cell disease and attenuated by hydroxyurea | Title |
|  | Lopez et al., 2000 | Bench to bedside: Nitric oxide in emergency medicine | TITLE |
|  | López-Galán et al., 2023 | Autonomic and Vascular Responses during Reactive Hyperemia in Healthy Individuals and Patients with Sickle Cell Anemia | TITLE |
|  | Loupy et al., 2008 | Fat emboli unleashed: An exceptional etiology of encephalitis in sickle cell disease | TITLE |
|  | Lu et al., 2020 | Myocarditis: A heart on fire | TITLE |
|  | Lundeen et al., 2019 | Acute Hypoxemia and Coma in a Patient With Hemoglobin SC Disease | TITLE |
|  | Lucas et al., 2023 | Correctly Establishing and Interpreting Oxygenation Status in Sickle Cell Disease | TITLE |
|  | Loscalzo et al., 2011 | Systems biology and personalized medicine: A network approach to human disease | TITLE |
|  | Lottenberg et al., 2009 | Sickle Cell Disease | TITLE |
|  | Lunt et al., 2014 | Pulmonary function, CT and echocardiographic abnormalities in sickle cell disease | TITLE |
|  | Lovett et al., 2014 | Sickle cell disease in the emergency department | TITLE |
|  | Lovett et al., 2017 | Sickle Cell Disease in the Emergency Department | Title |
|  | Lu et al., 2016 | Clinical Features and Morbidities of Hb H Disease in Taiwan | TITLE |
|  | Lu et al., 2022 | Incidence and prevalence of 121 rare diseases in China: Current status and challenges: 2022 revision | TITLE |
|  | Lucas et al., 2023 | Apohemoglobin-Haptoglobin Complex Improves Cardiac and Pulmonary Function in Homozygous Townes (HbSS Townes) Mice | TITLE |
|  | Luiking et al., 2012 | Arginine de novo and nitric oxide production in disease states | TITLE |
|  | Lukowski et al., 2022 | Recent developments in cGMP research: From mechanisms to medicines and back | TITLE |
|  | Luong et al., 2013 | Beneficial role of vitamin D3 in the prevention of certain respiratory diseases | TITLE |
|  | Lüscher et al., 2020 | Sex and gender and cardiovascular medicine: Impact in diabetes, acute coronary syndromes, and heart failure | TITLE |
|  | Lynch et al., 2004 | Cerebrovascular disorders in children | TITLE |
|  | Maarman et al., 2020 | Pulmonary hypertension in majority countries: opportunities amidst challenges | TITLE |
|  | Macdougall et al., 2019 | The effect of splenectomy on tricuspid jet velocity in pediatric patients with sickle cell disease | ABSTRACT |
|  | Machado et al., 2022 | The Association of Heterozygous β-Thalassemia and β-Spectrin Mutations May Result in a Phenotype of Severe Anemia | ABSTRACT |
|  | Machado et al., 2005 | Natriuretic Peptide Levels Correlate with Pulmonary Pressures and Prospective Mortality in SCD: Use of This Biomarker To Identify Prevalence and Mortality of Pulmonary Hypertension in the MSH Cohort | ABSTRACT |
|  | Macé et al., 2014 | Fetal anemia as a signal of congenital syphilis | ABSTRACT |
|  | Maddali et al., 2016 | Staged Single Ventricle Palliation and Homozygous Sickle Cell Disease | TITLE |
|  | Maddali et al., 2020 | Acquired Proximal Left Atrial Hypertension | TITLE |
|  | Maddali et al., 2006 | Management of sickle cell disease during CABG surgery: A case report | TITLE |
|  | Machado et al., 2007 | Sickle cell anemia-associated pulmonary arterial hypertension | TITLE |
|  | Machado et al., 2007 | Nitric oxide-based therapies in sickle cell disease: The evidence continues to mount | Abstract |
|  | Machado et al., 2008 | Amino acids and the erythrocyte under stress? | TITLE |
|  | Machadoet al., 2006 | N-terminal pro-brain natriuretic peptide levels and risk of death in sickle cell disease | TITLE |
|  | Machado et al., 2010 | Evaluation of sildenafil therapy for patients with sickle cell disease and increased tricuspid regurgitant velocity: Preliminary results of the Walk-PHaSST trial | TITLE |
|  | Machado et al., 2009 | Safety and efficacy of sildenafil therapy for Doppler-defined pulmonary hypertension in patients with sickle cell disease: Preliminary results of the walk-PHaSST clinical trial | TITLE |
|  | Machogu et al., 2021 | Lung clearance index in children with sickle cell disease | TITLE |
|  | Machogu et al., 2018 | How I treat hypoxia in adults with hemoglobinopathies and hemolytic disorders | TITLE |
|  | Maddali et al., 2006 | Cardiopulmonary bypass without preoperative exchange transfusion in sicklers | TITLE |
|  | Maddali et al., 2006 | Management of sickle cell disease during CABG surgery--a case report | TITLE |
|  | Mah et al., 2020 | Tilt-table Echocardiography Unmasks Early Diastolic Dysfunction in Patients With Hemoglobinopathies | TITLE |
|  | Madias et al., 2008 | Sickle cell anemia, left ventricular hypertrophy and T-wave alternans | ABSTRACT |
|  | Magrin et al., 2022 | Long-term outcomes of lentiviral gene therapy for the β-hemoglobinopathies: the HGB-205 trial | ABSTRACT |
|  | Majjiga et al., 2010 | Thrombotic thrombocytopenic purpura and multiorgan system failure in a child with sickle cell-hemoglobin c disease | TITLE |
|  | Malinowski et al., 2021 | Adverse outcome of acute splenic sequestration crisis in pregnancy | TITLE |
|  | Marie et al., 2003 | Diffuse osteocondensation | TITLE |
|  | Maitre et al., 2000 | Acute chest syndrome in adults with sickle cell disease | TITLE |
|  | Manga et al., 2008 | Adult purulent meningitis caused by Streptococcus pneumoniae in Dakar, Senegal | TITLE |
|  | Machado et al., 2011 | Hospitalization for pain in patients with sickle cell disease treated with sildenafil for elevated TRV and low exercise capacity | Title |
|  | Machado et al., 2013 | Pulmonary hypertension associated with chronic hemolytic anemia and other blood disorders | TITLE |
|  | Marchant et al., 2001 | Coronary artery bypass graft surgery in a patient with haemoglobin SC disease | ABSTRACT |
|  | Marchese et al., 2022 | Physical Impairment and Function in Children and Adolescents With Sickle Cell Disease: A Systematic Review | TITLE |
|  | Marinho et al., 2019 | Respiratory resistance and reactance in adults with sickle cell anemia: Part 2-Fractional-order modeling and a clinical decision support system for the diagnosis of respiratory disorders | TITLE |
|  | Marlin et al., 2008 | Cardiorespiratory responses during three repeated incremental exercise tests in sickle cell trait carriers | TITLE |
|  | Maron et al., 2014 | Assessment of the 12-lead ECG as a screening test for detection of cardiovascular disease in healthy general populations of young people (12-25 years of age) a scientific statement from the american heart association and the American College of cardiology | TITLE |
|  | Marouf et al., 2011 | Blood transfusion in sickle cell disease | TITLE |
|  | Marinho et al., 2017 | Respiratory resistance and reactance in adults with sickle cell anemia: Correlation with functional exercise capacity and diagnostic use | TITLE |
|  | Machado et al., 2005 | Chronic sickle cell lung disease: New insights into the diagnosis, pathogenesis and treatment of pulmonary hypertension | TITLE |
|  | Machadoet al., 2010 | Pulmonary hypertension in hemolytic disorders: pulmonary vascular disease: the global perspective | TITLE |
|  | Machado et al., 2011 | NT-pro brain natriuretic peptide levels and the risk of death in the cooperative study of sickle cell disease | TITLE |
|  | Machado et al., 2007 | Severity of pulmonary hypertension during vaso-occlusive pain crisis and exercise in patients with sickle cell disease | ABSTRACT |
|  | Machado et al., 2005 | Sildenafil therapy in patients with sickle cell disease and pulmonary hypertension | ABSTRACT |
|  | Madigan et al., 2006 | Pathophysiology and therapy for haemoglobinopathies; Part I: Sickle cell disease | ABSTRACT |
|  | Madu et al., 2022 | Phenotypic characterisation and associations of leg ulcers in adult sickle cell patients | TITLE |
|  | Mahesh et al., 2016 | Pulmonary endarterectomy is effective and safe in patients with haemoglobinopathies and abnormal red blood cells: the Papworth experience | TITLE |
|  | Mahesri et al., 2022 | PATIENTS WITH SEVERE SICKLE CELL DISEASE ON STANDARD OF CARE TREATMENT ARE VERY UNLIKELY TO BECOME VOC FREE FOR ONE YEAR: A COHORT STUDY OF MEDICAID ENROLLEES | TITLE |
|  | Mahut et al., 2004 | Measurement of nitrogen oxide in expired air of children | ABSTRACT |
|  | Mahut et al., 2004 | La mesure du monoxyde d’azote dans l’air expiré chez l’enfant | TITLE |
|  | Maier-Redelsperger et al., 2010 | Strong association between a new marker of hemolysis and glomerulopathy in sickle cell anemia | TITLE |
|  | Main et al., 2020 | Association of maternal comorbidity with severe maternal morbidity: A cohort study of california mothers delivering between 1997 and 2014 | TITLE |
|  | Maiocco et al., 2023 | Co-Diagnosis of Sickle Cell and Chronic Myelogenous Leukemia: Exploratory Analysis from a Nationally Representative Database | TITLE |
|  | Maitra et al., 2017 | Risk factors for mortality in adult patients with sickle cell disease: A meta-analysis of studies in North America and Europe | TITLE |
|  | Maitre et al., 2000 | Acute chest syndrome in adults with sickle cell disease: Therapeutic approach, outcome, and results of BAL in a monocentric series of 107 episodes | TITLE |
|  | Maitre et al., 2015 | Inhaled nitric oxide for acute chest syndrome in adult sickle cell patients: a randomized controlled study | Abstract |
|  | Maître et al., 2011 | Pulmonary complications in adult sickle cell disease | TITLE |
|  | Maître et al., 2011 | Complications pulmonaires des syndromes drépanocytaires majeurs chez l’adulte | ABSTRACT |
|  | Maitre et al., 2018 | Leukemoid Reaction in Infant Pertussis: Is There a Place for Hydroxyurea? A Case Report | TITLE |
|  | Majumdar et al., 2013 | The adolescent with sickle cell disease | TITLE |
|  | Majzner et al., 2016 | Pulmonary function after hematopoietic stem cell transplantation is significantly better in pediatric recipients following reduced toxicity compared with myeloablative conditioning | TITLE |
|  | Makani et al., 2013 | Sickle Cell Disease: New Opportunities and Challenges in Africa | Title |
|  | Malanchini et al., 2019 | Heart in sickle cell-beta thalassemia: Echocardiograpic appearance of an extremely rare disease | TITLE |
|  | Houeijeh et al., 2018 | French national survey on infective endocarditis and the Melody™ valve in percutaneous pulmonary valve implantation | Title |
|  | Malhan et al., 2022 | RWD90 Chart Review Study for Patients With Sickle Cell Disease With Central Nervous System Crises in Jazan – Saudi Arabia | TITLE |
|  | Malowany et al., 2012 | Pathology of sickle cell disease | TITLE |
|  | Mancano et al., 2018 | ISMP Adverse Drug Reactions: Minocycline-Induced Drug Fever Without Skin Rash Lupus Erythematosus–Like Eruption Induced by Hydroxyurea Cardiotoxicity and Fever Induced by Clozapine Denosumab-Induced Hepatotoxicity Severe Cardiotoxicity Induced by Bevacizumab | TITLE |
|  | Manci et al., 2003 | Causes of death in sickle cell disease: An autopsy study | TITLE |
|  | Manci et al., 2014 | High protein diet attenuates histopathologic organ damage and vascular leakage in transgenic murine model of sickle cell anemia | TITLE |
|  | Mandal et al., 2016 | Pulmonary Function Tests in Sickle Cell Disease: Correspondence | TITLE |
|  | Manganas et al., 2023 | THE USE OF LUSPATERCEPT IN PATIENTS WITH B -THALASSAEMIA AND MULTIPLE SYSTEMIC COMPLICATIONS INCREASES HEMOGLOBIN LEVEL, DECREASES TRANSFUSION BURDEN, AND IS UNAFFECTED BY COMPLICATIONS. SINGLE THALASSEMIA AND SICKLE CELL DEPARTEMENT'S EXPERIENCE | ABSTRACT |
|  | Mansueto et al., 2021 | Nailfold capillaroscopy: A comprehensive review on common findings and clinical usefulness in non-rheumatic disease | TITLE |
|  | Manwar et al., 2023 | A study of Electrocardigraphic and Echocardiographic changes in sickle cell anemia patients – Observational Study | TITLE |
|  | Manzur et al., 2015 | Cardiovascular manifestations of sickle cell disease | TITLE |
|  | Manzuret al., 2015 | Manifestaciones cardiovasculares de anemia de células falciformes |  |
|  | Maphumulo et al., 2022 | Role of Circulating Microparticles in Type 2 Diabetes Mellitus: Implications for Pathological Clotting | ABSTRACT |
|  | Maric et al., 2021 | Citrulline, biomarker of enterocyte functional mass and dietary supplement. Metabolism, transport, and current evidence for clinical use | TITLE |
|  | Mardan et al., 2021 | THE INCIDENCE OF PULMONARY HYPERTENSION AMONG PEDIATRIC THALASSEMIA PATIENTS IN BABYLON HEREDITARY BLOOD DISEASE CENTER IN BABYLON GOVERNORATE, IRAQ | TITLE |
|  | Marmaro et al., 2023 | Here Today, Gone Tomorrow: A Case of Transient Pulmonary Hypertension in Sickle Cell Crisis | Abstract |
|  | Maron et al., 2018 | Redefining pulmonary hypertension | TITLE |
|  | Maron et al., 2016 | Pulmonary vascular and ventricular dysfunction in the susceptible patient (2015 Grover Conference series) | TITLE |
|  | Marongiu et al., 2019 | Pulmonary Thrombosis: A Clinical Pathological Entity Distinct from Pulmonary Embolism? | Title |
|  | Maron et al., 2015 | Historical Perspectives on Sudden Deaths in Young Athletes With Evolution over 35 Years | TITLE |
|  | Maron et al., 2015 | Eligibility and Disqualification Recommendations for Competitive Athletes With Cardiovascular Abnormalities: Task Force 14: Sickle Cell Trait: A Scientific Statement From the American Heart Association and American College of Cardiology | TITLE |
|  | Martins Wde et al., 2012 | Cardiovascular autonomic dysfunction in sickle cell anemia | TITLE |
|  | Masese et al., 2021 | Sex-based differences in the manifestations and complications of sickle cell disease: Report from the Sickle Cell Disease Implementation Consortium | TITLE |
|  | Marouf et al., 2016 | Methodological contributions about systolic pulmonary artery pressure | TITLE |
|  | Marques et al., 2014 | Successful pulmonary thromboendarterectomy in a patient with sickle cell disease treated with a single preoperative red blood cell exchange | TITLE |
|  | Marra et al., 2023 | Challenges in Pulmonary Hypertension | TITLE |
|  | Marshall et al., 2022 | Elevated tricuspid regurgitation velocity is associated with increased adverse haematologic events during pregnancy in women with sickle cell disease | TITLE |
|  | Martí-Carvajal et al., 2013 | Antibiotics for treating acute chest syndrome in people with sickle cell disease | Abstract |
|  | Martin et al., 2015 | Clinical correlates of sleep-disordered breathing and nocturnal hypoxemia in sickle cell disease | TITLE |
|  | Masoumi et al., 2008 | Developments in the management of autosomal dominant polycystic kidney disease | TITLE |
|  | Maule et al., 2023 | Hidden Comorbidities in Asthma: A Perspective for a Personalized Approach | TITLE |
|  | Maunoury et al., 2003 | Impairment of myocardial perfusion in children with sickle cell disease | TITLE |
|  | McCavit et al., 2011 | Increase in invasive streptococcus pneumoniae infections in children with sickle cell disease since pneumococcal conjugate vaccine licensure | TITLE |
|  | McCaw-Binns et al., 2018 | The evolving contribution of non-communicable diseases to maternal mortality in Jamaica, 1998-2015: a population-based study | TITLE |
|  | McClain et al., 2016 | Improved Guideline Adherence With Integrated Sickle Cell Disease and Asthma Care | TITLE |
|  | McCutcheon et al., 2018 | The McConnell Sign is Seen in Patients With Acute Chest Syndrome | TITLE |
|  | McLornan et al., 2013 | Principles of haematopoietic stem cell transplantation | TITLE |
|  | Matoba et al., 2014 | Hypopituitarism possibly due to lymphocytic hypophysitis in a patient with type 1 diabetes | Title |
|  | Martins et al., 2022 | Endothelial dysfunction biomarkers in sickle cell disease: is there a role for ADMA and PAI-1? | TITLE |
|  | Martins et al., 2010 | Quality of sleep in clinically stable patients with sickle cell anaemia (HbSS) | TITLE |
|  | Marwick, et al., 2016 | Imaging and the Valley of Death Developing the Evidence Base for the Use of Imaging in Follow-Up | ABSTRACT |
|  | Maschmeyer et al., 2007 | Pulmonary hypertension in sickle cell disease - Epidemiology, pathogenesis, diagnosis and treatment | TITLE |
|  | Master et al., 2016 | Prevalence of disease related complications from in adult patients with sickle cell disease | ABSTRACT |
|  | Mathew et al., 2016 | Hematological disorders and pulmonary hypertension | ABSTRACT |
|  | McCarty et al., 2010 | Potential utility of full-spectrum antioxidant therapy, citrulline, and dietary nitrate in the management of sickle cell disease | ABSTRACT |
|  | McCavit et al., 2012 | Sickle cell disease | Title |
|  | McCormick et al., 2019 | Epidemiology and outcome of acute kidney injury in children with sickle cell disease | TITLE |
|  | McGann et al., 2017 | Clinical Features of β-Thalassemia and Sickle Cell Disease | TITLE |
|  | McLaughlinet al., 2007 | Sickle cell disease-associated pulmonary hypertension: A coat of many colors | ABSTRACT |
|  | McLaren et al., 2017 | Effect of Hydroxyurea Therapy on Pulmonary Function in Children with Sickle Cell Anemia | ABSTRACT |
|  | Mecabo et al., 2010 | Duffy-negative is associated with hemolytic phenotype of sickle cell anemia | ABSTRACT |
|  | Mechal et al., 2021 | Sickle cell disease and thalassemia: Significant causes of dilated cardiomyopathy, experience of Ibn Rochd university hospital-Casablanca | TITLE |
|  | Mehari et al., 2013 | Hemodynamic predictors of mortality in adults with sickle cell disease | TITLE |
|  | Mehari et al., 2012 | Mortality in adults with sickle cell disease and pulmonary hypertension | ABSTRACT |
|  | Mehari et al., 2019 | Abnormal ventilation-perfusion scan is associated with pulmonary hypertension in sickle cell adults | ABSTRACT |
|  | Mehari et al., 2016 | Chronic Pulmonary Complications of Sickle Cell Disease | TITLE |
|  | Mehari et al., 2016 | Review: Hemodynamic characteristics and outcomes of sickle cell disease associated pulmonary hypertension | ABSTRACT |
|  | Meerpohl et al., 2014 | Deferasirox for managing transfusional iron overload in people with sickle cell disease | TITLE |
|  | Mehta et al., 2013 | Daytime pulse oximetry measurements may not predict nocturnal desaturations in adult sickle cell patients | TITLE |
|  | Mendez et al., 2022 | Hemoglobin Concentration May Influence the Incidence of Postoperative Transient Neurological Events in Patients with Moyamoya after Extracranial-intracranial Arterial Bypass: A Retrospective Single Center Experience | TITLE |
|  | Mehrad et al., 2017 | Circulating fibrocytes as biomarkers of impaired lung function in adults with sickle cell disease | TITLE |
|  | Meloni et al., 2013 | Comparison of biventricular dimensions and function between pediatric sickle-cell disease and thalassemia major patients without cardiac iron | TITLE |
|  | Melvin et al., 2021 | A Case of Fat Embolism Syndrome with Cerebral Involvement in Sickle Cell Anemia | Title |
|  | Menet et al., 2018 | Subclinical Cardiac Dysfunction Is Associated With Extracardiac Organ Damages | TITLE |
|  | Menon et al., 2022 | Excess heme upregulates heme oxygenase 1 and promotes cardiac ferroptosis in mice with sickle cell disease | TITLE |
|  | Menon et al., 2007 | Subclinical sickle cell crisis during cardiopulmonary bypass: Timely detection and early management | TITLE |
|  | Menendez-Gonzalez et al., 2021 | Hematopoietic Stem Cell Mobilization: Current Collection Approaches, Stem Cell Heterogeneity, and a Proposed New Method for Stem Cell Transplant Conditioning | TITLE |
|  | Merckx et al., 2022 | Predictors of severe illness in children with multisystem inflammatory syndrome after SARS-CoV-2 infection: a multicentre cohort study | Title |
|  | Merlet et al., 2019 | Beneficial effects of endurance exercise training on skeletal muscle microvasculature in sickle cell disease patients | TITLE |
|  | Mittelstaedt et al., 2019 | Association of NT-proBNP with clinical outcomes in children with systemic inflammatory response syndrome | TITLE |
|  | Merlet et al., 2020 | Muscle structural, energetic and functional benefits of endurance exercise training in sickle cell disease | ABSTRACT |
|  | Messent et al., 2004 | Con: Exchange transfusion is not required for sickle cell trait patients undergoing cardiopulmonary bypass | TITLE |
|  | Mehta et al., 2011 | Cardiac iron overload causes clinically evident heart failure and arrhythmia in sickle cell anemia patients: Evidence from three cases | TITLE |
|  | Mehta et al., 2006 | Opportunities to improve outcomes in sickle cell disease | TITLE |
|  | Meier et al., 2012 | Sickle cell disease in children | TITLE |
|  | Mekontso Dessap et al., 2017 | Ten tips for managing critically ill patients with sickle cell disease | TITLE |
|  | Meloni et al., 2017 | Gender Differences in the Development of CMR Abnormalities and Cardiac Complications: A Multicentric Prospective Study in a Cohort of Sickle Cell Disease Patients | TITLE |
|  | Meloni et al., 2021 | Pancreatic iron is a marker of cardiovascular complications and in sickle cell disease | TITLE |
|  | Meloni et al., 2023 | Prognostic value of multiparametric cardiac magnetic resonance in sickle cell patients | ABSTRACT |
|  | Mendelsohn et al., 2012 | Association of plasma cell-free hemoglobin with physiological measures of nitric oxide responsiveness and estimated pulmonary artery systolic pressure: Requirement for rigorously optimized blood processing variables | TITLE |
|  | Mendonça et al., 2016 | Red cell DAMPs and inflammation | ABSTRACT |
|  | Mennes et al., 2012 | Sickle cell anaemia and the consequences on the anaesthetic management of cardiac surgery | TITLE |
|  | Messonnier et al., 2019 | Physiological Evaluation for Endurance Exercise Prescription in Sickle Cell Disease | TITLE |
|  | Metcalfe et al., 2015 | Validation of an obstetric comorbidity index in an external population | TITLE |
|  | Miller et al., 2001 | Sickle cell crisis in the adult: chest radiographic findings and comparison with pediatric sickle cell disease | TITLE |
|  | Mihalek et al., 2021 | Diseases Associated with Group 5 Pulmonary Hypertension | TITLE |
|  | Mocumbi et al., 2016 | Medical disease as a cause of maternal mortality: the pre-imminence of cardiovascular pathology | Title |
|  | Mohamed Jiffry et al., 2023 | Sickle Cell Anemia Associated With Increased In-Hospital Mortality in Post-Cardiac Arrest Patients | TITLE |
|  | Moll et al., 2020 | Severe Hypoxia and Compartment Syndrome in a Patient With Sickle Cell Trait After Redo Aortic Valve Replacement: A Case Report and Review of the Literature | TITLE |
|  | Monagel et al., 2022 | Pulmonary function in children and adolescents with sickle cell disease after nonmyeloablative hematopoietic cell transplantation | TITLE |
|  | Mondal et al., 2019 | Diagnostic value of spirometry vs impulse oscillometry: A comparative study in children with sickle cell disease | ABSTRACT |
|  | Monga et al., 2015 | Pulmonary extramedullary hematopoiesis involving the pulmonary artery | TITLE |
|  | Moutaouekkil et al., 2015 | Heart valve surgery in patients with homozygous sickle cell disease: A management strategy | TITLE |
|  | Moyssakis et al., 2005 | Systolic and diastolic function in middle aged patients with sickle β thalassaemia. An echocardiograpnic study | TITLE |
|  | Moodalbail et al., 2018 | Ambulatory hypertension in a pediatric cohort of sickle cell disease | TITLE |
|  | Moreb et al., 2021 | Fruits and Vegetables in the Management of Underlying Conditions for COVID-19 High-Risk Groups | TITLE |
|  | Morgan et al., 2019 | Bilateral internal mammary artery laceration after cardiac pulmonary resuscitation | TITLE |
|  | Morin et al., 2023 | Diffuse myocardial fibrosis occurs in young patients with sickle cell anemia despite early disease-modifying therapy | TITLE |
|  | Morris et al., 2017 | Arginine therapy shows promise for treatment of sickle cell disease clinical subphenotypes of hemolysis and arginine deficiency | TITLE |
|  | Morris et al., 2003 | Arginine therapy: A new treatment for pulmonary hypertension in sickle cell disease? | TITLE |
|  | Morris et al., 2011 | Risk factors and mortality associated with an elevated tricuspid regurgitant jet velocity measured by Doppler-echocardiography in thalassemia: A Thalassemia Clinical Research Network report | TITLE |
|  | Morris et al., 2005 | Hemolysis-associated pulmonary hypertension in thalassemia | Abstract |
|  | Morissens et al., 2019 | Added value of speckle tracking in the evaluation of cardiac function in patients with sickle cell disease | Title |
|  | Morais et al., 2016 | Giant Aneurysm of Left Coronary Artery Associated With Aneurysm of Right Sinus of Valsalva | TITLE |
|  | Morais et al., 2023 | Effects of a physiotherapeutic protocol in cardiorespiratory, muscle strength, aerobic capacity and quality of life after hematopoietic stem cell transplantation | TITLE |
|  | Metske et al., 2012 | Cooling the crisis: Therapeutic hypothermia after sickle cardiac arrest* | TITLE |
|  | Mian et al., 2013 | Characterization Of Pulmonary Compliance In Sickle Cell Patients Revealed Wide Variability | TITLE |
|  | Michael et al., 2017 | Identifying sickle cell anemia patients with pulmonary hypertension using 2d speckle tracking strain measurements | ABSTRACT |
|  | Michelet al., 2007 | Cardiac involvement during drepanocytosis (sickle cell anemia): Three types of cardiac involvement among the homozygotes | ABSTRACT |
|  | Michel et al., 2008 | Characteristics and Outcome of Connective Tissue Diseases in Patients with Sickle-Cell Disease: Report of 30 Cases | TITLE |
|  | Mihalek et al., 2021 | Encyclopedia of Respiratory Medicine, Second Edition | TITLE |
|  | Miller et al., 2012 | Pulmonary complications of sickle cell disease | TITLE |
|  | Milln et al., 2019 | Obstetric medicine in sub-Saharan Africa | ABSTRACT |
|  | Milner et al., 2020 | Preliminary safety results of defibrotide in scd patients with acute chest syndrome (ind 127812) | TITLE |
|  | Milner et al., 2020 | Preliminary Results of a Phase II Study to Determine the Safety of Defibrotide in Children and Adolescents with Sickle Cell Disease-Associated Acute Chest Syndrome | TITLE |
|  | Milton et al., 2011 | Clinical and genetic variability of red blood cell hemolysis in sickle cell anemia | TITLE |
|  | Minniti et al., 2015 | Phenotypic variations in sickle cell disease: An exploration of the role of the environment and skin microbiome on leg ulcers | ABSTRACT |
|  | Minniti et al., 2008 | Elevated Tricuspid Regurgitant Jet Velocity in Children and Adolescents with Sickle Cell Disease: Association with Hemolysis and Hemoglobin Oxygen Desaturation | ABSTRACT |
|  | Minniti et al., 2014 | Vasculopathy, inflammation, and blood flow in leg ulcers of patients with sickle cell anemia | ABSTRACT |
|  | Minniti et al., 2010 | Leg ulcers in sickle cell disease | ABSTRACT |
|  | Minniti et al., 2009 | Sickle cell leg ulcers are associated with hyperuricemia, hemolysis, pulmonary hypertension and death | TITLE |
|  | Minniti et al., 2016 | Critical Reviews: How we treat sickle cell patients with leg ulcers | ABSTRACT |
|  | Minniti et al., 2006 | Children's National Medical Center's transfusion protocol for sickle hemoglobinopathies | TITLE |
|  | Minniti et al., 2009 | Efficacy and safety of endothelin receptor antagonists for Pulmonary Hypertension in adult patients with sickle cell disease | TITLE |
|  | Minniti et al., 2009 | Endothelin receptor antagonists for pulmonary hypertension in adult patients with sickle cell disease | TITLE |
|  | Minniti et al., 2011 | Laboratory and echocardiography markers in sickle cell patients with leg ulcers | TITLE |
|  | Minniti et al., 2011 | Anti-haemolytic effect of senicapoc and decrease in NT-proBNP in adults with sickle cell anaemia | TITLE |
|  | Minniti et al., 2021 | Clinical predictors of poor outcomes in patients with sickle cell disease and COVID-19 infection | TITLE |
|  | Minter et al., 2001 | Pulmonary complications of sickle cell anemia: A need for increased recognition, treatment, and research | TITLE |
|  | Mirsaeidi et al., 2014 | Pneumococcal vaccine and patients with pulmonary diseases | Abstract |
|  | Misra et al., 2023 | Outcomes of Patients With Sickle Cell Disease and Trait After Congenital Heart Disease Surgery | TITLE |
|  | Misra et al., 2017 | A Phase Ib open label, randomized, safety study of SANGUINATE™ in patients with sickle cell anemia | TITLE |
|  | Misztal et al., 2011 | Pathophysiological consequences of hemolysis. Role of cell-free hemoglobin | TITLE |
|  | Mitka et al., 2007 | Researchers explore rare lung disorder | TITLE |
|  | Mneimneh et al., 2015 | Pulmonary vascular abnormalities in neurofibromatosis type 1 patients: An autopsy study of 8 cases | TITLE |
|  | MO et al., 2021 | Chronic opioid use in patients with sickle cell disease | TITLE |
|  | Mocumbi et al., 2015 | A global perspective on the epidemiology of pulmonary hypertension | TITLE |
|  | Moens et al., 2011 | Targeting endothelial and myocardial dysfunction with tetrahydrobiopterin | TITLE |
|  | Mogalapalli et al., 2019 | Cardiovascular abnormalities and determinant factors in children with sickle cell disease | TITLE |
|  | Mokhtar et al., 2010 | N-terminal natriuretic peptide and ventilation-perfusion lung scan in sickle cell disease and thalassemia patients with pulmonary hypertension | ABSTRACT |
|  | Mokhtar et al., 2017 | Tartrate-Resistant Acid Phosphatase 5b in Young Patients with Sickle Cell Disease and Trait Siblings: Relation to Vasculopathy and Bone Mineral Density | ABSTRACT |
|  | Morris et al., 2008 | Erythrocyte glutamine depletion, altered redox environment, and pulmonary hypertension in sickle cell disease | TITLE |
|  | Morton et al., 2015 | Toxicity of hydroxyurea in rats and dogs | ABSTRACT |
|  | Motta et al., 2019 | Curing Hemoglobinopathies: Challenges and Advances of Conventional and New Gene Therapy Approaches | TITLE |
|  | Moyssakis et al., 2005 | Systolic and diastolic function in middle aged patients with sickle beta thalassaemia. An echocardiographic study | TITLE |
|  | Mozos et al., 2015 | Mechanisms linking red blood cell disorders and cardiovascular diseases | Title |
|  | Mpalampa et al., 2012 | Foetal haemoglobin and disease severity in sickle cell anaemia patients in Kampala, Uganda | TITLE |
|  | Monagle et al., 2012 | Antithrombotic therapy in neonates and children: Antithrombotic therapy and prevention of thrombosis, 9th ed: American college of chest physicians evidence-based clinical practice guidelines | TITLE |
|  | Mondal et al., 2021 | Predictors of Diffusing Capacity in Children With Sickle Cell Disease: A Longitudinal Study | TITLE |
|  | Mondal et al., 2021 | Determinants of impaired gas exchange in children with sickle cell disease: A single-center longitudinal study | TITLE |
|  | Mondal et al., 2019 | The association of nocturnal hypoxia and an echocardiographic measure of pulmonary hypertension in children with sickle cell disease | TITLE |
|  | Moore et al., 2018 | Home based primary care for patients with sickle cell disease | TITLE |
|  | Moreau et al., 2012 | Vitamin K antagonists in children with heart disease: Height and VKORC1 genotype are the main determinants of the warfarin dose requirement | TITLE |
|  | Morissens et al., 2020 | Evaluation of cardiac function in patients with sickle cell disease with left ventricular global longitudinal strain | TITLE |
|  | Morris et al., 2006 | New strategies for the treatment of pulmonary hypertension in sickle cell disease: The rationale for arginine therapy | ABSTRACT |
|  | Morris et al., 2007 | Hemolysis-associated pulmonary hypertension in sickle cell disease: Global disruption of the arginine-nitric oxide pathway | ABSTRACT |
|  | Morris et al., 2008 | Mechanisms of vasculopathy in sickle cell disease and thalassemia | ABSTRACT |
|  | Morris et al., 2009 | Asthma management: reinventing the wheel in sickle cell disease | TITLE |
|  | Morris et al., 2009 | Hemolysis and arginine dysregulation | TITLE |
|  | Morris et al., 2011 | Vascular risk assessment in patients with sickle cell disease | TITLE |
|  | Morris et al., 2014 | Alterations of the arginine metabolome in sickle cell disease a growing rationale for arginine therapy | ABSTRACT |
|  | Morris et al., 2005 | Abnormal Pulmonary Function in Adults and Children with Sickle Cell Disease | TITLE |
|  | Morris et al., 2008 | Nitric oxide and arginine dysregulation: a novel pathway to pulmonary hypertension in hemolytic disorders | ABSTRACT |
|  | Morris et al., 2007 | High frequency of asthma, sepsis and acute chest syndrome in children with sickle cell disease and pulmonary hypertension | TITLE |
|  | Morris et al., 2017 | Acquired Amino Acid Deficiencies: A Focus on Arginine and Glutamine | TITLE |
|  | Morris et al., 2017 | The Defective Arginine-Nitric Oxide Pathway in Sickle Cell Disease | TITLE |
|  | Morris et al., 2010 | Low global arginine bioavailability: A novel mechanism in both primary & secondary pulmonary hypertension | TITLE |
|  | Morris et al., 2005 | Dysregulated arginine metabolism, hemolysis-associated pulmonary hypertension, and mortality in sickle cell disease | ABSTRACT |
|  | Morris et al., 2010 | Metabolic fate of oral glutamine supplementation within plasma and erythrocytes of patients with sickle cell disease: Preliminary pharmacokinetics results | ABSTRACT |
|  | Morris et al., 2022 | Implications for the metabolic fate of oral glutamine supplementation within plasma and erythrocytes of patients with sickle cell disease: A pharmacokinetics study | TITLE |
|  | Morris et al., 2011 | Oral glutamine supplementation improves global arginine bioavailability in patients with sickle cell disease and doppler-defined pulmonary hypertension: Preliminary pharmacokinetics results | TITLE |
|  | Morris et al., 2011 | Metabolic fate of oral glutamine supplementation within plasma and erythrocytes of patients with sickle cell disease and pulmonary hypertension: Preliminary pharmacokinetics results | Title |
|  | Morris et al., 2017 | Biomarker discovery: Novel insights into red blood cell metabolomics in patients with sickle cell disease at risk for early mortality | ABSTRACT |
|  | Morris et al., 2003 | Arginine therapy - A new treatment for pulmonary hypertension in sickle cell disease? | TITLE |
|  | Morris et al., 2006 | Clinical hemoglobinopathies: iron, lungs and new blood | TITLE |
|  | Morris et al., 2007 | Erythrocyte glutamine depletion and altered redox environment in pulmonary hypertension of sickle cell anemia | TITLE |
|  | Morris et al., 2012 | Arginases and arginine deficiency syndromes | ABSTRACT |
|  | Morrison et al., 2018 | Prevalence of left ventricular hypertrabeculation/noncompaction among children with sickle cell disease | TITLE |
|  | Morrone et al., 2018 | Association of silent infarcts in sickle cell anemia with decreased annexin A5 resistance | ABSTRACT |
|  | Mostafa et al., 2017 | Non-Sexual Implications of Phosphodiesterase Type 5 Inhibitors | TITLE |
|  | Motta et al., 2020 | COVID 19 and hemoglobinopathies: Update of the Italian experience | TITLE |
|  | Mousaidet al., 2022 | Hemoglobinopathies and dilated cardiomyopathy at the CHU Ibn Rochd-Casablanca | TITLE |
|  | Mueller et al., 2011 | Hydroxyurea for children with sickle cell disease: Are we starting too late? | TITLE |
|  | Mueller et al., 2004 | Prevalence of Respiratory Symptoms in Children with Sickle Cell Disease Compared to Children with Other Hematological Diseases | TITLE |
|  | Mueller et al., 2009 | Orbital compression syndrome in sickle cell crisis | ABSTRACT |
|  | Mukherjee et al., 2022 | Pulmonary Hypertension in Hemoglobinopathies: A Neglected Entity | TITLE |
|  | Murillo et al., 2008 | Large vegetations in Staphylococcus lugdunensis endocarditis | ABSTRACT |
|  | Murtuza et al., 2009 | The acute chest syndrome of sickle cell disease following aortic valve replacement | TITLE |
|  | Murugappan et al., 2019 | Case study: Fatal exertional rhabdomyolysis possibly related to drastic weight cutting | TITLE |
|  | Mpye et al., 2017 | Disease burden and the role of pharmacogenomics in African populations | TITLE |
|  | Muiru et al., 2022 | Black and White Adults With CKD Hospitalized With Acute Kidney Injury: Findings From the Chronic Renal Insufficiency Cohort (CRIC) Study | TITLE |
|  | Mukhopadhyay et al., 2000 | Role of hypoxia-inducible factor-1 in transcriptional activation of ceruloplasmin by iron deficiency | TITLE |
|  | Mullins et al., 2018 | Sickle Cell Hemoglobin C Disease Patient Undergoing Coronary Artery Bypass Grafting with Complete Exchange Blood Transfusion during Cardiopulmonary Bypass | TITLE |
|  | Mynarek et al., 2013 | Normalized transcranial Doppler velocities, stroke prevention and improved pulmonary function after stem cell transplantation in children with sickle cell anemia | TITLE |
|  | Kambala et al., 2023 | Schistosome Infection in the Humanized Mouse Model of Sickle Cell Disease | ABSTRACT |
|  | Mukhopadhyay et al., 2012 | Selection of a stroke risk model based on transcranial Doppler ultrasound velocity | TITLE |
|  | Mulumba et al., 2015 | Sickle cell disease among children in Africa: An integrative literature review and global recommendations | TITLE |
|  | Munshi et al., 2022 | IMPACT OF PULMONARY HYPERTENSION IN HOSPITALIZED PATIENTS WITH SICKLE CELL CRISES: ANALYSIS OF NATIONAL INPATIENT SAMPLES | TITLE |
|  | Murthy et al., 2013 | Acute, bilateral, concurrent central retinal artery occlusion in sickle cell disease after use of tadalafil (Cialis) | TITLE |
|  | Musa et al., 2016 | The global burden of pulmonary hypertension in sickle cell disease: a systematic review and meta-analysis | TITLE |
|  | Musa Et al., 2018 | Lower than expected elevated tricuspid regurgitant jet velocity in adults with sickle cell disease in Nigeria | TITLE |
|  | Steckelings et al., 2022 | The Angiotensin AT2 Receptor: Froma Binding Site to a Novel Therapeutic Target | ABSTRACT |
|  | Muschick et al., 2022 | Real-world data on voxelotor to treat patients with sickle cell disease | TITLE |
|  | Musso et al., 2012 | Vascular endothelial dysfunction, cytokines' overexpression, abnormal coagulation, and hyperomocysteinaemia may contribute to the development of pulmonary hypertension in sickle cell disease | TITLE |
|  | Muthyala et al., 2011 | Orphan/rare drug discovery through drug repositioning | ABSTRACT |
|  | Myers et al., 2002 | Cases from the Osler Medical Service at Johns Hopkins University | TITLE |
|  | Myers et al., 2010 | Clinical manifestations of high and low degrees of hemolysis in children with SCD | TITLE |
|  | Nader et al., 2021 | Vasculopathy in Sickle Cell Disease: From Red Blood Cell Sickling to Vascular Dysfunction | ABSTRACT |
|  | Nader et al., 2021 | Extracellular Vesicles in Sickle Cell Disease: Plasma Concentration, Blood Cell Types Origin Distribution and Biological Properties | ABSTRACT |
|  | Nader et al., 2020 | The Red Blood Cell-Inflammation Vicious Circle in Sickle Cell Disease | TITLE |
|  | Nader et al., 2022 | Nocturnal hypoxemia is associated with decreased erythrocyte deformability and enhanced hemolysis in sickle cell disease patients | TITLE |
|  | Naga et al., 2016 | Buccal Micronucleus Cytome Assay in Sickle Cell Disease | ABSTRACT |
|  | Nagalapuram et al., 2022 | Multi-organ dysfunction secondary to abrupt discontinuation of voxelotor in a patient with severe sickle cell disease | TITLE |
|  | National Collaborating Centre for Acute, Care, 2003 | National Institute for Health and Clinical Excellence: Guidance | TITLE |
|  | Naessens et al., 2018 | A proposed treatment algorithm for adults with Haemoglobin SC disease | TITLE |
|  | Nag et al., 2015 | Cardiac arrest following acute puerperal uterine | TITLE |
|  | Nemkov et al., 2022 | Plasma Levels of Acyl-Carnitines and Carboxylic Acids Correlate With Cardiovascular and Kidney Function in Subjects With Sickle Cell Trait | TITLE |
|  | Nagore Induráin et al., 2008 | The pharmacist, rare diseases and orphan medicines | ABSTRACT |
|  | Naik et al., 2013 | Venous thromboembolism in adults with sickle cell disease: a serious and under-recognized complication | TITLE |
|  | Naik et al., 2014 | Venous thromboembolism incidence in the Cooperative Study of Sickle Cell Disease | TITLE |
|  | Naik et al., 2013 | Sickle cell disease and venous thromboembolism: what the anticoagulation expert needs to know | TITLE |
|  | Nairet al., 2006 | Thrombotic Complications in Young Adults at High Altitude Areas: An Indian Experience | TITLE |
|  | Nambiar et al., 2012 | Multi-modality imaging of thoracic manifestations of sickle cell disease | TITLE |
|  | Namuyonga et al., 2021 | Pulmonary Hypertension in Children across Africa: The Silent Threat | TITLE |
|  | Naoman et al., 2010 | Echocardiographic findings in patients with sickle cell disease | TITLE |
|  | Nathan et al., 2011 | Guilt by association | ABSTRACT |
|  | Ndiaye et al., 2013 | Ischemic stroke in childhood: A Senegalese cohort | TITLE |
|  | Nasimuzzaman et al., 2019 | Role of the coagulation system in the pathogenesis of sickle cell disease | TITLE |
|  | Nathan et al., 2011 | Guilt by association | TITLE |
|  | Ndefo et al., 2008 | Pharmacological management of sickle cell disease | TITLE |
|  | Nebor et al., 2010 | Alpha-thalassemia is associated with a decreased occurrence and a delayed age-at-onset of albuminuria in sickle cell anemia patients | TITLE |
|  | Nekhai et al., 2022 | HIV-1 infection in sickle cell disease and sickle cell trait: role of iron and innate response | TITLE |
|  | Nekhaiet al., 2013 | Reduced sensitivity of the ferroportin Q248H mutant to physiological concentrations of hepcidin | TITLE |
|  | Nelsonet al., 2011 | Tobacco smoke exposure and pulmonary function in children with sickle cell disease | Title |
|  | Nemkov et al., 2017 | Metabolism of Citrate and Other Carboxylic Acids in Erythrocytes As a Function of Oxygen Saturation and Refrigerated Storage | ABSTRACT |
|  | Neumayr et al., 2006 | Left Ventricular Dysfunction in Chronically Transused Patients with Sickle Cell Anemia and Thalassemia | TITLE |
|  | Neumayr et al., 2019 | Sickle cell disease: current treatment and emerging therapies | ABSTRACT |
|  | Neumayr et al., 2009 | Prolonged QTc in patients with sickle cell disease (SCD) | TITLE |
|  | Neumann et al., 2007 | Children at altitude | TITLE |
|  | Nielsen et al., 2016 | Morbidity and mortality of sickle cell disease patients starting intermittent haemodialysis: a comparative cohort study with non- Sickle dialysis patients | TITLE |
|  | Nevilleet al., 2015 | Pharmacotherapy of sickle cell disease in children | TITLE |
|  | Newaskar et al., 2011 | Asthma in sickle cell disease | TITLE |
|  | Newmanet al., 2021 | Venous thromboembolism is associtated with increased disease severity and mortality risk in patients with sickle cell disease | TITLE |
|  | Newmanet al., 2005 | Pulmonary hypertension | ABSTRACT |
|  | Ngo et al., 2014 | Sickle cell disease | ABSTRACT |
|  | Nguwenezaet al., 2022 | Factors associated with blood pressure variation in sickle cell disease patients: a systematic review and meta-analyses | TITLE |
|  | Nguyen et al., 2021 | Using dual-energy computed tomographic imaging in children with sickle cell disease at risk for pulmonary hypertension to detect early signs of pulmonary vascular disease: A prospective observational pilot study | TITLE |
|  | Nguyen et al., 2016 | Elevated transpulmonary gradient and cardiac magnetic resonance-derived right ventricular remodeling predict poor outcomes in sickle cell disease | TITLE |
|  | Nguyen et al., 2017 | Sickle-cell and alpha-thalassemia traits resulting in non-atherosclerotic myocardial infarction: Beyond coincidence? | ABSTRACT |
|  | Ngwube et al., 2009 | Magnetic resonance t2∗ measurement of myocardial iron deposition in sickle cell disease: Risk factors and relationship with cardiac function | Title |
|  | Nicholson et al., 2011 | Coronary artery dilation in sickle cell disease | ABSTRACT |
|  | Niiharaet al., 2018 | A Phase 3 Trial of L-Glutamine in Sickle Cell Disease | TITLE |
|  | Nikparvaret al., 2016 | Intracardiac thrombosis in sickle cell disease | TITLE |
|  | Nimbkar et al., 2023 | A Tale of ‘AR’: A Rare All-round Review of Obstetric Autosomal Recessive Disorders | TITLE |
|  | Ninan et al., 2010 | The association between lipid levels and pulmonary hypertension in patients with sickle cell disease | TITLE |
|  | Niesor et al., 2015 | Potential Signal Transduction Regulation by HDL of the β2-Adrenergic Receptor Pathway. Implications in Selected Pathological Situations | ABSTRACT |
|  | Niesor et al., 2015 | Adenylyl Cyclase 9 Polymorphisms Reveal Potential Link to HDL Function and Cardiovascular Events in Multiple Pathologies: Potential Implications in Sickle Cell Disease | TITLE |
|  | Niesor et al., 2022 | Red Blood Cell Membrane Cholesterol May Be a Key Regulator of Sickle Cell Disease Microvascular Complications | TITLE |
|  | Ninkovich et al., 2010 | Sickle-cell anemia and pulmonary hypertension | TITLE |
|  | Niss et al., 2022 | Early initiation of disease-modifying therapy can impede or prevent diffuse myocardial fibrosis in sickle cell anemia | ABSTRACT |
|  | Nisset al., 2017 | Association between diffuse myocardial fibrosis and diastolic dysfunction in sickle cell anemia | TITLE |
|  | Nisset al., 2020 | Progression of albuminuria in patients with sickle cell anemia: a multicenter, longitudinal study | ABSTRACT |
|  | Niss et al., 2016 | Cardiomyopathy with Restrictive Physiology in Sickle Cell Disease | ABSTRACT |
|  | Niss et l., 2017 | Applications of cardiac magnetic resonance imaging in sickle cell disease | TITLE |
|  | Niazi et al., 2022 | Management of acute chest syndrome in patients with sickle cell disease: a systematic review of randomized clinical trials | TITLE |
|  | Niss et al., 2016 | Diffuse myocardial fibrosis is a common feature of sickle cell anemia that is associated with diastolic dysfunction and restrictive cardiac physiology | Title |
|  | Niu et al., 2009 | Angiogenic and inflammatory markers of cardiopulmonary changes in children and adolescents with sickle cell disease | Title |
|  | Njokuet al., 2023 | Associations of hemolysis and anemia with cardiopulmonary dysfunction in an adult sickle cell disease cohort | ABSTRACT |
|  | Nkya et al., 2017 | Fetal Hemoglobin is Associated with Peripheral Oxygen Saturation in Sickle Cell Disease in Tanzania | ABSTRACT |
|  | Noisetteet al., 2020 | Sickle Cell Disease Related Outcomes in Patients Evaluated for COVID-19 Infections in South Carolina | ABSTRACT |
|  | Nolanet al., 2018 | Hemolytic, vaso-occlusive and renal complications of SCD: Report from the central missouri cohort | TITLE |
|  | Nolanet al., 2006 | Sickle cell leg ulcers:: associations with haemolysis and SNPs in Klotho, TEK and genes of the TGF-β/BMP pathway | TITLE |
|  | Nobrega et al., 2022 | Subanesthetic ketamine: the way forward for pain management in sickle cell disease patients? | TITLE |
|  | Nouraie et al., 2020 | Serum albumin is independently associated with higher mortality in adult sickle cell patients: Results of three independent cohorts | TITLE |
|  | Noordstar et al., 2023 | Organized Sports Activities Are Safe for Children With Sickle Cell Disease: A Pilot Intervention Study | TITLE |
|  | Nouraie et al., 2011 | Predictors of osteoclast activity in patients with sickle cell disease | TITLE |
|  | Nouraie et al., 2015 | Blood transfusion and 30-day readmission rate in adult patients hospitalized with sickle cell disease crisis | TITLE |
|  | Novelli et al., 2014 | Elevated pulse pressure is associated with hemolysis, proteinuria and chronic kidney disease in sickle cell disease | TITLE |
|  | Nolan et al., 2005 | Leg Ulcers in Sickle Cell Anemia Are Associated with Laboratory Markers of Hemolysis and SNPs in KL and Genes of the TGF-β/BMP Pathway | ABSTRACT |
|  | Nomura rt al., 2010 | Maternal and perinatal outcomes in pregnancies complicated by sickle cell diseases | TITLE |
|  | Noronhaet al., 2020 | Cardiac causes of hypoxia in sickle cell disease | TITLE |
|  | Noronhaet al., 2016 | Management of sickle cell disease in children | ABSTRACT |
|  | Noubouossie et al., 2016 | Coagulation abnormalities of sickle cell disease: Relationship with clinical outcomes and the effect of disease modifying therapies | Abstract |
|  | Nouraie et al., 2010 | NT-proBNP as a marker of cardiopulmonary compromise and exercise limitation in adults with sickle cell anemia in the walk-phasst study | ABSTRACT |
|  | Nouraie et al., 2020 | Tricuspid regurgitation velocity and other biomarkers of mortality in children, adolescents and young adults with sickle cell disease in the United States: The PUSH study | ABSTRACT |
|  | Nouraieet al., 2013 | The relationship between the severity of hemolysis, clinical manifestations and risk of death in 415 patients with sickle cell anemia in the US and Europe | ABSTRACT |
|  | Nouraieet al., 2019 | Validation of a composite vascular high-risk profile for adult patients with sickle cell disease | ABSTRACT |
|  | Nouraie et al., 2008 | Association of Hemolysis with Clinical Manifestations of Sickle Cell Disease | ABSTRACT |
|  | Novelli et al., 2016 | Crises in Sickle Cell Disease | ABSTRACT |
|  | Novelliet al., 2012 | Plasma thrombospondin-1 is increased during acute sickle cell vaso-occlusive events and associated with acute chest syndrome, hydroxyurea therapy, and lower hemolytic rates | TITLE |
|  | Novelli et al., 2019 | Vascular TSP1-CD47 signaling promotes sickle cell-associated arterial vasculopathy and pulmonary hypertension in mice | ABSTRACT |
|  | Nunes et al., 2008 | Exercise-Induced Pulmonary Hypertension in Sickle Cell Anemia: a Study with Exercise Stress Echocardiography | TITLE |
|  | Nuret al., 2011 | Oxidative stress in sickle cell disease; pathophysiology and potential implications for disease management | ABSTRACT |
|  | Nur et al., 2010 | Plasma levels of advanced glycation end products are associated with haemolysis-related organ complications in sickle cell patients | ABSTRACT |
|  | Nugent et al., 2019 | Microvascular and systemic responses to novel PEGylated carboxyhaemoglobin-based oxygen carrier in a rat model of vaso-occlusive crisis | ABSTRACT |
|  | Nwankwo et al., 2017 | Calpain-1 regulates platelet function in a humanized mouse model of sickle cell disease | TITLE |
|  | Nyante et al., 2019 | Sex differences in physical activity among Ghanaian patients with sickle cell disease | TITLE |
|  | Ntaios et al., 2022 | Diagnostic Challenges and Uncertainties of Embolic Strokes of Undetermined Source in Young Adults | ABSTRACT |
|  | Ochocinski et al., 2020 | Life-Threatening Infectious Complications in Sickle Cell Disease: A Concise Narrative Review | ABSTRACT |
|  | Odhiambo et al., 2008 | Identification of Multiple Oxidative Post-Translational Modifications of Plasma Albumin in Patients with Pulmonary Hypertension of Sickle Cell Anemia | TITLE |
|  | Odhiamboet al., 2007 | Identification of oxidative post-translational modification of serum albumin in patients with idiopathic pulmonary arterial hypertension and pulmonary hypertension of sickle cell anemia | ABSTRACT |
|  | Odièvre et al., 2011 | Pathophysiological insights in sickle cell disease | TITLE |
|  | Oduor et al., 2017 | Severe cardiac iron toxicity in two adults with sickle cell disease | TITLE |
|  | Oduro-Boateyet al., 2017 | A ten-year review of chronic cor pulmonale secondary to respiratory diseases in Ghana | TITLE |
|  | Ofori-Acquah et al., 2020 | Sickle cell disease as a vascular disorder | ABSTRACT |
|  | Ogu et al., 2018 | Comorbidities in sickle cell disease: Adult providers needed! | TITLE |
|  | Ogah et al., 2008 | Spectrum of heart diseases in a new cardiac service in Nigeria: an echocardiographic study of 1441 subjects in Abeokuta | TITLE |
|  | Oguanobi et al., 2011 | Left ventricular dysfunction in sickle cell disease: the value of an electrocardiographic marker of increased risk of arrhythmia | TITLE |
|  | Oguanobi et al., 2015 | Echocardiographic findings in adult Nigerian sickle cell patients with cardiovascular autonomic dysfunction | ABSTRACT |
|  | Okwan-Duodu et al., 2018 | Impaired collateral vessel formation in sickle cell disease | ABSTRACT |
|  | Oguanobi et al., 2012 | Electrocardiographic findings in sickle cell cardiovascular autonomic neuropathy | TITLE |
|  | Oguanobi et al., 2012 | Clinical findings associated with cardiovascular autonomic dysfunction in adult sickle cell anaemia patients | TITLE |
|  | Oguanobi et al., 2010 | Electocardiographic findings in adult Nigerians with sickle cell anaemia | Title |
|  | Oguanobi et al., 2011 | P-wave dispersion: relationship to left ventricular function in sickle cell anaemia | ABSTRACT |
|  | Ogunsile et al., 2019 | Metabolic syndrome among adults living with sickle cell disease | TITLE |
|  | Ogunsile et al., 2022 | An evaluation of cardiopulmonary endurance and muscular strength in adults living with sickle cell disease | TITLE |
|  | Ogu et al., 2018 | Coagulation profile of sickle cell patients with leg ulcers | ABSTRACT |
|  | Oguanobi et al., 2011 | Left ventricular dysfunction in sickle cell disease: The value of an electrocardiographic marker of increased risk of arrhythmia | TITLE |
|  | Ogunlesi et al., 2011 | Sickle cell disease and the lung: Many questions still remain unanswered | TITLE |
|  | Oh et al., 2021 | Health Conditions and Psychotic Experiences: Cross-Sectional Findings From the American Life Panel | TITLE |
|  | Ojewunmi et al., 2019 | Current perspectives of sickle cell disease in Nigeria: changing the narratives | TITLE |
|  | Ojewunmi et al., 2018 | Haemoglobin oxygen saturation, leucocyte count and lactate dehydrogenase are predictors of elevated cerebral blood flow velocity in Nigerian children with sickle cell anaemia | ABSTRACT |
|  | Okam et al., 2012 | Novel approaches to the treatment of sickle cell disease: the potential of histone deacetylase inhibitors | ABSTRACT |
|  | Okorie et al., 2022 | Pediatric pulmonary year in review 2021: Sleep medicine | ABSTRACT |
|  | Okoye et al., 2022 | Prevalence of venous thromboembolism and its associations in a large racially homogenous population of sickle cell disease patients | ABSTRACT |
|  | Ohara et al., 2014 | Lung function and six-minute walk test performance in individuals with sickle cell disease | ABSTRACT |
|  | Okafor et al., 2012 | Kidney transplant in a 26-year-old nigerian patient with sickle cell nephropathy | ABSTRACT |
|  | Okar et al., 2022 | Dilemma in approach to stroke in sickle cell disease patient: A case report | TITLE |
|  | Okome-Nkoumou et al., 2000 | Typhoid and paratyphoid fever in adults in the Internal Medicine Department at Libreville (Gabon) | TITLE |
|  | Okpala et al., 2002 | Steady-state platelet count and complications of sickle cell disease | TITLE |
|  | Okpala et al., 2006 | Investigational agents for sickle cell disease | ABSTRACT |
|  | Okpala et al., 2005 | New therapies for sickle cell disease | TITLE |
|  | Okpere et al., 2012 | Prevalence of microalbuminuria among secondary school children | TITLE |
|  | Olaniran et al., 2019 | Cardiovascular Outcomes in African Americans with Sickle Cell Trait and Chronic Kidney Disease | TITLE |
|  | Oldham et al., 2021 | Computer Algorithm-Based Hydroxyurea Dosing Facilitates Titration to Maximum Tolerated Dose in Sickle Cell Anemia | ABSTRACT |
|  | Olson et al., 2016 | Tissue Doppler Imaging-derived Diastolic Function Assessment in Children With Sickle Cell Disease and Its Relation With Ferritin | TITLE |
|  | Olsen Ii et al., 2005 | Sickling in sports | ABSTRACT |
|  | Olatunji et al., 2018 | Neck circumference is independently associated with relative systemic hypertension in young adults with sickle cell anaemia | TITLE |
|  | Oliveira et al., 2021 | Cardiovascular risk assessment and management of patients undergoing hematopoietic cell transplantation | TITLE |
|  | Olnes et al., 2009 | Improvement in hemolysis and pulmonary arterial systolic pressure in adult patients with sickle cell disease during treatment with hydroxyurea | ABSTRACT |
|  | Onalo et al., 2022 | Arginine Therapy and Cardiopulmonary Hemodynamics in Hospitalized Children with Sickle Cell Anemia: A Prospective, Double-blinded, Randomized Placebo-controlled Clinical Trial | ABSTRACT |
|  | Onalo et al., 2020 | Cardiovascular changes in children with sickle cell crisis | ABSTRACT |
|  | Onalo et al., 2021 | Randomized control trial of oral arginine therapy for children with sickle cell anemia hospitalized for pain in Nigeria | ABSTRACT |
|  | Onalo et al., 2020 | Cardiovascular changes in children with sickle cell crisis | TITLE |
|  | Onget al., 2021 | Association of Acute Macular Neuroretinopathy or Paracentral Acute Middle Maculopathy with Sickle Cell Disease | Abstract |
|  | Oni et al., 2015 | Right ventricular systolic function assessment in sickle cell anaemia using echocardiography | Title |
|  | Oni et al., 2022 | Pulmonary hypertension and right ventricular function in the sickle cell populace | ABSTRACT |
|  | Oni et al., 2023 | Pulmonary Hypertension and Left Ventricular Geometric Types in Sickle Cell Anemia | ABSTRACT |
|  | Onimoe et al., 2020 | Sickle cell disease: A primary care update | ABSTRACT |
|  | Onyeamaet al., 2016 | Factors associated with continuous low-dose heparin infusion for central venous catheter patency in critically ill children worldwide | TITLE |
|  | Ondze-Kafata et al., 2014 | Echo-cardiographic aspects in sickle cell disease Guadeloupe | TITLE |
|  | O'Neill et al., 2016 | Routine preoperative tests for elective surgery: Summary of updated NICE guidance | ABSTRACT |
|  | Oyono-Enguéllé et al., 2000 | Cardiorespiratory and metabolic responses to exercise in HbSC sickle cell patients | TITLE |
|  | Onyekwere et al., 2008 | Pulmonary hypertension in children and adolescents with sickle cell disease | ABSTRACT |
|  | Onyekwere et al., 2004 | Prevalence of Pulmonary Hypertension (PHTN) in Sickle Cell Disease (SCD) Adolescents with Pulmonary Complications | ABSTRACT |
|  | Ortega-Laureano et al., 2018 | Surgical lung biopsy in children after hematopoietic cell transplantation | ABSTRACT |
|  | Osarogiagbon et al., 2009 | Effect of sickle cell disease type, age and gender on the prevalence of chronic organ damage in adults with sickle cell disease | TITLE |
|  | Osunkwo et al., 2019 | Impact of sickle cell disease from patients' and physicians' perspectives: Results from the international sickle cell world assessment survey (SWAY) | ABSTRACT |
|  | Onadeko et al., 2006 | Technetium 99m-diethylene triamine penta-acetic acid aerosol clearance in the evaluation of pulmonary involvement in sickle cell disease | TITLE |
|  | Ondze-Kafata et al., 2014 | Echocardiographic aspects of sickle cell disease in Guadeloupe | TITLE |
|  | Ong et al., 2013 | Respiratory muscle force and lung volume changes in a population of children with sickle cell disease | ABSTRACT |
|  | Oteng-Ntim et al., 2008 | Sickle cell disease in pregnancy | TITLE |
|  | Ovodov et al., 2000 | Nitric oxide: Clinical applications | TITLE |
|  | Owusu et al., 2015 | The interaction between sickle cell disease and HIV infection: a systematic review | Title |
|  | Oyedeji et al., 2022 | Characterizing Frailty in Adults with Sickle Cell Disease Using Frailty Phenotype | TITLE |
|  | Oyesanya et al., 2022 | A retrospective comparative study of the effect of automated exchange transfusion versus other treatment modalities on vaso-occlusive crisis admission rates in patients with sickle cell disease | TITLE |
|  | Oni et al., 2020 | Impact of relative systemic hypertension on the heart in sickle cell anaemia | TITLE |
|  | Oni et al., 2022 | Left ventricular geometry and electrocardiographic criteria in assessing left ventricular hypertrophy in sickle cell anemia patients | TITLE |
|  | Ouédraogo et al., 2017 | Impact of sickle cell trait on arterial stiffness in African subjects | ABSTRACT |
|  | Overturf et al., 2000 | American Academy of Pediatrics. Committee on Infectious Diseases. Technical report: prevention of pneumococcal infections, including the use of pneumococcal conjugate and polysaccharide vaccines and antibiotic prophylaxis | TITLE |
|  | Oyedeji et al., 2020 | Geriatric assessment for older adults with sickle cell disease: protocol for a prospective cohort pilot study | TITLE |
|  | Oyedeji et al., 2022 | The Sickle Cell Disease Functional Assessment (SCD-FA) tool: a feasibility pilot study | TITLE |
|  | Oyedeji et al., 2021 | Optimizing management of sickle cell disease in patients undergoing surgery | TITLE |
|  | Ozbek et al., 2007 | Airway hyperreactivity detected by methacholine challenge in children with sickle cell disease | TITLE |
|  | Ozaki et al., 2022 | Association between RNF213 c.14576G>A Variant (rs112735431) and Peripheral Pulmonary Artery Stenosis in Moyamoya Disease | TITLE |
|  | Ozçimen et al., 2014 | Tumor necrosis factor-superfamily 15 gene expression in patients with sickle cell disease | TITLE |
|  | Ozdogu et al., 2018 | Organ damage mitigation with the Baskent Sickle Cell Medical Care Development Program (BASCARE) | TITLE |
|  | Ozoh et al., 2019 | Pulmonary dysfunction among adolescents and adults with sickle cell disease in Nigeria: Implications for monitoring | TITLE |
|  | Ozturk et al., 2013 | Lipoprotein Subfraction Profile and HDL-Associated Enzymes in Sickle Cell Disease Patients | ABSTRACT |
|  | Pacheco et al., 2023 | Care and Monitoring of Pregnant Patients With Left Ventricular Assist Devices | Title |
|  | Pack-Mabien et al., 2009 | A primary care provider's guide to preventive and acute care management of adults and children with sickle cell disease | TITLE |
|  | Padilla et al., 2021 | Critical care in obstetrics: a strategy for addressing maternal mortality | TITLE |
|  | Padua et al., 2012 | Sickle cell anemia: a significant potential cause of pulmonary hypertension in Brazil | TITLE |
|  | Page et al., 2007 | Post Transplant Autoimmune Hemolytic Anemia and Other Cytopenias Are Increased in Very Young Babies after Unrelated Donor Umbilical Cord Blood Transplantation | TITLE |
|  | Pakbazet al., 2011 | Serum transferrin: An independent predictor of mortality in sickle cell anemia | TITLE |
|  | Panduranga et al., 2011 | Sickle cell disease with left ventricular non-compaction: A rare association | TITLE |
|  | Panwar et al., 2022 | Pulmonary Dysfunction in Transfusion-Dependent Thalassemia and Response to Intensive Chelation Therapy | TITLE |
|  | Pandyaet al., 2020 | ENLARGED MAIN PULMONARY ARTERY DIAMETER ON COMPUTED TOMOGRAPHY SCAN ASSOCIATED WITH SEVERE RESPIRATORY FAILURE IN PATIENTS WITH ACUTE CHEST SYNDROME | Title |
|  | Panepintoa et al., 2020 | Coronavirus Disease among Persons with Sickle Cell Disease, United States, March 20-May 21, 2020 | TITLE |
|  | Panepucci,et al., 2011 | Association study between SNPs in genes related to adenosine signaling and distinct clinical manifestations in sickle cell disease | TITLE |
|  | Paolini et al., 2004 | Pulmonary hypertension and systemic diseases | ABSTRACT |
|  | Papassotiriou et al., 2019 | Non invasive evaluation of bone marrow activity in patients with sickle cell disease: Correlation with disease features, genotype, markers of erythropoiesis, iron metabolism and hydroxyurea treatment | ABSTRACT |
|  | Pahl et al., 2016 | Original Research: Acute chest syndrome in sickle cell disease: Effect of genotype and asthma | TITLE |
|  | Pais et al., 2023 | Possible Isolated Pulmonic Valve Endocarditis in a Patient With Sickle Cell Disease: A Case Report | TITLE |
|  | Pai et al., 2015 | Spontaneous central retinal artery occlusion in a teenager with sickle cell trait | TITLE |
|  | Palmieri et al., 2009 | Neurologic emergency in children's hospital. Stroke | Title |
|  | Papa et al., 2018 | Secondary, AA, Amyloidosis | TITLE |
|  | Palomarez et al., 2022 | Cardiovascular consequences of sickle cell disease | TITLE |
|  | Park et al., 2011 | Splenic infarction in a patient with autoimmune hemolytic anemia and protein C deficiency | TITLE |
|  | Papassotiriou et al., 2009 | The sFLT-1 to PlGF ratio is impaired in patients with thalassemia and sickle cell disease | TITLE |
|  | Papassotiriouet al., 2010 | SFlt-1 and plgf levels in patients with thalassemia and sickle cell disease | TITLE |
|  | Papassotiriouet al., 2018 | Distinct biomarkers reflect pathophysiological differences of sickle cell disease (SCD) Sub-phenotypes: Viscosity-vaso-occlusion vs. hemolysis-endothelial dysfunction | TITLE |
|  | Patel et al., 2007 | Vasoocclusion by sickled RBCs in 5 autopsy cases of sudden death | ABSTRACT |
|  | Patel et al., 2010 | Placenta growth factor-induced early growth response 1 (Egr-1) regulates hypoxia-inducible factor-1alpha (HIF-1alpha) in endothelial cells | TITLE |
|  | Patel et al., 2022 | A meta-analysis of variability in conjunctival microvascular hemorheology metrics | TITLE |
|  | Papassotiriou et al., 2014 | Targeted metabolomic profiles are strongly correlated with metabolic alterations in patients with sickle cell/beta thalassemia disease | TITLE |
|  | Papassotiriouet al., 2018 | A pilot data analysis of a metabolomic liquid chromatography-tandem mass spectrometry (LC/MS/MS) based study of patients with sickle cell/beta thalassemia | TITLE |
|  | Papassotiriouet al., 2017 | Distinct biomarkers reflect pathophysiological differences of sickle cell disease sub-phenotypes: Viscosity-vaso-occlusion vs. hemolysis-endothelial dysfunction | TITLE |
|  | Papassotiriou et al., 2023 | Assessment of Circulating Retinol Binding Protein-4 Levels in Patients with Compound Heterozygosity for Hb S and β-Thalassemia | ABSTRACT |
|  | Parakaw et al., 2017 | Platelet inhibition and increased phosphorylated vasodilator-stimulated phosphoprotein following sodium nitrite inhalation | TITLE |
|  | Parazzi et al., 2015 | Ventilatory Efficiency in Children and Adolescents: A Systematic Review | Title |
|  | Park et al., 2004 | Hematologic issues in anesthesia: Preface | TITLE |
|  | Parente et al., 2023 | Unusual Forms of Pulmonary Hypertension | TITLE |
|  | Parikh et al., 2010 | Lack of clustering of SCD complications into two distinct subphenotypes in an adult patient population | TITLE |
|  | Parikh et al., 2010 | Are there distinct sub-phenotypes of sickle cell disease? | TITLE |
|  | Parise et al., 2016 | Sickle cell disease: Challenges and progress | TITLE |
|  | Parise et al., 2003 | Erythrocyte adhesion in sickle cell disease | TITLE |
|  | Pashankar et al., 2011 | Pulmonary hypertension and proteinuria in children with sickle cell disease | TITLE |
|  | Pashankaret al., 2013 | Longitudinal natural history study of tricuspid regurgitant jet velocity in untreated children with sickle cell disease | ABSTRACT |
|  | Pashankaret al., 2011 | Effect of hydroxyurea on elevated pulmonary artery pressures in children with sickle cell disease | ABSTRACT |
|  | Pashankar et al., 2009 | Longitudinal follow up of elevated pulmonary artery pressures in children with sickle cell disease | ABSTRACT |
|  | Patel et al., 2008 | Placenta growth factor augments endothelin-1 and endothelin-B receptor expression via hypoxia-inducible factor-1 alpha | ABSTRACT |
|  | Patel et al., 2009 | Placenta growth factor induces 5-lipoxygenase-activating protein to increase leukotriene formation in sickle cell disease | TITLE |
|  | Paterson et al., 2011 | The role of Doppler echocardiography in pulmonary artery hypertension: The importance of proving the obvious | TITLE |
|  | Patil et al., 2017 | Clinical 'pearls' of maternal critical care Part 2: sickle-cell disease in pregnancy | ABSTRACT |
|  | Paul et al., 2017 | Factors associated with pulmonary hypertension and overall survival in adult patients with sickle cell disease: A single centre retrospective study | TITLE |
|  | Paul et al., 2013 | Clinical correlates of acute pulmonary events in children and adolescents with sickle cell disease | ABSTRACT |
|  | Paul et al., 2011 | Acute chest syndrome: Sickle cell disease | ABSTRACT |
|  | Pavan et al., 2022 | The state of the art of fetal hemoglobin-inducing agents | TITLE |
|  | Patel et al., 2008 | Placenta growth factor augments endothelin-1 and endothelin-B receptor expression via hypoxia-inducible factor-1α | TITLE |
|  | Pérez et al., 2010 | Characterization of obstetric patients with multiple organ failure in the intensive care unit of a Havana teaching hospital, 1998 to 2006 | Title |
|  | Phan et al., 2023 | Effect of voxelotor on cardiopulmonary testing in youths with sickle cell anemia in a pilot study | TITLE |
|  | Peasgoodet al., 2023 | Systematic Review of the Effect of a One-Day Versus Seven-Day Recall Duration on Patient Reported Outcome Measures (PROMs) | TITLE |
|  | Peeke et al., 2020 | Immunophenotypes and Clinical Outcomes in Adult Patients with Sickle Cell Disease: Lymphopenia Correlates with End Organ Damage | TITLE |
|  | Pennell et al., 2014 | Review of journal of cardiovascular magnetic resonance 2013 | TITLE |
|  | Pepe et al., 2019 | Prediction of Cardiac Complications in SCD | TITLE |
|  | Pereira et al., 2009 | Iron chelation in patients with thalassemia intermedia and other non transfusion dependent congenital hemolytic anemias | TITLE |
|  | Pereira Nascimento et al., 2017 | Genetic hemolytic marker in sickle cell anaemia | TITLE |
|  | Pervaizet al., 2021 | Pulmonary complications of sickle cell disease: a narrative clinical review | TITLE |
|  | Peacock et al., 2014 | Recommendations for the evaluation and management of observation services: A consensus white paper: The society of cardiovascular patient care | TITLE |
|  | Petrolini et al., 2023 | The Anemic Newborn at Birth: From Diagnosis to Treatment | TITLE |
|  | Phylipsenet al., 2012 | Non-invasive prenatal diagnosis of beta-thalassemia and sickle-cell disease using pyrophosphorolysis-activated polymerization and melting curve analysis | TITLE |
|  | Piccin et al., 2008 | Plasma Adrenomedullin Levels Are Elevated in Pediatric Patients with Severe Sickle Cell Anemia | Abstract |
|  | Piccin et al., 2012 | Composition and significance of splenic Gamna-Gandy bodies in sickle cell anemia | Title |
|  | Pianosi et al., 2017 | Pediatric Exercise Testing: Value and Implications of Peak Oxygen Uptake | TITLE |
|  | Piccin et al., 207 | Circulating microparticles: pathophysiology and clinical implications | TITLE |
|  | Platt et al., 2016 | Endothelial cells and cathepsins: Biochemical and biomechanical regulation | TITLE |
|  | Porter et al., 2014 | Impact of sickle hemoglobinopathies on pregnancy-related venous thromboembolism | TITLE |
|  | Powell et al., 2019 | Abnormal submaximal cardiopulmonary exercise parameters predict impaired peak exercise performance in sickle cell anemia patients | TITLE |
|  | Picot et al., 2014 | Flow cytometry analyses reveal association between Lu/BCAM adhesion molecule and osteonecrosis in sickle cell disease | ABSTRACT |
|  | Piel et al., 2023 | Defining global strategies to improve outcomes in sickle cell disease: a Lancet Haematology Commission | TITLE |
|  | Piel et al., 2017 | Subphenotypes of sickle cell disease in Africa | TITLE |
|  | Pillai et al., 2005 | Sudden unexpected death in an undiagnosed sickle disease | TITLE |
|  | Pincez et al., 2016 | Pulmonary complications of sickle cell disease in children | TITLE |
|  | Pincez et al., 2016 | Atteintes pulmonaires au cours de la drépanocytose chez l’enfant | ABSTRACT |
|  | Pinto et al., 2015 | Effect of aging on cardiopulmonary disease in sickle cell anemia | TITLE |
|  | Pinto Simões et al., 2014 | Indication for stem cell transplantation in sickle cell disease: Is older age a contraindication? single center experience in brazil | ABSTRACT |
|  | Pinto et al., 2019 | Sickle cell disease: a review for the internist | TITLE |
|  | Pinto et al., 2023 | Morbidity and mortality of sickle cell disease patients is unaffected by splenectomy: evidence from three decades of follow-up in a high-income setting | TITLE |
|  | Pitman et al., 2016 | Recent advances in the development of sphingosine kinase inhibitors | ABSTRACT |
|  | Pittman et al., 2017 | Reversal of pre-capillary pulmonary hypertension in a patient with sickle cell anemia who underwent haploidentical peripheral blood stem cell transplantation | TITLE |
|  | Pierce et al., 2014 | Use of procalcitonin for the prediction and treatment of acute bacterial infection in children | ABSTRACT |
|  | Pinto et al., 2021 | Physical exercise in sickle cell anemia: a systematic review | TITLE |
|  | Platts et al., 2023 | Managing a new diagnosis of interstitial lung disease in pregnancy | TITLE |
|  | Pollock et al., 2023 | SNAP: Implementation of Supportive Non-Invasive Ventilation for Acute Chest Syndrome Prevention in Adults with Sickle Cell Disease | TITLE |
|  | Polonifi et al., 2014 | PDGF, PDGFR and TGF gene expression profile in patients with beta-thalassemia and sickle cell disease with and without pulmonary hypertension | TITLE |
|  | Poludasu et al., 2013 | Left Ventricular Systolic Function in Sickle Cell Anemia: A Meta-Analysis | ABSTRACT |
|  | Pontier et al., 2009 | 2009 innovations on idiopathic or familial pulmonary arterial hypertension | ABSTRACT |
|  | Porembskaya et al., 2020 | Pulmonary artery thrombosis: A diagnosis that strives for its independence | TITLE |
|  | Potoka et al., 2015 | Vasculopathy and pulmonary hypertension in sickle cell disease | TITLE |
|  | Potoka et al., 2018 | Nitric oxide–independent soluble guanylate cyclase activation improves vascular function and cardiac remodeling in sickle cell disease | ABSTRACT |
|  | Powars et al., 2005 | Outcome of sickle cell anemia: a 4-decade observational study of 1056 patients | ABSTRACT |
|  | Prakash et al., 2005 | Lungs in hemoglobinopathies, erythrocyte disorders, and hemorrhagic diatheses | ABSTRACT |
|  | Prashant et al., 2020 | Effects of endothelins and matrix metalloproteinases in various pathological disorders and their antagonism at a better treatment modality in modern medicine | TITLE |
|  | Premontet al., 2022 | The enzymatic function of the honorary enzyme: S-nitrosylation of hemoglobin in physiology and medicine | TITLE |
|  | Presley et al., 2010 | Effects of a Single Sickling Event on the Mechanical Fragility of Sickle Cell Trait Erythrocytes | TITLE |
|  | Prasad et al., 2010 | Stroke in young: An Indian perspective | ABSTRACT |
|  | Purandare et al., 2006 | Paradoxical embolization: A potential cause of cerebral damage in Alzheimer's disease? | TITLE |
|  | Puymirat et al., 2019 | Chest pain after a cesarean -section with a puzzling ECG | TITLE |
|  | Premont et al., 2020 | Role of Nitric Oxide Carried by Hemoglobin in Cardiovascular Physiology: Developments on a Three-Gas Respiratory Cycle | TITLE |
|  | Prevost et al., 2018 | Management of patients with sickle cell disease in oral surgery. Literature review and update | ABSTRACT |
|  | Priftis et al., 2006 | Quantification of siderophages in bronchoalveolar fluid in transfusional and primary pulmonary hemosiderosis | TITLE |
|  | Prosser et al., 2002 | Anaphylactic shock due to cefuroxime in a patient taking penicillin prophylaxis | TITLE |
|  | Purohit et al., 2016 | Pulmonary Function Tests in Sickle Cell Disease | TITLE |
|  | Priceet al., 2012 | Pathophysiology of pulmonary hypertension in acute lung injury | TITLE |
|  | Prohaska et al., 2021 | The different facets of sickle cell disease-related pulmonary hypertension | ABSTRACT |
|  | Prohaska et al., 2023 | RASA3 is a candidate gene in sickle cell disease-associated pulmonary hypertension and pulmonary arterial hypertension | TITLE |
|  | Purcell et al., 2007 | Study of Patients Having Splenectomy Due to Trauma for Long Term Infectious and Vascular Complications | ABSTRACT |
|  | Purdy et al., 2022 | P- and E- selectin in venous thrombosis and non-venous pathologies | TITLE |
|  | Pyne et al., 2020 | Sphingosine kinases as druggable targets | TITLE |
|  | Quelal et al., 2023 | Prevalence and predictors of non-rheumatic valvular heart disease in patients with sickle cell disease: insights from the National In-Patient Database in 2016 and 2017 | TITLE |
|  | Quimby et al., 2014 | Clinical findings associated with homozygous sickle cell disease in the Barbadian population--do we need a national SCD registry? | ABSTRACT |
|  | Quinn et al., 2017 | Losartan for the nephropathy of sickle cell anemia: A phase-2, multicenter trial | Abstract |
|  | Quimby et al., 2014 | Clinical findings associated with homozygous sickle cell disease in the Barbadian population - Do we need a national SCD registry? | TITLE |
|  | Qureshi et al., 2015 | Oral contraceptive use and incident stroke in women with sickle cell disease | ABSTRACT |
|  | Quimby et al., 2015 | Intravenous infusion of haptoglobin for the prevention of adverse clinical outcome in Sickle Cell Disease | TITLE |
|  | Quinn et al., 2013 | Sickle cell disease in childhood. from newborn screening through transition to adult medical care | TITLE |
|  | Quinnet al., 2016 | Minireview: Clinical severity in sickle cell disease: the challenges of definition and prognostication | ABSTRACT |
|  | Quinnet al., 2016 | Clinical severity in sickle cell disease: the challenges of definition and prognostication | TITLE |
|  | Quinn et al., 2005 | Clinical correlates of steady-state oxyhaemoglobin desaturation in children who have sickle cell disease | TITLE |
|  | Quinn et al., 2004 | Risk factors and prediction of outcomes in children and adolescents who have sickle cell anemia | ABSTRACT |
|  | Quinnet al., 2016 | A multi-center, phase-2 trial of losartan for the nephropathy of sickle cell anemia | ABSTRACT |
|  | Quinn et al., 2016 | Biochemical surrogate markers of hemolysis do not correlate with directly measured erythrocyte survival in sickle cell anemia | TITLE |
|  | Quirolo et al., 2010 | How do I transfuse patients with sickle cell disease? | ABSTRACT |
|  | Quirozet al., 2010 | Automated red cell exchange in patients with sickle cell disease | TITLE |
|  | Qureshiet al., 2018 | Guidelines for the use of hydroxycarbamide in children and adults with sickle cell disease: A British Society for Haematology Guideline | TITLE |
|  | Qureshiet al., 2004 | Utility of Holter Electrocardiogram Monitoring in Iron over Loaded β Thalassemia and Sickle Cell Disease | TITLE |
|  | Qureshiet al., 2006 | Right ventricular abnormalities in sickle cell anemia: Evidence of a progressive increase in pulmonary vascular resistance | TITLE |
|  | Raamet al., 2016 | Sickle Cell Crisis and You: A How-to Guide | ABSTRACT |
|  | Rabassedaet al., 2010 | A report from the 106th International Conference of the American Thoracic Society (May 14-19, 2010 - New Orleans, Louisiana, USA) | TITLE |
|  | Racho et al., 2020 | Liver Transplantation for Acute Liver Failure Secondary to Acute Sickle Intrahepatic Cholestasis | TITLE |
|  | Ramani et al., 2009 | Standard measures of right ventricular function assessment in adult patients with acute sickle cell crises | TITLE |
|  | Ramgopal et al., 2023 | Comparing two definitions of pediatric complexity among children cared for in general and pediatric emergency departments in a statewide sample | TITLE |
|  | Raghunathan et al., 2018 | Sleep-disordered breathing in patients with sickle cell disease | TITLE |
|  | Raghupathy et al., 2010 | Iron overload in sickle cell disease | TITLE |
|  | Rahimiet al., 2019 | New guidelines for home oxygen therapy in children | TITLE |
|  | Raouf et al., 2000 | Pulmonary complications in sickling diseases | TITLE |
|  | Rai et al., 2021 | Longitudinal effect of disease-modifying therapy on tricuspid regurgitant velocity in children with sickle cell anemia | Abstract |
|  | Rai et al., 2017 | A reappraisal of the mechanisms underlying the cardiac complications of sickle cell anemia | ABSTRACT |
|  | Rai et al., 2023 | The effects of cardio-selective b blockade on diastolic dysfunction in children with sickle cell disease | TITLE |
|  | Raiet al., 2021 | Association of Thrombospondin-1 Gene Polymorphism with Elevated Tricuspid Regurgitant Velocity in Sickle Cell Anemia | ABSTRACT |
|  | Rai et al., 2019 | Hydroxyurea lowers tricuspid regurgitant jet velocity in children with sickle cell anemia | ABSTRACT |
|  | Raiet al., 2019 | HYDROXYUREA LOWERS TRICUSPID REGURGITANT JET VELOCITY IN CHILDREN WITH SICKLE CELL ANEMIA | ABSTRACT |
|  | Ramadas et al., 2023 | The APC-EPCR-PAR1 axis in sickle cell disease | ABSTRACT |
|  | Ramadass et al., 2020 | Small molecule nf-kb pathway inhibitors in clinic | TITLE |
|  | Ramakrishnanet al., 2018 | Pulmonary arterial hypertension: Iron matters | TITLE |
|  | Ramachandran et al., 2020 | Low morbidity and mortality with COVID-19 in sickle cell disease: A single center experience | Abstract |
|  | Raffield et al., 2017 | D-Dimer in African Americans: Whole Genome Sequence Analysis and Relationship to Cardiovascular Disease Risk in the Jackson Heart Study | Title |
|  | Rai et al., 2023 | The effects of cardio-selective β blockade on diastolic dysfunction in children with sickle cell disease | TITLE |
|  | Rai et al., 2023 | Longitudinal effect of disease-modifying therapy on left ventricular diastolic function in children with sickle cell anemia | ABSTRACT |
|  | Raj et al., 2005 | Quantitative assessment of ventricular function in sickle cell disease: effect of long-term erythrocytapheresis | ABSTRACT |
|  | Ramachandran et al., 2020 | Low morbidity and mortality with COVID-19 in sickle cell disease: A single center experience | ABSTRACT |
|  | Ranabothu et al., 2020 | Ambulatory Hypertension in Pediatric Patients With Sickle Cell Disease and Its Association With End-Organ Damage | TITLE |
|  | Rankine-Mullings et al., 2016 | EXpanding Treatment for Existing Neurological Disease (EXTEND): An Open-Label Phase II Clinical Trial of Hydroxyurea Treatment in Sickle Cell Anemia | TITLE |
|  | Ramanet al., 2006 | Myocardial ischemia and right ventricular dysfunction in adult patients with sickle cell disease | TITLE |
|  | Ramseyet al., 2022 | Prevalence of comorbidities associated with sickle cell disease among non-elderly individuals with commercial insurance-A retrospective cohort study | TITLE |
|  | Rani et al., 2015 | Correlates of nocturnal hypoxemia in children with sickle cell disease | ABSTRACT |
|  | Ranque et al., 2020 | Studying the determinants of sickle cell disease vasculopathy in sub-saharan Africa : The biocadre study | Title |
|  | Ranque et al., 2014 | Early renal damage in patients with sickle cell disease in sub-Saharan Africa: A multinational, prospective, cross-sectional study | ABSTRACT |
|  | Ranqueet al., 2023 | Association of haemolysis markers, blood viscosity and microcirculation function with organ damage in sickle cell disease in sub-Saharan Africa (the BIOCADRE study) | ABSTRACT |
|  | Ranu et al., 2011 | Breathlessness and chest pain in a patient with sickle cell disease | ABSTRACT |
|  | Rao et al., 2020 | The Author's Contributions to Echocardiography Literature (Part I-1978-1990) (†) | Title |
|  | Rasulo et al., 2019 | Transcranial Doppler and Optic Nerve Sonography | TITLE |
|  | Raut et al., 2014 | Perioperative considerations in a sickle cell patient undergoing cardiopulmonary bypass | TITLE |
|  | Ravishankar et al., 2021 | Aortic valve replacement in a patient with sickle cell disease-Are we justified to perform surgery in the TAVI era? | TITLE |
|  | Ranuet al., 2011 | The knowledge of pulmonary complications associated with sickle cell disease amongst a group of respiratory trainees in the UK | TITLE |
|  | Rasheedet al., 2012 | Patterns and prevalence of pulmonary funtion test (PFT) abnormalities in children and adolescents with sickle cell disease (SCD) | ABSTRACT |
|  | Rashidet al., 2020 | Therapeutic Potential of Citrulline as an Arginine Supplement: A Clinical Pharmacology Review | ABSTRACT |
|  | Rasseet al., 2015 | Capillary-hemangiomatosis like lesions in the lungs of sickle cell anemia patients | TITLE |
|  | Ravi et al., 2022 | Measuring Physical Activity in Younger and Older Adults with Sickle Cell Disease Using Accelerometers | TITLE |
|  | Ravishankar et al., 2021 | Aortic valve replacement in a patient with sickle cell disease—Are we justified to perform surgery in the TAVI era? | TITLE |
|  | Rayburg et al., 2010 | Fatal bone marrow embolism in a child with hemoglobin SE disease | TITLE |
|  | Razdan et al., 2016 | Patent foramen ovale in adults with sickle cell disease and stroke | TITLE |
|  | Rayet al., 2020 | Left or Right? When Diffuse T-Wave Inversion Is Worse Than Coronary Ischemia | TITLE |
|  | Rechaviet al., 2008 | Regulation of iron absorption in hemoglobinopathies | TITLE |
|  | Redding-Lallingeret al., 2006 | Questions in the management of sickle cell | TITLE |
|  | Redding-Lallinger et al., 2006 | Sickle Cell Disease-Pathophysiology and Treatment | TITLE |
|  | Reddy et al., 2016 | High-Output Heart Failure in Sickle Cell Anemia | TITLE |
|  | Redinus et al., 2019 | An Hb-mediated circulating macrophage contributing to pulmonary vascular remodeling in sickle cell disease | TITLE |
|  | Reed et al., 2014 | Multiple bilateral branch retinal artery occlusions in a patient with sickle cell disease with vancomycin red man syndrome | Title |
|  | Reedet al., 2014 | Multiple bilateral branch retinal artery occlusions in a patient with sickle cell disease with vancomycin red man syndrome | TITLE |
|  | Reeset al., 2008 | A simple index using age, hemoglobin, and aspartate transaminase predicts increased intracerebral blood velocity as measured by transcranial Doppler scanning in children with sickle cell anemia | TITLE |
|  | Reidy et al., 2018 | Genetic risk of APOL1 and kidney disease in children and young adults of African ancestry | ABSTRACT |
|  | Resende Cardoso et al., 2014 | Clinical complications in pregnant women with sickle cell disease: prospective study of factors predicting maternal death or near miss | TITLE |
|  | Resiere et al., 2016 | Inter-regional medical cooperation in the Caribbean : a major asset for the influence of the French medicine | TITLE |
|  | Rees et al., 2012 | Biomarkers in sickle cell disease | TITLE |
|  | Rees et al., 2018 | How I manage red cell transfusions in patients with sickle cell disease | TITLE |
|  | Rees et al., 2010 | Sickle-cell disease | TITLE |
|  | Reinet al., 2023 | Evaluation of lung function in a German single center cohort of young patients with sickle cell disease using EIT and standard techniques | TITLE |
|  | Reiteret al., 2003 | An emerging role for nitric oxide in sickle cell disease vascular homeostasis and therapy | Title |
|  | Reiteret al., 2018 | Cardiac magnetic resonance T1 mapping. Part 2: Diagnostic potential and applications | ABSTRACT |
|  | Renet al., 2017 | Cardiorespiratory pathogenesis of sickle cell disease in a mouse model | TITLE |
|  | Resendeet al., 2020 | Prognostic value of left ventricular longitudinal strain by speckle-tracking echocardiography in patients with sickle cell disease | TITLE |
|  | Restenet al., 2005 | CT imaging of peripheral pulmonary vessel disease | ABSTRACT |
|  | Reyes Barronet al., 2021 | Myocardial Calcification and the Demise of an Infant With Surgically Treated Hypoplastic Left Heart Syndrome | TITLE |
|  | Rezendeet al., 2018 | Clinical and hematological profile in a newborn cohort with hemoglobin SC | Title |
|  | Rifkin-Zenenberget al., 2020 | Cteph in a homozygous sickle cell patient without significant right heart disease | TITLE |
|  | Riganoet al., 2018 | Real-life experience with hydroxyurea in sickle cell disease: A multicenter study in a cohort of patients with heterogeneous descent | TITLE |
|  | Ristowet al., 2011 | Pulmonary hypertension in sickle cell disease | ABSTRACT |
|  | Ribeiro et al., 2019 | The challenges of handling deferasirox in sickle cell disease patients older than 40 years | ABSTRACT |
|  | Rich et al., 2013 | Rebuttal from Dr Rich | ABSTRACT |
|  | Riel-Romero et al., 2009 | Childhood and teenage stroke | TITLE |
|  | Rizk et al., 2017 | Perinatal Maternal Mortality in Sickle Cell Anemia: Two Case Reports and Review of the Literature | TITLE |
|  | Rissatto-Lago et al., 2017 | Association between endothelial dysfunction and otoneurological symptoms in children with sickle cell disease | TITLE |
|  | Rosa et al., 2015 | Paediatric arterial ischemic stroke: Acute management, recent advances and remaining issues | TITLE |
|  | Rotta et al., 2020 | Characterization of In-Flight Medical Events Involving Children on Commercial Airline Flights | TITLE |
|  | Roach et al., 2008 | Management of stroke in infants and children: a scientific statement from a Special Writing Group of the American Heart Association Stroke Council and the Council on Cardiovascular Disease in the Young | TITLE |
|  | Roach et al., 2020 | Ca(2+) signalling in fibroblasts and the therapeutic potential of K(Ca)3.1 channel blockers in fibrotic diseases | TITLE |
|  | Robinson et al., 2019 | The Impact of APOL1 on Chronic Kidney Disease and Hypertension | TITLE |
|  | Roger et al., 2021 | Risk factors for CKD stage II onset in a prospective cohort of homozygous sickle cell adults | TITLE |
|  | Robbins et al., 2012 | National Heart, Lung, and Blood Institute workshop: Improving outcomes for pulmonary vascular disease | TITLE |
|  | Robinson al., 2014 | The crossroads of iron with hypoxia and cellular metabolism: Implications in the pathobiology of pulmonary hypertension | TITLE |
|  | Rodríguez-Rodríguez et al., 2019 | Genome editing: A perspective on the application of CRISPR/Cas9 to study human diseases (Review) | Title |
|  | Roe et al., 2022 | Sex as an Independent Risk Factor for Venous Thromboembolism in Sickle Cell Disease: A Cross-Sectional Study | Title |
|  | Roberts et al., 2012 | Queenan's Management of High-Risk Pregnancy: An Evidence-Based Approach: Sixth Edition | ABSTRACT |
|  | Roeuthet al., 2023 | FAT EMBOLISM SYNDROME IN A PATIENT WITH SICKLE CELL CRISIS MIMICKING THROMBOTIC THROMBOCYTOPENIC PURPURA | Title |
|  | Rogers et al., 2006 | Review: Clinical transfusion management in sickle cell disease | TITLE |
|  | Rojas et al., 2002 | Pulmonary hypertension in patients with sickle cell anemia | TITLE |
|  | Rojas-Jiménez et al., 2013 | Cardiopulmonary complications in sickle cell anemia | ABSTRACT |
|  | Romana et al., 2018 | Microparticles in sickle cell disease | ABSTRACT |
|  | Romanoet al., 2020 | Management of cesarean delivery in a parturient with sickle cell disease | TITLE |
|  | Romero et al., 2006 | Therapeutic use of citrulline in cardiovascular disease | TITLE |
|  | Rondelli, et al., 2016 | Chemotherapy-free allogeneic hematopoietic stem cell transplantation for adult patients with sickle cell disease (SCD) | TITLE |
|  | Rosa et al., 2022 | Effectiveness and safety of gonadotropins used in female infertility: a population-based study in the Lazio region, Italy | TITLE |
|  | Rosanwo et al., 2019 | Care of the Critically Ill Pediatric Sickle Cell Patient | TITLE |
|  | Rosart et al., 2018 | Validation of sickle cell disease severity score in a cohort of hemoglobin SC disease patients | TITLE |
|  | Rosenberg et al., 2010 | Sickle retinopathy: Associated factors and predictive value for systemic manifestations | ABSTRACT |
|  | Rosenberg et al., 2011 | Pediatric sickle cell retinopathy: correlation with clinical factors | TITLE |
|  | Rosenzweig et al., 2008 | Pulmonary arterial hypertension in children: A medical update | TITLE |
|  | Rother et al., 2005 | The clinical sequelae of intravascular hemolysis and extracellular plasma hemoglobin: a novel mechanism of human disease | ABSTRACT |
|  | Rossaint et al., 2014 | Our paper 20 years later: Inhaled nitric oxide for the acute respiratory distress syndrome—discovery, current understanding, and focussed targets of future applications | TITLE |
|  | Rother et al., 2005 | The clinical sequelae of intravascular hemolysis and extracellular plasma hemoglobin - A novel mechanism of human disease | TITLE |
|  | Rotin et al., 2020 | Allogeneic Hematopoietic Stem Cell Transplant Versus Gene Therapy in Sickle Cell Disease: Updated Results from a Systematic Review | ABSTRACT |
|  | Rotinet al., 2023 | A systematic review comparing allogeneic hematopoietic stem cell transplant to gene therapy in sickle cell disease | Title |
|  | Rotzet al., 2015 | Traffic Light: prognosis-based eligibility for clinical trials of hematopoietic SCT in adults with sickle cell anemia | TITLE |
|  | Royet et al., 2023 | Interventions for chronic kidney disease in people with sickle cell disease | TITLE |
|  | Rouxel et al., 2008 | Pulmonary infarction without fat or thrombo-embolism in sickle cell anaemia | TITLE |
|  | Rozi et al., 2021 | Use of rivaroxaban in sickle cell disease and venous thromboembolism: A case report | TITLE |
|  | Rubin et al., 2011 | New frontiers in pulmonary hypertension | TITLE |
|  | Rucknagel et al., 2001 | The role of rib infarcts in the acute chest syndrome of sickle cell diseases | TITLE |
|  | Ruhlet al., 2019 | Identifying Clinical and Research Priorities in Sickle Cell Lung Disease. An Official American Thoracic Society Workshop Report | TITLE |
|  | Ryan et a;., 2023 | PREGNANCY OUTCOMES IN WOMEN WITH SICKLE CELL DISEASE IN IRELAND: A RETROSPECTIVE REVIEW | TITLE |
|  | Saah et al., 2022 | Sickle Cell Disease Pathophysiology and Related Molecular and Biophysical Biomarkers | TITLE |
|  | Sabatini et al., 2022 | Echocardiographic Evaluation in Paediatric Sickle Cell Disease Patients: A Pilot Study | TITLE |
|  | Sachdev et al., 2019 | Reversal of a rheologic cardiomyopathy following hematopoietic stem cell transplantation for sickle cell disease | ABSTRACT |
|  | Sachdev et al., 2023 | Cardiac effects 2 years after successful non-myeloablative human leukocyte antigen-matched related donor hematopoietic cell transplants in sickle cell disease | TITLE |
|  | Sadeghi et al., 2014 | Pregnancy-related acute myocardial infarction: A review of epidemiology, Diagnosis, Medical and surgical management | TITLE |
|  | Sacco et al., 2006 | Guidelines for prevention of stroke in patients with ischemic stroke or transient ischemic attack: a statement for healthcare professionals from the American Heart Association/American Stroke Association Council on Stroke: co-sponsored by the Council on Cardiovascular Radiology and Intervention: the American Academy of Neurology affirms the value of this guideline | TITLE |
|  | Sachdev et al., 2021 | A phenotypic risk score for predicting mortality in sickle cell disease | TITLE |
|  | Sachdev et al., 2007 | Diastolic Dysfunction Is an Independent Risk Factor for Death in Patients With Sickle Cell Disease | ABSTRACT |
|  | Sachdevt al., 2021 | Cardiovascular complications of sickle cell disease | ABSTRACT |
|  | Sachithanandan et al., 2008 | Mitral and tricuspid valve surgery in homozygous sickle cell disease: perioperative considerations for a successful outcome | ABSTRACT |
|  | Sadreameli et al., 2017 | Asthma Screening in Pediatric Sickle Cell Disease: A Clinic-Based Program Using Questionnaires and Spirometry | ABSTRACT |
|  | Sadeghi Ghahrodi et al., 2013 | Ambulatory blood pressure monitoring for children with Beta-Thalassemia major: A preliminary | TITLE |
|  | Sadreameli et al., 2016 | Secondhand Smoke Is an Important Modifiable Risk Factor in Sickle Cell Disease: A Review of the Current Literature and Areas for Future Research | Title |
|  | Salama et al., 2020 | The relationships between pancreatic T2* values and pancreatic iron loading with cardiac dysfunctions,  hepatic and cardiac iron siderosis among Egyptian children and young adults with β-thalassaemia major and sickle cell disease: a cross-sectional study | TITLE |
|  | Salih et al., 2006 | Cardiac diseases as a risk factor for stroke in Saudi children | TITLE |
|  | Samb et al., 2005 | Physical performance and thermoregulatory study of subjects with sickle cell trait during a sub-maximal exercise | TITLE |
|  | Sanchis-Gomar et al., 2015 | Effects of allopurinol on exercise-induced muscle damage: new therapeutic approaches? | ABSTRACT |
|  | Sangkatumvong et al., 2008 | Abnormal autonomic cardiac response to transient hypoxia in sickle cell anemia | TITLE |
|  | Sangkatumvong et al., 2008 | Abnormal cardiac autonomic control in sickle cell disease following transient hypoxia | TITLE |
|  | Sanya et al., 2010 | Cardiovascular autonomic reflex function in sickle cell anaemia patients | TITLE |
|  | Sadaf et al., 2020 | L-glutamine for sickle cell disease: Knight or pawn? | ABSTRACT |
|  | Saenz et al., 2015 | Public titles of clinical trials should have ethics review | TITLE |
|  | Sahay et al., 2012 | Partial anomalous pulmonary venous connection and pulmonary arterial hypertension | TITLE |
|  | Sahu et al., 2021 | Common, But Neglected: A Comprehensive Review of Leg Ulcers in Sickle Cell Disease | TITLE |
|  | Sakhalkar et al., 2009 | Case study category: Pediatric type: Transfusion, pulmonary hypertension: Delayed hemolytic transfusion reaction in a patient with sickle cell disease | TITLE |
|  | Said et al., 2022 | Prevalence of iron deficiency and iron deficiency anaemia among children with congenital heart defects at tertiary hospitals in Dar es Salaam, Tanzania: a cross-sectional study | TITLE |
|  | Saleemi et al., 2014 | Saudi Guidelines on the Diagnosis and Treatment of Pulmonary Hypertension: Pulmonary hypertension associated with hemolytic anemia | TITLE |
|  | Salib et al., 2021 | Digital AI in Hematology - Integration of the Scopio Labs x100 Scanner with Newly Implemented AI Capabilities into Routine Clinical Workflow | TITLE |
|  | Salvagno et al., 2015 | Red blood cell distribution width: A simple parameter with multiple clinical applications | TITLE |
|  | Salvatore et al., 2021 | Selection of the optimal candidate to mitraclip for secondary mitral regurgitation: Beyond mitral valve morphology | TITLE |
|  | Samarah et al., 2021 | Plasma Lipids and Lipoproteins in Sickle Cell Disease Patients in the Northern West Bank, Palestine | TITLE |
|  | Samrahet al., 2020 | Fusarium-Induced Cellulitis in an Immunocompetent Patient With Sickle Cell Disease: A Case Report | Title |
|  | Sanders et al., 2014 | Sickle cell disease and complex congenital cardiac surgery: A case report and review of the pathophysiology and perioperative management | TITLE |
|  | Sandhu et al., 2015 | Aging in sickle cell disease: Co-morbidities and new issues in management | TITLE |
|  | Sara et al., 2006 | Lactate distribution in the blood compartments of sickle cell trait carriers during incremental exercise and recovery | TITLE |
|  | Sariakçali et al., 2013 | Radiocontrast-related leukocytoclastic vasculitis misdiagnosed as diabetic foot ulcer in a type 2 diabetic patient: A case report | TITLE |
|  | Sandner et al., 2018 | Discovery and development of sGC stimulators for the treatment of pulmonary hypertension and rare diseases | TITLE |
|  | Sandneret al., 2021 | Soluble Guanylate Cyclase Stimulators and Activators | TITLE |
|  | Sange et al., 2021 | Sickle Cell Disease and the Respiratory System: A Tangential Perspective to the Hematopulmonological Dilemma | TITLE |
|  | Sangokoya et al., 2010 | microRNA miR-144 modulates oxidative stress tolerance and associates with anemia severity in sickle cell disease | TITLE |
|  | Sanil, et al., 2019 | Effect of Iron Overload on Right Ventricular Function in Children with Sickle Cell Anemia | TITLE |
|  | Santiagoet al., 2020 | <i>TGFBR3</i> Polymorphisms (rs1805110 and rs7526590) Are Associated with Laboratory Biomarkers and Clinical Manifestations in Sickle Cell Anemia | TITLE |
|  | Santiagoet al., 2020 | Transforming Growth Factor Beta Receptor 3 Haplotypes in Sickle Cell Disease Are Associated with Lipid Profile and Clinical Manifestations | ABSTRACT |
|  | Santoset al., 2022 | INDICATIONS OF HEMATOPOIETIC STEM CELL TRANSPLANT ACCORDING TO DIFFERENT SETS OF INDICATIONS AND ANALYSIS OF COMORBIDITY INDEX SCORES IN BRAZILIAN PATIENTS WITH SICKLE CELL DISEASE | ABSTRACT |
|  | Saraf et al., 2017 | Associations of a-thalassemia and BCL11A with stroke in Nigerian, United States, and United Kingdom sickle cell anemia cohorts | TITLE |
|  | Saraf et al., 2017 | Associations of α-thalassemia and BCL11A with stroke in Nigerian, United States, and United Kingdom sickle cell anemia cohorts | ABSTRACT |
|  | Saraf et al., 2016 | Nonmyeloablative Stem Cell Transplantation with Alemtuzumab/Low-Dose Irradiation to Cure and Improve the Quality of Life of Adults with Sickle Cell Disease | ABSTRACT |
|  | Schaer et al., 2013 | Mechanisms of haptoglobin protection against hemoglobin peroxidation triggered endothelial damage | TITLE |
|  | Schaer et al., 2016 | Haptoglobin Preserves Vascular Nitric Oxide Signaling during Hemolysis | TITLE |
|  | Sarafet al., 2016 | Genetic modifiers identify a high risk group for stroke in three independent cohorts of sickle cell anemia patients | TITLE |
|  | Sarafet al., 2019 | Manifestations of Reduced Kidney Function Occur at a Higher Estimated Glomerular Filtration Rate in Sickle Cell Anemia | ABSTRACT |
|  | Saraf et al., 2014 | Differences in the clinical and genotypic presentation of sickle cell disease around the world | TITLE |
|  | Saraf,et al., 2014 | Haemoglobinuria is associated with chronic kidney disease and its progression in patients with sickle cell anaemia | ABSTRACT |
|  | Sarafet al., 2015 | Genetic variants and cell-free hemoglobin processing in sickle cell nephropathy | TITLE |
|  | Sarodeet al., 2017 | Red blood cell exchange: 2015 American Society for Apheresis consensus conference on the management of patients with sickle cell disease | TITLE |
|  | Sathiet al., 2019 | Unusually High Prevalence of Silent Stroke and Cerebral Vasculopathy in Hemoglobin SC Disease | TITLE |
|  | Saunthararajahet al., 2019 | Targeting sickle cell disease root-cause pathophysiology with small molecules | Title |
|  | Saunders et al., 2022 | Plastic bronchitis: Autopsy findings in the sudden death of a healthy pediatric patient with sickle cell disease | TITLE |
|  | Savage et al., 2011 | Mobile right atrial thrombi in a patient with the hemoglobin SC disease | TITLE |
|  | Sathi et al., 2022 | Unusually High Prevalence of Stroke and Cerebral Vasculopathy in Hemoglobin SC Disease: A Retrospective Single Institution Study | TITLE |
|  | Savaleet al., 2019 | Clinical phenotypes and outcomes of precapillary pulmonary hypertension of sickle cell disease | Abstract |
|  | Savaleet al., 2018 | Predictive Factors for Survival in Sickle Cell Disease: A Cohort Study Using Etendard Data | ABSTRACT |
|  | Savale et al., 2013 | Pulmonary arterial hypertension and sickle cell disease | ABSTRACT |
|  | Sayinet al., 2019 | MITRACLIP FOR THE TREATMENT OF SEVERE MITRAL REGURGITATION IN PATIENTS WITH HEART FAILURE | ABSTRACT |
|  | Sayres et al., 2020 | Preconception Genetic Screening | TITLE |
|  | Schaeret al., 2013 | Cell-free hemoglobin and its scavenger proteins: New disease models leading the way to targeted therapies | Title |
|  | Schaffer et al., 2016 | Rare presentation of four primary pediatric cardiac tumors | TITLE |
|  | Schatlo et al., 2007 | Clinical applications of transcranial Doppler sonography | TITLE |
|  | Scheint al., 2008 | Magnetic resonance detection of kidney iron deposition in sickle cell disease: A marker of chronic hemolysis | TITLE |
|  | Schimmelet al., 2013 | Relatively low mortality of sickle cell patients with elevated tricuspid regurgitant jet flow velocity after extended follow up in a dutch cohort | TITLE |
|  | Schimmelet al., 2015 | N-terminal pro-B-type natriuretic peptide, tricuspid jet flow velocity, and death in adults with sickle cell disease | ABSTRACT |
|  | Schmidt et al., 2023 | Release of hepatic xanthine oxidase (XO) to the circulation is protective in intravascular hemolytic crisis | ABSTRACT |
|  | Schmitz et al., 2012 | Sedation in the radiology suite | TITLE |
|  | Schnoget al., 2005 | Plasma levels of asymmetric dimethylarginine (ADMA), an endogenous nitric oxide synthase inhibitor, are elevated in sickle cell disease | TITLE |
|  | Schnoget al., 2023 | An analytical view of the BJH publication of ‘a clinician's view of voxelotor’ | TITLE |
|  | Schizas et al., 2020 | Concomitant Laparoscopic Splenectomy and Cholecystectomy: A Systematic Review of the Literature | TITLE |
|  | Schmidt et al., 2021 | Xanthine Oxidase Drives Hemolysis and Vascular Malfunction in Sickle Cell Disease | Title |
|  | Schmitz et al., 2013 | Increased monocyte adhesion by endothelial expression of VCAM-1 missense variation in vitro | TITLE |
|  | Schommer et al., 2012 | Health risk for athletes at moderate altitude and normobaric hypoxia | TITLE |
|  | Schreieret al., 2016 | Increased Red Blood Cell Stiffness Increases Pulmonary Vascular Resistance and Pulmonary Arterial Pressure | TITLE |
|  | Schreier et al., 2021 | Effects of Red Blood Cell Sickling on Right Ventricular Afterload in vivo | ABSTRACT |
|  | Schyrr et al., 2020 | Perioperative care of children with sickle cell disease: A systematic review and clinical recommendations | TITLE |
|  | Sehatzadeh et al., 2012 | Influenza and pneumococcal vaccinations for patients with chronic obstructive pulmonary disease (COPD): an evidence-based review | TITLE |
|  | Schreieret al., 2021 | Effects of Red Blood Cell Sickling on Right Ventricular Afterload <i>in vivo</i> | TITLE |
|  | Schubert et al., 2005 | Schisis in sickle cell retinopathy | TITLE |
|  | Schumackeret al., 2018 | Rescuing decrepit soluble guanylate cyclase: A therapy for sickle cell disease? | TITLE |
|  | Schwaiger et al., 2013 | Screening patients with scleroderma for pulmonary arterial hypertension and implications for other at-risk populations | TITLE |
|  | Scottet al., 2022 | Clinical Vignettes, Part II: Eyes, Teeth, and Bone | TITLE |
|  | Sebastiani et al., 2007 | A network model to predict the risk of death in sickle cell disease | TITLE |
|  | Sebastianiet al., 2006 | Severity of Sickle Cell Disease: Modeling Interrelationships among Hemolysis, Pulmonary Hypertension and Risk of Death | TITLE |
|  | See et al., 2023 | Factors linked to Staphylococcus aureus healthcare-associated infections among pediatric intensive care unit colonized patients | ABSTRACT |
|  | Ségot et al., 2016 | Pulmonary and cerebral microvasculopathy in a patient with sickle cell disease: A role for dense red blood cells? | TITLE |
|  | Segovia et al., 2018 | High Risk Haitian pregnant patient and utility of the ultrasound in the differential diagnosis in a cardiac arrest: A case report | ABSTRACT |
|  | Selim et al., 2021 | The relationships between pancreatic T2* values and pancreatic iron loading with cardiac dysfunctions, hepatic and cardiac iron siderosis among Egyptian children and young adults with β-thalassaemia major and sickle cell disease: A cross-sectional study | TITLE |
|  | Seliem et al., 2002 | Left ventricular diastolic dysfunction in congenital chronic anaemias during childhood as determined by comprehensive echocardiographic imaging including acoustic quantification | Abstract |
|  | Sen et al., 2009 | Pulmonary function and airway hyperresponsiveness in adults with sickle cell disease | ABSTRACT |
|  | Sengupta et al., 2012 | Left ventricular myocardial performance assessed by 2-dimensional speckle tracking echocardiography in patients with sickle cell crisis | ABSTRACT |
|  | Sebastiani et al., 2010 | Genetic modifiers of the severity of sickle cell anemia identified through a genome-wide association study | ABSTRACT |
|  | Sebastianiet al., 2007 | A Repertoire of Genes Modifying the Risk of Death in Sickle Cell Anemia | ABSTRACT |
|  | Sewaralthahab et al., 2018 | Successful Use of Veno-Venous Extracorporeal Membrane Oxygenation in an Adult Patient with Sickle Cell Anemia and Severe Acute Chest Syndrome | ABSTRACT |
|  | Sedraket al., 2006 | Pulmonary Hypertension in Children and Adolescents with Sickle Cell Disease | TITLE |
|  | Segel et al., 2021 | Balloon pulmonary angioplasty for inoperable chronic thromboembolic pulmonary hypertension: First Experience at the Israeli national CTEPH referral center | ABSTRACT |
|  | Semionov et al., 2018 | Thoracic diseases with musculoskeletal manifestations and vice versa: A review | TITLE |
|  | Serarslan et al., 2009 | High prevalence of pulmonary hypertension in homozygous sickle cell patients with leg ulceration | TITLE |
|  | Serarslan et al., 2006 | Leg ulcer, pulmonary hypertension and silent cerebral infarct in two patients with sickle cell disease | ABSTRACT |
|  | Sernich et al., 2013 | Rapidly growing cardiac papillary fibroelastoma in a teenager with sickle cell disease | TITLE |
|  | Seto et al., 2011 | The preparticipation physical examination: An update | TITLE |
|  | Setty et al., 2008 | Heme induces endothelial tissue factor expression: potential role in hemostatic activation in patients with hemolytic anemia | TITLE |
|  | Setty et al., 2017 | Pathophysiologic implications of type-2 phosphatidylserine-positive erythrocytes in hemoglobin ss disease (sickle cell anemia) and β-thalassemia | ABSTRACT |
|  | Shah et al., 2019 | Evaluation of Vaso-occlusive Crises in United States Sickle Cell Disease Patients: A Retrospective Claims-based Study | Title |
|  | Shah et al., 2021 | Tricuspid regurgitant jet velocity and myocardial tissue Doppler parameters predict mortality in a cohort of patients with sickle cell disease spanning from pediatric to adult age groups - revisiting this controversial concept after 16 years of additional evidence | TITLE |
|  | Shahet al., 2021 | A CASE OF PULMONARY ARTERIAL HYPERTENSION FROM VITAMIN C DEFICIENCY | ABSTRACT |
|  | Shaikh et al., 2020 | Correlation of Iron Levels with Asthma and Lung Function in Pediatric Patients with Sickle Cell Anemia | TITLE |
|  | Shahid et al., 2019 | Risk factors and subtypes of ischemic stroke in young patients: An observational study from a teaching hospital in Saudi Arabia | TITLE |
|  | Shalby et al., 2022 | Extremes of Anemia: The Lowest Hemoglobin Values Probably Ever Reported in the Pediatric Literature Attributed to Iron Deficiency Anemia | ABSTRACT |
|  | Sharma et al., 2011 | Role of diagnostic ultrasound in patient selection for stroke intervention | TITLE |
|  | Shalevet al., 2017 | Morbidity and mortality of adult patients with congenital dyserythropoietic anemia type I | TITLE |
|  | Sharma et al., 2020 | RED CELL DISTRIBUTION WIDTH: A DIAGNOSTIC AID FOR PULMONARY HYPERTENSION? | TITLE |
|  | Sharma et al., 2013 | Pulmonary artery occlusion pressure may overdiagnose pulmonary artery hypertension in sickle cell disease | TITLE |
|  | Silva et al., 2023 | Functional capacity and quality of life in children and adolescents with sickle cell anemia | ABSTRACT |
|  | Sharpe et al., 2014 | How I treat renal complications in sickle cell disease | ABSTRACT |
|  | Shawkat et al., 2011 | Chronic thromboembolic pulmonary hypertension in a patient with sickle cell trait | TITLE |
|  | Sheemole et al., 2016 | Phytochemical analysis of benincasa hispida(Thunb.)cogn. fruit using LC-MS technique | TITLE |
|  | Sheikh et al., 2021 | Sickle Cell Disease-Induced Pulmonary Hypertension: A Review of Pathophysiology, Management, and Current Literature | TITLE |
|  | Shelton et al., 2022 | Factors associated with contraceptive use among postpartum women with substance use disorder | ABSTRACT |
|  | Shen et al., 2022 | Chinese Herbal Medicines Have Potentially Beneficial Effects on the Perinatal Outcomes of Pregnant Women | TITLE |
|  | Shenoy et al., 2016 | A trial of unrelated donor marrow transplantation for children with severe sickle cell disease | Title |
|  | Sheriff et al., 2020 | Neurocritical Care Ultrasound | TITLE |
|  | Shet et al., 2020 | The molecular basis for the prothrombotic state in sickle cell disease | TITLE |
|  | Shenoy et al., 2007 | Has stem cell transplantation come of age in the treatment of sickle cell disease? | TITLE |
|  | Shenoy et al., 2013 | Hematopoietic stem-cell transplantation for sickle cell disease: Current evidence and opinions | TITLE |
|  | Shenoye et al., 2013 | Umbilical cord blood: An evolving stem cell source for sickle cell disease transplants | TITLE |
|  | Shetet al., 2019 | A Growing Population of Older Adults with Sickle Cell Disease | TITLE |
|  | Shi et al., 2019 | Rare diseases in China: analysis of 2014-2015 hospitalization summary reports for 281 rare diseases from 96 tertiary hospitals | TITLE |
|  | Shiloet al., 2017 | Pathways to pulmonary hypertension in sickle cell disease: the search for prevention and early intervention | TITLE |
|  | Shizukuda et al., 2019 | Iron overload and arrhythmias: Influence of confounding factors | TITLE |
|  | Siddeswari et al., 2019 | Tetralogy of fallot with right internal jugular vein thrombosis | TITLE |
|  | Silaghi et al., 2023 | RESOLUTION OF SICKLE CELL CRISIS FOLLOWING ADMINISTRATION OF AMIODARONE | TITLE |
|  | Shinde et al., 2023 | Screening for Multiple Organ Damage in Indian Sickle Cell Disease Cohort- a Step Towards Comprehensive Care | TITLE |
|  | Shinde et al., 2011 | Rare but fatal: The seventh sickle cell nephropathy | TITLE |
|  | Shivaet al., 2019 | Editorial overview: Redox regulation of health and disease | TITLE |
|  | Sinha et al., 2023 | Using Less Than Perfect Data Is Better Than Using No Data At All | TITLE |
|  | Sissons et al., 2019 | Anaesthesia for patients with sickle cell and other haemoglobinopathies | TITLE |
|  | Sliwa et al., 2023 | Nutritional Heart Disease and Cardiomyopathies: JACC Focus Seminar 4/4 | TITLE |
|  | Small et al., 2022 | Diagnostic Features of Emphysematous Osteomyelitis | TITLE |
|  | Smith et al., 2014 | A technique for mitral valve surgery in sickle cell disease | TITLE |
|  | Shomeet al., 2018 | The platelet count and its implications in sickle cell disease patients admitted for intensive care | Title |
|  | Shusterman et al., 2021 | Multi-organ system failure in a patient with sickle cell disease and COVID-19 infection | TITLE |
|  | Siddiqui et al., 2003 | Pulmonary manifestations of sickle cell disease | TITLE |
|  | Siddiquiet al., 2021 | Further evidence for the benefit of therapeutic plasma exchange for acute multi-organ failure syndrome refractory to red cell exchange in sickle cell disease | ABSTRACT |
|  | Silveira et al., 2018 | Aortic Valve Replacement Combined with Ascending Aortic Aneurysmectomy in a Patient with Sickle Cell Disease: a Case Report | TITLE |
|  | Singh et al., 2010 | Live/Real time three-dimensional transthoracic echocardiographic assessment of inferior vena cava and hepatic vein thrombosis in sickle cell disease | TITLE |
|  | Singh et al., 2006 | Cardiopulmonary bypass in sickle cell disease | TITLE |
|  | Silbermins et al., 2009 | Retrospective review of the natural history of pulmonary hypertension in sickle cell disease demonstrates that progressive enlargement of the left atrium is a strong predictor of death | TITLE |
|  | Silbermins et al., 2008 | Prolonged Survival despite High Disease Burden in Elderly (≥55) Patients with Hb SS or Hb Sβ0 Thalassemia | TITLE |
|  | Silva et al., 2023 | From Stress to Sick(le) and Back Again-Oxidative/Antioxidant Mechanisms, Genetic Modulation, and Cerebrovascular Disease in Children with Sickle Cell Anemia | ABSTRACT |
|  | Silva-Pinto et al., 2014 | Sickle cell disease and pregnancy: analysis of 34 patients followed at the Regional Blood Center of Ribeirão Preto, Brazil | Title |
|  | Sivalingam et al., 2020 | COVID‐19 and the pulmonary complications of sickle cell disease | TITLE |
|  | Simba et al., 2023 | Preventable deaths from respiratory diseases in children in low- and middle-income countries | Title |
|  | Simonneauet al., 2012 | Pulmonary hypertension in patients with sickle cell disease: Not so frequent but so different | TITLE |
|  | Simonson et al., 2021 | Enlarged pulmonary artery on computed tomography and respiratory failure in sickle cell disease acute chest syndrome | ABSTRACT |
|  | Singeret al., 2008 | Hypercoagulability in sickle cell disease and beta-thalassemia | TITLE |
|  | Singer et al., 2010 | The effect of thalassemia and other RBC hemolytic disorders and splenectomy on the frequency of pulmonary hypertension | TITLE |
|  | Singhet al., 2016 | When the JAK fails the pump | ABSTRACT |
|  | Singh et al., 2015 | Emerging drugs for sickle cell anemia | TITLE |
|  | Sinhaet al., 2019 | Splenectomy is not associated with a higher tricuspid regurgitant jet velocity in people with sickle cell anemia | TITLE |
|  | Sipeet al., 2020 | Hemoglobinopathies in Pregnancy | ABSTRACT |
|  | Sipe et al., 2020 | Protocols for High-Risk Pregnancies: an Evidence-Based Approach: Seventh Edition | TITLE |
|  | Smerlinget al., 2022 | Cardiac complications of sickle cell disease in pediatric patients: A case report and contemporary literature review | Title |
|  | Smith et al., 2013 | Is resistance futile? Hemodynamics in sickle cell disease | TITLE |
|  | Smith et al., 2019 | Red Blood Cell Disorders: Perioperative Considerations for Patients Undergoing Cardiac Surgery | TITLE |
|  | Smith et al., 2015 | Hemopexin and haptoglobin: allies against heme toxicity from hemoglobin not contenders | TITLE |
|  | Smith et al., 2022 | Safety of maximal cardiopulmonary exercise testing in individuals with sickle cell disease: a systematic review | TITLE |
|  | Smith et al., 2022 | Prevalence of complications among adult and pediatric Medicaid-insured patients with sickle cell disease in the United States | TITLE |
|  | Smithet al., 2014 | Evidence for the endothelin system as an emerging therapeutic target for the treatment of chronic pain | TITLE |
|  | Snyderey al., 2019 | Improving an Administrative Case Definition for Longitudinal Surveillance of Sickle Cell Disease | TITLE |
|  | Snyderet al., 2019 | Improving an Administrative Case Definition for Longitudinal Surveillance of Sickle Cell Disease | TITLE |
|  | Sohet al., 2016 | Mildly raised tricuspid regurgitant velocity 2.5–3.0 m/s in pregnant women with sickle cell disease is not associated with poor obstetric outcome – An observational cross-sectional study | TITLE |
|  | Sostin et al., 2021 | Pediatric Hemovigilance and Adverse Transfusion Reactions | ABSTRACT |
|  | Solovey et al., 2010 | Endothelial nitric oxide synthase and nitric oxide regulate endothelial tissue factor expression in vivo in the sickle transgenic mouse | Title |
|  | Solovey et al., 2013 | Interference With TNFα Using Long-Term Etanercept In S+SAntilles Sickle Transgenic Mice Ameliorates Abnormal Endothelial Activation, Vasoocclusion, and Pulmonary Hypertension Including Its Pulmonary Arterial Wall Remodeling | TITLE |
|  | Solovieffet al., 2010 | Fetal hemoglobin in sickle cell anemia: genome-wide association studies suggest a regulatory region in the 5′ olfactory receptor gene cluster | ABSTRACT |
|  | Soliman et al., 2022 | Blood transfusion and iron overload in patients with Sickle Cell Disease (SCD): Personal experience and a short update of diabetes mellitus occurrence | ABSTRACT |
|  | Spiegel et al., 2010 | Expanding the clinical spectrum of SLC29A3 gene defects | Abstract |
|  | Sobush et al., 2013 | A 2-year-old girl with co-inherited cystic fibrosis and sickle cell-β+ thalassemia presenting with recurrent vaso-occlusive events during cystic fibrosis pulmonary exacerbations: a case report | TITLE |
|  | Soh et al., 2016 | Mildly raised tricuspid regurgitant velocity 2.5-3.0 m/s in pregnant women with sickle cell disease is not associated with poor obstetric outcome - An observational cross-sectional study | TITLE |
|  | Soni et al., 2022 | Perioperative Management of Sickle Cell Disease in Complex Congenital Cardiac Surgery: A Compilation of Two Cases | ABSTRACT |
|  | Souza et al., 2007 | Quality of sleep and pulmonary function in clinically stable adolescents with sickle cell anemia | TITLE |
|  | Sonatiet al., 2008 | The genetics of blood disorders: hereditary hemoglobinopathies | ABSTRACT |
|  | Soniet al., 2014 | Outcomes of matched sibling donor hematopoietic stem cell transplantation for severe sickle cell disease with myeloablative conditioning and intermediate-dose of rabbit anti-thymocyte globulin | TITLE |
|  | Sonveauxt et al., 2007 | Transport and peripheral bioactivities of nitrogen oxides carried by red blood cell hemoglobin: Role in oxygen delivery | TITLE |
|  | Sosat al., 2018 | Sickle cell disease and pregnancy | TITLE |
|  | Souzaet al., 2006 | The need for national registries in rare diseases | TITLE |
|  | Souzaet al., 2014 | Reply: Pulmonary hypertension of sickle cell disease beyond classification constraints | TITLE |
|  | Sparkenbaughet al., 218 | High Molecular Weight Kininogen Contributes to End-Organ Damage and Mortality in a Mouse Model of Sickle Cell Disease | TITLE |
|  | Sparkenbaughet al., 2020 | High molecular weight kininogen contributes to early mortality and kidney dysfunction in a mouse model of sickle cell disease | TITLE |
|  | Spencer et al., 2020 | Chronic Thromboembolic Pulmonary Hypertension in a Child With Sickle Cell Disease | TITLE |
|  | Spring et al., 2022 | Venous Thromboembolism Is Associated with Right Sided Cardiac Dysfunction and Mortality in Patients with Sickle Cell Disease | TITLE |
|  | Spring et al., 2023 | Transthoracic Echocardiography Can Help to Predict Venous Thromboembolism and Mortality in Sickle Cell Disease | ABSTRACT |
|  | Springet al., 2022 | Venous Thromboembolism Is Associated with Right-Sided Cardiac Dysfunction and Increased Mortality in Patients with Sickle Cell Disease | ABSTRACT |
|  | Springet al., 2023 | Longitudinal Worsening of Left and Right Sided Cardiac Function in Patients with Sickle Cell Disease | Abstract |
|  | Sporns et al., 2022 | Childhood stroke | ABSTRACT |
|  | Squires et al., 2014 | Evaluation of the pediatric patient for liver transplantation: 2014 practice guideline by the american association for the study of liver diseases, american society of transplantation and the north american society for pediatric gastroenterology, hepatology and nutrition | TITLE |
|  | Starakis et al., 2007 | Endocarditis due to Salmonella enterica subsp. arizonae in a patient with sickle cell disease: A case report and review of the literature | TITLE |
|  | Sravanthi et al., 2020 | A RETROSPECTIVE STUDY OF RED CELL DISTRIBUTION WIDTH AND RIGHT VENTRICULAR SYSTOLIC PRESSURE IN PULMONARY HYPERTENSION | TITLE |
|  | Sridharan et al., 2020 | Possible effects of excipients used in the parenteral drugs administered in critically ill adults, children, and neonates | TITLE |
|  | Srinivasan et al., 2017 | Should modulation of p50 be a therapeutic target in the critically ill? | TITLE |
|  | Srivastava et al., 2018 | Hemolysis paradigm in the setting of sickle cell disease exacerbated by red blood cell transfusion | TITLE |
|  | Stamleret al., 2003 | Hemoglobin and nitric oxide | TITLE |
|  | Stanhopeet al., 2022 | Double, Triple, and Quadruple Jeopardy: Entering Pregnancy With Two or More Multimorbid Diagnoses and Increased Risk of Severe Maternal Morbidity and Postpartum Readmission | TITLE |
|  | Starr et al., 2013 | The regulation of vascular tetrahydrobiopterin bioavailability | TITLE |
|  | Stauffert al., 2021 | Nocturnal Hypoxemia Rather Than Obstructive Sleep Apnea Is Associated With Decreased Red Blood Cell Deformability and Enhanced Hemolysis in Patients With Sickle Cell Disease | TITLE |
|  | Stein et al., 2013 | Nationwide emergency department visits for priapism in the United States | TITLE |
|  | Steinberg et al., 2005 | Predicting clinical severity in sickle cell anaemia | TITLE |
|  | Steinberget al., 2006 | Pathophysiologically based drug treatment of sickle cell disease | Abstract |
|  | Steinberg et al., 2008 | Clinical trials in sickle cell disease: Adopting the combination chemotherapy paradigm | TITLE |
|  | Stabouli et al., 2020 | Unmasking hypertension in children and adolescents with sickle/beta-thalassemia | TITLE |
|  | Stack et al., 2017 | A Diagnostic Approach to Stroke in Young Adults | TITLE |
|  | Starke et al., 2006 | Prion protein in patients with renal failure | TITLE |
|  | Stasch et al., 2013 | Soluble guanylate cyclase stimulators in pulmonary hypertension | TITLE |
|  | Steinberg et al., 2008 | SNPing away at sickle cell pathophysiology | TITLE |
|  | Stram et al., 2022 | Neuropathology of Pediatric SARS-CoV-2 Infection in the Forensic Setting: Novel Application of Ex Vivo Imaging in Analysis of Brain Microvasculature | TITLE |
|  | Strauss et al., 2023 | Opening Pandora's box: abnormal genetic carrier screening and need for lifetime follow-up | TITLE |
|  | Steinberg et al., 2008 | Sickle Cell Anemia, the First Molecular Disease: Overview of Molecular Etiology, Pathophysiology, and Therapeutic Approaches | TITLE |
|  | Steinberg et al., 2009 | Genetic etiologies for phenotypic diversity in sickle cell anemia | TITLE |
|  | Steinberget al., 2006 | Modifier genes and sickle cell anemia | TITLE |
|  | Steinberg et al., 2010 | The risks and benefits of long-term use of hydroxyurea in sickle cell anemia: A 17.5 year follow-up | TITLE |
|  | Steinberg et al., 2009 | Clinical and pathophysiological aspects of sickle cell anemia | TITLE |
|  | Steinberg et al., 2012 | Genetic modifiers of sickle cell disease | TITLE |
|  | Steiner et al., 2018 | Recommendations on RBC transfusion support in children with hematologic and oncologic diagnoses from the pediatric critical care transfusion and anemia expertise initiative | TITLE |
|  | Steppanet al., 2016 | Arginase Inhibition Reverses Endothelial Dysfunction, Pulmonary Hypertension, and Vascular Stiffness in Transgenic Sickle Cell Mice | TITLE |
|  | Stojanovic et al., 2016 | Lactate dehydrogenase in sickle cell disease | TITLE |
|  | Stokes et al., 2014 | Complication rates in patients with sickle cell disease living chronically at moderate altitude | TITLE |
|  | Stoyanovaet al., 2012 | Vascular Endothelial Dysfunction in β-Thalassemia Occurs Despite Increased eNOS Expression and Preserved Vascular Smooth Muscle Cell Reactivity to NO | ABSTRACT |
|  | Strijbos et al., 2009 | Circulating endothelial cells: A potential parameter of organ damage in sickle cell anemia? | ABSTRACT |
|  | Stroumsa et al., 2023 | Pregnancy Outcomes in a US Cohort of Transgender People | ABSTRACT |
|  | Strouse et al., 2009 | Low levels of apolipoprotein a1 are associated with silent cerebral infarcts in children with sickle cell disease | Title |
|  | Stuart et al., 2004 | Sickle-cell disease | TITLE |
|  | Starosta et al., 2022 | Infantile-onset Pompe disease complicated by sickle cell anemia: Case report and management considerations | TITLE |
|  | Stöllbergeret al., 2015 | Does professional athlete training cause acquired left ventricular hypertrabeculation/noncompaction? | TITLE |
|  | Stollings et al., 2006 | Oxygen therapeutics: Oxygen delivery without blood | TITLE |
|  | Stolmeijer et al., 2015 | Interplay of co-inherited diseases can turn benign syndromes in a deadly combination: Haemoglobinopathy and bilirubin transport disorder | TITLE |
|  | Summers et al., 2003 | Prenatal diagnosis for paediatricians | TITLE |
|  | Syamasundar Rao et al., 2020 | The author’s contributions to echocardiography literature (Part i—1978–1990)† | TITLE |
|  | Stubbset al., 2022 | Severe Pediatric COVID-19 Pneumonia Treated With Adjuvant Anakinra | TITLE |
|  | Styles et al., 2008 | Nitric Oxide Effects in Sickle Cell Disease | TITLE |
|  | Styles et al., 2000 | Evidence for HLA-related susceptibility for stroke in children with sickle cell disease | TITLE |
|  | Suárez et al., 2023 | Clinical and demographic characteristics of sickle cell disease in Colombian children | ABSTRACT |
|  | Suellt al., 2005 | Increased pulmonary artery pressures among adolescents with sickle cell disease | ABSTRACT |
|  | Sullivan et al., 2008 | Nitric oxide metabolism and the acute chest syndrome of sickle cell anemia | ABSTRACT |
|  | Sullivan et al., 2010 | Effect of Oral Arginine Supplementation on Exhaled Nitric Oxide Concentration in Sickle Cell Anemia and Acute Chest Syndrome | TITLE |
|  | Sun et al., 2017 | Purinergic control of red blood cell metabolism: Novel strategies to improve red cell storage quality | ABSTRACT |
|  | Sun et al., 2017 | Drug discovery and development for rare genetic disorders | TITLE |
|  | Sun et al., 2023 | Acute Events Significantly Increase Risk for Long-Term End-Organ Sequelae in Sickle Cell Disease - a Retrospective Cohort Study Using Optum Electronic Health Records | TITLE |
|  | Sundaram et al., 2011 | Biomarkers for early detection of sickle nephropathy | TITLE |
|  | Sundaramet al., 2010 | High levels of placenta growth factor in sickle cell disease promote pulmonary hypertension | TITLE |
|  | Sundararajan et al., 2012 | Sickle cell disease does not decrease pulmonary nitric oxide | Title |
|  | Sundd et al., 2019 | Pathophysiology of Sickle Cell Disease | TITLE |
|  | Svarch et al., 2009 | Physiopathologic features of drepanocytemia | TITLE |
|  | Syed et al., 2023 | THE DEVIL IS IN THE DETAILS -MCCONNELL'S SIGN IN A SICKLE CELL PATIENT PRESENTING WITH ACUTE CHEST SYNDROME | TITLE |
|  | Syverson et al., 2016 | Adolescents' Perceptions of Transition Importance, Readiness, and Likelihood of Future Success: The Role of Anticipatory Guidance | TITLE |
|  | Sylvester et al., 2006 | Computed tomography and pulmonary function abnormalities in sickle cell disease | TITLE |
|  | Sylvester et al., 2004 | Pulmonary function abnormalities in children with sickle cell disease | TITLE |
|  | Szaboet al., 2010 | Gaseotransmitters: New frontiers for translational science | ABSTRACT |
|  | Tabarroki al., 2014 | Nitric Oxide As a Mediator of Bone Marrow Fibrosis in Patients with Myelofibrosis | TITLE |
|  | Taggartet al., 2016 | Shortness of breath: Looking beyond the usual suspects | TITLE |
|  | Takatsukiet al., 2012 | Correlation of N-Terminal Fragment of B-type Natriuretic Peptide Levels with Clinical, Laboratory, and Echocardiographic Abnormalities in Children with Sickle Cell Disease | TITLE |
|  | Taksande et al., 2021 | Variation in pulmonary function tests among children with sickle cell anemia: a systematic review and meta-analysis | TITLE |
|  | Tajmalzai et al., 2021 | Early manifestation of Moyamoya syndrome in a 2-year-old child with Down syndrome | TITLE |
|  | Talahma et al., 2014 | Sickle cell disease and stroke | TITLE |
|  | Tan et al., 2016 | Original Research: Diametric effects of hypoxia on pathophysiology of sickle cell disease in a murine model | TITLE |
|  | Tanenbaum et al., 2021 | The Use of Extracorporeal Membrane Oxygenation During Acute Chest Crisis in an Adult Sickle Cell Disease Patient: Identification of Pearls and Pitfalls | TITLE |
|  | Tanriverdi et al., 2021 | The efficacy of voxelotor, 900 mg in patients with sickle cell anaemia: A meta-analysis of the randomised controlled trials | TITLE |
|  | Tantawyet al., 2016 | Genotype in children and adolescents with sickle cell disease: Relation to vascular complications and subclinical atherosclerosis | TITLE |
|  | Tantawyet al., 2013 | Endothelial nitric oxide synthase gene introne4 VNTR polymorphism in sickle cell disease and transfusion-dependent b-Thalassemia major: Relation to cardio-vascular complications | TITLE |
|  | Tantawyet al., 2015 | Soluble FMS-like tyrosine kinase-1 in children and adolescents with thalassemia intermedia: Relation to pulmonary vasculopathy and subclinical atherosclerosis | Title |
|  | Tantawyet al., 2023 | Sleep disordered breathing and its relation to stroke and pulmonary hypertension in children with sickle cell disease: a single-center cross-sectional study | ABSTRACT |
|  | Tantawyet al., 2014 | Growth differentiation factor-15 in young sickle cell disease patients: relation to hemolysis, iron overload and vascular complications | ABSTRACT |
|  | Tantawy et al., 2013 | Circulating platelet and erythrocyte microparticles in young children and adolescents with sickle cell disease: Relation to cardiovascular complications | ABSTRACT |
|  | Tantawyet al., 2016 | Genotype/phenotype pattern in Egyptian patients with sickle cell disease | TITLE |
|  | Tantawy et al., 2020 | Cardiac fibrosis in patients with sickle cell anemia using MRI T1 cardiac mapping | TITLE |
|  | Tantawy et al., 2017 | Clinical Predictive Value of Cystatin C in Pediatric Sickle Cell Disease: A Marker of Disease Severity and Subclinical Cardiovascular Dysfunction | TITLE |
|  | Tantawy et al., 2015 | Endothelial nitric oxide synthase gene intron 4 VNTR polymorphism in sickle cell disease: Relation to vasculopathy and disease severity | ABSTRACT |
|  | Tasselet al., 2011 | Correlations between lung, cardiac functions and hematologic parameters in SCA-chidren | TITLE |
|  | Taylor et al., 2006 | Mutations and Polymorphisms Influencing Hemolysis in Hemoglobin Genes and Risk of Pulmonary Hypertension in Sickle Cell Disease: Effect of Hemoglobin SC | TITLE |
|  | Tackett et al., 2019 | Preliminary exploration of secondhand smoke exposure in youth with Sickle Cell Disease: biochemical verification, pulmonary functioning, and health care utilization | TITLE |
|  | Taksande et al., 2005 | Left ventricular systolic and diastolic functions in patients with sickle cell anemia | TITLE |
|  | Tang et al., 2015 | Pulmonary Embolism and Infarct After Bilateral Cochlear Implantation in a Patient with Newly Diagnosed Sickle/β+ Thalassemia | ABSTRACT |
|  | Tani et al., 2009 | Recent progressions in stem cell research: breakthroughs achieved and challenges faced | Title |
|  | Tannor et al., 2022 | The Impact of Low Socioeconomic Status on Progression of Chronic Kidney Disease in Low- and Lower Middle-Income Countries | TITLE |
|  | Taylor et al., 2009 | A GCH1 haplotype associated with susceptibility to vasoocclusive pain and impaired vascular function in sickle cell anemia | TITLE |
|  | Taylor et al., 2022 | Axillary intra-aortic balloon pump, biventricular assist device implantation and subsequent orthotopic heart transplantation in a patient with sickle cell trait | ABSTRACT |
|  | Taylor et al., 2006 | The hyperhemolysis phenotype in sickle cell anemia: Increased risk of leg ulcers, priapism, pulmonary hypertension and death with decreased risk of vasoocclusive events | TITLE |
|  | Taylor et al., 2008 | Chronic Hyper-Hemolysis in Sickle Cell Anemia: Association of Vascular Complications and Mortality with Less Frequent Vasoocclusive Pain | ABSTRACT |
|  | Taylor et al., 2008 | Mutations and polymorphisms in hemoglobin genes and the risk of pulmonary hypertension and death in sickle cell disease | ABSTRACT |
|  | Taylor Vi et al., 2008 | Severe pulmonary hypertension in an adolescent with sickle cell disease | ABSTRACT |
|  | Tayo et al., 2017 | Co-inheritance of apol1 risk variants and polymorphisms of the BCL11A, G6PD, HBA1 and HBA2 genes in sickle cell anemia patients from Nigeria | ABSTRACT |
|  | Tantawy et al., 2019 | Surfactant protein D as a marker for pulmonary complications in pediatric patients with sickle cell disease: Relation to lung function tests | ABSTRACT |
|  | Tariq et al., 2016 | An unfortunate case of acquired hemochromatosis: a case report review of the clinical presentation, diagnosis, management, and prognosis | TITLE |
|  | Tassel et al., 2011 | Leukocytosis is a risk factor for lung function deterioration in children with sickle cell disease | TITLE |
|  | Terroni et al., 2022 | Vascular Effects of the Fetal Hemoglobin Inducer Agent 3-(1,3-Dioxoisoindolin-2-yl) Benzyl Nitrate | ABSTRACT |
|  | Tetsuhara et al., 2016 | Pediatric Thromboembolism in Japan | TITLE |
|  | Thomas et al., 2022 | Pediatric hypertension: Review of the definition, diagnosis, and initial management | TITLE |
|  | Terry et al., 2019 | Imatinib-induced pericardial effusion in a child | TITLE |
|  | Tewari et al., 2015 | Environmental determinants of severity in sickle cell disease | TITLE |
|  | Teixeira de Barros et al., 2007 | Therapeutic profile of orphan medicines | TITLE |
|  | Telen et al., 2008 | It really IS the red cell | TITLE |
|  | Telen et al., 2015 | Biomarkers and recent advances in the management and therapy of sickle cell disease | TITLE |
|  | Telenet al., 2023 | Biologic Assessment of RBC Biology and Neutrophil Activation: Correlation with Sickle Cell Disease Activity | TITLE |
|  | Tennenbaum et al., 2023 | Splenic dysfunction in sickle cell disease: An update | TITLE |
|  | Tennenbaum et al., 2023 | Increased risk of venous thromboembolism in splenectomized patients with sickle cell disease | TITLE |
|  | Terrieet al., 2010 | Pulmonary arterial hypertension: Keeping patients informed | TITLE |
|  | Teulieret al., 2021 | Severe COVID-19 with acute respiratory distress syndrome (ARDS) in a sickle cell disease adult patient: case report | TITLE |
|  | Thachil et al., 2009 | The enigma of pulmonary hypertension after splenectomy - Does the megakaryocyte provide a clue? | TITLE |
|  | Thachil et al., 2010 | Reticulocytes and erythroid precursors in haemolysis vasculopathy | TITLE |
|  | Theinet al., 2017 | Sickle cell disease in the older adult | TITLE |
|  | Thein et al., 2008 | Genetic modifiers of the β-haemoglobinopathies | TITLE |
|  | Thein et al., 2011 | Genetic modifiers of sickle cell disease | TITLE |
|  | Thambiayya et al., 2012 | Functional role of intracellular labile zinc in pulmonary endothelium | TITLE |
|  | Thatcheret al., 2005 | An introduction to NO-related therapeutic agents | TITLE |
|  | Then et al., 2018 | Children as haematopoietic stem cell donors: Ethically challenging and legally complex | TITLE |
|  | Thompsonet al., 2006 | Advances in the management of sickle cell disease | Title |
|  | Thompson et al., 2019 | Lentiglobin gene therapy in patients with sickle cell disease: Updated interim results from HGB-206 | TITLE |
|  | Tian et al., 2019 | Targeting LOX-1 in atherosclerosis and vasculopathy: current knowledge and future perspectives | TITLE |
|  | Timofeevet al., 2009 | Genetic ancestry of sickle cell anemia patients | TITLE |
|  | Timon et al., 2021 | Fat embolism syndrome – A qualitative review of its incidence, presentation, pathogenesis and management | TITLE |
|  | Tino et al., 2007 | Clinical year in review I: Lung cancer, interventional pulmonology, pediatric pulmonary disease, and pulmonary vascular disease | TITLE |
|  | Tidake et al., 2015 | Left ventricular function by echocardiogram in children with sickle cell anaemia in Mumbai, Western India | TITLE |
|  | Tinti et al., 2010 | Benefits of kinesiotherapy and aquatic rehabilitation on sickle cell anemia. A case report | ABSTRACT |
|  | Tipaneeet al., 2017 | Preclinical and clinical advances in transposon-based gene therapy | TITLE |
|  | Tisdaleet al., 2018 | Single-agent plerixafor mobilization to collect autologous stem cells for use in gene therapy for severe sickle cell disease | TITLE |
|  | Tisdale et al., 2018 | Current Results of Lentiglobin Gene Therapy in Patients with Severe Sickle Cell Disease Treated Under a Refined Protocol in the Phase 1 Hgb-206 Study | TITLE |
|  | Tisdale et al., 2017 | Successful plerixafor-mediated mobilization, apheresis, and lentiviral vector transduction of hematopoietic stem cells in patients with severe sickle cell disease | TITLE |
|  | Tisdaleet al., 2021 | Polyclonality Strongly Correlates with Biological Outcomes and Is Significantly Increased Following Improvements to the Phase 1/2 HGB-206 Protocol and Manufacturing of LentiGlobin for Sickle Cell Disease (SCD; bb1111) Gene Therapy (GT) | TITLE |
|  | Tissot et al., 2010 | Medical therapy for pediatric pulmonary arterial hypertension | TITLE |
|  | Tiwariet al., 2013 | Pathophysiological consequences of hemolysis in sickle cell disease | TITLE |
|  | Tobin et al., 2003 | Chronic obstructive pulmonary disease, pollution, pulmonary vascular disease, transplantation, pleural disease, and lung cancer in AJRCCM 2002 | TITLE |
|  | Tofovicet al., 2009 | Adenosine deaminase-adenosine pathway in hemolysis-associated pulmonary hypertension | TITLE |
|  | Toledoet al., 2019 | Sickle cell disease: Hemostatic and inflammatory changes, and their interrelation | TITLE |
|  | Tolu et al., 2019 | High Hemoglobin F in Sickle Cell Disease: Waning Protection with Age | TITLE |
|  | Tolu et al., 2020 | Hemoglobin F mitigation of sickle cell complications decreases with aging | TITLE |
|  | Tomeret al., 2001 | Reduction of pain episodes and prothrombotic activity in sickle cell disease by dietary n-3 fatty acids | TITLE |
|  | Tomilleroet al., 2009 | Gateways to clinical trials | TITLE |
|  | Tolba et al., 2017 | Speckle tracking evaluation of right ventricular functions in children with sickle cell disease | TITLE |
|  | Tolan Jr et al., 2010 | Fever of unknown origin: A diagnostic approach to this vexing problem | TITLE |
|  | Tonino et al., 2013 | Chest pain in sickle cell disease | TITLE |
|  | Toorani et al., 2018 | A rare concurrence of hodgkin’s lymphoma, sickle cell disease and diabetes mellitus | TITLE |
|  | Tomaszewski et al., 2012 | Cardiomegaly in tropical Africa | TITLE |
|  | Tonguç et al., 2022 | Investigation of the relationship between periodontal and systemic inflammation in children with Sickle Cell Disease: A case- control study | TITLE |
|  | Torres Filho et al., 2014 | Perfluorocarbon emulsion improves oxygen transport of normal and sickle cell human blood in vitro | TITLE |
|  | Tournamille et al., 2010 | Partial C antigen in sickle cell disease patients: clinical relevance and prevention of alloimmunization | TITLE |
|  | Toyoda et al., 2021 | Spontaneous splenic rupture, mesenteric ischemia and spinal infarction after aortic repair for acute type A dissection in a patient with sickle cell trait | TITLE |
|  | Topcuoglu et al., 2010 | Advances in transcranial Doppler clinical applications | TITLE |
|  | Toprak et al., 2015 | The pathology of lethal exposure to the Riot Control Agents: Towards a forensics-based methodology for determining misuse | TITLE |
|  | Torjesen et al., 2016 | Healthy people need fewer preoperative tests for elective surgery, says nice | TITLE |
|  | Tormene et al., 2006 | Thrombosis and thrombophilia in children: A systematic review | TITLE |
|  | Towerman et al., 2023 | Hyperammonemia and acute liver failure associated with deferasirox in two adolescents with sickle cell disease | TITLE |
|  | Tommasi et al., 2011 | Marked increase in etravirine and saquinavir plasma concentrations during atovaquone/proguanil prophylaxis | TITLE |
|  | Tostaet al., 2009 | Maternal morbidity in pregnant women with sickle cell disease | TITLE |
|  | Tostat al., 2009 | Perinatal and maternal outcomes in pregnant women with sickle cell disease and sickle cell trait | TITLE |
|  | Touzotet al., 2012 | Cytokine Environement Analysis During Allogeneic Hematopoietic Stem Cell Transplantation for Inherited Diseases | TITLE |
|  | Tripathi et al., 2011 | Clinical complications in severe pediatric sickle cell disease and the impact of hydroxyurea | TITLE |
|  | Trow et al., 2005 | Clinical Year in Review I: Lung cancer, interventional pulmonology, noninvasive mask ventilation, and pulmonary vascular disease | Abstract |
|  | Tran et al., 2007 | Peripheral retinal neovascularization in talc retinopathy | TITLE |
|  | Truonget al., 2022 | Inferior STEMI Electrocardiogram in a Young Postpartum Female with Sickle Cell Trait with Chest Pain - A Case Report | TITLE |
|  | Tsou et al., 2021 | The burden of obstructive sleep apnea in pediatric sickle cell disease: a Kids' inpatient database study | TITLE |
|  | Tsironiet al., 2005 | The heart in sickle cell disease | TITLE |
|  | Tsironi et al., 2005 | Pulmonary function and pulmonary hypertension in sickle cell disease | TITLE |
|  | Tsitsikas et al., 2021 | Automated red cell exchange in the management of sickle cell disease | ABSTRACT |
|  | Tsitsikaset al., 2017 | Distinct patterns of response to transfusion therapy for different chronic complications of sickle cell disease: A useful insight | TITLE |
|  | Tsitsikaset al., 2014 | Regular automated red cell exchange transfusion in the management of pulmonary hypertension in sickle cell disease | TITLE |
|  | Tsitsikas et al., 2016 | Safety, tolerability, and outcomes of regular automated red cell exchange transfusion in the management of sickle cell disease | ABSTRACT |
|  | Tsang et al., 2022 | 17-year-old boy with syncope and cardiac arrest | TITLE |
|  | Tyc et al., 2006 | Smoking rates and the state of smoking interventions for children and adolescents with chronic illness | TITLE |
|  | Tziomalos et al., 2005 | Mitral valve reconstruction in a compound heterozygote for sickle cell anemia and hemoglobin Lepore | TITLE |
|  | Patel et al., 2020 | Pneumothorax and pneumomediastinum secondary to COVID-19 disease unrelated to mechanical ventilation | TITLE |
|  | Turpinet al., 2018 | Chronic blood exchange transfusions in the management of pre-capillary pulmonary hypertension complicating sickle cell disease | TITLE |
|  | Turner et al., 2009 | Exchange versus simple transfusion for acute chest syndrome in sickle cell anemia adults | TITLE |
|  | Ubuane et al., 2022 | Reference values and equations for the 6-minute walk distance of Nigerian children aged 6–11 years: A cross-sectional study | TITLE |
|  | Uddinet al., 2023 | Clinical outcomes and 30-day readmissions associated with high-output heart failure | TITLE |
|  | Udezeet al., 2023 | Economic and Clinical Burden of Managing Sickle Cell Disease with Recurrent Vaso-Occlusive Crises in the United States | Title |
|  | Udeze et al., 2022 | PROJECTED LIFETIME ECONOMIC BURDEN OF SEVERE SICKLE CELL DISEASE IN THE UNITED STATES | TITLE |
|  | Underwood et al., 2005 | Nursing contributions to the elimination of health disparities among African-Americans: review and critique of a decade of research--Part III | TITLE |
|  | Unal et al., 2015 | Fanconis anemia effect or sickle cell anemia effect: That is the question | TITLE |
|  | Uppuluri et al., 2021 | Risk factors for central retinal artery occlusion in young patients | TITLE |
|  | Upadhya et al., 2013 | Prolongation of QTc intervals and risk of death among patients with sickle cell disease | TITLE |
|  | Usman et al., 2010 | Warm, beating heart aortic valve replacement in a sickle cell patient | TITLE |
|  | Ussia et al., 2009 | Percutaneous closure of patent foramen ovale with a bioabsorbable occluder device: single-centre experience | TITLE |
|  | Uk Kanget al., 2019 | Association of hyperuricemia and pulmonary hypertension: A systematic review and meta-analysis | TITLE |
|  | Ukachiet al., 2017 | Hydroxyurea improves FEV1/FVC in children with sickle cell anemia | TITLE |
|  | Ukejet al., 2021 | The Burden of Atrial Fibrillation in Sickle Cell Disease | TITLE |
|  | Unalt al., 2015 | Fanconi's Anemia Effect or Sickle Cell Anemia Effect: That is the Question | TITLE |
|  | Unalet al., 2021 | Chronic complications of sickle cell disease patients in Turkey | TITLE |
|  | Upadhya et al., 2014 | Echocardiography-derived tricuspid regurgitant jet velocity is an important marker for the progression of sickle-cell disease | ABSTRACT |
|  | Uppaluri et al., 2014 | A retrospective analysis of the prevalence of restrictive pulmonary disease in pediatric patients with sickle cell anemia | ABSTRACT |
|  | Urbinati et al., 2006 | Pathophysiology and therapy for haemoglobinopathies. Part II: Thalassaemias | TITLE |
|  | Usmani et al., 2018 | Vascular complications of sickle cell disease | TITLE |
|  | Uzet al., 2016 | Methodological Contributions about Systolic Pulmonary Artery Pressure | ABSTRACT |
|  | Vaidyaet al., 2015 | A sickle cell disease patient with severe tricuspid regurgitation and early developed pulmonary hypertension | TITLE |
|  | van Beemet al., 2009 | Elevated endothelial progenitor cells during painful sickle cell crisis | Title |
|  | Van Beers et al., 2010 | Letter in response to: "Pulmonary thrombi are not detected by 3D magnetic resonance angiography in adults with sickle cell anemia and an elevated triscuspid regurgitant jet velocity" | TITLE |
|  | VanderJagt et al., 2002 | Hypocholesterolemia in Nigerian children with sickle cell disease | TITLE |
|  | Valchanov et al., 2008 | Preoperative investigation of the surgical patient | TITLE |
|  | Van Westerloo et al., 2013 | A little fat every day keeps the doctor away | TITLE |
|  | Vassilopoulou et al., 2011 | Cerebral venous sinus thrombosis in an adult with sickle β °-thalassemia | TITLE |
|  | VanderJagt et al., 2008 | Pulmonary function correlates with body composition in Nigerian children and young adults with sickle cell disease | TITLE |
|  | Veras et al., 2005 | Plastic bronchitis in a child with thalassemia alpha | ABSTRACT |
|  | Van Beers et al., 2009 | Lung function tests in patients with sickle cell disease: A reply | TITLE |
|  | Van Beers et al., 2008 | No association of the hypercoagulable state with sickle cell disease related pulmonary hypertension | TITLE |
|  | van Beerset al., 2014 | Exercise tolerance, lung function abnormalities, anemia, and cardiothoracic ratio in sickle cell patients | ABSTRACT |
|  | Van Beers et al., 2008 | Large and medium-sized pulmonary artery obstruction does not play a role of primary importance in the etiology of sickle-cell disease-associated pulmonary hypertension | ABSTRACT |
|  | Van Beers et al., 2008 | Cardiopulmonary imaging, functional and laboratory studies in sickle cell disease associated pulmonary hypertension | ABSTRACT |
|  | Vlachou et al., 2021 | Biochemical and imaging markers in patients with thalassaemia | Abstract |
|  | van Beerset al., 2008 | Sickle cell disease-related organ damage occurs irrespective of pain rate: implications for clinical practice | Title |
|  | Van Der Land et al., 2013 | Markers of endothelial dysfunction differ between subphenotypes in children with sickle cell disease | TITLE |
|  | Van Der Plas et al., 2011 | Mortality and causes of death in children with sickle cell disease in the Netherlands, before the introduction of neonatal screening | ABSTRACT |
|  | van Dijk et al., 2023 | One-year safety and efficacy of mitapivat in sickle cell disease: follow-up results of a phase 2, open-label study | ABSTRACT |
|  | Van et al., 2010 | Low Mortality In Sickle Cell Patient Cohort with Elevated Tricuspid Regurgitant Jet Flow Velocity | Title |
|  | Vanet al., 2006 | Systematic Evaluation of Sickle Cell-Related Organ Damage and Complications: Implications for Sickle Cell Disease Management | ABSTRACT |
|  | van Hamel Parsons et al., 2016 | Venous thromboembolism in adults with sickle cell disease: experience of a single centre in the UK | Abstract |
|  | Van Hook et al., 2022 | McConnell's Sign in the Setting of Pulmonary Contusion | Abstract |
|  | Van Rensburg et al., 2016 | Paediatric cardiac anaesthesia in sickle cell disease: A case series | TITLE |
|  | Van Tuijnet al., 2010 | Chronic organ damage is not related to pregnancy complications in sickle cell disease | TITLE |
|  | Van Tuijnet al., 2013 | Systematic evaluation of chronic organ damage in adult sickle cell patients. A seven-year follow-up study | TITLE |
|  | van Tuijnet al., 2017 | Prospective evaluation of chronic organ damage in adult sickle cell patients: A seven-year follow-up study | ABSTRACT |
|  | Van Tuijn et al., 2009 | Pain rate and social circumstances rather than cumulative organ damage determine the quality of life in adults with sickle cell disease | ABSTRACT |
|  | Van Tuijn et al., 2010 | Pregnancy complications in sickle cell disease are more prevalent in women with vaso-occlusion related organ damage than hemolysis related organ damage | ABSTRACT |
|  | van Vuren et al., 2021 | Lactate dehydrogenase to carboxyhemoglobin ratio as a biomarker of heme release to heme processing is associated with higher tricuspid regurgitant jet velocity and early death in sickle cell disease | TITLE |
|  | Van Vurenet al., 2021 | A Unique Monocyte Transcriptome Discriminates Sickle Cell Disease From Other Hereditary Hemolytic Anemias and Shows the Particular Importance of Lipid and Interferon Signaling | ABSTRACT |
|  | Vandergraesenet al., 2016 | Successful Transition from Paediatric to Adult Care in Sickle Cell Disease: An Experience in a Belgian Center | TITLE |
|  | Vanderpool et al., 2018 | Hematocrit-corrected pulmonary vascular resistance | ABSTRACT |
|  | Vasconceloset al., 2015 | Left ventricular remodeling in patients with sickle cell disease: determinants factors and impact on outcome | TITLE |
|  | Veilet al., 2019 | Trajectories of biological values and vital parameters: An observational cohort study of adult patients with sickle cell disease hospitalized for a non-complicated vaso-occlusive crisis | ABSTRACT |
|  | Vekilovet al., 2004 | Nucleation of polymers of sickle cell anemia hemoglobin | ABSTRACT |
|  | Vendraminiet al., 2006 | Lung function and airway hyperresponsiveness in adult patients with sickle cell disease | TITLE |
|  | Verlhac et al., 2011 | Transcranial Doppler in children | TITLE |
|  | Vercellotti et al., 2007 | The "NO" tipping point | TITLE |
|  | Verissimoet al., 2015 | Comment on sickle cell disease and left ventricular hypertrophy | TITLE |
|  | Vermeulen Windsant et al., 2011 | Cardiovascular surgery and organ damage: Time to reconsider the role of hemolysis | TITLE |
|  | Vichinsky et al., 2007 | Hematology / the Education Program of the American Society of Hematology. American Society of Hematology. Education Program | TITLE |
|  | Vicariet al., 2015 | Interleukin-1β and interleukin-6 gene polymorphisms are associated with manifestations of sickle cell anemia | TITLE |
|  | Vicariet al., 2005 | Echocardiographic abnormalities in Brazilian sickle cell patients | TITLE |
|  | Vianello et al., 2014 | Arginine butyrate per os protects mdx mice against cardiomyopathy, kyphosis and changes in axonal excitability | ABSTRACT |
|  | Vieira et al., 2016 | Pulmonary function in children and adolescents with sickle cell disease: have we paid proper attention to this problem? | TITLE |
|  | Vinchiet al., 2013 | Hemopexin therapy improves cardiovascular function by preventing heme-induced endothelial toxicity in mouse models of hemolytic diseases | ABSTRACT |
|  | Vicari et al., 2007 | Procoagulant Activity in Sickle Cell Anemia | TITLE |
|  | Vichinsky et al., 2007 | Hemoglobin e syndromes | TITLE |
|  | Vichinskyet al., 2012 | Emerging 'A' therapies in hemoglobinopathies: agonists, antagonists, antioxidants, and arginine | TITLE |
|  | Vichinskyet al., 2018 | Results from Part A of the Hemoglobin Oxygen Affinity Modulation to Inhibit HbS Polymerization (HOPE) Trial (GBT440-031), a Placebo-Controlled Randomized Study Evaluating Voxelotor (GBT440) in Adults and Adolescents with Sickle Cell Disease | TITLE |
|  | Vichinskyet al., 2011 | A randomized phase II study evaluating the efficacy and safety of deferasirox versus deferoxamine in patients with sickle cell disease (SCD): 2-year results including pharmacokinetics (PK) and safety of deferasirox with concomitant hydroxyurea therapy | TITLE |
|  | Vichinsky et al., 2004 | Pulmonary Hypertension in Sickle Cell Disease | TITLE |
|  | Vichinskyt al., 2010 | Medical management of sickle cell disease | ABSTRACT |
|  | Vichinsky et al., 2010 | Preface to Cooley's anemia: Ninth symposium | TITLE |
|  | Vichinskyet al., 2011 | Approaches to transfusion therapy and iron overload in patients with sickle cell disease: Results of an international survey | TITLE |
|  | Victor et al., 2016 | Echocardiographic measures of pulmonary hypertension and the prediction of end-points in sickle cell disease | TITLE |
|  | Machadot al., 2010 | Pulmonary complications of hemoglobinopathies | Abstract |
|  | Vilas-Boas et al., 2010 | Arginase levels and their association with Th17-related cytokines, soluble adhesion molecules (sICAM-1 and sVCAM-1) and hemolysis markers among steady-state sickle cell anemia patients | ABSTRACT |
|  | Villagraet al., 2007 | Platelet activation in patients with sickle disease, hemolysis-associated pulmonary hypertension, and nitric oxide scavenging by cell-free hemoglobin | ABSTRACT |
|  | Villamizaret al., 2023 | MACITENTAN IN ADULTS WITH SICKLE CELL DISEASE AND PULMONARY HYPERTENSION | ABSTRACT |
|  | Villavicencioet al., 2008 | Symptomatic Pulmonary Hypertension in a Child with Sickle Cell Disease | TITLE |
|  | Villerset al., 2008 | Morbidity associated with sickle cell disease in pregnancy | ABSTRACT |
|  | Vinchi et al., 2013 | Therapeutic Approaches to Limit Hemolysis-Driven Endothelial Dysfunction: Scavenging Free Heme to Preserve Vasculature Homeostasis | TITLE |
|  | Vinhaes et al., 2020 | Hydroxyurea treatment is associated with reduced degree of oxidative perturbation in children and adolescents with sickle cell anemia | TITLE |
|  | Vives Corronset al., 2020 | Rare anaemias, sickle-cell disease and covid-19 | ABSTRACT |
|  | Vlachopoulost al., 2009 | Cardiovascular effects of phosphodiesterase type 5 inhibitors | TITLE |
|  | Vlachopouloset al., 2009 | PDE5 inhibitors in non-urological conditions | TITLE |
|  | Vo et al., 2016 | Streptococcus pneumoniae meningitis complicated by an intramedullary abscess: A case report and review of the literature | TITLE |
|  | Vobugari et al., 2022 | Sideroblastic anaemia in a patient with sickle cell disease | TITLE |
|  | Vocelkaet al., 2001 | Cardiopulmonary bypass with deep hypothermic circulatory arrest for a patient with sickle cell anemia: a case report | TITLE |
|  | Von Ungern-Sternberget al., 2007 | Pediatric anesthesia - Potential risks and their assessment: Part I | TITLE |
|  | Vonaet al., 2021 | Sickle Cell Disease: Role of Oxidative Stress and Antioxidant Therapy | TITLE |
|  | Voskaridou et al., 2010 | The effect of prolonged administration of hydroxyurea on morbidity and mortality in adult patients with sickle cell syndromes: results of a 17-year, single-center trial (LaSHS) | TITLE |
|  | Voskaridouet al., 2012 | Circulating activin-a is elevated in patients with thalassemia major and double heterozygous sickle-cell/beta-thalassemia and correlates with markers of hemolysis and bone mineral density | ABSTRACT |
|  | Voskaridou et al., 2012 | Sickle-cell disease and the heart: Review of the current literature | Abstract |
|  | Vos et al., 2017 | Global, regional, and national incidence, prevalence, and years lived with disability for 328 diseases and injuries for 195 countries, 1990-2016: A systematic analysis for the Global Burden of Disease Study 2016 | TITLE |
|  | Voskaridou et al., 2007 | Pulmonary hypertension in patients with sickle cell/beta thalassemia: incidence and correlation with serum N-terminal pro-brain natriuretic peptide concentrations |  |
|  | Vuottoet al., 2008 | Pseudoxanthoma elasticum-like syndrome associated with hemoglobinopathy: a case report | ABSTRACT |
|  | Voskaridou et al., 2006 | The serum levels of N-terminal pro B-Type natriuretic peptide (NT-proBNP) is a strong indicator of pulmonary hypertension in patients with sickle Cell/Beta thalassemia | TITLE |
|  | Vuonget al., 2021 | Does the ‘halo effect’ of trauma center verification extend to severe postpartum hemorrhage? A four-year retrospective review of level 1 trauma centers in the United States | TITLE |
|  | Wagdy et al., 2018 | Subclinical myocardial injury during vaso-occlusive crisis in pediatric sickle cell disease | TITLE |
|  | Waggass et al., 2023 | The Prevalence of Cardiovascular Manifestations in Pediatric Sickle Cell Anemia Patients in a Large Tertiary Care Hospital in the Western Region of Saudi Arabia | TITLE |
|  | Wahl et al., 2009 | Current issues in blood transfusion for sickle cell disease | ABSTRACT |
|  | Wahlt al., 2010 | Pulmonary hypertension in hemolytic anemias | TITLE |
|  | Wali et al., 2000 | Cardiovascular function in Omani children with sickle cell anaemia | ABSTRACT |
|  | Wallaceet al., 2014 | Children with sickle cell disease on chronic red cell transfusion experience fewer hospitalizations for acute vaso-occlusive episodes irrespective of the indication for transfusion | ABSTRACT |
|  | Walteret al., 2015 | The effects of glutamine supplementation on markers of autophagy and apoptosis in peripheral blood mononuclear cells from patients with sickle cell disease | ABSTRACT |
|  | Walter et al., 2022 | The effects of glutamine supplementation on markers of apoptosis and autophagy in sickle cell disease peripheral blood mononuclear cells | TITLE |
|  | Walterst al., 2016 | Indications and Results of HLA-Identical Sibling Hematopoietic Cell Transplantation for Sickle Cell Disease | TITLE |
|  | Wamkpahet al., 2022 | Renin-Angiotensin Blockade Reduces Readmission for Acute Chest Syndrome in Sickle Cell Disease | Title |
|  | Wagdy et al., 2023 | Evaluation of cardiac fibrosis and subclinical cardiac changes in children with sickle cell disease using magnetic resonance imaging, echocardiography, and serum galectin-3 | TITLE |
|  | Wallace et al., 2010 | Adenosine A2A receptors induced on iNKT and NK cells reduce pulmonary inflammation and injury in mice with sickle cell disease | TITLE |
|  | Walters et al., 2010 | Pulmonary, gonadal, and central nervous system status after bone marrow transplantation for sickle cell disease | TITLE |
|  | Wadhwa et al., 2020 | Knowledge about obstructive sleep apnea among medical undergraduate students: A long way to go! | ABSTRACT |
|  | Wang et al., 2004 | Acute myocardial infarction in hemoglobin SC disease | TITLE |
|  | Ware et al., 2017 | Technological Advances in Sickle Cell Disease | TITLE |
|  | Walters et al., 2000 | Impact of bone marrow transplantation for symptomatic sickle cell disease: an interim report. Multicenter investigation of bone marrow transplantation for sickle cell disease | TITLE |
|  | Waltz et al., 2013 | Hematological and hemorheological determinants of the six-minute walk test performance in children with sickle cell anemia | TITLE |
|  | Wang et al., 2023 | Mechanism Research of Platelet Core Marker Prediction and Molecular Recognition in Cardiovascular Events | TITLE |
|  | Wandereret al., 2009 | Rationale for IL-1β targeted therapy for ischemia-reperfusion induced pulmonary and other complications in sickle cell disease | TITLE |
|  | Wang et al., 2011 | Quality-of-care indicators for children with sickle cell disease | ABSTRACT |
|  | Wang et al., 2018 | The role of nitrite in muscle function, susceptibility to contraction injury, and fatigability in sickle cell mice | TITLE |
|  | Wanget al., 2022 | EE194 Modelling Long-Term Clinical Outcomes of Patients With Sickle Cell Disease With Recurrent Vaso-Occlusive Crises in the United States | TITLE |
|  | Wanget al., 2013 | Targeting potassium channels Kv1.3 and KCa3.1: Routes to selective immunomodulators in autoimmune disorder treatment? | TITLE |
|  | Wanget al., 2022 | Investigation of the Role of Fgf23 in the Pathogenesis of Post-Splenectomy Cardiovascular Dysfunction | TITLE |
|  | Wanget al., 2018 | Red blood cell derived microparticles contribute to development of post-splentomy pulmonary hypertension in a thalassemic murine model | TITLE |
|  | Wang et al., 2018 | Manifestations of sickle cell disease on thoracic imaging | TITLE |
|  | Wanget al., 2007 | The pathophysiology, prevention, and treatment of stroke in sickle cell disease | TITLE |
|  | Wang et al., 2008 | The pharmacotherapy of sickle cell disease | TITLE |
|  | Wanget al., 2016 | Minireview: Prognostic factors and the response to hydroxurea treatment in sickle cell disease | TITLE |
|  | Wanget al., 2010 | Levels of placental growth factor and endothelin-1 in adults with sickle cell disease are linked to markers of hemolysis, inflammation, iron overload and pulmonary hypertension | TITLE |
|  | Wangt al., 2014 | Heme-bound iron activates placenta growth factor in erythroid cells via erythroid Kruppel-like factor | ABSTRACT |
|  | Wanget al., 2023 | Ferroptosis: a new strategy for cardiovascular disease | TITLE |
|  | Wankoet al., 2005 | Transfusion management in sickle cell disease | TITLE |
|  | Ware et al., 2009 | Clinical year in review I: Interstitial lung disease, pulmonary vascular disease, pulmonary infections, and cardiopulmonary exercise testing and pulmonary rehabilitation | TITLE |
|  | Watcht al., 2023 | Children with palliative care needs in Papua New Guinea, and perspectives from their parents and health care workers: a qualitative study | TITLE |
|  | Waughet al., 2005 | Arginine metabolism, pulmonary hypertension, and sickle cell disease | TITLE |
|  | Weirt al., 2017 | Prostacyclin-analog therapy in sickle cell pulmonary hypertension | TITLE |
|  | Wengrofskyet al., 2019 | Functional Mitral Regurgitation and Congestive Heart Failure in Sickle Cell Anemia and Hemoglobin SC Disease | TITLE |
|  | Westermanet al., 2004 | Plasma 'free' HB is related to red cell derived vesicle numbers in sickle cell anemia and thalassemia intermedia: Implications for nitric oxide (NO) scavenging and pulmonary hypertension | TITLE |
|  | Westermanet al., 2005 | Hydroxyurea Treated Sickle Disorders Demonstrate Decreased Plasma Hb, Red Cell Vesicles and Thrombolysis | ABSTRACT |
|  | Wells et al., 2019 | Heparin Resistance due to an Acquired Antithrombin Deficiency in a Patient With Sickle Cell Disease During a Pregnancy Complicated by Bilateral Pulmonary Emboli: A Case Report | Title |
|  | Westwood et al., 2007 | Myocardial tissue characterization and the role of chronic anemia in sickle cell cardiomyopathy | TITLE |
|  | Wheeler et al., 2019 | Genomic characterization of the RH locus detects complex and novel structural variation in multi-ethnic cohorts | ABSTRACT |
|  | Whipple et al., 2021 | Sickle cell disease and ventricular myocardial strain: A systematic review | TITLE |
|  | Whipple et al., 2018 | Ventricular global longitudinal strain is altered in children with sickle cell disease | TITLE |
|  | Westerman al., 2009 | Phenotypic and Genetic Discordance in Monozygotic Twins with Sickle Anemia and ð-Thalassemia | TITLE |
|  | Whipplet al., 2015 | Altered ventricular global longitudinal strain in children with sickle cell disease | TITLE |
|  | Whiteheadet al., 2003 | Pregnancy-related mortality due to cardiomyopathy: United States, 1991-1997 | TITLE |
|  | Whitesellet al., 2016 | Sleep-disordered breathing and nocturnal hypoxemia in young adults with sickle cell disease | TITLE |
|  | White et al., 2006 | Applications of transcranial Doppler in the ICU: a review | TITLE |
|  | Wilhelm et al., 2000 | Differential diagnosis and management of an infant presenting in shock with a history of sickle cell anemia and a recent fall | TITLE |
|  | Willen et al., 2018 | Age is a predictor of a small decrease in lung function in children with sickle cell anemia | TITLE |
|  | Williams et al., 2021 | Investigational curative gene therapy approaches to sickle cell disease | TITLE |
|  | Wiegand et al., 2019 | Recent advances in the applications of iPSC technology | TITLE |
|  | Wilkinset al., 2013 | Update in pulmonary vascular diseases 2012 | TITLE |
|  | Willenet al., 2017 | What is the role of screening for pulmonary hypertension in adults and children with sickle cell disease? | TITLE |
|  | Williams et al., 2023 | Rhabdomyolysis aggravates renal iron accumulation and acute kidney injury in a humanized mouse model of sickle cell disease | TITLE |
|  | Wills et al., 2009 | Immunomagnetic isolation of canine circulating endothelial and endothelial progenitor cells | TITLE |
|  | Willenet al., 2019 | Asthma in children with sickle cell disease | TITLE |
|  | Williamst al., 2018 | Sickle Cell Anemia and Its Phenotypes | TITLE |
|  | Williamset al., 2023 | Prediction of Adverse Obstetrical Outcomes in Pregnant Patients with Sickle Cell Disease Based on Tricuspid Regurgitant Velocity - A Retrospective Cohort Study | TITLE |
|  | Winslowet al., 2013 | Oxygen: the poison is in the dose | TITLE |
|  | Wiseet al., 2011 | Executive summary of respiratory indications for polysomnography in children: An evidence-based review | TITLE |
|  | Winslow et al., 2000 | Blood substitutes | TITLE |
|  | Wolfet al., 2022 | Updated Guidance on Use and Prioritization of Monoclonal Antibody Therapy for Treatment of COVID-19 in Adolescents | TITLE |
|  | Wonget al., 2013 | Reactive oxygen species and antioxidants in pulmonary hypertension | TITLE |
|  | Wongtongt al., 2015 | Monocytosis is associated with hemolysis in sickle cell disease | TITLE |
|  | Wonkamet al., 2015 | Sickle cell disease and H3Africa: Enhancing genomic research on cardiovascular diseases in African patients | TITLE |
|  | Wonkam et al., 2023 | The future of sickle cell disease therapeutics rests in genomics | TITLE |
|  | Wood et al., 2008 | Cardiac iron across different transfusion-dependent diseases | TITLE |
|  | Woodet al., 2016 | The heart in sickle cell disease, a model for heart failure with preserved ejection fraction | TITLE |
|  | Wood et al., 2004 | Differential expression of E- and P-selectin in the microvasculature of sickle cell transgenic mice | TITLE |
|  | Wood et al., 2022 | Loss of Cyb5R3 Function Silences Fetal Hemoglobin and Hematocrit Responses to Hydroxyurea in Patients with Sickle Cell Anemia | TITLE |
|  | Woodet al., 2019 | Smooth muscle cytochrome b5 reductase 3 deficiency accelerates pulmonary hypertension development in sickle cell mice | TITLE |
|  | Woodet al., 2020 | Sickle cell disease: At the crossroads of pulmonary hypertension and diastolic heart failure | TITLE |
|  | Woodet al., 2008 | Sickle cell disease vasculopathy: a state of nitric oxide resistance | TITLE |
|  | Wriggeet al., 2015 | Adjunctive therapy with inhaled nitric oxide for severe acute chest syndrome in patients with sickle cell disease | TITLE |
|  | Wrightet al., 2019 | 360o View of a Day Hospital Program Performing Exchange Transfusion and Outpatient Pain Management on Adults with Sickle Cell Disease | TITLE |
|  | Wright et al., 2016 | Central venous access device-related Bacillus cereus endocarditis: A case report and review of the literature | TITLE |
|  | Wu et al., 2020 | Research Advances in the Subtype of Sepsis-Associated Thrombocytopenia | TITLE |
|  | Wuet al., 2007 | Mixed haematopoietic chimerism for sickle cell disease prevents intravascular haemolysis | TITLE |
|  | Wu et al., 2023 | Increased Susceptibility for Adverse Reactions to Ultrasound Enhancing Agents in Sickle Cell Disease | TITLE |
|  | Wu et al., 2015 | Abnormal Regulation of Microvascular Tone in a Murine Model of Sickle Cell Disease Assessed by Contrast Ultrasound | TITLE |
|  | Xie et al., 2013 | Sickle cell anemia: the impact of discovery, politics, and business | TITLE |
|  | Xu et al., 2020 | The Gut Microbiome Regulates Psychological-Stress-Induced Inflammation | TITLE |
|  | Yacobovichet al., 2014 | Thalassemia major and sickle cell disease in adolescents and young adults | TITLE |
|  | Yadavet al., 2023 | Clinical Correlates of Acute and Chronic Pulmonary Complications in Children with Sickle Cell Disease from Madhya Pradesh, India: A Cross-sectional Study | TITLE |
|  | Yamaja Setty et al., 2001 | Thrombophilia in sickle cell disease: The red cell connection | ABSTRACT |
|  | Yan et al., 2022 | A National Analysis of Inpatient Pediatric Adenoidectomy | TITLE |
|  | Yang et al., 2022 | Application of a new precision surgical system in treatment of hypertrophic obstructive cardiomyopathy model pigs | Title |
|  | Yasaraet al., 2021 | A comprehensive review of hydroxyurea for β-haemoglobinopathies: the role revisited during COVID-19 pandemic | Title |
|  | Yateset al., 2019 | Elevated tricuspid regurgitation velocity in congenital hemolytic anemias: Prevalence and laboratory correlates | TITLE |
|  | Yawnet al., 2015 | Management of patients with sickle cell disease-reply | ABSTRACT |
|  | Yawnet al., 2014 | Management of sickle cell disease: summary of the 2014 evidence-based report by expert panel members | TITLE |
|  | Yinet al., 2013 | Pulmonary hypertension risk in patients with hemoglobin h disease: low incidence and absence of correlation with splenectomy | TITLE |
|  | Yaseen et al., 2018 | Red blood-cell alloantibodies in multiply transfused patients in the occupied Palestinian territory: a pilot study | ABSTRACT |
|  | Yeo et al., 2010 | Role of transcranial doppler ultrasonography in cerebrovascular disease | TITLE |
|  | Yousafzai et al., 2010 | Open heart surgery in patients with sickle cell hemoglobinopathy | Title |
|  | Yotsumoto Fertrinet al., 2012 | Monocyte Shift to a Non-Classical CD14dim/CD16+ Phenotype Correlates with Fetal Hemoglobin Levels in Sickle Cell Anemia Patients Treated with Hydroxyurea | Title |
|  | Young et al., 2004 | Pulmonary Hypertension in Pediatric Patients with Sickle Cell Disease; a Retrospective Study | ABSTRACT |
|  | Younget al., 2016 | Fatal sickle cell crisis due to Candida albicans infection in a child | ABSTRACT |
|  | Youngblood et al., 2010 | A GCH1 haplotype associated with susceptibility to vasoocclusive pain and impaired vascular function in sickle cell anemia | TITLE |
|  | Yousry et al., 2016 | Endothelial nitric oxide synthase gene polymorphisms and the risk of vasculopathy in sickle cell disease | ABSTRACT |
|  | Youssry al., 2020 | Right ventricular functions in subphenotypes of sickle cell disease | TITLE |
|  | Yousef et al., 2022 | Acute chest syndrome in pediatric sickle cell disease: A 19-year tertiary center experience | TITLE |
|  | Yu et al., 2019 | Inhaled nitric oxide | Title |
|  | Yuan et al., 2022 | Deep learning-based quality-controlled spleen assessment from ultrasound images | TITLE |
|  | Yuditskayaet al., 2009 | Proteomic identification of altered apolipoprotein patterns in pulmonary hypertension and vasculopathy of sickle cell disease | Title |
|  | Yürüket al., 2018 | Treatment of chronic back and chest pain in a patient with sickle cell disease using spinal cord stimulation | TITLE |
|  | Zadeh et al., 2021 | Acute soft head syndrome in a sickle cell disease patient | TITLE |
|  | Zathar et al., 2020 | Constrictive pericarditis masquerading as hepatic sequestration crisis in a patient with sickle cell disease: A case report | Title |
|  | Zaïenet al., 2015 | Pulmonary complications of haematological malignancies | Title |
|  | Zaïent al., 2015 | Atteintes pulmonaires spécifiques des hémopathies malignes | TITLE |
|  | Zaïenet al., 2015 | Pulmonary complications of sickle cell disease | TITLE |
|  | Zaïenet al., 2015 | Drépanocytoses et complications pulmonaires | ABSTRACT |
|  | Zanetteet al., 2008 | Alloimmunization Does Not Modify the Clinical Profile of Sickle Cell Disease Patients from Salvador-Brazil | TITLE |
|  | Zangaladze et al., 2017 | The effects of iron status on pulmonary hypertenision in patients with sickle cell disease | TITLE |
|  | Zanjaniet al., 2012 | Platelets in pulmonary hypertension: A causative role or a simple association? | ABSTRACT |
|  | Zawar et al., 2005 | Non-invasive detection of endothelial dysfunction in sickle cell disease by Doppler ultrasonography | ABSTRACT |
|  | Zeppieri et al., 2021 | Impact of autoimmune disease and its treatment on adults with sickle cell disease | Abstract |
|  | Zeitoune et al., 2020 | Diaphragm ultrasonography in adults with sickle cell anemia: evaluation of morphological and functional aspects | TITLE |
|  | Zeno et al., 2023 | Outcomes before and after providing interdisciplinary hematology and pulmonary care for children with sickle cell disease | TITLE |
|  | Zhanget al., 2022 | Molecular insight into pentraxin-3: Update advances in innate immunity, inflammation, tissue remodeling, diseases, and drug role | TITLE |
|  | Zhanget al., 2020 | Influence of single parenthood on cardiopulmonary function in pediatric patients with sickle cell anemia | TITLE |
|  | Zhanget al., 2016 | Magnetic resonance imaging in pediatric sickle cell anemia | ABSTRACT |
|  | Zhanget al., 2019 | Biomarkers of Cardiopulmonary, Renal, and Liver Dysfunction in an Adult Sickle Cell Disease Cohort | TITLE |
|  | Zhanget al., 2014 | Genetic association of a MAPK8 expression quantitative trait locus with pre-capillary pulmonary hypertension in sickle cell disease | ABSTRACT |
|  | Zhanget al., 2014 | Hypoxic response contributes to altered gene expression and precapillary pulmonary hypertension in patients with sickle cell disease | ABSTRACT |
|  | Zhanget al., 2011 | Association of genetic variation in the catechol-O-methyl transferase gene with pain and six minute walk distance in sickle cell anemia patients from the walk-PHaSST study | ABSTRACT |
|  | Zheng et al., 2016 | Sickle Cell Crisis Complicated by Synthetic Cannabinoid Abuse: A Case Report | ABSTRACT |
|  | Zhou et al., 2012 | Molecular link between intravascular hemolysis and vascular occlusion in sickle cell disease | Title |
|  | Zimbarra Cabritaet al., 2013 | The association between tricuspid regurgitation velocity and 5-year survival in a North West London population of patients with sickle cell disease in the United Kingdom | ABSTRACT |
|  | Zilberman et al., 207 | Evaluation of left ventricular diastolic function in pediatric sickle cell disease patients | ABSTRACT |
|  | Zorcaet al., 2010 | Lipid levels in sickle-cell disease associated with haemolytic severity, vascular dysfunction and pulmonary hypertension | Abstract |
|  | Zuckerman et al., 2011 | Pulmonary hypertension in children with sickle cell disease | ABSTRACT |
|  | Zumberg et al., 2005 | Hydroxyurea therapy for sickle cell disease in community-based practices: a survey of Florida and North Carolina hematologists/oncologists | ABSTRACT |
|  | Zubair et al., 2018 | Incorporating low-cost spirometry testing of pulmonary function into comprehensive care for children with sickle cell anemia in northwestern Nigeria: a multicountry collaboration for achieving a sustainable initiative | TITLE |
