# Supplementary material for: Global prevalence of elevated estimated pulmonary artery systolic pressure in clinically stable children and adults with sickle cell disease: A systematic review and meta-analysis
Source: PLoS One. 2025 Feb 13;20(2):e0318751. doi: 10.1371/journal.pone.0318751 (PMC11825009; doi:10.1371/journal.pone.0318751)
Supplement: S4 File — (DOCX) [file pone.0318751.s004.docx]

**eTable.** A list of excluded studies at full text review with brief report.

| No. | Author | Title | Brief report for excluded studies |
| --- | --- | --- | --- |
| 1 | Akkus et al., 2021 | Troponin Elevation in Sickle Cell Disease | Insufficient data |
| 2 | Benites et al., 2017 | Echocardiografic Abnormalities in Patients with Sickle Cell/β-Thalassemia Do Not Depend on the β-Thalassemia Phenotype | Insufficient data |
| 3 | Duru et al., 2021 | Variations and characteristics of the various clinical phenotypes in a cohort of Nigerian sickle cell patients | Insufficient data |
| 4 | Fitzhugh et al., 2015 | Hydroxyurea-Increased Fetal Hemoglobin Is Associated with Less Organ Damage and Longer Survival in Adults with Sickle Cell Anemia | Insufficient data |
| 5 | Giray et al., 2023 | Longitudinal Changes in Cardiac Function Based on Serial Tissue Doppler and Doppler Imaging for Patients With Sickle Cell Anemia | Insufficient data |
| 6 | Gladwin et al., 2014 | Risk factors for death in 632 patients with sickle cell disease in the United States and United Kingdom | Insufficient data |
| 7 | Hammoudi et al., 2015 | Left atrial volume is not an index of left ventricular diastolic dysfunction in patients with sickle cell anaemia | Insufficient data |
| 8 | Resende et al., 2020 | Prognostic value of left ventricular longitudinal strain by speckle-tracking echocardiography in patients with sickle cell disease | Insufficient data |
| 9 | Akgul et al., 2019 | Decreased Heart Rate Variability in Sickle Cell Anemia as Effect of Pulmonary Arterial Hypertension | Non-compliance with inclusion criteria |
| 10 | Akgül et al., 2007 | Increased QT dispersion in sickle cell disease: Effect of pulmonary hypertension | Non-compliance with inclusion criteria |
| 11 | Akgül et al., 2006 | Right ventricular and pulmonary function in sickle cell disease patients with pulmonary hypertension | Non-compliance with inclusion criteria |
| 12 | Alameri et al., 2008 | Dyspnea, pulmonary function and exercise capacity in adult Saudi patients with sickle cell disease | Non-compliance with inclusion criteria |
| 13 | Molavi et al., 2015 | Evaluation of Tricuspid Regurgitation Jet Velocity in Children with Sickle Cell Disease in Iran 2012-2013 | Non-compliance with inclusion criteria |
| 14 | Aliyu et al., 2010 | NT-proBNP as a marker of cardiopulmonary status in sickle cell anaemia in Africa: Research paper | Non-compliance with inclusion criteria |
| 15 | Alkholy et al., 2019 | Vascular endothelial growth factor and pulmonary hypertension in children with beta thalassemia major | Non-compliance with inclusion criteria |
| 16 | Al-Khoufi et al., 2013 | Prevalence of pulmonary arterial hypertension among sickle cell disease patients in Al Hassa | Non-compliance with inclusion criteria |
| 17 | Alkindi et al., 2020 | Clinical and laboratory parameters, risk factors predisposing to the development of priapism in sickle cell patients | Non-compliance with inclusion criteria |
| 18 | Ambrusko et al., 2006 | Elevation of tricuspid regurgitant jet velocity, a marker for pulmonary hypertension in children with sickle cell disease | Non-compliance with inclusion criteria |
| 19 | Amoozgar et al., 2015 | Evaluation of cardiac sequelae in patients with sickle cell anemia | Non-compliance with inclusion criteria |
| 20 | Amoozgar et al., 2017 | The relationship of coronary sinus dilation with pulmonary artery pressure in pediatric patients | Non-compliance with inclusion criteria |
| 21 | Anthi et al., 2007 | Hemodynamic and functional assessment of patients with sickle cell disease and pulmonary hypertension | Non-compliance with inclusion criteria |
| 22 | Ashley-Koch et al., 2008 | Identification of genetic polymorphisms associated with risk for pulmonary hypertension in sickle cell disease | Non-compliance with inclusion criteria |
| 23 | Ataga et al., 2010 | Association of soluble fms-like tyrosine kinase-1 (sFLT-1) with pulmonary hypertension and hemolysis in sickle cell disease | Non-compliance with inclusion criteria |
| 24 | Ataga et al., 2008 | Coagulation activation and inflammation in sickle cell disease-associated pulmonary hypertension | Non-compliance with inclusion criteria |
| 25 | Ataga et al., 2006 | Pulmonary hypertension in patients with sickle cell disease | Non-compliance with inclusion criteria |
| 26 | Ataga et al., 2004 | Pulmonary hypertension in sickle cell disease | Non-compliance with inclusion criteria |
| 27 | Bakhtar et al., 2020 | Study of pulmonary hypertension as a complication of sickle cell disease | Non-compliance with inclusion criteria |
| 28 | Barbosa et al., 2014 | Assessment of Ventricular Function in Adults with Sickle Cell Disease: Role of Two-Dimensional Speckle-Tracking Strain | Non-compliance with inclusion criteria |
| 29 | Barst et al., 2010 | Exercise capacity and haemodynamics in patients with sickle cell disease with pulmonary hypertension treated with bosentan: Results of the ASSET studies | Non-compliance with inclusion criteria |
| 30 | Belisário et al., 2020 | Association of HIV infection with clinical and laboratory characteristics of sickle cell disease | Non-compliance with inclusion criteria |
| 31 | Biassi et al., 2022 | miRNA profile and disease severity in patients with sickle cell anemia | Non-compliance with inclusion criteria |
| 32 | Chanet al., 2023 | Pulmonary hypertension screening in children with sickle cell disease | Non-compliance with inclusion criteria |
| 33 | Chaturvedi et al., 2018 | Clustering of end-organ disease and earlier mortality in adults with sickle cell disease: A retrospective-prospective cohort study | Non-compliance with inclusion criteria |
| 34 | Chaturvedi et al., 2017 | Elevated tricuspid regurgitant jet velocity, reduced forced expiratory volume in 1 second, and mortality in adults with sickle cell disease | Non-compliance with inclusion criteria |
| 35 | Chaurasia et al., 2023 | Study of electrocardiographic and echocardiographic changes in sickle cell anaemia patients | Non-compliance with inclusion criteria |
| 36 | Chaurasia et al., 2023 | Prevalence of pulmonary hypertension in children with sickle cell disease | Non-compliance with inclusion criteria |
| 37 | Colella et al., 2015 | Elevated hypercoagulability markers in hemoglobin SC disease | Non-compliance with inclusion criteria |
| 38 | Connor et al., 2018 | Brief topical sodium nitrite and its impact on the quality of life in patients with sickle leg ulcers | Non-compliance with inclusion criteria |
| 39 | Cramer-Bour et al., 2021 | Long-term tolerability of phosphodiesterase-5 inhibitors in pulmonary hypertension of sickle cell disease | Non-compliance with inclusion criteria |
| 40 | Desai et al., 2013 | Longitudinal study of echocardiography-derived tricuspid regurgitant jet velocity in sickle cell disease | Non-compliance with inclusion criteria |
| 41 | Dham et al., 2009 | Prospective echocardiography assessment of pulmonary hypertension and its potential etiologies in children with sickle cell disease | Non-compliance with inclusion criteria |
| 42 | Dickerson et al., 2012 | Young adults with SCD in US children's hospitals: Are they different from adolescents? | Non-compliance with inclusion criteria |
| 43 | Dubert et al., 2017 | Degree of anemia, indirect markers of hemolysis, and vascular complications of sickle cell disease in Africa | Non-compliance with inclusion criteria |
| 44 | Elalfy et al., 2018 | Left Ventricular Structural and Functional Changes in Children With β-Thalassemia and Sickle Cell Disease: Relationship to Sleep-disordered Breathing | Non-compliance with inclusion criteria |
| 45 | Ellithy et al., 2015 | Relation between glutathione s transferase genes (GSTM1, GSTT1 and GSTP1) polymorphisms and clinical manifestations of sickle cell disease in Egyptian patients | Non-compliance with inclusion criteria |
| 46 | Enakpene et al., 2014 | Non-invasive estimation of pulmonary artery pressures in patients with sickle cell anaemia in Ibadan, Nigeria: an echocardiographic study | Non-compliance with inclusion criteria |
| 47 | Ershler et al., 2023 | Hemoglobin and End-Organ Damage in Individuals with Sickle Cell Disease | Non-compliance with inclusion criteria |
| 48 | Estrada Del Cueto et al., 2010 | Analysis of some hematological, biochemical and clinical variables in cases of sickle cell anemia. Preliminary results | Non-compliance with inclusion criteria |
| 49 | Feld et al., 2015 | Liver injury is associated with mortality in sickle cell disease | Non-compliance with inclusion criteria |
| 50 | Garnier et al., 2017 | Differences of microparticle patterns between sickle cell anemia and hemoglobin SC patients | Non-compliance with inclusion criteria |
| 51 | Gladwin et al., 2004 | Pulmonary Hypertension as a Risk Factor for Death in Patients with Sickle Cell Disease | Non-compliance with inclusion criteria |
| 52 | Graham et al., 2007 | Sickle cell lung disease and sudden death: A retrospective/prospective study of 21 autopsy cases and literature review | Non-compliance with inclusion criteria |
| 53 | Hagar et al., 2008 | Clinical differences between children and adults with pulmonary hypertension and sickle cell disease | Non-compliance with inclusion criteria |
| 54 | Halabi-Tawil et al., 2008 | Sickle cell leg ulcers: a frequently disabling complication and a marker of severity | Non-compliance with inclusion criteria |
| 55 | Halphen et al., 2014 | Severe Nocturnal and Postexercise Hypoxia in Children and Adolescents with Sickle Cell Disease | Non-compliance with inclusion criteria |
| 56 | Jiang et al., 2014 | A retrospective analysis of 19 patients with thalassemia complicated with pulmonary hypertension | Non-compliance with inclusion criteria |
| 57 | Jutant et al., 2021 | Endothelial dysfunction and hypercoagulability in severe sickle-cell acute chest syndrome | Non-compliance with inclusion criteria |
| 58 | Kato et al., 2005 | Levels of soluble endothelium-derived adhesion molecules in patients with sickle cell disease are associated with pulmonary hypertension, organ dysfunction, and mortality | Non-compliance with inclusion criteria |
| 59 | Kato et al., 2009 | Endogenous nitric oxide synthase inhibitors in sickle cell disease: Abnormal levels and correlations with pulmonary hypertension, desaturation, haemolysis, organ dysfunction and death | Non-compliance with inclusion criteria |
| 60 | Liem et al., 2009 | Tricuspid regurgitant jet velocity elevation and its relationship to lung function in pediatric sickle cell disease | Non-compliance with inclusion criteria |
| 61 | Liem et al., 2009 | Functional capacity in children and young adults with sickle cell disease undergoing evaluation for cardiopulmonary disease | Non-compliance with inclusion criteria |
| 62 | Liem et al., 2007 | Tricuspid regurgitant jet velocity is associated with hemolysis in children and young adults with sickle cell disease evaluated for pulmonary hypertension | Non-compliance with inclusion criteria |
| 63 | Manwar et al., 2023 | A study of Electrocardigraphic and Echocardiographic changes in sickle cell anemia patients – Observational Study | Non-compliance with inclusion criteria |
| 64 | Mbakamma, et al., 2019 | Pulmonary Hypertension in Adults with Sickle Cell Anaemia: A Prevalence Study in the Niger Delta Region of Nigeria | Non-compliance with inclusion criteria |
| 65 | Mehari et al., 2019 | Abnormal ventilation-perfusion scan is associated with pulmonary hypertension in sickle cell adults | Non-compliance with inclusion criteria |
| 66 | Michel et al., 2008 | Characteristics and Outcome of Connective Tissue Diseases in Patients with Sickle-Cell Disease: Report of 30 Cases | Non-compliance with inclusion criteria |
| 67 | Mokhtar et al., 2010 | N-terminal natriuretic peptide and ventilation-perfusion lung scan in sickle cell disease and thalassemia patients with pulmonary hypertension | Non-compliance with inclusion criteria |
| 68 | Morris et al., 2011 | Risk factors and mortality associated with an elevated tricuspid regurgitant jet velocity measured by Doppler-echocardiography in thalassemia: a Thalassemia Clinical Research Network report | Non-compliance with inclusion criteria |
| 69 | Musa et al., 2018 | Lower than expected elevated tricuspid regurgitant jet velocity in adults with sickle cell disease in Nigeria | Non-compliance with inclusion criteria |
| 70 | Niu et al., 2009 | Angiogenic and inflammatory markers of cardiopulmonary changes in children and adolescents with sickle cell disease | Non-compliance with inclusion criteria |
| 71 | Oguanobi et al., 2015 | Echocardiographic assessment of adult Nigerian sickle cell patients with pulmonary hypertension | Non-compliance with inclusion criteria |
| 72 | Oni et al., 2019 | Right ventricular function assessment in sickle cell anaemia patients using echocardiography | Non-compliance with inclusion criteria |
| 73 | Oni et al., 2022 | Pulmonary hypertension and right ventricular function in the sickle cell populace | Non-compliance with inclusion criteria |
| 74 | Oni et al., 2023 | Pulmonary Hypertension and Left Ventricular Geometric Types in Sickle Cell Anemia | Non-compliance with inclusion criteria |
| 75 | Onyekwere et al., 2008 | Pulmonary hypertension in children and adolescents with sickle cell disease | Non-compliance with inclusion criteria |
| 76 | Ozoh et al., 2019 | Pulmonary dysfunction among adolescents and adults with sickle cell disease in Nigeria: Implications for monitoring | Non-compliance with inclusion criteria |
| 77 | Pashankar et al., 2009 | Longitudinal follow up of elevated pulmonary artery pressures in children with sickle cell disease | Non-compliance with inclusion criteria |
| 78 | Prohaska et al., 2023 | RASA3 is a candidate gene in sickle cell disease-associated pulmonary hypertension and pulmonary arterial hypertension | Non-compliance with inclusion criteria |
| 79 | Rai et al., 2021 | Longitudinal effect of disease-modifying therapy on tricuspid regurgitant velocity in children with sickle cell anemia | Non-compliance with inclusion criteria |
| 80 | Ranque et al., 2023 | Association of haemolysis markers, blood viscosity and microcirculation function with organ damage in sickle cell disease in sub-Saharan Africa (the BIOCADRE study) | Non-compliance with inclusion criteria |
| 81 | Shah et al., 2021 | Tricuspid regurgitant jet velocity and myocardial tissue Doppler parameters predict mortality in a cohort of patients with sickle cell disease spanning from pediatric to adult age groups - revisiting this controversial concept after 16 years of additional evidence | Non-compliance with inclusion criteria |
| 82 | Soh et al., 2016 | Mildly raised tricuspid regurgitant velocity 2.5–3.0 m/s in pregnant women with sickle cell disease is not associated with poor obstetric outcome – An observational cross-sectional study | Non-compliance with inclusion criteria |
| 83 | Sokal et al., 2016 | African sickle beta zero-thalassemia patients vs sickle cell anemia patients: similar clinical features but less severe hemolysis | Non-compliance with inclusion criteria |
| 84 | Tantawy et al., 2023 | Sleep disordered breathing and its relation to stroke and pulmonary hypertension in children with sickle cell disease: a single-center cross-sectional study | Non-compliance with inclusion criteria |
| 85 | Tantawy et al., 2014 | Growth differentiation factor-15 in young sickle cell disease patients: relation to hemolysis, iron overload and vascular complications | Non-compliance with inclusion criteria |
| 86 | Upadhya et al., 2014 | Echocardiography-derived tricuspid regurgitant jet velocity is an important marker for the progression of sickle-cell disease | Non-compliance with inclusion criteria |
| 87 | van Beers et al., 2014 | Exercise tolerance, lung function abnormalities, anemia, and cardiothoracic ratio in sickle cell patients | Non-compliance with inclusion criteria |
| 88 | Villers et al., 2008 | Morbidity associated with sickle cell disease in pregnancy | Non-compliance with inclusion criteria |
| 89 | Yates et al., 2019 | Elevated tricuspid regurgitation velocity in congenital hemolytic anemias: Prevalence and laboratory correlates | Non-compliance with inclusion criteria |
| 90 | Zimbarra Cabrita et al., 2013 | The association between tricuspid regurgitation velocity and 5-year survival in a North West London population of patients with sickle cell disease in the United Kingdom | Non-compliance with inclusion criteria |
| 91 | Hariharan et al., 2023 | Pulmonary Hypertension among Children and Adolescents with Sickle Cell Anemia: A Systematic Review and Meta-analysis | Review |
| 92 | Caughey et al., 2015 | Estimated pulmonary artery systolic pressure and sickle cell disease: a meta-analysis and systematic review | Review |
| 93 | Bigna et al., 2017 | Prevalence and etiologies of pulmonary hypertension in Africa: A systematic review and meta-analysis | Review |
