# Supplementary material for: Global prevalence of elevated estimated pulmonary artery systolic pressure in clinically stable children and adults with sickle cell disease: A systematic review and meta-analysis
Source: PLoS One. 2025 Feb 13;20(2):e0318751. doi: 10.1371/journal.pone.0318751 (PMC11825009; doi:10.1371/journal.pone.0318751)
Supplement: S5 File — (DOC) [file pone.0318751.s005.doc]

**Modified Checklist**

This checklist was considered based on the Newcastle–Ottawa scale (NOS) checklist and the Joanna Briggs Institute (JBI) critical appraisal checklist for the risk of bias assessment. Based on the two checklists, our study design, and findings of the included studies, we prepared a modified version of the checklist. In the following, the items of the modified checklist are mentioned in detail.

1. **Was the sample frame appropriate to address the target population? :** 1 point was given if the sample frame appropriately addressed.
2. **Representativeness of the sample:** 1 point was given if the sample was truly representative of the average in the target population (all subjects or random sampling) or somewhat representative (non-random sampling).
3. **Sample size:** 1 point was given if sample size was justified and satisfactory (≥ 150).
4. **Were the study subjects and the setting described in detail? :** 1 point was given if they described in detail.
5. **Was the data analysis conducted with sufficient coverage of the identified sample? :** 1 point was given if the sufficient coverage ≥ 85%.
6. **Were valid methods used for the identification of the condition?**
7. **Was the condition measured in a standard, reliable way for all participants? :** 1 point was given if the condition measured by one/the same observer or an inter-observer agreement of more than 95%.
8. **Was the response rate adequate, and if not, was the low response rate managed appropriately? :** 1 point was given if the inclusion rate was satisfactory or if comparability between included and non-included subjects was established in studies where the included subjects were not satisfactory.
9. **Risk of bias assessment (adult studies)**

| **Author, Year** | **Type of study** | **item 1** | **item 2** | **item 3** | **item 4** | **item 5** | **item 6** | **item 7** | **Item 8** | **Risk of bias** |
| --- | --- | --- | --- | --- | --- | --- | --- | --- | --- | --- |
| Afriyie-Mensah, 2018 | Cross-sectional | 0 | 1 | 0 | 1 | 1 | 1 | 0 | 1 | Medium |
| Amadi, 2017 | Cross-sectional | 0 | 0 | 0 | 1 | 1 | 1 | 0 | 1 | High |
| Billy-Brissac, 2009 | Cross-sectional | 1 | 0 | 1 | 1 | 1 | 1 | 0 | 1 | Medium |
| Caughey, 2012 | Cross-sectional | 1 | 0 | 0 | 0 | 0 | 1 | 1 | 0 | High |
| Chiadika, 2018 | Cross-sectional | 1 | 1 | 0 | 1 | 1 | 1 | 0 | 1 | Medium |
| Dei-Adomakoh, 2019 | Cross-sectional | 0 | 1 | 0 | 1 | 1 | 1 | 1 | 1 | Medium |
| Elmariah, 2014 | Cross-sectional | 0 | 0 | 1 | 1 | 0 | 1 | 0 | 0 | High |
| Klings, 2008 | Cross-sectional | 1 | 1 | 0 | 1 | 0 | 1 | 0 | 0 | High |
| Lopes, 2017 | Cross-sectional | 0 | 0 | 0 | 1 | 1 | 1 | 0 | 1 | High |
| Maioli, 2016 | Cross-sectional | 0 | 0 | 0 | 1 | 1 | 1 | 1 | 1 | Medium |
| Maikap, 2023 | Cross-sectional | 0 | 1 | 0 | 1 | 1 | 1 | 0 | 1 | Medium |
| de Lima Marinho, 2016 | Cross-sectional | 0 | 0 | 0 | 1 | 0 | 1 | 1 | 0 | High |
| Odeyemi, 2022 | Cross-sectional | 0 | 0 | 0 | 1 | 1 | 1 | 1 | 1 | Medium |
| Oguanobi, 2012 | Cross-sectional | 0 | 0 | 0 | 1 | 1 | 1 | 0 | 1 | High |
| Akgül, 2007 | Case-control | 0 | 0 | 0 | 0 | 1 | 1 | 1 | 1 | High |
| Anjum, 2012 | Case-control | 0 | 0 | 0 | 0 | 1 | 1 | 1 | 1 | High |
| Dosunmu, 2014 | Case-control | 0 | 0 | 0 | 1 | 1 | 1 | 1 | 1 | Medium |
| Abdul-Mohsen, 2012 | Cohort | 0 | 0 | 0 | 1 | 1 | 1 | 0 | 1 | High |
| Aessopos, 2009 | Cohort | 0 | 0 | 0 | 1 | 1 | 1 | 1 | 1 | Medium |
| Aleem, 2007 | Cohort | 0 | 0 | 0 | 1 | 1 | 1 | 1 | 1 | Medium |
| Aliyu, 2008 | Cohort | 0 | 0 | 1 | 1 | 1 | 1 | 1 | 1 | Medium |
| Cabrita, 2013 | Cohort | 1 | 1 | 1 | 1 | 1 | 1 | 1 | 1 | Low |
| Damy, 2016 | Cohort | 0 | 0 | 1 | 1 | 1 | 1 | 0 | 1 | Medium |
| Delclaux, 2005 | Cohort | 0 | 0 | 0 | 1 | 1 | 1 | 0 | 1 | High |
| d'Humières, 2021 | Cohort | 0 | 0 | 1 | 1 | 1 | 1 | 1 | 1 | Medium |
| Fonseca, 2012 | Cohort | 0 | 0 | 0 | 1 | 1 | 1 | 1 | 1 | Medium |
| Garadah, 2019 | Cohort | 0 | 0 | 0 | 1 | 1 | 1 | 1 | 1 | Medium |
| Garrido, 2012 | Cohort | 0 | 0 | 0 | 0 | 1 | 1 | 0 | 1 | High |
| Guedeney, 2018 | Cohort | 0 | 1 | 0 | 0 | 1 | 1 | 1 | 1 | Medium |
| Kato, 2006 | Cohort | 1 | 0 | 1 | 1 | 1 | 1 | 0 | 1 | Medium |
| Knight-Perry, 2011 | Cohort | 0 | 0 | 0 | 1 | 1 | 1 | 1 | 1 | Medium |
| Lobo, 2015 | Cohort | 0 | 0 | 0 | 1 | 1 | 1 | 1 | 1 | Medium |
| Mushemi-Blake, 2015 | Cohort | 0 | 0 | 0 | 1 | 1 | 1 | 0 | 1 | High |
| Olatunya, 2019 | Cohort | 1 | 0 | 0 | 1 | 0 | 1 | 0 | 0 | High |
| Ranque, 2016 | Cohort | 1 | 1 | 1 | 1 | 1 | 1 | 1 | 1 | Low |
| Sachdev, 2011 | Cohort | 1 | 1 | 1 | 1 | 1 | 1 | 0 | 1 | Low |
| Sharma, 2013 | Cohort | 0 | 1 | 0 | 1 | 1 | 1 | 0 | 1 | Medium |
| van Beers, 2008 | Cohort | 0 | 0 | 0 | 1 | 1 | 1 | 0 | 1 | High |
| Victor, 2016 | Cohort | 0 | 0 | 1 | 0 | 1 | 1 | 1 | 1 | Medium |
| Voskaridou, 2007 | Cohort | 0 | 0 | 0 | 0 | 1 | 1 | 0 | 1 | High |
| Parent, 2011 | Trial | 1 | 0 | 1 | 1 | 1 | 1 | 0 | 1 | Medium |

High risk of bias: score 3 or 4, Medium risk of bias: score 5 or 6, Low risk of bias: score 7 or 8.

**B) Risk of bias assessment (children studies)**

| **Author, Year** | **Type of study** | **item 1** | **item 2** | **item 3** | **item 4** | **item 5** | **item 6** | **item 7** | **Item 8** | **Risk of bias** |  |
| --- | --- | --- | --- | --- | --- | --- | --- | --- | --- | --- | --- |
| AboHadeed, 2015 | Cross-sectional | 0 | 0 | 0 | 0 | 0 | 1 | 1 | 1 | High |  |
| Adly, 2016 | Cross-sectional | 0 | 0 | 0 | 1 | 1 | 1 | 0 | 1 | High |  |
| Allen, 2019 | Cross-sectional | 0 | 0 | 0 | 1 | 0 | 1 | 1 | 0 | High |  |
| Al-Allawi, 2016 | Cross-sectional | 0 | 1 | 0 | 1 | 1 | 1 | 0 | 1 | Medium |  |
| Chinawa, 2020 | Cross-sectional | 0 | 0 | 0 | 1 | 1 | 1 | 0 | 1 | High |  |
| Elbarbary, 2016 | Cross-sectional | 0 | 0 | 0 | 1 | 1 | 1 | 1 | 1 | Medium |  |
| El‐Shanshory, 2013 | Cross-sectional | 0 | 0 | 0 | 1 | 1 | 1 | 0 | 1 | High |  |
| Johnson, 2010 | Cross-sectional | 0 | 1 | 0 | 1 | 1 | 1 | 1 | 1 | Medium |  |
| Lamina, 2019 | Cross-sectional | 1 | 1 | 1 | 1 | 1 | 1 | 1 | 1 | Low |  |
| Odeyemi, 2022 | Cross-sectional | 0 | 0 | 0 | 1 | 1 | 1 | 1 | 1 | Medium |  |
| Peter, 2019 | Cross-sectional | 0 | 1 | 0 | 1 | 1 | 1 | 1 | 1 | Medium |  |
| Ribera, 2015 | Cross-sectional | 0 | 1 | 0 | 1 | 0 | 1 | 0 | 0 | High |  |
| Sokunbi, 2017 | Cross-sectional | 0 | 0 | 1 | 1 | 1 | 1 | 1 | 1 | Medium |  |
| ElAlfy, 2019 | Case-control | 0 | 1 | 0 | 1 | 1 | 1 | 1 | 1 | Medium | |
| Hanna, 2021 | Case-control | 0 | 1 | 0 | 1 | 1 | 1 | 1 | 1 | Medium | |
| Marouf, 2013 | Case-control | 0 | 0 | 0 | 1 | 0 | 1 | 1 | 0 | High | |
| Agha, 2014 | Cohort | 0 | 0 | 0 | 1 | 1 | 1 | 0 | 1 | High |  |
| Blanc, 2012 | Cohort | 0 | 0 | 0 | 1 | 0 | 1 | 1 | 0 | High |  |
| Caldas,2008 | Cohort | 0 | 1 | 0 | 0 | 1 | 1 | 0 | 1 | High |  |
| Chaudry, 2011 | Cohort | 0 | 0 | 0 | 1 | 1 | 1 | 0 | 1 | High |  |
| Colombatti, 2010 | Cohort | 1 | 1 | 0 | 1 | 1 | 1 | 1 | 1 | Low |  |
| Cox, 2014 | Cohort | 0 | 0 | 1 | 1 | 1 | 1 | 1 | 1 | Medium |  |
| Dahoui, 2010 | Cohort | 1 | 1 | 0 | 1 | 1 | 1 | 1 | 1 | Low |  |
| Eddine, 2012 | Cohort | 0 | 0 | 0 | 1 | 0 | 1 | 1 | 0 | High |  |
| Forrest, 2012 | Cohort | 0 | 0 | 0 | 1 | 1 | 1 | 0 | 1 | High |  |
| Garnier, 2017 | Cohort | 0 | 0 | 0 | 1 | 0 | 1 | 1 | 0 | High |  |
| Gordeuk, 2009 | Cohort | 0 | 0 | 1 | 1 | 1 | 1 | 0 | 1 | Medium |  |
| Hebson, 2015 | Cohort | 1 | 1 | 1 | 1 | 1 | 1 | 0 | 1 | Low |  |
| Lee, 2009 | Cohort | 0 | 0 | 0 | 1 | 1 | 1 | 0 | 1 | High |  |
| Liem, 2009 | Cohort | 1 | 1 | 0 | 1 | 1 | 1 | 0 | 1 | Medium |  |
| Lilje, 2017 | Cohort | 1 | 0 | 0 | 1 | 1 | 1 | 1 | 1 | Medium |  |
| Minniti, 2009 | Cohort | 1 | 1 | 1 | 1 | 1 | 1 | 0 | 1 | Low |  |
| Nelson, 2007 | Cohort | 0 | 0 | 0 | 1 | 1 | 1 | 1 | 1 | Medium |  |
| Pashankar, 2008 | Cohort | 0 | 1 | 0 | 1 | 0 | 1 | 0 | 0 | High |  |
| Ranque, 2016 | Cohort | 1 | 1 | 1 | 1 | 1 | 1 | 1 | 1 | Low |  |
| Sedrak, 2009 | Cohort | 0 | 1 | 0 | 0 | 1 | 1 | 0 | 1 | High |  |
| Tantawy, 2012 | Cohort | 0 | 0 | 0 | 1 | 1 | 1 | 0 | 1 | High |  |
| Zilberman, 2007 | Cohort | 0 | 0 | 0 | 1 | 0 | 1 | 1 | 0 | High |  |

High risk of bias: score 3 or 4, Medium risk of bias: score 5 or 6, Low risk of bias: score 7 or 8.
