# Supplementary material for: Global prevalence of elevated estimated pulmonary artery systolic pressure in clinically stable children and adults with sickle cell disease: A systematic review and meta-analysis
Source: PLoS One. 2025 Feb 13;20(2):e0318751. doi: 10.1371/journal.pone.0318751 (PMC11825009; doi:10.1371/journal.pone.0318751)
Supplement: S6 File — (DOCX) [file pone.0318751.s006.docx]

**eTable.** **Characteristics of the included studies for adults with sickle cell disease.**

| Author | Year of publication | Study design | Total case | Definition / cut-off | No. Elevated ePASP cases |
| --- | --- | --- | --- | --- | --- |
| Abdul-Mohsen et al., 2012 [1] | 2012 | Cohort | 45 | ePASP, 30 | 18 |
| Aessopos et al., 2009 [2] | 2009 | Cohort | 110 | ePASP, 30 | 31 |
| Afriyie-Mensah et al., 2018 [3] | 2018 | Cross-sectional | 76 | TRV, 2.5 | 25 |
| Akgül et al., 2007 [4] | 2007 | Case-control | 87 | Age, BMI, and Sex ePASP ranges | 33 |
| Aleem et al., 2007 [5] | 2007 | Cohort | 65 | TRV, 2.5 | 25 |
| Aliyu et al., 2008 [6] | 2008 | Cohort | 178 | TRV, 2.5 | 45 |
| Amadi et al., 2017 [7] | 2017 | Cross-sectional | 92 | TRJV, 2.5 | 22 |
| Anjum et al., 2012 [8] | 2012 | Case-control | 36 | TRJV, 2.5 | 14 |
| Billy-Brissac et al., 2009 [9] | 2009 | Cross-sectional | 405 | ePASP, 30 | 42 |
| Cabrita et al., 2013 [10] | 2013 | Cohort | 164 | TRV, 2.5 | 48 |
| Caughey et al., 2012 [11] | 2012 | Cross-sectional | 104 | Age, BMI, and Sex ePASP ranges | 34 |
| Chiadika et al., 2018 [12] | 2018 | Cross-sectional | 91 | TRJV, 2.5 | 35 |
| Damy et al., 2016 [13] | 2016 | Cohort | 656 | TRJV, 2.5 | 114 |
| Dei-Adomakoh et al., 2019 [14] | 2019 | Cross-sectional | 71 | TRJV, 2.5 | 23 |
| Delclaux et al., 2005 [15] | 2005 | Cohort | 49 | ePASP, 30 | 13 |
| d'Humières et al., 2021 [16] | 2021 | Cohort | 379 | TRJV, 2.5 | 94 |
| Dosunmu et al., 2014 [17] | 2014 | Case-control | 56 | ePASP, 25 | 2 |
| Elmariah et al., 2014 [18] | 2014 | Cross-sectional | 222 | TRJV, 2.5 | 73 |
| Fonseca et al., 2012 [19] | 2012 | Cohort | 80 | TRJV, 2.5 | 32 |
| Garrido et al., 2012 [20] | 2012 | Cohort | 41 | TRV, 2.5 | 7 |
| Guedeney et al., 2018 [21] | 2018 | Cohort | 115 | TRJV, 2.5 | 36 |
| Garadah et al., 2019 [22] | 2019 | Cohort | 110 | TRJV, 2.5 | 38 |
| Kato et al., 2006 [23] | 2006 | Cohort | 210 | TRJV, 2.5 | 72 |
| Klings et al., 2008 [24] | 2008 | Cross-sectional | 88 | TRJV, 2.5 | 35 |
| Knight-Perry et al., 2011 [25] | 2011 | Cohort | 53 | TRJV, 2.5 | 21 |
| Lobo et al., 2015 [26] | 2015 | Cohort | 125 | TRJV, 2.5 | 43 |
| Lopes et al., 2017 [27] | 2017 | Cross-sectional | 38 | TRJV, 2.5 | 16 |
| Maioli et al., 2016 [28] | 2016 | Cross-sectional | 59 | TRJV, 2.5 | 25 |
| Maikap et al., 2023 [29] | 2023 | Cross-sectional | 130 | TRJV, 2.5 | 33 |
| de Lima Marinho et al., 2016 [30] | 2016 | Cross-sectional | 45 | TRJV, 2.5 | 22 |
| Mushemi-Blake et al., 2015 [31] | 2015 | Cohort | 122 | TRJV, 2.5 | 44 |
| Odeyemi et al., 2022 [32] | 2022 | Cross-sectional | 51 | TRJV, 2.5 | 6 |
| Oguanobi et al., 2012 [33] | 2012 | Cross-sectional | 62 | ePASP, 30 | 26 |
| Olatunya et al., 2019 [34] | 2019 | Cohort | 90 | TRJV, 2.5 | 44 |
| Parent et al., 2011 [35] | 2011 | Trial | 398 | TRJV, 2.5 | 109 |
| Ranque et al., 2016 [36] | 2016 | Cohort | 751 | TRJV, 2.5 | 26 |
| Sachdev et al., 2011 [37] | 2011 | Cohort | 435 | TRJV, 2.7 | 177 |
| Sharma et al., 2013 [38] | 2013 | Cohort | 106 | TRJV, 2.5 | 43 |
| van Beers et al., 2008 [39] | 2008 | Cohort | 78 | TRJV, 2.5 | 25 |
| Victor et al., 2016 [40] | 2016 | Cohort | 443 | TRJV, 2.6 | 111 |
| Voskaridou et al., 2007 [41] | 2007 | Cohort | 84 | TRJV, 2.5 | 28 |

** All personal information removed.

**eTable 2.** **Characteristics of the included studies for adults with sickle cell disease.**

| Author | Mild Definition / cut-off | Mild cases | Severe genotype | Lack of blood transfusion during 3 months ago | Hydroxyurea-naive |
| --- | --- | --- | --- | --- | --- |
| Abdul-Mohsen et al., 2012 [1] | N/M | N/M | SS | N/M | N/M |
| Aessopos et al., 2009 [2] | N/M | N/M | N/M | N/M | N/M |
| Afriyie-Mensah et al., 2018 [3] | TRV, 2.5-2.9 | 19 | N/M | N/M | yes |
| Akgül et al., 2007 [4] | N/M | N/M | SS | N/M | N/M |
| Aleem et al., 2007 [5] | ePASP, 31-40 | 19 | SS & Sβ° | N/M | N/M |
| Aliyu et al., 2008 [6] | N/M | N/M | N/M | N/M | yes |
| Amadi et al., 2017 [7] | N/M | N/M | SS | yes | N/M |
| Anjum et al., 2012 [8] | N/M | N/M | SS | N/M | N/M |
| Billy-Brissac et al., 2009 [9] | N/M | N/M | N/M | N/M | N/M |
| Cabrita et al., 2013 [10] | N/M | N/M | N/M | N/M | N/M |
| Caughey et al., 2012 [11] | TRV<3 | 20 | N/M | N/M | N/M |
| Chiadika et al., 2018 [12] | N/M | N/M | N/M | N/M | N/M |
| Damy et al., 2016 [13] | N/M | N/M | N/M | N/M | N/M |
| Dei-Adomakoh et al., 2019 [14] | N/M | N/M | N/M | N/M | N/M |
| Delclaux et al., 2005 [15] | ePASP, 30-38 | 13 | N/M | N/M | N/M |
| d'Humières et al., 2021 [16] | N/M | N/M | SS & Sβ° | yes | N/M |
| Dosunmu et al., 2014 [17] | N/M | N/M | SS | N/M | N/M |
| Elmariah et al., 2014 [18] | N/M | N/M | N/M | yes | N/M |
| Fonseca et al., 2012 [19] | N/M | N/M | N/M | N/M | N/M |
| Garrido et al., 2012 [20] | N/M | N/M | N/M | N/M | N/M |
| Guedeney et al., 2018 [21] | N/M | N/M | N/M | N/M | N/M |
| Garadah et al., 2019 [22] | N/M | N/M | SS | yes | N/M |
| Kato et al., 2006 [23] | TRV, 2.5-2.9 | 51 | N/M | N/M | N/M |
| Klings et al., 2008 [24] | TRV, 2.5-2.9 | 20 | N/M | N/M | N/M |
| Knight-Perry et al., 2011 [25] | N/M | N/M | N/M | N/M | N/M |
| Lobo et al., 2015 [26] | N/M | N/M | SS & Sβ° | N/M | N/M |
| Lopes et al., 2017 [27] | N/M | N/M | N/M | yes | N/M |
| Maioli et al., 2016 [28] | N/M | N/M | N/M | yes | N/M |
| Maikap et al., 2023 [29] | TRV, 2.5-2.9 | 28 | SS | N/M | N/M |
| de Lima Marinho et al., 2016 [30] | N/M | N/M | N/M | yes | N/M |
| Mushemi-Blake et al., 2015 [31] | N/M | N/M | N/M | N/M | N/M |
| Odeyemi et al., 2022 [32] | N/M | N/M | N/M | yes | N/M |
| Oguanobi et al., 2012 [33] | N/M | N/M | SS | N/M | N/M |
| Olatunya et al., 2019 [34] | N/M | N/M | N/M | yes | N/M |
| Parent et al., 2011 [35] | N/M | N/M | SS & Sβ° | N/M | N/M |
| Ranque et al., 2016 [36] | N/M | N/M | N/M | N/M | N/M |
| Sachdev et al., 2011 [37] | N/M | N/M | N/M | N/M | N/M |
| Sharma et al., 2013 [38] | N/M | N/M | SS | N/M | N/M |
| van Beers et al., 2008 [39] | TRV, 2.5-2.9 | 23 | N/M | yes | N/M |
| Victor et al., 2016 [40] | N/M | N/M | N/M | N/M | N/M |
| Voskaridou et al., 2007 [41] | N/M | N/M | N/M | N/M | N/M |

** All personal information removed.

**eTable 3.** **Characteristics of the included studies for children with sickle cell disease.**

| Author | Year of publication | Study design | Total case | Definition / cut-off | Elevated ePASP cases |
| --- | --- | --- | --- | --- | --- |
| AboHadeed et al., 2015 [42] | 2015 | Cross-sectional | 97 | TRJV, 2.5 | 12 |
| Adly et al., 2016 [43] | 2016 | Cross-sectional | 35 | TRJV, 2.5 | 11 |
| Agha et al., 2014 [44] | 2014 | Cohort | 80 | TRJV, 2.5 | 23 |
| Allen et al., 2019 [45] | 2019 | Cross-sectional | 105 | TRJV, 2.5 | 32 |
| Al-Allawi et al., 2016 [46] | 2016 | Cross-sectional | 94 | TRJV, 2.5 | 19 |
| Blanc et al., 2012 [47] | 2012 | Cohort | 18 | TRJV, 2.5 | 6 |
| Caldas et al., 2008 [48] | 2008 | Cohort | 92 | Not specified | 12 |
| Chaudry et al., 2011 [49] | 2011 | Cohort | 50 | TRJV, 2.5 | 16 |
| Chinawa et al., 2020 [50] | 2020 | Cross-sectional | 51 | TRJV, 2.5 | 13 |
| Colombatti et al., 2010 [51] | 2010 | Cohort | 37 | TRJV, 2.5 | 8 |
| Cox et al., 2014 [52] | 2014 | Cohort | 188 | TRJV, 2.5 | 27 |
| Dahoui et al., 2010 [53] | 2010 | Cohort | 85 | TRJV, 2.5 | 27 |
| ElAlfy et al., 2019 [54] | 2019 | Case-control | 46 | TRJV, 2.5 | 8 |
| Eddine et al., 2012 [55] | 2012 | Cohort | 40 | TRJV, 2.5 | 12 |
| Elbarbary et al., 2016 [56] | 2016 | Cross-sectional | 40 | TRJV, 2.5 | 8 |
| El‐Shanshory et al., 2013 [57] | 2013 | Cross-sectional | 30 | TRJV, 2.5 | 9 |
| Forrest et al., 2012 [58] | 2012 | Cohort | 85 | TRJV, 2.5 | 28 |
| Garnier et al., 2017 [59] | 2017 | Cohort | 102 | TRJV, 2.5 | 19 |
| Gordeuk et al., 2009 [60] | 2009 | Cohort | 372 | TRJV, 2.5 | 81 |
| Hanna et al., 2021 [61] | 2021 | case-control | 40 | TRV, 2.5 | 12 |
| Hebson et al., 2015 [62] | 2015 | Cohort | 630 | TRJV, 2.5 | 120 |
| Johnson et al., 2010 [63] | 2010 | Cross-sectional | 40 | TRJV, 2.5 | 4 |
| Lamina et al., 2019 [64] | 2019 | Cross-sectional | 200 | TRJV, 2.5 | 16 |
| Lee et al., 2009 [65] | 2009 | Cohort | 88 | TRJV, 2.5 | 18 |
| Liem et al., 2009 [66] | 2009 | Cohort | 76 | TRJV, 2.5 | 23 |
| Lilje et al., 2017[67] | 2017 | Cohort | 91 | TRJV, 2.5 | 23 |
| Marouf et al., 2014 [68] | 2013 | Case-control | 57 | TRJV, 2.5 | 14 |
| Minniti et al., 2009 [69] | 2009 | Cohort | 290 | TRJV, 2.6 | 32 |
| Nelson et al., 2007 [70] | 2007 | Cohort | 53 | TRJV, 2.5 | 15 |
| Odeyemi et al., 2022 [32] | 2022 | Cross-sectional | 62 | TRJV, 2.5 | 1 |
| Pashankar et al., 2008 [71] | 2008 | Cohort | 62 | TRJV, 2.5 | 19 |
| Peter et al., 2019 [72] | 2019 | Cross-sectional | 100 | TRJV, 2.5 | 22 |
| Ranque et al., 2016 [36] | 2016 | Cohort | 2456 | TRJV, 2.5 | 138 |
| Ribera et al., 2015 [73] | 2015 | Cross-sectional | 36 | TRJV, 2.5 | 7 |
| Sedrak et al., 2009 [74] | 2009 | Cohort | 48 | TRJV, 2.5 | 4 |
| Sokunbi et al., 2017 [75] | 2017 | Cross-sectional | 175 | TRJV, 2.5 | 40 |
| Tantawy et al., 2012 [76] | 2012 | Cohort | 60 | TRJV, 2.5 | 22 |
| Zilberman et al., 2007 [77] | 2007 | Cohort | 45 | TRJV, 2.5 | 25 |

** All personal information removed.

**eTable 4.** **Characteristics of the included studies for children with sickle cell disease.**

| Author | Mild Definition / cut-off | Mild cases | Moderate to Severe definition / cut-off | Moderate to Severe cases | Severe genotype | Lack of blood transfusion during 3 months ago | Hydroxyurea-naive |
| --- | --- | --- | --- | --- | --- | --- | --- |
| AboHadeed et al., 2015 [42] | N/M | N/M | N/M | N/M | SS | N/M | N/M |
| Adly et al., 2016 [43] | N/M | N/M | N/M | N/M | SS & Sβ° | N/M | N/M |
| Agha et al., 2014 [44] | TRV, 2.5-2.9 | 21 | TRV≥3 | 2 | N/M | yes | N/M |
| Allen et al., 2019 [45] | N/M | N/M | N/M | N/M | N/M | N/M | N/M |
| Al-Allawi et al., 2016 [46] | N/M | N/M | N/M | N/M | SS | N/M | yes |
| Blanc et al., 2012 [47] | N/M | N/M | N/M | N/M | N/M | N/M | N/M |
| Caldas et al., 2008 [48] | N/M | N/M | N/M | N/M | Not specified (severe) | N/M | N/M |
| Chaudry et al., 2011 [49] | N/M | N/M | N/M | N/M | N/M | N/M | N/M |
| Chinawa et al., 2020 [50] | N/M | N/M | N/M | N/M | SS | yes | N/M |
| Colombatti et al., 2010 [51] | N/M | N/M | N/M | N/M | N/M | N/M | N/M |
| Cox et al., 2014 [52] | N/M | N/M | N/M | N/M | SS | N/M | yes |
| Dahoui et al., 2010 [53] | TRV, 2.5-2.9 | 22 | TRV≥3 | 5 | N/M | N/M | N/M |
| ElAlfy et al., 2019 [54] | N/M | N/M | N/M | N/M | N/M | N/M | N/M |
| Eddine et al., 2012 [55] | Not specified | 12 | N/M | N/M | N/M | N/M | N/M |
| Elbarbary et al., 2016 [56] | N/M | N/M | N/M | N/M | N/M | N/M | N/M |
| El‐Shanshory et al., 2013 [57] | N/M | N/M | N/M | N/M | N/M | N/M | N/M |
| Forrest et al., 2012 [58] | N/M | N/M | N/M | N/M | SS & Sβ° | N/M | N/M |
| Garnier et al., 2017 [59] | N/M | N/M | N/M | N/M | N/M | yes | N/M |
| Gordeuk et al., 2009 [60] | N/M | N/M | N/M | N/M | N/M | N/M | N/M |
| Hanna et al., 2021 [61] | N/M | N/M | N/M | N/M | N/M | N/M | N/M |
| Hebson et al., 2015 [62] | N/M | N/M | N/M | N/M | N/M | N/M | N/M |
| Johnson et al., 2010 [63] | N/M | N/M | N/M | N/M | SS | N/M | yes |
| Lamina et al., 2019 [64] | TRV, 2.5-2.9 | 15 | TRV≥3 | 1 | SS | yes | yes |
| Lee et al., 2009 [65] | N/M | N/M | N/M | N/M | N/M | N/M | N/M |
| Liem et al., 2009 [66] | N/M | N/M | N/M | N/M | N/M | yes | N/M |
| Lilje et al., 2017[67] | N/M | N/M | N/M | N/M | N/M | N/M | N/M |
| Marouf et al., 2014 [68] | TRV, 2.6-2.9 | 3 | TRV≥3 | 11 | SS & Sβ° | N/M | N/M |
| Minniti et al., 2009 [69] | N/M | N/M | N/M | N/M | N/M | N/M | N/M |
| Nelson et al., 2007 [70] | TRV, 2.5-2.9 | 12 | TRV≥3 | 3 | N/M | N/M | N/M |
| Odeyemi et al., 2022 [32] | N/M | N/M | N/M | N/M | N/M | yes | N/M |
| Pashankar et al., 2008 [71] | TRV, 2.5-2.9 | 13 | TRV≥3 | 6 | SS & Sβ° | N/M | N/M |
| Peter et al., 2019 [72] | N/M | N/M | N/M | N/M | SS | N/M | yes |
| Ranque et al., 2016 [36] | N/M | N/M | N/M | N/M | N/M | N/M | N/M |
| Ribera et al., 2015 [73] | N/M | N/M | N/M | N/M | N/M | N/M | N/M |
| Sedrak et al., 2009 [74] | N/M | N/M | N/M | N/M | N/M | N/M | N/M |
| Sokunbi et al., 2017 [75] | N/M | N/M | N/M | N/M | SS | N/M | N/M |
| Tantawy et al., 2012 [76] | N/M | N/M | N/M | N/M | N/M | N/M | N/M |
| Zilberman et al., 2007 [77] | N/M | N/M | N/M | N/M | N/M | N/M | N/M |

** All personal information removed.

**eTable 5. Clinical and laboratory findings of adult SCD patients comparing elevated ePASP to those without.**

| Author | Year | No. Hb 1 | Hb 1 | No. Hb 2 | Hb 2 | No. Hb 3 | Hb 3 | No. 6MWD 1 | 6MWD 1 | No. 6MWD 2 | 6MWD 2 | No. 6MWD 3 | 6MWD 3 |
| --- | --- | --- | --- | --- | --- | --- | --- | --- | --- | --- | --- | --- | --- |
| Abdul-Mohsen | 2012 | N/M | N/M | N/M | N/M | N/M | N/M | N/M | N/M | N/M | N/M | N/M | N/M |
| Aessopos | 2009 | N/M | N/M | N/M | N/M | N/M | N/M | N/M | N/M | N/M | N/M | N/M | N/M |
| Afriyie-Mensah | 2018 | 25 | 6.9 | N/M | N/M | 51 | 8.6 | N/M | N/M | N/M | N/M | N/M | N/M |
| Akgül | 2007 | 33 | 8.7 | N/M | N/M | 54 | 9.6 | N/M | N/M | N/M | N/M | N/M | N/M |
| Aleem | 2007 | N/M | N/M | N/M | N/M | N/M | N/M | N/M | N/M | N/M | N/M | N/M | N/M |
| Aliyu | 2008 | N/M | N/M | N/M | N/M | N/M | N/M | 32 | 380 | 7 | 370 | 120 | 387 |
| Amadi | 2017 | N/M | N/M | N/M | N/M | N/M | N/M | 22 | 380.3 | N/M | N/M | 70 | 474.3 |
| Anjum | 2012 | 14 | 7.99 | N/M | N/M | 22 | 8.8 | 14 | 283 | N/M | N/M | 22 | 577 |
| Billy-Brissac | 2009 | N/M | N/M | N/M | N/M | N/M | N/M | N/M | N/M | N/M | N/M | N/M | N/M |
| Cabrita | 2013 | 48 | 9.1 | N/M | N/M | 116 | 9.9 | N/M | N/M | N/M | N/M | N/M | N/M |
| Caughey | 2012 | N/M | N/M | N/M | N/M | N/M | N/M | N/M | N/M | N/M | N/M | N/M | N/M |
| Chiadika | 2018 | N/M | N/M | N/M | N/M | N/M | N/M | N/M | N/M | N/M | N/M | N/M | N/M |
| Damy | 2016 | N/M | N/M | N/M | N/M | N/M | N/M | N/M | N/M | N/M | N/M | N/M | N/M |
| Dei-Adomakoh | 2019 | N/M | N/M | N/M | N/M | N/M | N/M | N/M | N/M | N/M | N/M | N/M | N/M |
| Delclaux | 2005 | N/M | N/M | N/M | N/M | N/M | N/M | N/M | N/M | N/M | N/M | N/M | N/M |
| d'Humières | 2021 | N/M | N/M | N/M | N/M | N/M | N/M | N/M | N/M | N/M | N/M | N/M | N/M |
| Dosunmu | 2014 | N/M | N/M | N/M | N/M | N/M | N/M | N/M | N/M | N/M | N/M | N/M | N/M |
| Elmariah | 2014 | N/M | N/M | N/M | N/M | N/M | N/M | N/M | N/M | N/M | N/M | N/M | N/M |
| Fonseca | 2012 | 32 | 7.5 | N/M | N/M | 48 | 8.7 | 32 | 475 | N/M | N/M | 48 | 515 |
| Garrido | 2012 | N/M | N/M | N/M | N/M | N/M | N/M | N/M | N/M | N/M | N/M | N/M | N/M |
| Guedeney | 2018 | 36 | 8.6 | N/M | N/M | 79 | 10.6 | N/M | N/M | N/M | N/M | N/M | N/M |
| Garadah | 2019 | N/M | N/M | N/M | N/M | N/M | N/M | N/M | N/M | N/M | N/M | N/M | N/M |
| Kato | 2006 | N/M | N/M | N/M | N/M | N/M | N/M | N/M | N/M | N/M | N/M | N/M | N/M |
| Klings | 2008 | 30 | 8.5 | N/M | N/M | 33 | 9.7 | N/M | N/M | N/M | N/M | N/M | N/M |
| Knight-Perry | 2011 | N/M | N/M | N/M | N/M | N/M | N/M | N/M | N/M | N/M | N/M | N/M | N/M |
| Lobo | 2015 | 43 | 7.8 | N/M | N/M | 82 | 9 | N/M | N/M | N/M | N/M | N/M | N/M |
| Lopes | 2017 | N/M | N/M | N/M | N/M | N/M | N/M | N/M | N/M | N/M | N/M | N/M | N/M |
| Maioli | 2016 | N/M | N/M | N/M | N/M | N/M | N/M | N/M | N/M | N/M | N/M | N/M | N/M |
| Maikap | 2023 | 33 | 8.7 | N/M | N/M | 97 | 8 | N/M | N/M | N/M | N/M | N/M | N/M |
| de Lima Marinho | 2016 | N/M | N/M | N/M | N/M | N/M | N/M | N/M | N/M | N/M | N/M | N/M | N/M |
| Mushemi-Blake | 2015 | 44 | 8.1 | N/M | N/M | 78 | 9.9 | N/M | N/M | N/M | N/M | N/M | N/M |
| Odeyemi | 2022 | N/M | N/M | N/M | N/M | N/M | N/M | N/M | N/M | N/M | N/M | N/M | N/M |
| Oguanobi | 2012 | N/M | N/M | N/M | N/M | N/M | N/M | N/M | N/M | N/M | N/M | N/M | N/M |
| Olatunya | 2019 | 44 | 8.4 | N/M | N/M | 46 | 9.1 | N/M | N/M | N/M | N/M | N/M | N/M |
| Parent | 2011 | N/M | N/M | N/M | N/M | N/M | N/M | N/M | N/M | N/M | N/M | N/M | N/M |
| Ranque | 2016 | N/M | N/M | N/M | N/M | N/M | N/M | N/M | N/M | N/M | N/M | N/M | N/M |
| Sachdev | 2011 | 120 | 8.3 | 52 | 8.4 | 252 | 9 | 120 | 438 | 52 | 409 | 258 | 458 |
| Sharma | 2013 | N/M | N/M | N/M | N/M | N/M | N/M | N/M | N/M | N/M | N/M | N/M | N/M |
| van Beers | 2008 | 25 | 5.4 | N/M | N/M | 53 | 6.2 | N/M | N/M | N/M | N/M | N/M | N/M |
| Victor | 2016 | N/M | N/M | N/M | N/M | N/M | N/M | N/M | N/M | N/M | N/M | N/M | N/M |
| Voskaridou | 2007 | 28 | 8.8 | N/M | N/M | 56 | 9.5 | N/M | N/M | N/M | N/M | N/M | N/M |

SCD: Sickle cell disease, ePASP: Estimated pulmonary artery systolic pressure, Hb: Hemoglobin, 6MWD: six-minute walk distance, N/M; Not mentioned

*Both Hb (g/dl) and 6MWD (meter) 1 and 2 relate to the cut-off values of 1 and 2, respectively (patients with elevated ePASP).

** Both Hb and 6MWD 3 relate to the cut-off values of 3 (patients with normal ePASP).

***** All personal information removed.**

**eTable 5. Continued.** **Clinical and laboratory findings of adult SCD patients comparing elevated ePASP to those without.**

| Author | Year | No. BMI 1 | BMI 1 | No. BMI 2 | BMI 2 | No. BMI 3 | BMI 3 | No. HbF1 | HbF 1 | No.  HbF 2 | HbF 2 | No. Hb F 3 | HbF 3 |
| --- | --- | --- | --- | --- | --- | --- | --- | --- | --- | --- | --- | --- | --- |
| Abdul-Mohsen | 2012 | N/M | N/M | N/M | N/M | N/M | N/M | N/M | N/M | N/M | N/M | N/M | N/M |
| Aessopos | 2009 | N/M | N/M | N/M | N/M | N/M | N/M | N/M | N/M | N/M | N/M | N/M | N/M |
| Afriyie-Mensah | 2018 | N/M | N/M | N/M | N/M | N/M | N/M | N/M | N/M | N/M | N/M | N/M | N/M |
| Akgül | 2007 | 33 | 20 | N/M | N/M | 54 | 18.6 | 33 | 8.1 | N/M | N/M | 54 | 10.9 |
| Aleem | 2007 | N/M | N/M | N/M | N/M | N/M | N/M | N/M | N/M | N/M | N/M | N/M | N/M |
| Aliyu | 2008 | N/M | N/M | N/M | N/M | N/M | N/M | N/M | N/M | N/M | N/M | N/M | N/M |
| Amadi | 2017 | 22 | 19.1 | N/M | N/M | 70 | 18.9 | N/M | N/M | N/M | N/M | N/M | N/M |
| Anjum | 2012 | 14 | 21.3 | N/M | N/M | 22 | 20.8 | 14 | 6.5 | N/M | N/M | 22 | 5.9 |
| Billy-Brissac | 2009 | N/M | N/M | N/M | N/M | N/M | N/M | N/M | N/M | N/M | N/M | N/M | N/M |
| Cabrita | 2013 | 48 | 24 | N/M | N/M | 116 | 25 | 48 | 3.7 | N/M | N/M | 116 | 5.1 |
| Caughey | 2012 | N/M | N/M | N/M | N/M | N/M | N/M | N/M | N/M | N/M | N/M | N/M | N/M |
| Chiadika | 2018 | N/M | N/M | N/M | N/M | N/M | N/M | N/M | N/M | N/M | N/M | N/M | N/M |
| Damy | 2016 | N/M | N/M | N/M | N/M | N/M | N/M | N/M | N/M | N/M | N/M | N/M | N/M |
| Dei-Adomakoh | 2019 | N/M | N/M | N/M | N/M | N/M | N/M | N/M | N/M | N/M | N/M | N/M | N/M |
| Delclaux | 2005 | N/M | N/M | N/M | N/M | N/M | N/M | N/M | N/M | N/M | N/M | N/M | N/M |
| d'Humières | 2021 | N/M | N/M | N/M | N/M | N/M | N/M | N/M | N/M | N/M | N/M | N/M | N/M |
| Dosunmu | 2014 | N/M | N/M | N/M | N/M | N/M | N/M | N/M | N/M | N/M | N/M | N/M | N/M |
| Elmariah | 2014 | N/M | N/M | N/M | N/M | N/M | N/M | N/M | N/M | N/M | N/M | N/M | N/M |
| Fonseca | 2012 | 32 | 20 | N/M | N/M | 48 | 21.1 | 32 | 6.2 | N/M | N/M | 48 | 8.3 |
| Garrido | 2012 | N/M | N/M | N/M | N/M | N/M | N/M | N/M | N/M | N/M | N/M | N/M | N/M |
| Guedeney | 2018 | 36 | 22.3 | N/M | N/M | 79 | 22.4 | N/M | N/M | N/M | N/M | N/M | N/M |
| Garadah | 2019 | N/M | N/M | N/M | N/M | N/M | N/M | N/M | N/M | N/M | N/M | N/M | N/M |
| Kato | 2006 | N/M | N/M | N/M | N/M | N/M | N/M | N/M | N/M | N/M | N/M | N/M | N/M |
| Klings | 2008 | N/M | N/M | N/M | N/M | N/M | N/M | N/M | N/M | N/M | N/M | N/M | N/M |
| Knight-Perry | 2011 | N/M | N/M | N/M | N/M | N/M | N/M | N/M | N/M | N/M | N/M | N/M | N/M |
| Lobo | 2015 | 43 | 20 | N/M | N/M | 82 | 20.4 | 43 | 6.6 | N/M | N/M | 82 | 8.7 |
| Lopes | 2017 | N/M | N/M | N/M | N/M | N/M | N/M | N/M | N/M | N/M | N/M | N/M | N/M |
| Maioli | 2016 | N/M | N/M | N/M | N/M | N/M | N/M | N/M | N/M | N/M | N/M | N/M | N/M |
| Maikap | 2023 | N/M | N/M | N/M | N/M | N/M | N/M | 33 | 20.3 |  |  | 97 | 22.5 |
| de Lima Marinho | 2016 | N/M | N/M | N/M | N/M | N/M | N/M | N/M | N/M | N/M | N/M | N/M | N/M |
| Mushemi-Blake | 2015 | N/M | N/M | N/M | N/M | N/M | N/M | N/M | N/M | N/M | N/M | N/M | N/M |
| Odeyemi | 2022 | 7 | 20.8 | N/M | N/M | 106 | 17.4 | N/M | N/M | N/M | N/M | N/M | N/M |
| Oguanobi | 2012 | 26 | 19.9 | N/M | N/M | 36 | 21.4 | N/M | N/M | N/M | N/M | N/M | N/M |
| Olatunya | 2019 | N/M | N/M | N/M | N/M | N/M | N/M | 44 | 13.1 | N/M | N/M | 46 | 12.9 |
| Parent | 2011 | N/M | N/M | N/M | N/M | N/M | N/M | N/M | N/M | N/M | N/M | N/M | N/M |
| Ranque | 2016 | N/M | N/M | N/M | N/M | N/M | N/M | N/M | N/M | N/M | N/M | N/M | N/M |
| Sachdev | 2011 | 122 | 23.8 | 50 | 24.1 | 254 | 22.9 | 110 | 5.3 | 48 | 4.3 | 231 | 6 |
| Sharma | 2013 | N/M | N/M | N/M | N/M | N/M | N/M | N/M | N/M | N/M | N/M | N/M | N/M |
| van Beers | 2008 | N/M | N/M | N/M | N/M | N/M | N/M | 25 | 6.96 | N/M | N/M | 53 | 7.12 |
| Victor | 2016 | N/M | N/M | N/M | N/M | N/M | N/M | N/M | N/M | N/M | N/M | N/M | N/M |
| Voskaridou | 2007 | N/M | N/M | N/M | N/M | N/M | N/M | 28 | 19.4 | N/M | N/M | 56 | 16.3 |

SCD: Sickle cell disease, ePASP: Estimated pulmonary artery systolic pressure, BMI: Body mass index, HbF: Fetal hemoglobin, N/M; Not mentioned

*Both BMI (Kg/m^2^) and HbF (%) 1 and 2 relate to the cut-off values of 1 and 2, respectively (patients with elevated ePASP).

** Both BMI and HbF 3 relate to the cut-off values of 3 (patients with normal ePASP).

**eTable 5. Continued.** **Laboratory findings of adult SCD patients comparing elevated ePASP to those without.**

| Author | Year | No. WBC 1 | WBC 1 | No. WBC 2 | WBC 2 | No. WBC 3 | WBC 3 | No. Plt 1 | Plt 1 | No. Plt 2 | Plt 2 | No. Plt 3 | Plt 3 |
| --- | --- | --- | --- | --- | --- | --- | --- | --- | --- | --- | --- | --- | --- |
| Abdul-Mohsen | 2012 | N/M | N/M | N/M | N/M | N/M | N/M | N/M | N/M | N/M | N/M | N/M | N/M |
| Aessopos | 2009 | N/M | N/M | N/M | N/M | N/M | N/M | N/M | N/M | N/M | N/M | N/M | N/M |
| Afriyie-Mensah | 2018 | 25 | 10.3 | N/M | N/M | 51 | 10 | 25 | 362.6 | N/M | N/M | 51 | 341.8 |
| Akgül | 2007 | 33 | 11.2 | N/M | N/M | 54 | 10.8 | 33 | 438.5 | N/M | N/M | 54 | 418.2 |
| Aleem | 2007 | N/M | N/M | N/M | N/M | N/M | N/M | N/M | N/M | N/M | N/M | N/M | N/M |
| Aliyu | 2008 | N/M | N/M | N/M | N/M | N/M | N/M | N/M | N/M | N/M | N/M | N/M | N/M |
| Amadi | 2017 | N/M | N/M | N/M | N/M | N/M | N/M | N/M | N/M | N/M | N/M | N/M | N/M |
| Anjum | 2012 | 14 | 10.5 | N/M | N/M | 22 | 10.5 | 14 | 425.7 | N/M | N/M | 22 | 410.5 |
| Billy-Brissac | 2009 | N/M | N/M | N/M | N/M | N/M | N/M | N/M | N/M | N/M | N/M | N/M | N/M |
| Cabrita | 2013 | 48 | 9.4 | N/M | N/M | 116 | 9.2 | 48 | 298 | N/M | N/M | 116 | 307.7 |
| Caughey | 2012 | N/M | N/M | N/M | N/M | N/M | N/M | N/M | N/M | N/M | N/M | N/M | N/M |
| Chiadika | 2018 | N/M | N/M | N/M | N/M | N/M | N/M | N/M | N/M | N/M | N/M | N/M | N/M |
| Damy | 2016 | N/M | N/M | N/M | N/M | N/M | N/M | N/M | N/M | N/M | N/M | N/M | N/M |
| Dei-Adomakoh | 2019 | N/M | N/M | N/M | N/M | N/M | N/M | N/M | N/M | N/M | N/M | N/M | N/M |
| Delclaux | 2005 | N/M | N/M | N/M | N/M | N/M | N/M | N/M | N/M | N/M | N/M | N/M | N/M |
| d'Humières | 2021 | N/M | N/M | N/M | N/M | N/M | N/M | N/M | N/M | N/M | N/M | N/M | N/M |
| Dosunmu | 2014 | N/M | N/M | N/M | N/M | N/M | N/M | N/M | N/M | N/M | N/M | N/M | N/M |
| Elmariah | 2014 | N/M | N/M | N/M | N/M | N/M | N/M | N/M | N/M | N/M | N/M | N/M | N/M |
| Fonseca | 2012 | 32 | 12.1 | N/M | N/M | 48 | 11 | 32 | 378 | N/M | N/M | 48 | 427 |
| Garrido | 2012 | N/M | N/M | N/M | N/M | N/M | N/M | N/M | N/M | N/M | N/M | N/M | N/M |
| Guedeney | 2018 | N/M | N/M | N/M | N/M | N/M | N/M | N/M | N/M | N/M | N/M | N/M | N/M |
| Garadah | 2019 | N/M | N/M | N/M | N/M | N/M | N/M | N/M | N/M | N/M | N/M | N/M | N/M |
| Kato | 2006 | N/M | N/M | N/M | N/M | N/M | N/M | N/M | N/M | N/M | N/M | N/M | N/M |
| Klings | 2008 | 30 | 11 | N/M | N/M | 33 | 10.8 | 30 | 365.8 | N/M | N/M | 33 | 336.3 |
| Knight-Perry | 2011 | N/M | N/M | N/M | N/M | N/M | N/M | N/M | N/M | N/M | N/M | N/M | N/M |
| Lobo | 2015 | 43 | 10.7 | N/M | N/M | 82 | 10.1 | 43 | 432 | N/M | N/M | 82 | 410.1 |
| Lopes | 2017 | N/M | N/M | N/M | N/M | N/M | N/M | N/M | N/M | N/M | N/M | N/M | N/M |
| Maioli | 2016 | N/M | N/M | N/M | N/M | N/M | N/M | N/M | N/M | N/M | N/M | N/M | N/M |
| Maikap | 2023 | 33 | 10.3 | N/M | N/M | 97 | 10.8 | 33 | 274.3 | N/M | N/M | 97 | 303.9 |
| de Lima Marinho | 2016 | N/M | N/M | N/M | N/M | N/M | N/M | N/M | N/M | N/M | N/M | N/M | N/M |
| Mushemi-Blake | 2015 | N/M | N/M | N/M | N/M | N/M | N/M | N/M | N/M | N/M | N/M | N/M | N/M |
| Odeyemi | 2022 | 7 | 11 | N/M | N/M | 106 | 13.7 | 7 | 286.6 | N/M | N/M | 106 | 329.8 |
| Oguanobi | 2012 | N/M | N/M | N/M | N/M | N/M | N/M | N/M | N/M | N/M | N/M | N/M | N/M |
| Olatunya | 2019 | 44 | 6.5 | N/M | N/M | 46 | 8.1 | 44 | 335 | N/M | N/M | 46 | 345.7 |
| Parent | 2011 | N/M | N/M | N/M | N/M | N/M | N/M | N/M | N/M | N/M | N/M | N/M | N/M |
| Ranque | 2016 | N/M | N/M | N/M | N/M | N/M | N/M | N/M | N/M | N/M | N/M | N/M | N/M |
| Sachdev | 2011 | 120 | 9.6 | 52 | 10.2 | 253 | 9.4 | 120 | 355 | 52 | 333.3±54.9 | 252 | 361 |
| Sharma | 2013 | N/M | N/M | N/M | N/M | N/M | N/M | N/M | N/M | N/M | N/M | N/M | N/M |
| van Beers | 2008 | 25 | 8.8 | N/M | N/M | 53 | 8.2 | N/M | N/M | N/M | N/M | N/M | N/M |
| Victor | 2016 | N/M | N/M | N/M | N/M | N/M | N/M | N/M | N/M | N/M | N/M | N/M | N/M |
| Voskaridou | 2007 | N/M | N/M | N/M | N/M | N/M | N/M | N/M | N/M | N/M | N/M | N/M | N/M |

SCD: Sickle cell disease, ePASP: Estimated pulmonary artery systolic pressure, WBC: White Blood Cell, Plt: Platelet, N/M; Not mentioned

*Both WBC (×10^3^/ml) and Plt (×10^3^/ml) 1 and 2 relate to the cut-off values of 1 and 2, respectively (patients with elevated ePASP).

** Both WBC and Plt 3 relate to the cut-off value of 3 (patients with normal ePASP).

**eTable 5. Continued.** **Laboratory findings of adult SCD patients comparing elevated ePASP to those without.**

| Author | Year | No. LDH 1 | LDH 1 | No. LDH 2 | LDH 2 | No. LDH 3 | LDH 3 | No. Retic 1 | Retic 1 | No. Retic 2 | Retic 2 | No. Retic 3 | Retic 3 |
| --- | --- | --- | --- | --- | --- | --- | --- | --- | --- | --- | --- | --- | --- |
| Abdul-Mohsen | 2012 | N/M | N/M | N/M | N/M | N/M | N/M | N/M | N/M | N/M | N/M | N/M | N/M |
| Aessopos | 2009 | N/M | N/M | N/M | N/M | N/M | N/M | N/M | N/M | N/M | N/M | N/M | N/M |
| Afriyie-Mensah | 2018 | N/M | N/M | N/M | N/M | N/M | N/M | N/M | N/M | N/M | N/M | N/M | N/M |
| Akgül | 2007 | 33 | 1070.5 | N/M | N/M | 54 | 683.8 | 33 | 10.1 | N/M | N/M | 54 | 9.4 |
| Aleem | 2007 | N/M | N/M | N/M | N/M | N/M | N/M | N/M | N/M | N/M | N/M | N/M | N/M |
| Aliyu | 2008 | N/M | N/M | N/M | N/M | N/M | N/M | N/M | N/M | N/M | N/M | N/M | N/M |
| Amadi | 2017 | N/M | N/M | N/M | N/M | N/M | N/M | N/M | N/M | N/M | N/M | N/M | N/M |
| Anjum | 2012 | 14 | 548 | N/M | N/M | 22 | 436.8 | 14 | 24.8 | N/M | N/M | 22 | 11.6 |
| Billy-Brissac | 2009 | N/M | N/M | N/M | N/M | N/M | N/M | N/M | N/M | N/M | N/M | N/M | N/M |
| Cabrita | 2013 | 48 | 759 | N/M | N/M | 116 | 577.7 | 48 | 21.6 | N/M | N/M | 116 | 21.4 |
| Caughey | 2012 | N/M | N/M | N/M | N/M | N/M | N/M | N/M | N/M | N/M | N/M | N/M | N/M |
| Chiadika | 2018 | N/M | N/M | N/M | N/M | N/M | N/M | N/M | N/M | N/M | N/M | N/M | N/M |
| Damy | 2016 | N/M | N/M | N/M | N/M | N/M | N/M | N/M | N/M | N/M | N/M | N/M | N/M |
| Dei-Adomakoh | 2019 | N/M | N/M | N/M | N/M | N/M | N/M | N/M | N/M | N/M | N/M | N/M | N/M |
| Delclaux | 2005 | N/M | N/M | N/M | N/M | N/M | N/M | N/M | N/M | N/M | N/M | N/M | N/M |
| d'Humières | 2021 | N/M | N/M | N/M | N/M | N/M | N/M | N/M | N/M | N/M | N/M | N/M | N/M |
| Dosunmu | 2014 | N/M | N/M | N/M | N/M | N/M | N/M | N/M | N/M | N/M | N/M | N/M | N/M |
| Elmariah | 2014 | N/M | N/M | N/M | N/M | N/M | N/M | N/M | N/M | N/M | N/M | N/M | N/M |
| Fonseca | 2012 | 32 | 1427 | N/M | N/M | 48 | 909 | 32 | 11.8 | N/M | N/M | 48 | 10.9 |
| Garrido | 2012 | N/M | N/M | N/M | N/M | N/M | N/M | N/M | N/M | N/M | N/M | N/M | N/M |
| Guedeney | 2018 | 36 | 380.8 | N/M | N/M | 79 | 279.2 | N/M | N/M | N/M | N/M | N/M | N/M |
| Garadah | 2019 | N/M | N/M | N/M | N/M | N/M | N/M | N/M | N/M | N/M | N/M | N/M | N/M |
| Kato | 2006 | N/M | N/M | N/M | N/M | N/M | N/M | N/M | N/M | N/M | N/M | N/M | N/M |
| Klings | 2008 | N/M | N/M | N/M | N/M | N/M | N/M | N/M | N/M | N/M | N/M | N/M | N/M |
| Knight-Perry | 2011 | N/M | N/M | N/M | N/M | N/M | N/M | N/M | N/M | N/M | N/M | N/M | N/M |
| Lobo | 2015 | 43 | 1064.8 | N/M | N/M | 82 | 837 | 43 | 12.1 | N/M | N/M | 82 | 10.4 |
| Lopes | 2017 | N/M | N/M | N/M | N/M | N/M | N/M | N/M | N/M | N/M | N/M | N/M | N/M |
| Maioli | 2016 | N/M | N/M | N/M | N/M | N/M | N/M | N/M | N/M | N/M | N/M | N/M | N/M |
| Maikap | 2023 | 33 | 974.5 | N/M | N/M | 97 | 572.6 | N/M | N/M | N/M | N/M | N/M | N/M |
| de Lima Marinho | 2016 | N/M | N/M | N/M | N/M | N/M | N/M | N/M | N/M | N/M | N/M | N/M | N/M |
| Mushemi-Blake | 2015 | 44 | 441 | N/M | N/M | 78 | 326 | 44 | 30.9 | N/M | N/M | 78 | 30.3 |
| Odeyemi | 2022 | N/M | N/M | N/M | N/M | N/M | N/M | N/M | N/M | N/M | N/M | N/M | N/M |
| Oguanobi | 2012 | N/M | N/M | N/M | N/M | N/M | N/M | N/M | N/M | N/M | N/M | N/M | N/M |
| Olatunya | 2019 | 44 | 409.7 | N/M | N/M | 46 | 344.3 | 44 | 26.1 | N/M | N/M | 46 | 24.3 |
| Parent | 2011 | N/M | N/M | N/M | N/M | N/M | N/M | N/M | N/M | N/M | N/M | N/M | N/M |
| Ranque | 2016 | N/M | N/M | N/M | N/M | N/M | N/M | N/M | N/M | N/M | N/M | N/M | N/M |
| Sachdev | 2011 | 110 | 452.7 | 49 | 501 | 235 | 404 | 117 | 9.4 | 51 | 9.5 | 231 | 8.2 |
| Sharma | 2013 | N/M | N/M | N/M | N/M | N/M | N/M | N/M | N/M | N/M | N/M | N/M | N/M |
| van Beers | 2008 | 25 | 583.7 | N/M | N/M | 53 | 353.5 | N/M | N/M | N/M | N/M | N/M | N/M |
| Victor | 2016 | N/M | N/M | N/M | N/M | N/M | N/M | N/M | N/M | N/M | N/M | N/M | N/M |
| Voskaridou | 2007 | 28 | 872.7 | N/M | N/M | 56 | 855.7 | 28 | 41.1 | N/M | N/M | 56 | 28.9 |

SCD: Sickle cell disease, ePASP: Estimated pulmonary artery systolic pressure, LDH: Lactate Dehydrogenase, N/M; Not mentioned

*Both LDH (U/l) and retic (%) 1 and 2 relate to the cut-off values of 1 and 2, respectively (patients with elevated ePASP).

** Both LDH and retic 3 relate to the cut-off values of 3 (patients with normal ePASP).

**eTable 5. Continued.** **Clinical findings of adult SCD patients comparing elevated ePASP to those without.**

| Author | Year | No. O2 sat 1 | O2sat 1 | No. O2 sat 3 | O2sat 3 |
| --- | --- | --- | --- | --- | --- |
| Abdul-Mohsen | 2012 | N/M | N/M | N/M | N/M |
| Aessopos | 2009 | N/M | N/M | N/M | N/M |
| Afriyie-Mensah | 2018 | 25 | 92 | 51 | 94 |
| Akgül | 2007 | N/M | N/M | N/M | N/M |
| Aleem | 2007 | N/M | N/M | N/M | N/M |
| Aliyu | 2008 | N/M | N/M | N/M | N/M |
| Amadi | 2017 | N/M | N/M | N/M | N/M |
| Anjum | 2012 | N/M | N/M | N/M | N/M |
| Billy-Brissac | 2009 | N/M | N/M | N/M | N/M |
| Cabrita | 2013 | N/M | N/M | N/M | N/M |
| Caughey | 2012 | N/M | N/M | N/M | N/M |
| Chiadika | 2018 | N/M | N/M | N/M | N/M |
| Damy | 2016 | N/M | N/M | N/M | N/M |
| Dei-Adomakoh | 2019 | N/M | N/M | N/M | N/M |
| Delclaux | 2005 | N/M | N/M | N/M | N/M |
| d'Humières | 2021 | N/M | N/M | N/M | N/M |
| Dosunmu | 2014 | N/M | N/M | N/M | N/M |
| Elmariah | 2014 | N/M | N/M | N/M | N/M |
| Fonseca | 2012 | 32 | 90 | 48 | 93 |
| Garrido | 2012 | N/M | N/M | N/M | N/M |
| Guedeney | 2018 | N/M | N/M | N/M | N/M |
| Garadah | 2019 | N/M | N/M | N/M | N/M |
| Kato | 2006 | N/M | N/M | N/M | N/M |
| Klings | 2008 | N/M | N/M | N/M | N/M |
| Knight-Perry | 2011 | N/M | N/M | N/M | N/M |
| Lobo | 2015 | N/M | N/M | N/M | N/M |
| Lopes | 2017 | N/M | N/M | N/M | N/M |
| Maioli | 2016 | N/M | N/M | N/M | N/M |
| Maikap | 2023 | N/M | N/M | N/M | N/M |
| de Lima Marinho | 2016 | N/M | N/M | N/M | N/M |
| Mushemi-Blake | 2015 | 44 | 94 | 78 | 96 |
| Odeyemi | 2022 | 7 | 93 | 106 | 94.4 |
| Oguanobi | 2012 | N/M | N/M | N/M | N/M |
| Olatunya | 2019 | N/M | N/M | N/M | N/M |
| Parent | 2011 | N/M | N/M | N/M | N/M |
| Ranque | 2016 | N/M | N/M | N/M | N/M |
| Sachdev | 2011 | N/M | N/M | N/M | N/M |
| Sharma | 2013 | N/M | N/M | N/M | N/M |
| van Beers | 2008 | N/M | N/M | N/M | N/M |
| Victor | 2016 | N/M | N/M | N/M | N/M |
| Voskaridou | 2007 | N/M | N/M | N/M | N/M |

SCD: Sickle cell disease, ePASP: Estimated pulmonary artery systolic pressure, O2sat: O2 saturation, N/M; Not mentioned

*O2 saturation (%) 1 relates to the cut-off value of 1 (patients with elevated ePASP).

**O2 saturation 3 relates to the cut-off value of 3 (patients with normal ePASP).

**eTable 6. Laboratory findings of children comparing elevated ePASP to those without.**

| Author | Year | No. Hb 1 | Hb 1 | No. Hb 2 | Hb 2 | No. Hb 3 | Hb 3 | No. HbF 1 | HbF 1 | No. HbF 2 | HbF 2 | No. HbF 3 | HbF 3 |
| --- | --- | --- | --- | --- | --- | --- | --- | --- | --- | --- | --- | --- | --- |
| AboHadeed | 2015 | N/M | N/M | N/M | N/M | N/M | N/M | N/M | N/M | N/M | N/M | N/M | N/M |
| Adly | 2016 | N/M | N/M | N/M | N/M | N/M | N/M | N/M | N/M | N/M | N/M | N/M | N/M |
| Agha | 2014 | 23 | 7.9 | N/M | N/M | 57 | 7.9 | 23 | 12.3 | N/M | N/M | 57 | 14.4 |
| Allen | 2019 | 32 | 9 | N/M | N/M | 73 | 9.5 | N/M | N/M | N/M | N/M | N/M | N/M |
| Al-Allawi | 2016 | 10 | 8.6 | N/M | N/M | 84 | 8.5 | 10 | 9.3 | N/M | N/M | 84 | 14.1 |
| Blanc | 2012 | N/M | N/M | N/M | N/M | N/M | N/M | N/M | N/M | N/M | N/M | N/M | N/M |
| Caldas | 2008 | N/M | N/M | N/M | N/M | N/M | N/M | N/M | N/M | N/M | N/M | N/M | N/M |
| Chaudry | 2011 | N/M | N/M | N/M | N/M | N/M | N/M | 16 | 6.4 | N/M | N/M | 34 | 3.3 |
| Chinawa | 2020 | N/M | N/M | N/M | N/M | N/M | N/M | N/M | N/M | N/M | N/M | N/M | N/M |
| Colombatti | 2010 | 8 | 8.2 | N/M | N/M | 29 | 8.3 | 8 | 9.7 | N/M | N/M | 29 | 9.6 |
| Cox | 2014 | 22 | 6.9 | 5 | 5.8 | 161 | 7.3 | 19 | 6 | 5 | 2.8 | 158 | 4.6 |
| Dahoui | 2010 | 27 | 8.8 | N/M | N/M | 58 | 9.4 | 27 | 15.6 | N/M | N/M | 58 | 13.4 |
| ElAlfy | 2019 | N/M | N/M | N/M | N/M | N/M | N/M | N/M | N/M | N/M | N/M | N/M | N/M |
| Eddine | 2012 | N/M | N/M | N/M | N/M | N/M | N/M | N/M | N/M | N/M | N/M | N/M | N/M |
| Elbarbary | 2016 | N/M | N/M | N/M | N/M | N/M | N/M | N/M | N/M | N/M | N/M | N/M | N/M |
| El‐Shanshory | 2013 | 9 | 7.4 | N/M | N/M | 21 | 7.5 | N/M | N/M | N/M | N/M | N/M | N/M |
| Forrest | 2012 | 28 | 8.3 | N/M | N/M | 57 | 8.5 | N/M | N/M | N/M | N/M | N/M | N/M |
| Garnier | 2017 | N/M | N/M | N/M | N/M | N/M | N/M | N/M | N/M | N/M | N/M | N/M | N/M |
| Gordeuk | 2009 | N/M | N/M | N/M | N/M | N/M | N/M | N/M | N/M | N/M | N/M | N/M | N/M |
| Hanna | 2021 | N/M | N/M | N/M | N/M | N/M | N/M | N/M | N/M | N/M | N/M | N/M | N/M |
| Hebson | 2015 | 120 | 8.9 | N/M | N/M | 120 | 9.8 | N/M | N/M | N/M | N/M | N/M | N/M |
| Johnson | 2010 | N/M | N/M | N/M | N/M | N/M | N/M | N/M | N/M | N/M | N/M | N/M | N/M |
| Lamina | 2019 | N/M | N/M | N/M | N/M | N/M | N/M | N/M | N/M | N/M | N/M | N/M | N/M |
| Lee | 2009 | 18 | 8.9 | N/M | N/M | 70 | 9.5 | N/M | N/M | N/M | N/M | N/M | N/M |
| Liem | 2009 | N/M | N/M | N/M | N/M | N/M | N/M | N/M | N/M | N/M | N/M | N/M | N/M |
| Lilje | 2017 | 18 | 8.5 | 5 | 7.9 | 68 | 9.5 | N/M | N/M | N/M | N/M | N/M | N/M |
| Marouf | 2013 | 8 | 9.3 | N/M | N/M | 49 | 10.7 | N/M | N/M | N/M | N/M | N/M | N/M |
| Minniti | 2009 | 30 | 8.9 | N/M | N/M | 251 | 9.4 | N/M | N/M | N/M | N/M | N/M | N/M |
| Nelson | 2007 | 15 | 8.6 | N/M | N/M | 38 | 9.4 | 15 | 10.8 | N/M | N/M | 38 | 12.3 |
| Odeyemi | 2022 | N/M | N/M | N/M | N/M | N/M | N/M | N/M | N/M | N/M | N/M | N/M | N/M |
| Pashankar | 2008 | 13 | 8.4 | 6 | 7.6 | 43 | 8.7 | N/M | N/M | N/M | N/M | N/M | N/M |
| Peter | 2019 | N/M | N/M | N/M | N/M | N/M | N/M | 22 | 8.2 | N/M | N/M | 78 | 9.9 |
| Ranque | 2016 | N/M | N/M | N/M | N/M | N/M | N/M | N/M | N/M | N/M | N/M | N/M | N/M |
| Ribera | 2015 | N/M | N/M | N/M | N/M | N/M | N/M | N/M | N/M | N/M | N/M | N/M | N/M |
| Sedrak | 2009 | 4 | 7.6 | N/M | N/M | 44 | 9.2 | N/M | N/M | N/M | N/M | N/M | N/M |
| Sokunbi | 2017 | 40 | 7.7 | N/M | N/M | 135 | 7.9 | 40 | 7.6 | N/M | N/M | 135 | 6.5 |
| Tantawy | 2012 | N/M | N/M | N/M | N/M | N/M | N/M | N/M | N/M | N/M | N/M | N/M | N/M |
| Zilberman | 2007 | N/M | N/M | N/M | N/M | N/M | N/M | N/M | N/M | N/M | N/M | N/M | N/M |

SCD: Sickle cell disease, ePASP: Estimated pulmonary artery systolic pressure, Hb: Hemoglobin , Hb F: Fetal hemoglobin, N/M; Not mentioned

*Both Hb (g/dl) and HbF (%) 1 and 2 relate to the cut-off values of 1 and 2, respectively (patients with elevated ePASP).

** Both Hb and Hb F 3 relate to the cut-off value of 3 (patients with normal ePASP).

***** All personal information removed.**

**eTable 6. Continued.** **Laboratory findings of children comparing elevated ePASP to those without.**

| Author | Year | No. WBC 1 | WBC 1 | No. WBC 2 | WBC 2 | No. WBC 3 | WBC 3 | No. Plt 1 | Plt 1 | No. Plt 2 | Plt 2 | No. Plt 3 | Plt 3 |
| --- | --- | --- | --- | --- | --- | --- | --- | --- | --- | --- | --- | --- | --- |
| AboHadeed | 2015 | N/M | N/M | N/M | N/M | N/M | N/M | N/M | N/M | N/M | N/M | N/M | N/M |
| Adly | 2016 | N/M | N/M | N/M | N/M | N/M | N/M | N/M | N/M | N/M | N/M | N/M | N/M |
| Agha | 2014 | 23 | 13 | N/M | N/M | 57 | 11.4 | 23 | 489.4 | N/M | N/M | 57 | 388.7 |
| Allen | 2019 | N/M | N/M | N/M | N/M | N/M | N/M | N/M | N/M | N/M | N/M | N/M | N/M |
| Al-Allawi | 2016 | 10 | 15.1 | N/M | N/M | 84 | 14.5 | 10 | 423.3 | N/M | N/M | 84 | 396.1 |
| Blanc | 2012 | N/M | N/M | N/M | N/M | N/M | N/M | N/M | N/M | N/M | N/M | N/M | N/M |
| Caldas | 2008 | N/M | N/M | N/M | N/M | N/M | N/M | N/M | N/M | N/M | N/M | N/M | N/M |
| Chaudry | 2011 | N/M | N/M | N/M | N/M | N/M | N/M | N/M | N/M | N/M | N/M | N/M | N/M |
| Chinawa | 2020 | N/M | N/M | N/M | N/M | N/M | N/M | N/M | N/M | N/M | N/M | N/M | N/M |
| Colombatti | 2010 | 8 | 16.4 | N/M | N/M | 29 | 12.9 | 8 | 556 | N/M | N/M | 29 | 405.8 |
| Cox | 2014 | N/M | N/M | N/M | N/M | N/M | N/M | N/M | N/M | N/M | N/M | N/M | N/M |
| Dahoui | 2010 | N/M | N/M | N/M | N/M | N/M | N/M | N/M | N/M | N/M | N/M | N/M | N/M |
| ElAlfy | 2019 | N/M | N/M | N/M | N/M | N/M | N/M | N/M | N/M | N/M | N/M | N/M | N/M |
| Eddine | 2012 | N/M | N/M | N/M | N/M | N/M | N/M | N/M | N/M | N/M | N/M | N/M | N/M |
| Elbarbary | 2016 | N/M | N/M | N/M | N/M | N/M | N/M | N/M | N/M | N/M | N/M | N/M | N/M |
| El‐Shanshory | 2013 | N/M | N/M | N/M | N/M | N/M | N/M | N/M | N/M | N/M | N/M | N/M | N/M |
| Forrest | 2012 | 28 | 13.5 | N/M | N/M | 57 | 14.2 | 28 | 505.4 | N/M | N/M | 57 | 406.5 |
| Garnier | 2017 | N/M | N/M | N/M | N/M | N/M | N/M | N/M | N/M | N/M | N/M | N/M | N/M |
| Gordeuk | 2009 | N/M | N/M | N/M | N/M | N/M | N/M | N/M | N/M | N/M | N/M | N/M | N/M |
| Hanna | 2021 | N/M | N/M | N/M | N/M | N/M | N/M | N/M | N/M | N/M | N/M | N/M | N/M |
| Hebson | 2015 | N/M | N/M | N/M | N/M | N/M | N/M | N/M | N/M | N/M | N/M | N/M | N/M |
| Johnson | 2010 | N/M | N/M | N/M | N/M | N/M | N/M | N/M | N/M | N/M | N/M | N/M | N/M |
| Lamina | 2019 | N/M | N/M | N/M | N/M | N/M | N/M | N/M | N/M | N/M | N/M | N/M | N/M |
| Lee | 2009 | 18 | 11.3 | N/M | N/M | 70 | 10.6 | 18 | 475 | N/M | N/M | 70 | 398 |
| Liem | 2009 | N/M | N/M | N/M | N/M | N/M | N/M | N/M | N/M | N/M | N/M | N/M | N/M |
| Lilje | 2017 | N/M | N/M | N/M | N/M | N/M | N/M | N/M | N/M | N/M | N/M | N/M | N/M |
| Marouf | 2013 | N/M | N/M | N/M | N/M | N/M | N/M | N/M | N/M | N/M | N/M | N/M | N/M |
| Minniti | 2009 | 30 | 11.2 | N/M | N/M | 252 | 10.3 | N/M | N/M | N/M | N/M | N/M | N/M |
| Nelson | 2007 | 15 | 12.6 | N/M | N/M | 38 | 10.3 | N/M | N/M | N/M | N/M | N/M | N/M |
| Odeyemi | 2022 | N/M | N/M | N/M | N/M | N/M | N/M | N/M | N/M | N/M | N/M | N/M | N/M |
| Pashankar | 2008 | 13 | 13.2 | 6 | 14.4 | 43 | 12.7 | 13 | 498.5 | 6 | 533.6 | 43 | 404.7 |
| Peter | 2019 | 22 | 14.2 | N/M | N/M | 78 | 12.9 | 22 | 374.2 | N/M | N/M | 78 | 362.5 |
| Ranque | 2016 | N/M | N/M | N/M | N/M | N/M | N/M | N/M | N/M | N/M | N/M | N/M | N/M |
| Ribera | 2015 | N/M | N/M | N/M | N/M | N/M | N/M | N/M | N/M | N/M | N/M | N/M | N/M |
| Sedrak | 2009 | 4 | 12.4 | N/M | N/M | 44 | 11.4 | N/M | N/M | N/M | N/M | N/M | N/M |
| Sokunbi | 2017 | 40 | 12.1 | N/M | N/M | 135 | 12.9 | 40 | 333.6 | N/M | N/M | 135 | 357.1 |
| Tantawy | 2012 | N/M | N/M | N/M | N/M | N/M | N/M | N/M | N/M | N/M | N/M | N/M | N/M |
| Zilberman | 2007 | N/M | N/M | N/M | N/M | N/M | N/M | N/M | N/M | N/M | N/M | N/M | N/M |

SCD: Sickle cell disease, ePASP: Estimated pulmonary artery systolic pressure, WBC: White Blood Cell, Plt: Platelet, N/M; Not mentioned

*Both WBC (×10^3^/ml) and Plt (×10^3^/ml) 1 and 2 relate to the cut-off values of 1 and 2, respectively (patients with elevated ePASP).

** Both WBC and Plt 3 relate to the cut-off values of 3 (patients with normal ePASP).

**eTable 6. Continued.** **Laboratory findings of children comparing elevated ePASP to those without.**

| Author | Year | No. LDH 1 | LDH 1 | No. LDH 2 | LDH 2 | No. LDH 3 | LDH 3 | No. Retic 1 | Retic 1 | No. Retic 2 | Retic 2 | No. Retic 3 | Retic 3 |
| --- | --- | --- | --- | --- | --- | --- | --- | --- | --- | --- | --- | --- | --- |
| AboHadeed | 2015 | N/M | N/M | N/M | N/M | N/M | N/M | N/M | N/M | N/M | N/M | N/M | N/M |
| Adly | 2016 | N/M | N/M | N/M | N/M | N/M | N/M | N/M | N/M | N/M | N/M | N/M | N/M |
| Agha | 2014 | 23 | 598.9 | N/M | N/M | 57 | 574.7 | 23 | 13.4 | N/M | N/M | 57 | 6 |
| Allen | 2019 | N/M | N/M | N/M | N/M | N/M | N/M | N/M | N/M | N/M | N/M | N/M | N/M |
| Al-Allawi | 2016 | 10 | 661.5 | N/M | N/M | 84 | 624.9 | 10 | 22.5 | N/M | N/M | 84 | 16.4 |
| Blanc | 2012 | N/M | N/M | N/M | N/M | N/M | N/M | N/M | N/M | N/M | N/M | N/M | N/M |
| Caldas | 2008 | N/M | N/M | N/M | N/M | N/M | N/M | N/M | N/M | N/M | N/M | N/M | N/M |
| Chaudry | 2011 | N/M | N/M | N/M | N/M | N/M | N/M | N/M | N/M | N/M | N/M | N/M | N/M |
| Chinawa | 2020 | N/M | N/M | N/M | N/M | N/M | N/M | N/M | N/M | N/M | N/M | N/M | N/M |
| Colombatti | 2010 | 7 | 1370.5 | N/M | N/M | 28 | 1361.7 | 8 | 36.9 | N/M | N/M | 29 | 25.7 |
| Cox | 2014 | 21 | 586 | 4 | 753 | 147 | 659.3 | N/M | N/M | N/M | N/M | N/M | N/M |
| Dahoui | 2010 | 27 | 581 | N/M | N/M | 58 | 425 | 27 | 10.9 | N/M | N/M | 58 | 9.3 |
| ElAlfy | 2019 | N/M | N/M | N/M | N/M | N/M | N/M | N/M | N/M | N/M | N/M | N/M | N/M |
| Eddine | 2012 | N/M | N/M | N/M | N/M | N/M | N/M | N/M | N/M | N/M | N/M | N/M | N/M |
| Elbarbary | 2016 | N/M | N/M | N/M | N/M | N/M | N/M | N/M | N/M | N/M | N/M | N/M | N/M |
| El‐Shanshory | 2013 | 9 | 935.7 | N/M | N/M | 21 | 406.3 | 9 | 12.9 | N/M | N/M | 21 | 7.7 |
| Forrest | 2012 | 28 | 650.5 | N/M | N/M | 57 | 648.2 | 28 | 13.7 | N/M | N/M | 57 | 10.4 |
| Garnier | 2017 | N/M | N/M | N/M | N/M | N/M | N/M | N/M | N/M | N/M | N/M | N/M | N/M |
| Gordeuk | 2009 | N/M | N/M | N/M | N/M | N/M | N/M | N/M | N/M | N/M | N/M | N/M | N/M |
| Hanna | 2021 | N/M | N/M | N/M | N/M | N/M | N/M | N/M | N/M | N/M | N/M | N/M | N/M |
| Hebson | 2015 | N/M | N/M | N/M | N/M | N/M | N/M | N/M | N/M | N/M | N/M | N/M | N/M |
| Johnson | 2010 | N/M | N/M | N/M | N/M | N/M | N/M | N/M | N/M | N/M | N/M | N/M | N/M |
| Lamina | 2019 | N/M | N/M | N/M | N/M | N/M | N/M | N/M | N/M | N/M | N/M | N/M | N/M |
| Lee | 2009 | 18 | 578 | N/M | N/M | 68 | 408 | 18 | 10.1 | N/M | N/M | 70 | 8.5 |
| Liem | 2009 | N/M | N/M | N/M | N/M | N/M | N/M | N/M | N/M | N/M | N/M | N/M | N/M |
| Lilje | 2017 | 18 | 1484 | 5 | 1380 | 68 | 1035 | 18 | 10.5 | 5 | 14.6 | 68 | 7.8 |
| Marouf | 2013 | N/M | N/M | N/M | N/M | N/M | N/M | 8 | 6.9 | N/M | N/M | 49 | 4.9 |
| Minniti | 2009 | 29 | 456.3 | N/M | N/M | 247 | 374 | 30 | 9.5 | N/M | N/M | 244 | 7.4 |
| Nelson | 2007 | N/M | N/M | N/M | N/M | N/M | N/M | N/M | N/M | N/M | N/M | N/M | N/M |
| Odeyemi | 2022 | N/M | N/M | N/M | N/M | N/M | N/M | N/M | N/M | N/M | N/M | N/M | N/M |
| Pashankar | 2008 | 13 | 735 | 6 | 442.5 | 43 | 649 | 13 | 11.7 | 6 | 17.8 | 43 | 9.6 |
| Peter | 2019 | 22 | 723.3 | N/M | N/M | 78 | 789.8 | 22 | 11.3 | N/M | N/M | 78 | 11.5 |
| Ranque | 2016 | N/M | N/M | N/M | N/M | N/M | N/M | N/M | N/M | N/M | N/M | N/M | N/M |
| Ribera | 2015 | N/M | N/M | N/M | N/M | N/M | N/M | N/M | N/M | N/M | N/M | N/M | N/M |
| Sedrak | 2009 | N/M | N/M | N/M | N/M | N/M | N/M | 4 | 13.1 | N/M | N/M | 44 | 8.6 |
| Sokunbi | 2017 | 40 | 641.9 | N/M | N/M | 135 | 748.9 | 40 | 2.5 | N/M | N/M | 135 | 2.8 |
| Tantawy | 2012 | N/M | N/M | N/M | N/M | N/M | N/M | N/M | N/M | N/M | N/M | N/M | N/M |
| Zilberman | 2007 | N/M | N/M | N/M | N/M | N/M | N/M | N/M | N/M | N/M | N/M | N/M | N/M |

SCD: Sickle cell disease, ePASP: Estimated pulmonary artery systolic pressure, LDH: Lactate Dehydrogenase, N/M; Not mentioned

*Both LDH (U/l) and retic (%) 1 and 2 relate to the cut-off values of 1 and 2, respectively (patients with elevated ePASP).

** Both LDH and retic 3 relate to the cut-off values of 3 (patients with normal ePASP).

**eTable 6. Continued.** **Clinical findings of children comparing elevated ePASP to those without.**

| Author | Year | No. O2sat 1 | O2sat 1 | No. O2sat 2 | O2sat 2 | No. O2sat 3 | O2sat 3 |
| --- | --- | --- | --- | --- | --- | --- | --- |
| AboHadeed | 2015 | N/M | N/M | N/M | N/M | N/M | N/M |
| Adly | 2016 | N/M | N/M | N/M | N/M | N/M | N/M |
| Agha | 2014 | 23 | 94.4 | N/M | N/M | 57 | 97.5 |
| Allen | 2019 | N/M | N/M | N/M | N/M | N/M | N/M |
| Al-Allawi | 2016 | N/M | N/M | N/M | N/M | N/M | N/M |
| Blanc | 2012 | N/M | N/M | N/M | N/M | N/M | N/M |
| Caldas | 2008 | N/M | N/M | N/M | N/M | N/M | N/M |
| Chaudry | 2011 | N/M | N/M | N/M | N/M | N/M | N/M |
| Chinawa | 2020 | N/M | N/M | N/M | N/M | N/M | N/M |
| Colombatti | 2010 | 8 | 97 | N/M | N/M | 29 | 98 |
| Cox | 2014 | 22 | 96.8 | 5 | 95.6 | 161 | 97.7 |
| Dahoui | 2010 | N/M | N/M | N/M | N/M | N/M | N/M |
| ElAlfy | 2019 | N/M | N/M | N/M | N/M | N/M | N/M |
| Eddine | 2012 | N/M | N/M | N/M | N/M | N/M | N/M |
| Elbarbary | 2016 | N/M | N/M | N/M | N/M | N/M | N/M |
| El‐Shanshory | 2013 | N/M | N/M | N/M | N/M | N/M | N/M |
| Forrest | 2012 | 28 | 95.6 | N/M | N/M | 57 | 97.3 |
| Garnier | 2017 | N/M | N/M | N/M | N/M | N/M | N/M |
| Gordeuk | 2009 | N/M | N/M | N/M | N/M | N/M | N/M |
| Hanna | 2021 | N/M | N/M | N/M | N/M | N/M | N/M |
| Hebson | 2015 | N/M | N/M | N/M | N/M | N/M | N/M |
| Johnson | 2010 | N/M | N/M | N/M | N/M | N/M | N/M |
| Lamina | 2019 | N/M | N/M | N/M | N/M | N/M | N/M |
| Lee | 2009 | 18 | 97 | N/M | N/M | 70 | 98 |
| Liem | 2009 | N/M | N/M | N/M | N/M | N/M | N/M |
| Lilje | 2017 | N/M | N/M | N/M | N/M | N/M | N/M |
| Marouf | 2013 | N/M | N/M | N/M | N/M | N/M | N/M |
| Minniti | 2009 | 30 | 97 | N/M | N/M | 250 | 98.3 |
| Nelson | 2007 | 15 | 95.4 | N/M | N/M | 38 | 96.7 |
| Odeyemi | 2022 | N/M | N/M | N/M | N/M | N/M | N/M |
| Pashankar | 2008 | 13 | 96.1 | 6 | 92.6 | 43 | 97.1 |
| Peter | 2019 | N/M | N/M | N/M | N/M | N/M | N/M |
| Ranque | 2016 | N/M | N/M | N/M | N/M | N/M | N/M |
| Ribera | 2015 | N/M | N/M | N/M | N/M | N/M | N/M |
| Sedrak | 2009 | 4 | 98 | N/M | N/M | 44 | 97 |
| Sokunbi | 2017 | N/M | N/M | N/M | N/M | N/M | N/M |
| Tantawy | 2012 | N/M | N/M | N/M | N/M | N/M | N/M |
| Zilberman | 2007 | N/M | N/M | N/M | N/M | N/M | N/M |

SCD: Sickle cell disease, ePASP: Estimated pulmonary artery systolic pressure, O2sat: O2 saturation, N/M; Not mentioned

*O2 saturation (%) 1 and 2 relate to the cut-off values of 1 and 2, respectively (patients with elevated ePASP).

**O2 saturation 3 relate to the cut-off value of 3 (patients with normal ePASP).

**References**

1. Abdul-Mohsen, M.F., *Echocardiographic evaluation of left ventricular diastolic and systolic function in Saudi patients with sickle cell disease.* Journal of the Saudi Heart Association, 2012. **24**(4): p. 217-224.

2. Aessopos, A., et al., *Cardiac involvement in sickle β-thalassemia.* Annals of hematology, 2009. **88**: p. 557-564.

3. Afriyie-Mensah, J.S., et al., *Clinical and laboratory predictors of elevated TRV in sickle cell anaemia.* Journal of Hematalogy and Oncology Research, 2018. **3**(1): p. 29-38.

4. Akgül, F., et al., *Pulmonary hypertension in sickle-cell disease: comorbidities and echocardiographic findings.* Acta haematologica, 2007. **118**(1): p. 53-60.

5. Aleem, A., et al., *Echocardiographic abnormalities in adolescent and adult Saudi patients with sickle cell disease.* Saudi medical journal, 2007. **28**(7): p. 1072.

6. Aliyu, Z.Y., et al., *Prevalence and risk factors for pulmonary artery systolic hypertension among sickle cell disease patients in Nigeria.* American journal of hematology, 2008. **83**(6): p. 485-490.

7. Amadi, V.N., et al., *Pulmonary hypertension in Nigerian adults with sickle cell anemia.* Vascular Health and Risk Management, 2017: p. 153-160.

8. Anjum, F., et al., *Characterization of altered patterns of endothelial progenitor cells in sickle cell disease related pulmonary arterial hypertension.* Pulmonary circulation, 2012. **2**(1): p. 54-60.

9. Billy-Brissac, R., et al., *Pulmonary hypertension in an adult sickle cell population in Guadeloupe.* International journal of cardiology, 2009. **135**(1): p. 122-123.

10. Zimbarra Cabrita, I., et al., *The association between tricuspid regurgitation velocity and 5‐year survival in a N orth W est L ondon population of patients with sickle cell disease in the U nited K ingdom.* British journal of haematology, 2013. **162**(3): p. 400-408.

11. Caughey, M.C., et al., *Hemodynamic characteristics and predictors of pulmonary hypertension in patients with sickle cell disease.* The American journal of cardiology, 2012. **109**(9): p. 1353-1357.

12. Chiadika, S., et al., *Echocardiographic parameters to identify sickle cell patients with cardio‐pathology.* Echocardiography, 2018. **35**(9): p. 1271-1276.

13. Damy, T., et al., *Haematological determinants of cardiac involvement in adults with sickle cell disease.* European Heart Journal, 2016. **37**(14): p. 1158-1167.

14. Dei-Adomakoh, Y.A., et al., *Lung function abnormalities in sickle cell anaemia.* Advances in Hematology, 2019. **2019**(1): p. 1783240.

15. Delclaux, C., et al., *Factors associated with dyspnea in adult patients with sickle cell disease.* Chest, 2005. **128**(5): p. 3336-3344.

16. d'Humières, T., et al., *Cardiovascular phenotypes predict clinical outcomes in sickle cell disease: An echocardiography‐based cluster analysis.* American Journal of Hematology, 2021. **96**(9): p. 1166-1175.

17. Dosunmu, A.O., et al., *Prevalence of pulmonary hypertension in sickle cell anaemia patients of a tertiary hospital in Nigeria.* Nigerian Medical Journal, 2014. **55**(2): p. 161-165.

18. Elmariah, H., et al., *Factors associated with survival in a contemporary adult sickle cell disease cohort.* American journal of hematology, 2014. **89**(5): p. 530-535.

19. Fonseca, G.H.H., et al., *Pulmonary hypertension diagnosed by right heart catheterisation in sickle cell disease.* European Respiratory Journal, 2012. **39**(1): p. 112-118.

20. Garrido, V.T., et al., *Elevated plasma levels and platelet‐associated expression of the pro‐thrombotic and pro‐inflammatory protein, T NFSF14 (LIGHT), in sickle cell disease.* British journal of haematology, 2012. **158**(6): p. 788-797.

21. Guedeney, P., et al., *Cardiac manifestations in sickle cell disease varies with patient genotype.* British journal of haematology, 2018. **181**(5): p. 664-671.

22. Garadah, T., et al., *The effects of hydroxyurea therapy on the six-minute walk distance in patients with adult sickle cell anemia: an echocardiographic study.* Journal of Blood Medicine, 2019: p. 443-452.

23. Kato, G.J., et al., *Lactate dehydrogenase as a biomarker of hemolysis-associated nitric oxide resistance, priapism, leg ulceration, pulmonary hypertension, and death in patients with sickle cell disease.* Blood, 2006. **107**(6): p. 2279-2285.

24. Klings, E.S., et al., *Pulmonary arterial hypertension and left‐sided heart disease in sickle cell disease: Clinical characteristics and association with soluble adhesion molecule expression.* American journal of hematology, 2008. **83**(7): p. 547-553.

25. Knight-Perry, J.E., et al., *Abnormalities in cardiac structure and function in adults with sickle cell disease are not associated with pulmonary hypertension.* Journal of the American Society of Echocardiography, 2011. **24**(11): p. 1285-1290.

26. Lobo, C.L.d.C., et al., *Risk factors of pulmonary hypertension in Brazilian patients with sickle cell anemia.* PLoS One, 2015. **10**(9): p. e0137539.

27. Lopes, A., et al., *Relationship between ventilation heterogeneity and exercise intolerance in adults with sickle cell anemia.* Brazilian Journal of Medical and Biological Research, 2017. **50**: p. e6512.

28. Maioli, M.C.P., et al., *Relationship between pulmonary and cardiac abnormalities in sickle cell disease: implications for the management of patients.* Revista Brasileira de Hematologia e Hemoterapia, 2016. **38**: p. 21-27.

29. Maikap, D., et al., *Prevalence of pulmonary hypertension and factors associated with it in patients with sickle cell disease attending to sickle cell centre in a tertiary care hospital in Western Odisha: A cross sectional study.* Journal of Integrative Medicine and Research, 2023. **1**(3): p. 96-101.

30. de Lima Marinho, C., et al., *Predictive models of six-minute walking distance in adults with sickle cell anemia: Implications for rehabilitation.* Journal of bodywork and movement therapies, 2016. **20**(4): p. 824-831.

31. Mushemi-Blake, S., et al., *Pulmonary haemodynamics in sickle cell disease are driven predominantly by a high-output state rather than elevated pulmonary vascular resistance: a prospective 3-dimensional echocardiography/Doppler study.* PLoS One, 2015. **10**(8): p. e0135472.

32. Odeyemi, A.O., et al., *Pulmonary hypertension in people with sickle cell disease in a Nigerian tertiary hospital.* Assam Journal of Internal Medicine, 2022. **12**(1): p. 3-9.

33. Oguanobi, N., et al., *Clinical and electrocardiographic evaluation of sickle-cell anaemia patients with pulmonary hypertension.* ISRN hematology, 2012. **2012**.

34. Olatunya, O.S., et al., *Red blood cells microparticles are associated with hemolysis markers and may contribute to clinical events among sickle cell disease patients.* Annals of hematology, 2019. **98**: p. 2507-2521.

35. Parent, F., et al., *A hemodynamic study of pulmonary hypertension in sickle cell disease.* New England Journal of Medicine, 2011. **365**(1): p. 44-53.

36. Ranque, B., et al., *Arterial stiffness impairment in sickle cell disease associated with chronic vascular complications: the multinational African CADRE study.* Circulation, 2016. **134**(13): p. 923-933.

37. Sachdev, V., et al., *Echocardiographic markers of elevated pulmonary pressure and left ventricular diastolic dysfunction are associated with exercise intolerance in adults and adolescents with homozygous sickle cell anemia in the United States and United Kingdom.* Circulation, 2011. **124**(13): p. 1452-1460.

38. Sharma, S., et al., *Pulmonary artery occlusion pressure may overdiagnose pulmonary artery hypertension in sickle cell disease.* Clinical cardiology, 2013. **36**(9): p. 524-530.

39. van Beers, E.J., et al., *Cardiopulmonary imaging, functional and laboratory studies in sickle cell disease associated pulmonary hypertension.* American journal of hematology, 2008. **83**(11): p. 850-854.

40. Victor, K., et al., *Echocardiographic measures of pulmonary hypertension and the prediction of end‐points in sickle cell disease.* Sonography, 2016. **3**(1): p. 12-18.

41. Voskaridou, E., et al., *Pulmonary hypertension in patients with sickle cell/β thalassemia: incidence and correlation with serum N-terminal pro-brain natriuretic peptide concentrations.* Haematologica, 2007. **92**(6): p. 738-743.

42. AboHadeed, H.M., et al., *Assessment of cardiac functions in children with sickle cell anemia: Doppler tissue imaging study.* Archives of Medical Research, 2015. **46**(6): p. 462-469.

43. Adly, A.A., et al., *Soluble Fas/FasL ratio as a marker of vasculopathy in children and adolescents with sickle cell disease.* Cytokine, 2016. **79**: p. 52-58.

44. Agha, H., et al., *The 6-min walk test: an independent correlate of elevated tricuspid regurgitant jet velocity in children and young adult sickle cell patients.* Annals of hematology, 2014. **93**: p. 1131-1138.

45. Allen, K.Y., et al., *Echocardiographic screening of cardiovascular status in pediatric sickle cell disease.* Pediatric cardiology, 2019. **40**: p. 1670-1678.

46. Al-Allawi, N., A.M. Mohammad, and S. Jamal, *Doppler-Defined Pulmonary Hypertension in Sickle Cell Anemia in Kurdistan, Iraq.* PLoS One, 2016. **11**(9): p. e0162036.

47. Blanc, J., et al., *Right ventricular systolic strain is altered in children with sickle cell disease.* Journal of the American Society of Echocardiography, 2012. **25**(5): p. 511-517.

48. Caldas, M.C., Z.A. Meira, and M.M. Barbosa, *Evaluation of 107 patients with sickle cell anemia through tissue Doppler and myocardial performance index.* Journal of the American Society of Echocardiography, 2008. **21**(10): p. 1163-1167.

49. Chaudry, R.A., et al., *Paediatric sickle cell disease: pulmonary hypertension but normal vascular resistance.* Archives of disease in childhood, 2011. **96**(2): p. 131-136.

50. Chinawa, J.M., et al., *Right ventricular function among South East Nigeria children with sickle cell anaemia.* BMC pediatrics, 2020. **20**: p. 1-9.

51. Colombatti, R., et al., *Pulmonary hypertension in sickle cell disease children under 10 years of age.* British journal of haematology, 2010. **150**(5): p. 601-609.

52. Cox, S.E., et al., *Tricuspid regurgitant jet velocity and hospitalization in Tanzanian children with sickle cell anemia.* Haematologica, 2014. **99**(1): p. e1.

53. Dahoui, H.A., et al., *Pulmonary hypertension in children and young adults with sickle cell disease: evidence for familial clustering.* Pediatric blood & cancer, 2010. **54**(3): p. 398-402.

54. ElAlfy, M.S., et al., *Angiotensinogen M235T gene polymorphism is a genetic determinant of cerebrovascular and cardiopulmonary morbidity in adolescents with sickle cell disease.* Journal of Stroke and Cerebrovascular Diseases, 2019. **28**(2): p. 441-449.

55. Eddine, A.C., et al., *Ventricular structure and function in children with sickle cell disease using conventional and tissue Doppler echocardiography.* The American journal of cardiology, 2012. **109**(9): p. 1358-1364.

56. Elbarbary, N.S., et al., *Serum apelin as a novel non-invasive marker for subclinical cardiopulmonary complications in children and adolescents with sickle cell disease.* Blood Cells, Molecules, and Diseases, 2016. **57**: p. 1-7.

57. El‐Shanshory, M., et al., *Asymmetric dimethylarginine levels in children with sickle cell disease and its correlation to tricuspid regurgitant jet velocity.* European Journal of Haematology, 2013. **91**(1): p. 55-61.

58. Forrest, S., et al., *Proteinuria is associated with elevated tricuspid regurgitant jet velocity in children with sickle cell disease.* Pediatric blood & cancer, 2012. **58**(6): p. 937-940.

59. Garnier, Y., et al., *Differences of microparticle patterns between sickle cell anemia and hemoglobin SC patients.* PLoS One, 2017. **12**(5): p. e0177397.

60. Gordeuk, V.R., et al., *Relationship of erythropoietin, fetal hemoglobin, and hydroxyurea treatment to tricuspid regurgitation velocity in children with sickle cell disease.* Blood, The Journal of the American Society of Hematology, 2009. **114**(21): p. 4639-4644.

61. Hanna, D., et al., *Assessment of cystatin C in pediatric sickle cell disease and β-thalassemia as a marker of subclinical cardiovascular dysfunction: a case-control study.* Pediatric Hematology and Oncology, 2021. **38**(7): p. 620-632.

62. Hebson, C., et al., *Elevated tricuspid regurgitant velocity as a marker for pulmonary hypertension in children with sickle cell disease: less prevalent and predictive than previously thought?* Journal of pediatric hematology/oncology, 2015. **37**(2): p. 134-139.

63. Johnson, M.C., et al., *Left ventricular hypertrophy and diastolic dysfunction in children with sickle cell disease are related to asleep and waking oxygen desaturation.* Blood, The Journal of the American Society of Hematology, 2010. **116**(1): p. 16-21.

64. Lamina, M.O., et al., *Doppler echocardiographic assessment of pulmonary artery pressure in children with sickle cell anaemia.* Cardiovascular Diagnosis and Therapy, 2019. **9**(3): p. 204.

65. Lee, M.T., et al., *Doppler‐defined pulmonary hypertension and the risk of death in children with sickle cell disease followed for a mean of three years.* British journal of haematology, 2009. **146**(4): p. 437-441.

66. Liem, R.I., L.T. Young, and A.A. Thompson, *Prolonged QTc interval in children and young adults with sickle cell disease at steady state.* Pediatric Blood & Cancer, 2009. **52**(7): p. 842-846.

67. Lilje, C., et al., *A modified noninvasive screening protocol for pulmonary hypertension in children with sickle cell disease—Who should be sent for invasive evaluation?* Pediatric Blood & Cancer, 2017. **64**(11): p. e26606.

68. Marouf, R., et al., *Transthoracic echocardiography and 6-minute walk test in Kuwaiti sickle cell disease patients.* Medical Principles and Practice, 2014. **23**(3): p. 212-217.

69. Minniti, C.P., et al., *Elevated tricuspid regurgitant jet velocity in children and adolescents with sickle cell disease: association with hemolysis and hemoglobin oxygen desaturation.* haematologica, 2009. **94**(3): p. 340.

70. Nelson, S.C., et al., *High prevalence of pulmonary hypertension in children with sickle cell disease.* Journal of Pediatric Hematology/Oncology, 2007. **29**(5): p. 334-337.

71. Pashankar, F.D., et al., *Prevalence and risk factors of elevated pulmonary artery pressures in children with sickle cell disease.* Pediatrics, 2008. **121**(4): p. 777-782.

72. Peter, I.D., et al., *Pulmonary hypertension and right ventricular function in Nigerian children with sickle cell anaemia.* Transactions of The Royal Society of Tropical Medicine and Hygiene, 2019. **113**(8): p. 489-496.

73. Ribera, M.C., et al., *Echocardiography in sickle cell anaemia patients under 20 years of age: a descriptive study in the Brazilian Western Amazon.* Cardiology in the Young, 2015. **25**(1): p. 63-69.

74. Sedrak, A., et al., *A prospective appraisal of pulmonary hypertension in children with sickle cell disease.* Journal of pediatric hematology/oncology, 2009. **31**(2): p. 97-100.

75. Sokunbi, O.J., et al., *Pulmonary hypertension among 5 to 18 year old children with sickle cell anaemia in Nigeria.* PLoS One, 2017. **12**(9): p. e0184287.

76. Tantawy, A.A.G., A.A.M. Adly, and E.A.R. Ismail, *Soluble CD163 in young sickle cell disease patients and their trait siblings: a biomarker for pulmonary hypertension and vaso-occlusive complications.* Blood coagulation & fibrinolysis, 2012. **23**(7): p. 640-648.

77. Zilberman, M.V., et al., *Evaluation of left ventricular diastolic function in pediatric sickle cell disease patients.* American journal of hematology, 2007. **82**(6): p. 433-438.
