# Supplementary material for: Global prevalence of elevated estimated pulmonary artery systolic pressure in clinically stable children and adults with sickle cell disease: A systematic review and meta-analysis
Source: PLoS One. 2025 Feb 13;20(2):e0318751. doi: 10.1371/journal.pone.0318751 (PMC11825009; doi:10.1371/journal.pone.0318751)
Supplement: S7 File — (DOC) [file pone.0318751.s007.doc]

eFigures

Supplementary file


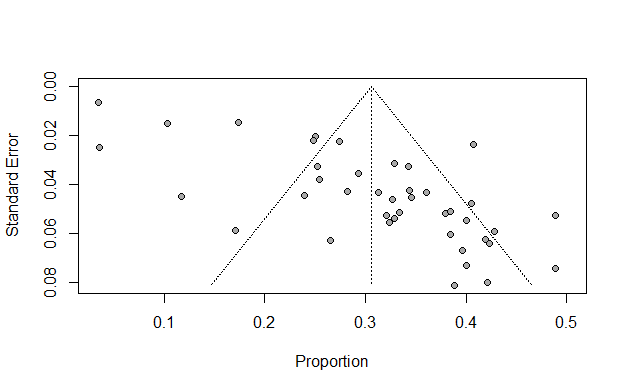


**eFigure 1.** Funnel plot of publication bias of primary studies to estimate the overall prevalence of elevated ePASP in adults.


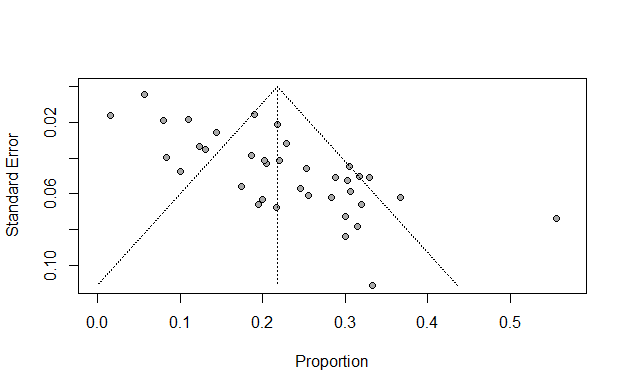


**eFigure 2.** Funnel plot of publication bias of primary studies to estimate the prevalence of elevated ePASP in children.


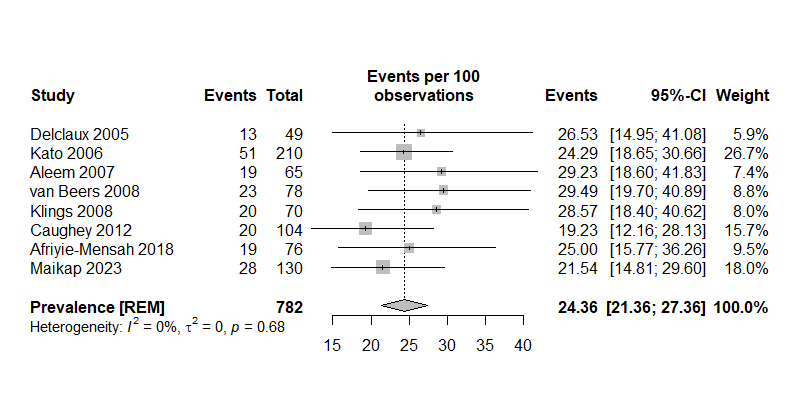


**eFigure 3.** The forest plot of the prevalence of mild elevated ePASP in adults by the primary studies and the overall estimate (95% CI).


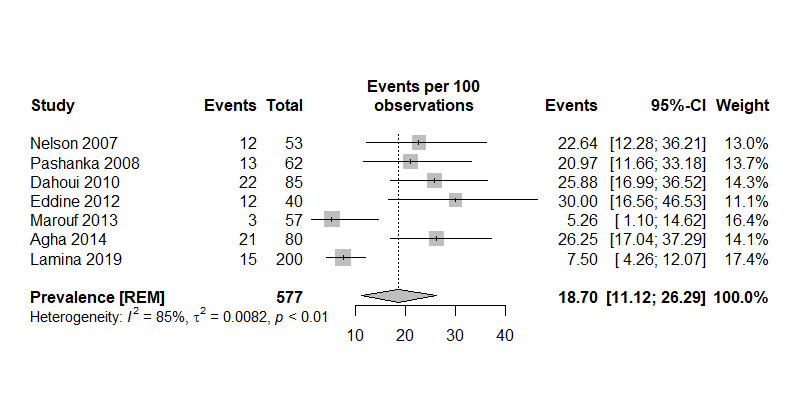


**eFigure 4.** The forest plot of the prevalence of mild elevated ePASP in children by the primary studies and the overall estimate (95% CI).


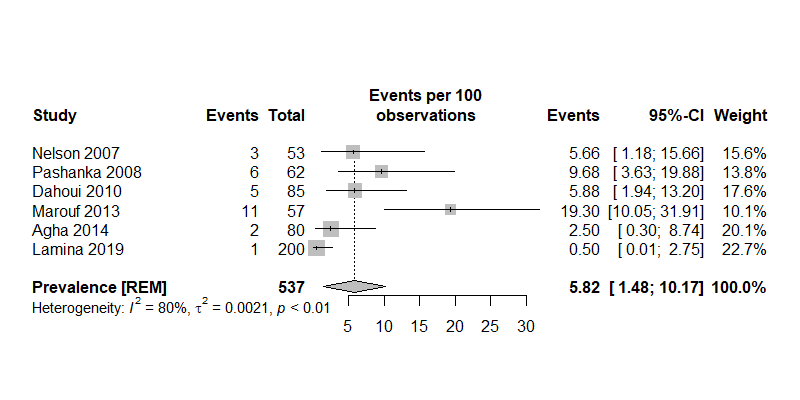


**eFigure 5.** The forest plot of the prevalence of moderate to severe elevated ePASP in children by the primary studies and the overall estimate (95% CI).


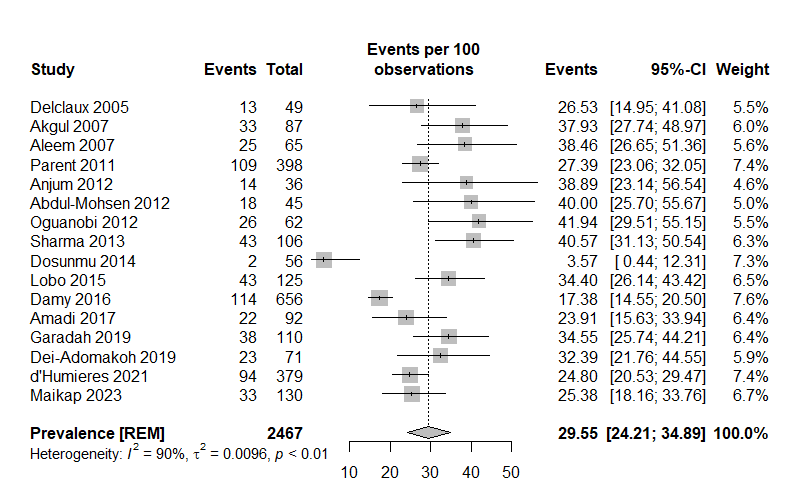


**eFigure 6.** The forest plot of the prevalence of elevated ePASP among adult studies with severe genotype of SCDs by the primary studies and the overall estimate (95% CI).


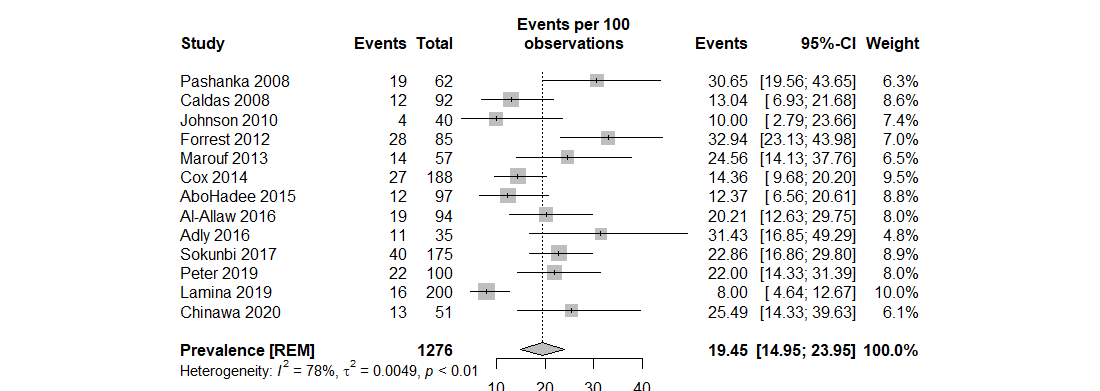


**eFigure 7.** The forest plot of the prevalence of elevated ePASP among children studies with severe genotype of SCDs by the primary studies and the overall estimate (95% CI).


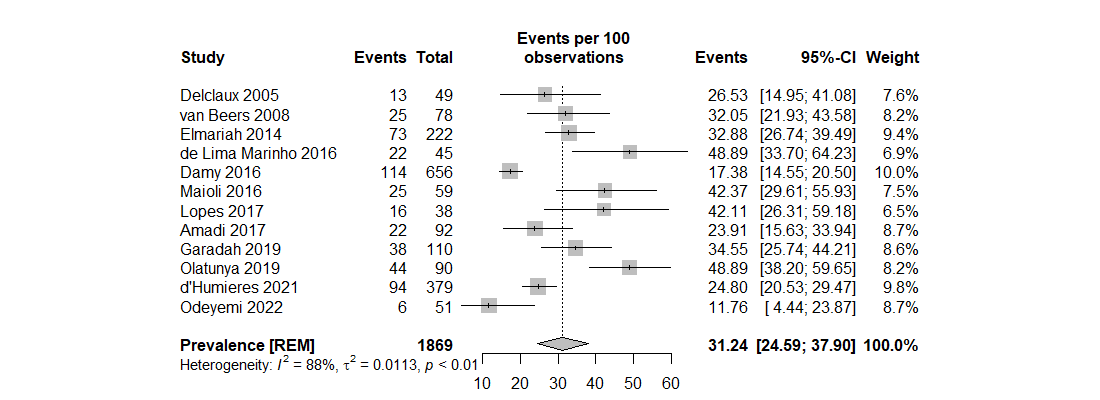


**eFigure 8.** The forest plot of the prevalence of elevated ePASP among adult studies with lack of blood transfusion in the preceding 3 months by the primary studies and the overall estimate (95% CI).


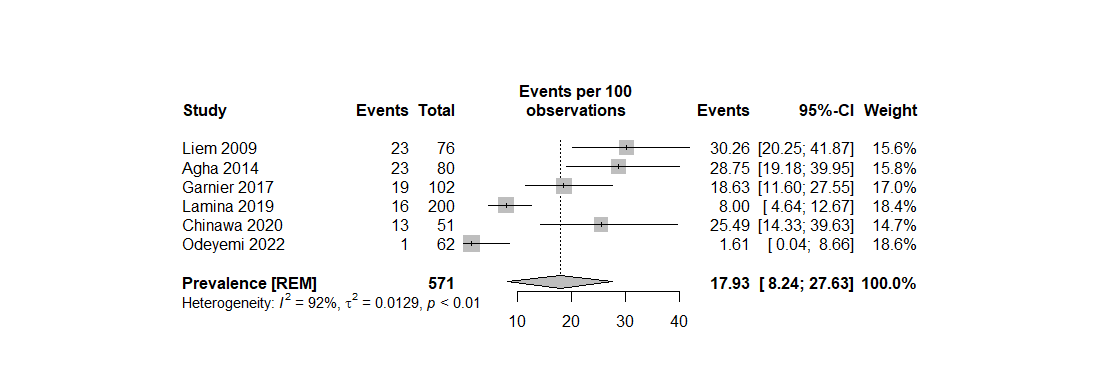


**eFigure 9.** The forest plot of the prevalence of elevated ePASP among children studies with lack of blood transfusion in the preceding 3 months by the primary studies and the overall estimate (95% CI).


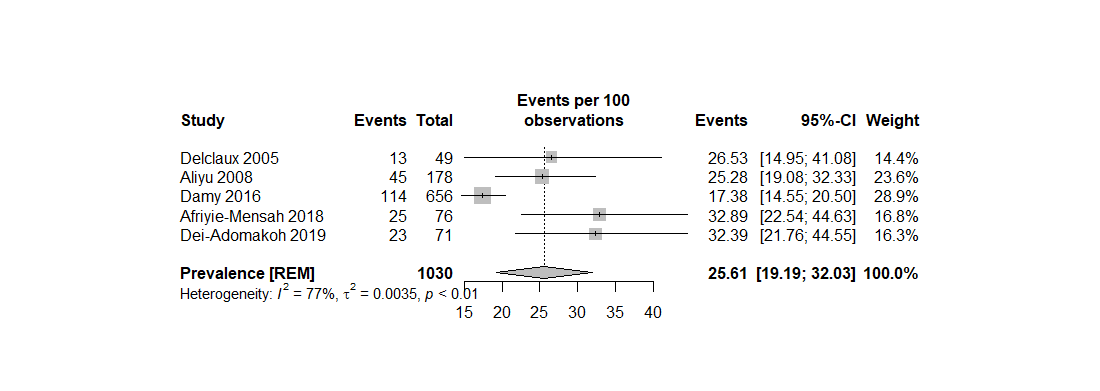


**eFigure 10.** The forest plot of the prevalence of elevated ePASP among adult studies with hydroxyurea-naive SCD patients by the primary studies and the overall estimate (95% CI).


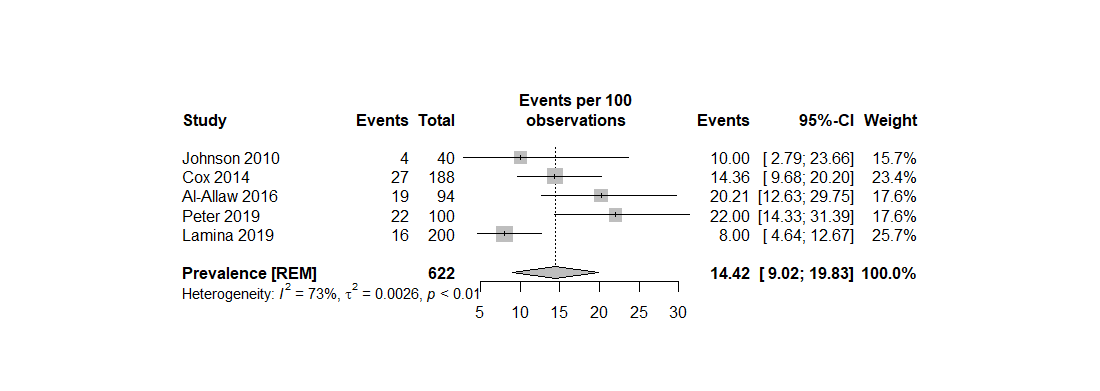


**eFigure 11.** The forest plot of the prevalence of elevated ePASP among children studies with hydroxyurea-naive SCD patients by the primary studies and the overall estimate (95% CI).


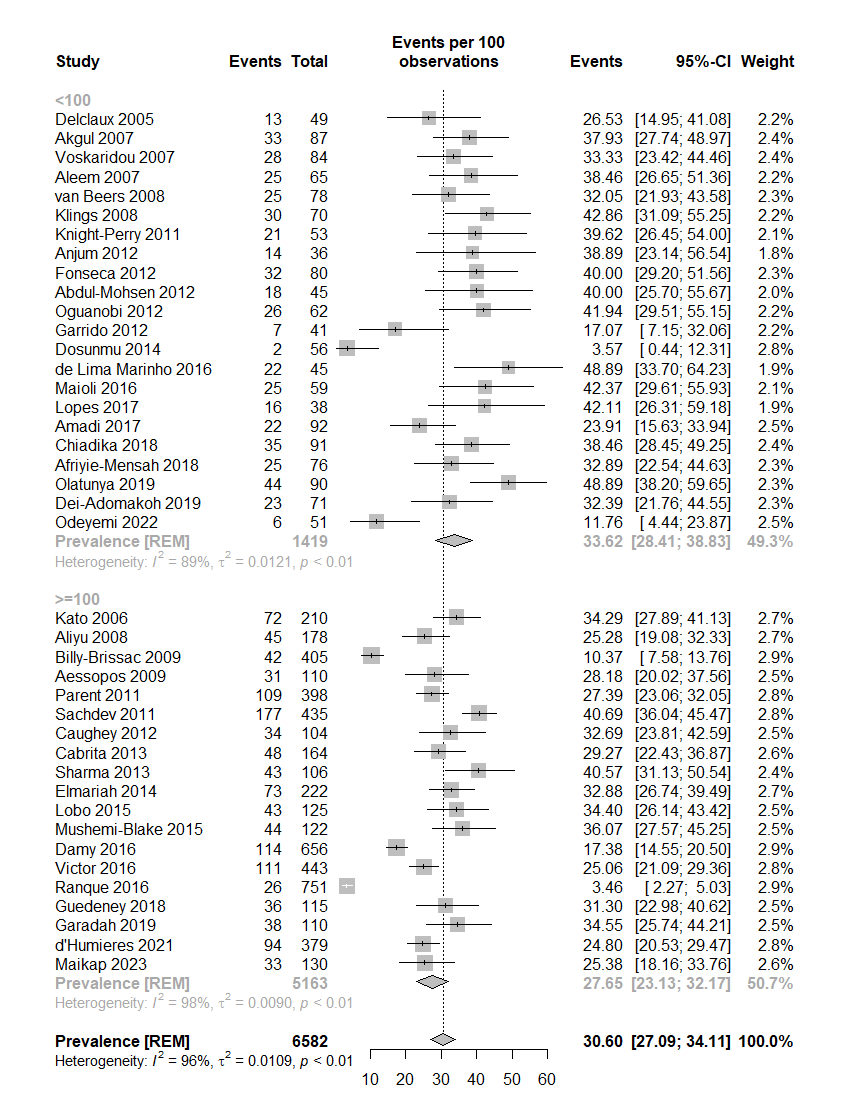


**eFigure 12.** The forest plot of the prevalence of elevated ePASP among adult studies based on the sample size by the primary studies and the overall estimate (95% CI).


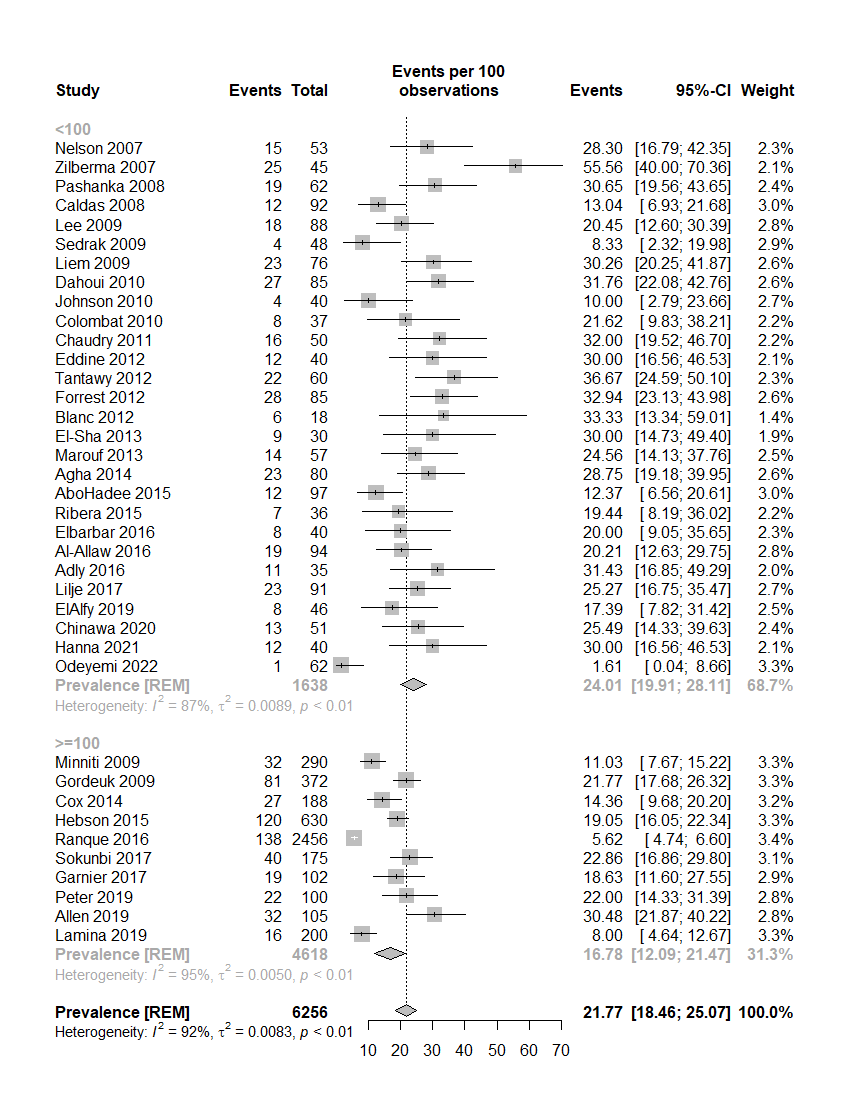


**eFigure 13.** The forest plot of the prevalence of elevated ePASP among children studies based on the sample size by the primary studies and the overall estimate (95% CI).


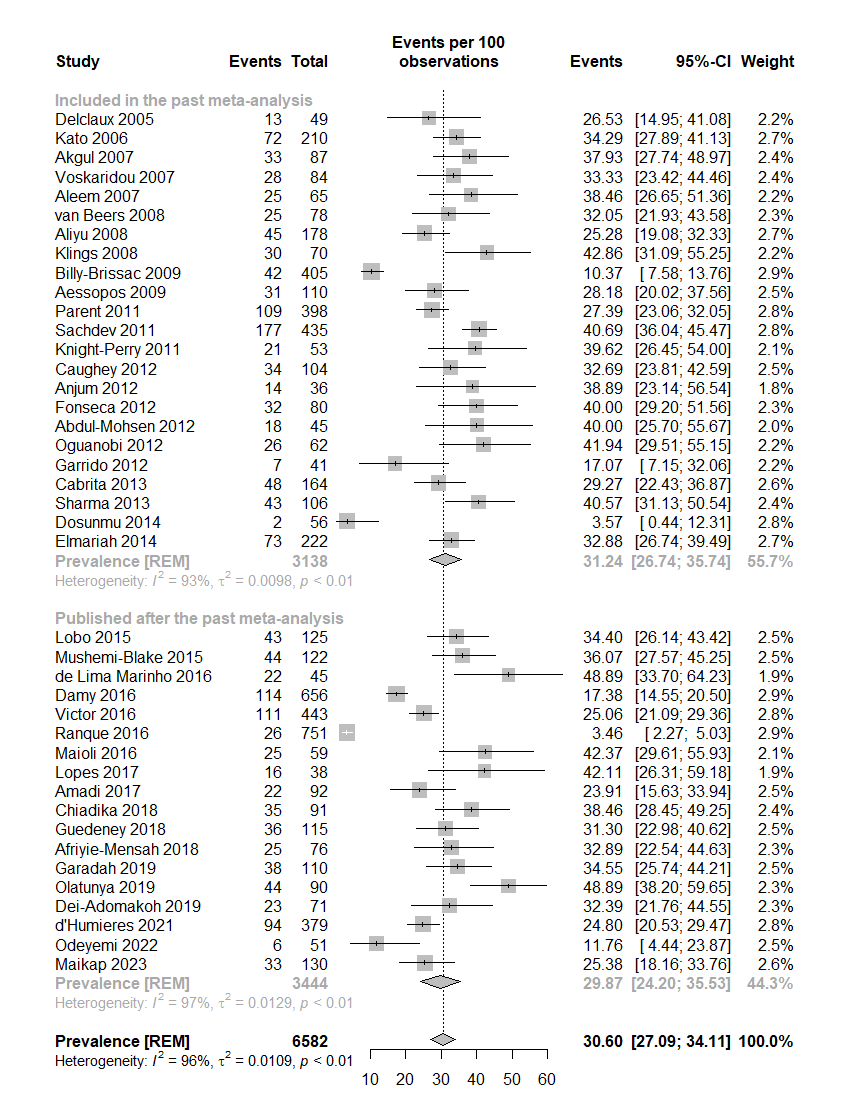


**eFigure 14.** The forest plot of the prevalence of elevated ePASP among adult studies based on the publication date by the primary studies and the overall estimate (95% CI).


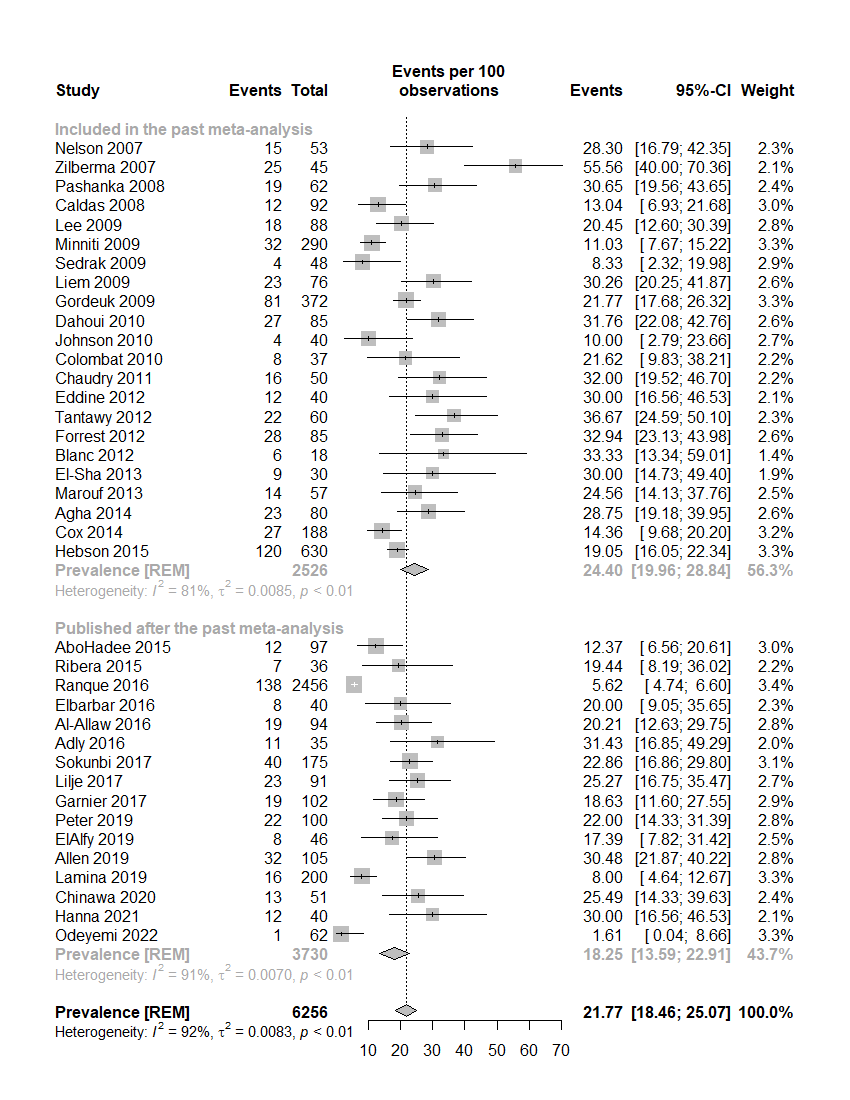


**eFigure 15.** The forest plot of the prevalence of elevated ePASP among children studies based on the publication date by the primary studies and the overall estimate (95% CI).


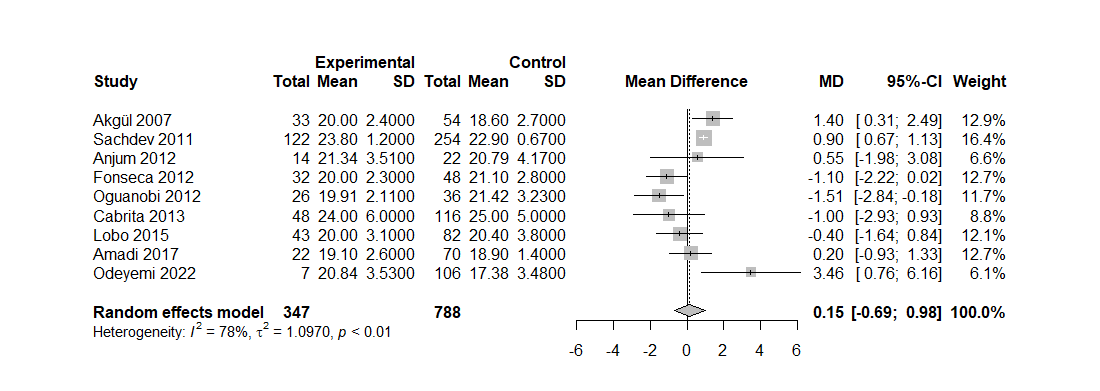


**eFigure 16.** Random-effects meta-analysis of Body mass index (mean difference) comparing adults with elevated ePASP to those without. Horizontal lines = width of 95% confidence interval, diamond = mean of random effects distribution, diamond width = width of 95% confidence interval, horizontal lines surrounding diamond = 95% predictive interval.


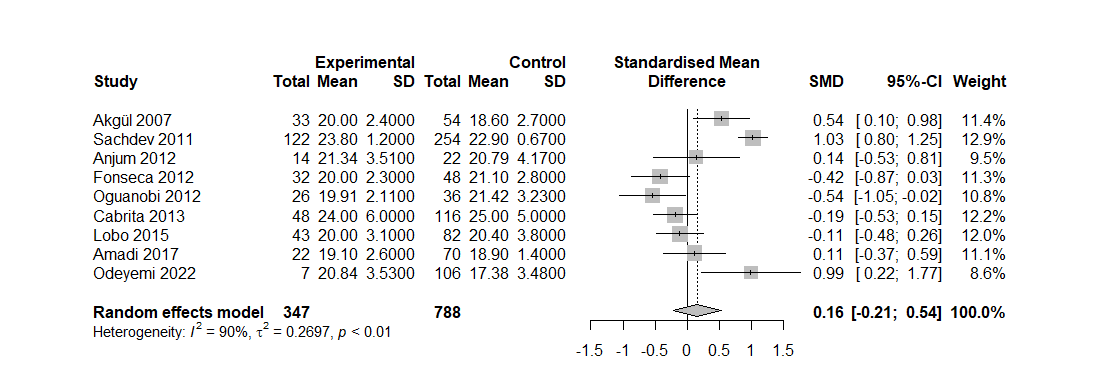


**eFigure 17.** Random-effects meta-analysis of Body mass index (standardized mean difference) comparing adults with elevated ePASP to those without. Horizontal lines = width of 95% confidence interval, diamond = standardized mean of random effects distribution, diamond width = width of 95% confidence interval, horizontal lines surrounding diamond = 95% predictive interval.


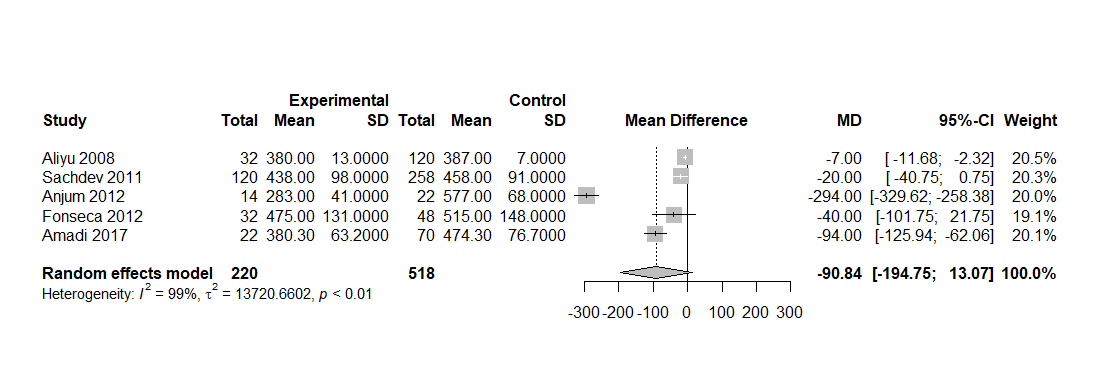


**eFigure 18.** Random-effects meta-analysis of six-minute walk distance (mean difference) comparing adults with elevated ePASP to those without. Horizontal lines = width of 95% confidence interval, diamond = mean of random effects distribution, diamond width = width of 95% confidence interval, horizontal lines surrounding diamond = 95% predictive interval.


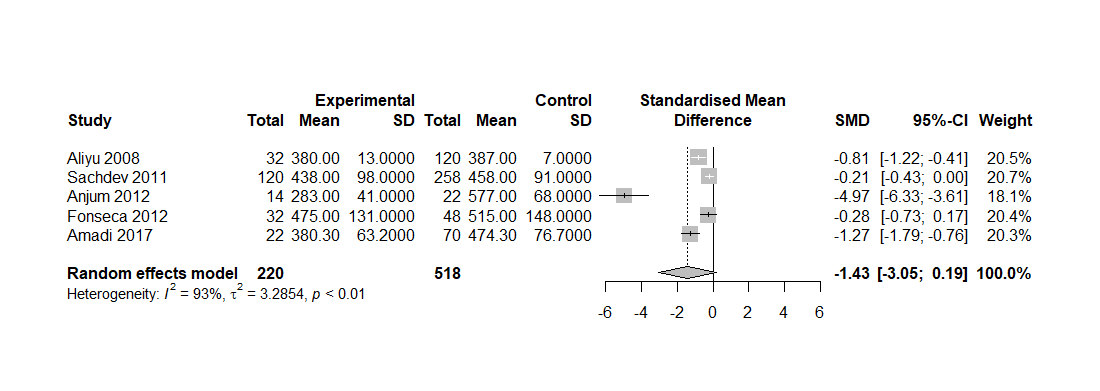


**eFigure 19.** Random-effects meta-analysis of six-minute walk distance (standardized mean difference) comparing adults with elevated ePASP to those without. Horizontal lines = width of 95% confidence interval, diamond = standardized mean of random effects distribution, diamond width = width of 95% confidence interval, horizontal lines surrounding diamond = 95% predictive interval.


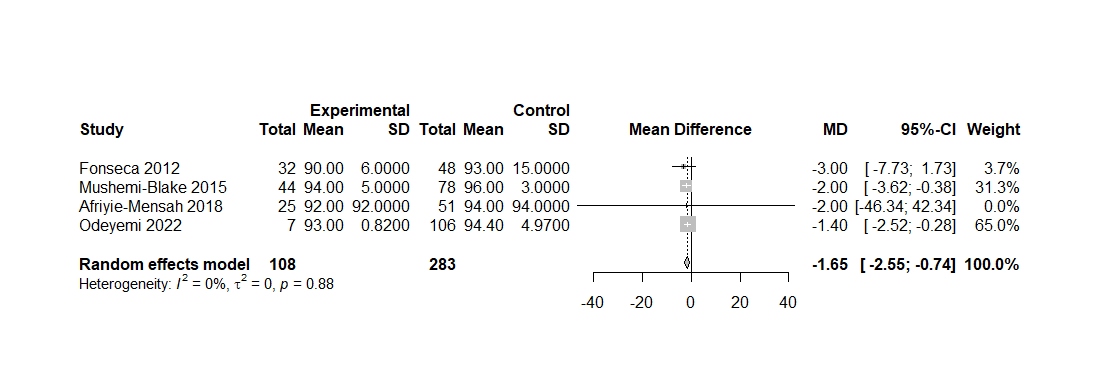


**eFigure 20.** Random-effects meta-analysis of O2 saturation (mean difference) comparing adults with elevated ePASP to those without. Horizontal lines = width of 95% confidence interval, diamond = mean of random effects distribution, diamond width = width of 95% confidence interval, horizontal lines surrounding diamond = 95% predictive interval.


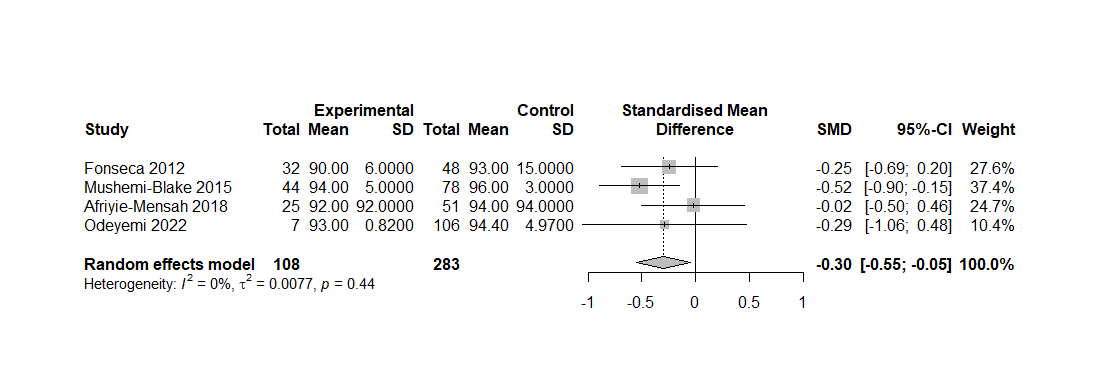


**eFigure 21.** Random-effects meta-analysis of O2 saturation (standardized mean difference) comparing adults with elevated ePASP to those without. Horizontal lines = width of 95% confidence interval, diamond = standardized mean of random effects distribution, diamond width = width of 95% confidence interval, horizontal lines surrounding diamond = 95% predictive interval.


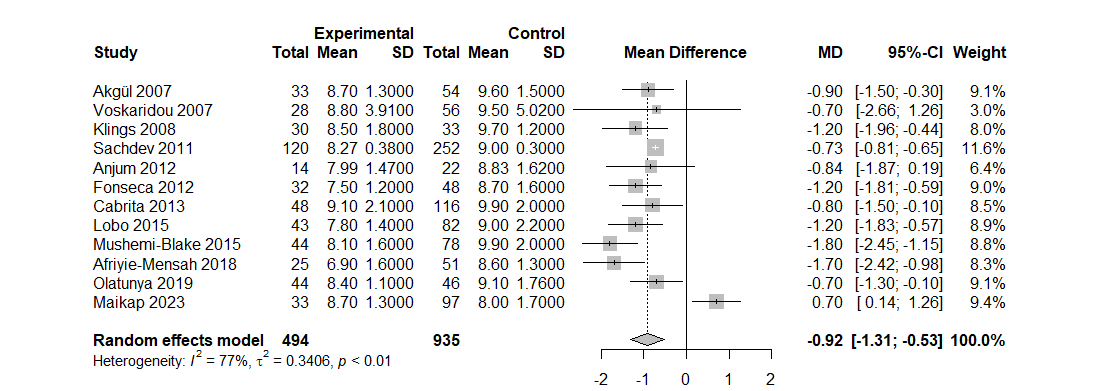


**eFigure 22.** Random-effects meta-analysis of hemoglobin level (mean difference) comparing adults with elevated ePASP to those without. Horizontal lines = width of 95% confidence interval, diamond = mean of random effects distribution, diamond width = width of 95% confidence interval, horizontal lines surrounding diamond = 95% predictive interval.


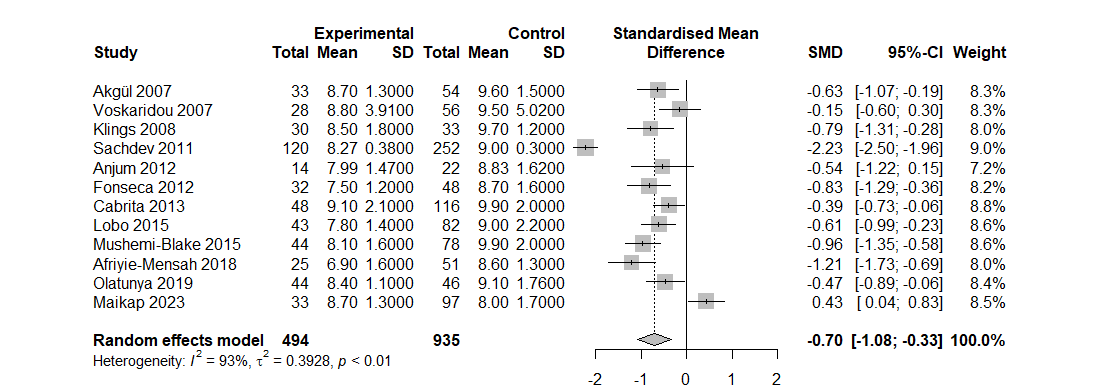


**eFigure 23.** Random-effects meta-analysis of hemoglobin level (standardized mean difference) comparing adults with elevated ePASP to those without. Horizontal lines = width of 95% confidence interval, diamond = standardized mean of random effects distribution, diamond width = width of 95% confidence interval, horizontal lines surrounding diamond = 95% predictive interval.


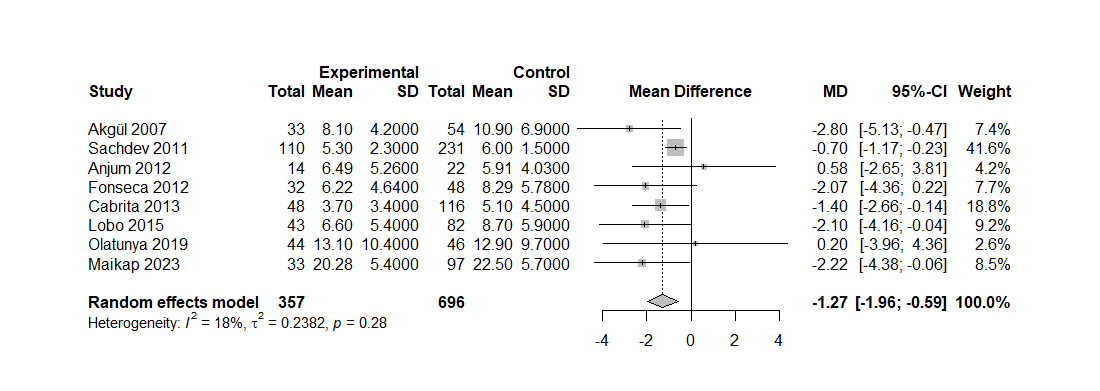


**eFigure 24.** Random-effects meta-analysis of fetal hemoglobin level (mean difference) comparing adults with elevated ePASP to those without. Horizontal lines = width of 95% confidence interval, diamond = mean of random effects distribution, diamond width = width of 95% confidence interval, horizontal lines surrounding diamond = 95% predictive interval.


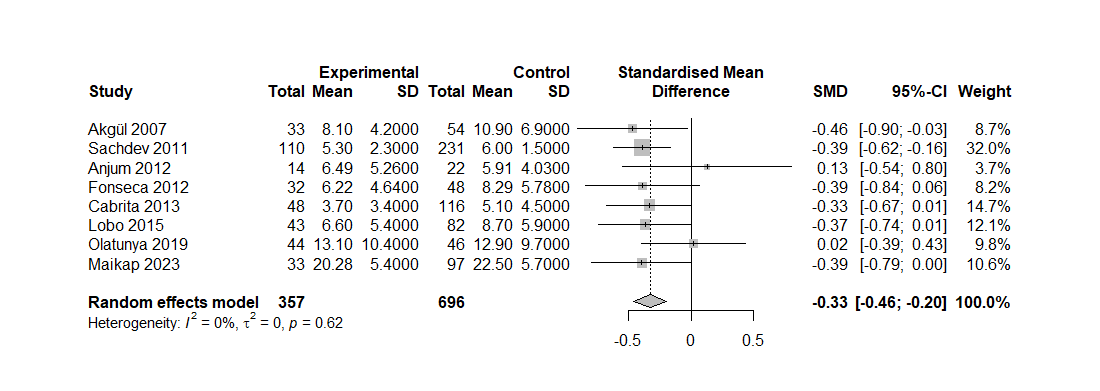


**eFigure 25.** Random-effects meta-analysis of fetal hemoglobin level (standardized mean difference) comparing adults with elevated ePASP to those without. Horizontal lines = width of 95% confidence interval, diamond = standardized mean of random effects distribution, diamond width = width of 95% confidence interval, horizontal lines surrounding diamond = 95% predictive interval.


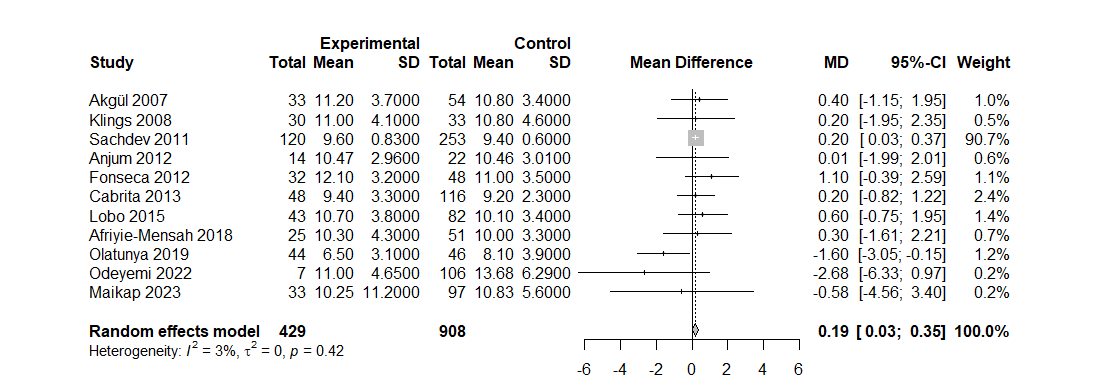


**eFigure 26.** Random-effects meta-analysis of white blood cell level (mean difference) comparing adults with elevated ePASP to those without. Horizontal lines = width of 95% confidence interval, diamond = mean of random effects distribution, diamond width = width of 95% confidence interval, horizontal lines surrounding diamond = 95% predictive interval.


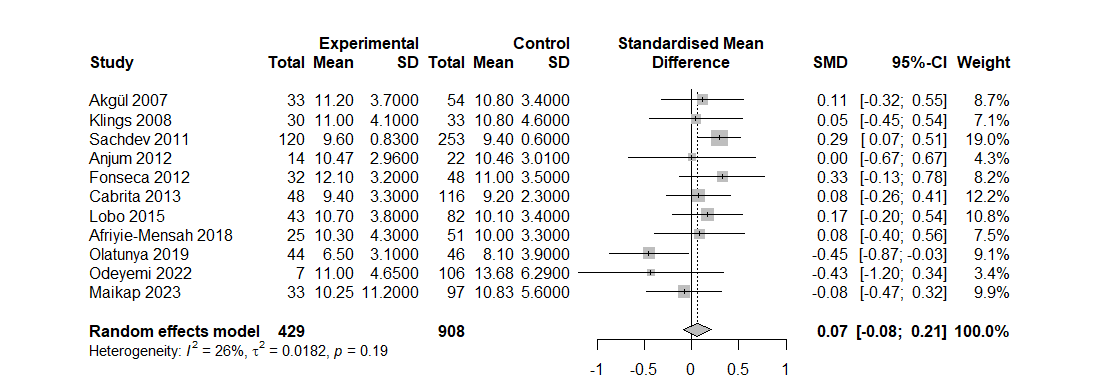


**eFigure 27.** Random-effects meta-analysis of white blood cell level (standardized mean difference) comparing adults with elevated ePASP to those without. Horizontal lines = width of 95% confidence interval, diamond = standardized mean of random effects distribution, diamond width = width of 95% confidence interval, horizontal lines surrounding diamond = 95% predictive interval.


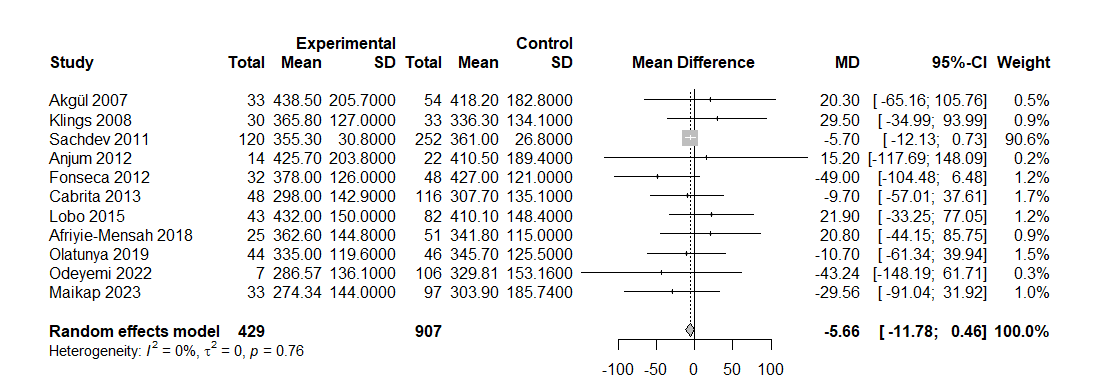


**eFigure 28.** Random-effects meta-analysis of platelets level (mean difference) comparing adults with elevated ePASP to those without. Horizontal lines = width of 95% confidence interval, diamond = mean of random effects distribution, diamond width = width of 95% confidence interval, horizontal lines surrounding diamond = 95% predictive interval.


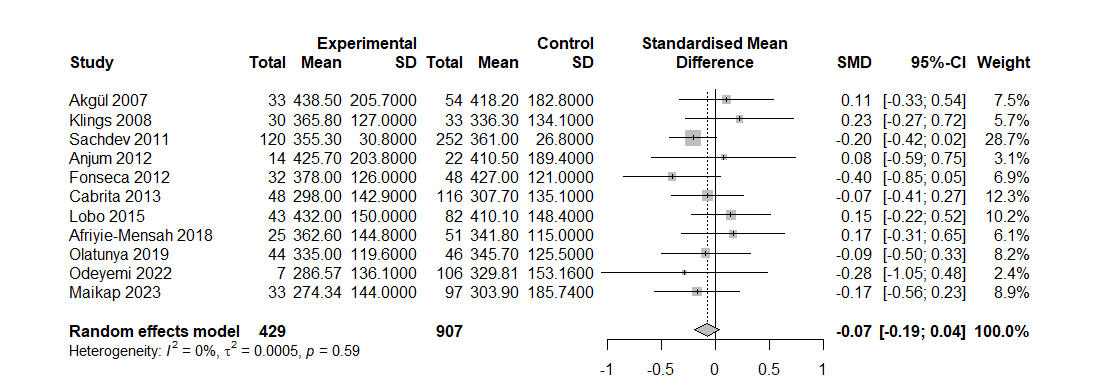


**eFigure 29.** Random-effects meta-analysis of platelets level (standardized mean difference) comparing adults with elevated ePASP to those without. Horizontal lines = width of 95% confidence interval, diamond = standardized mean of random effects distribution, diamond width = width of 95% confidence interval, horizontal lines surrounding diamond = 95% predictive interval.


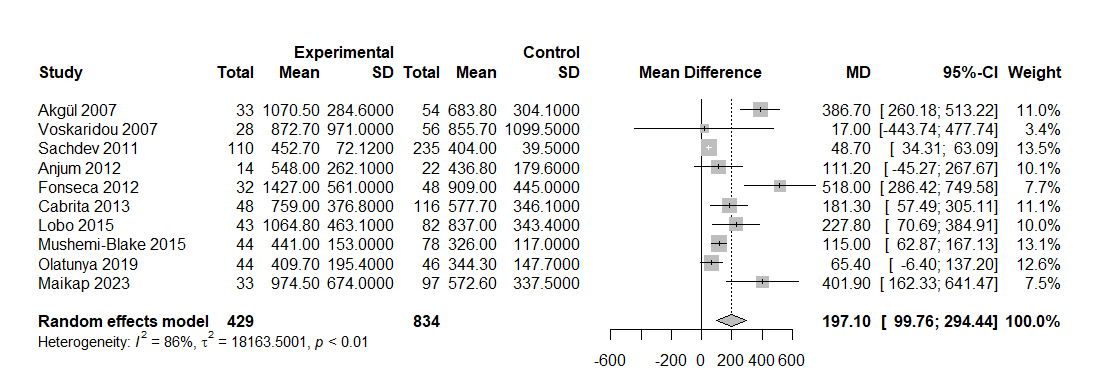


**eFigure 30.** Random-effects meta-analysis of lactate dehydrogenase level (mean difference) comparing adults with elevated ePASP to those without. Horizontal lines = width of 95% confidence interval, diamond = mean of random effects distribution, diamond width = width of 95% confidence interval, horizontal lines surrounding diamond = 95% predictive interval.


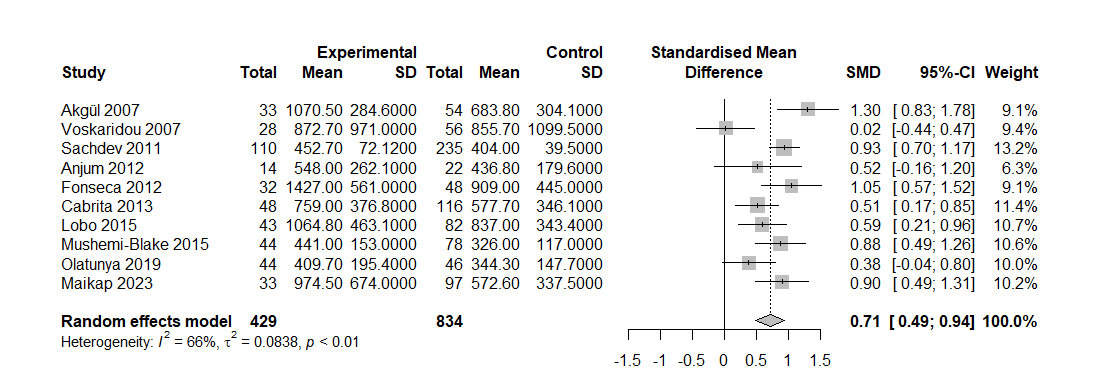


**eFigure 31.** Random-effects meta-analysis of lactate dehydrogenase level (standardized mean difference) comparing adults with elevated ePASP to those without. Horizontal lines = width of 95% confidence interval, diamond = standardized mean of random effects distribution, diamond width = width of 95% confidence interval, horizontal lines surrounding diamond = 95% predictive interval.


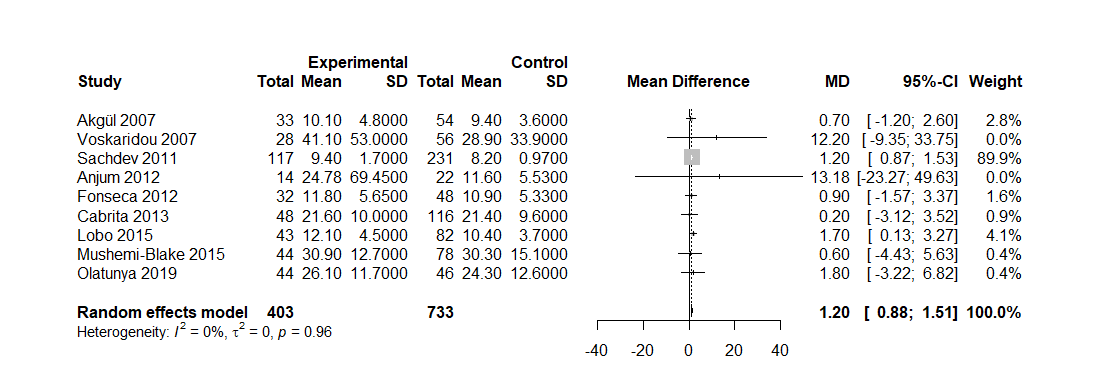


**eFigure 32.** Random-effects meta-analysis of reticulocyte count (mean difference) comparing adults with elevated ePASP to those without. Horizontal lines = width of 95% confidence interval, diamond = mean of random effects distribution, diamond width = width of 95% confidence interval, horizontal lines surrounding diamond = 95% predictive interval.


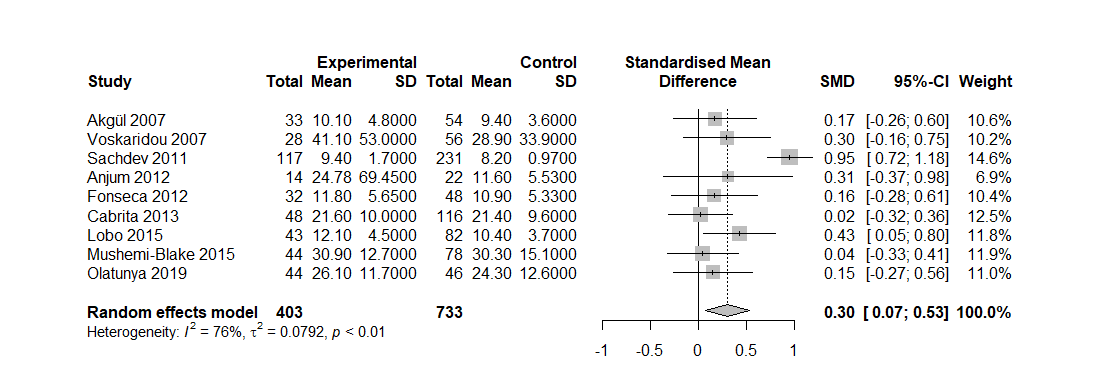


**eFigure 33.** Random-effects meta-analysis of reticulocyte count (standardized mean difference) comparing adults with elevated ePASP to those without. Horizontal lines = width of 95% confidence interval, diamond = standardized mean of random effects distribution, diamond width = width of 95% confidence interval, horizontal lines surrounding diamond = 95% predictive interval.


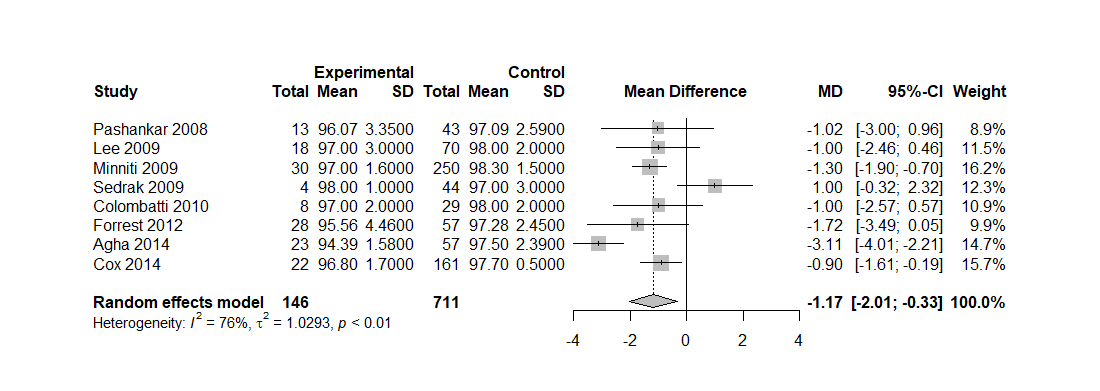


**eFigure 34.** Random-effects meta-analysis of Oxygen saturation (mean difference) comparing children with elevated ePASP to those without. Horizontal lines = width of 95% confidence interval, diamond = mean of random effects distribution, diamond width = width of 95% confidence interval, horizontal lines surrounding diamond = 95% predictive interval.


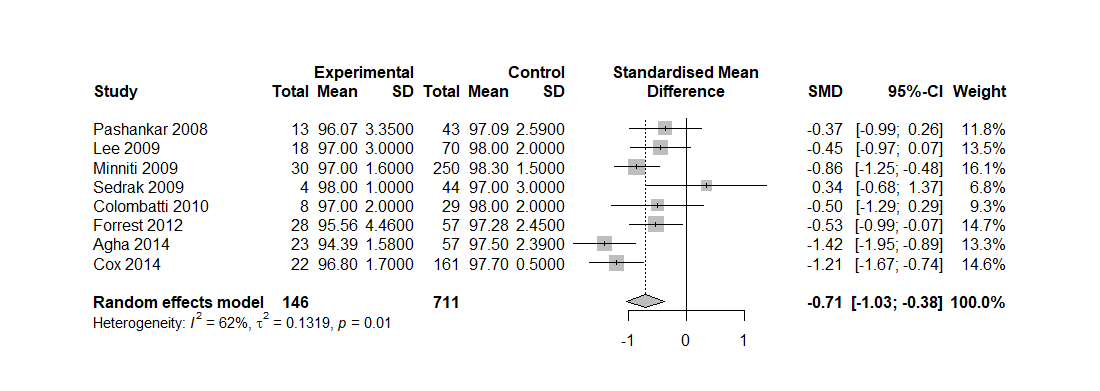


**eFigure 35.** Random-effects meta-analysis of Oxygen saturation (standardized mean difference) comparing children with elevated ePASP to those without. Horizontal lines = width of 95% confidence interval, diamond = standardized mean of random effects distribution, diamond width = width of 95% confidence interval, horizontal lines surrounding diamond = 95% predictive interval.


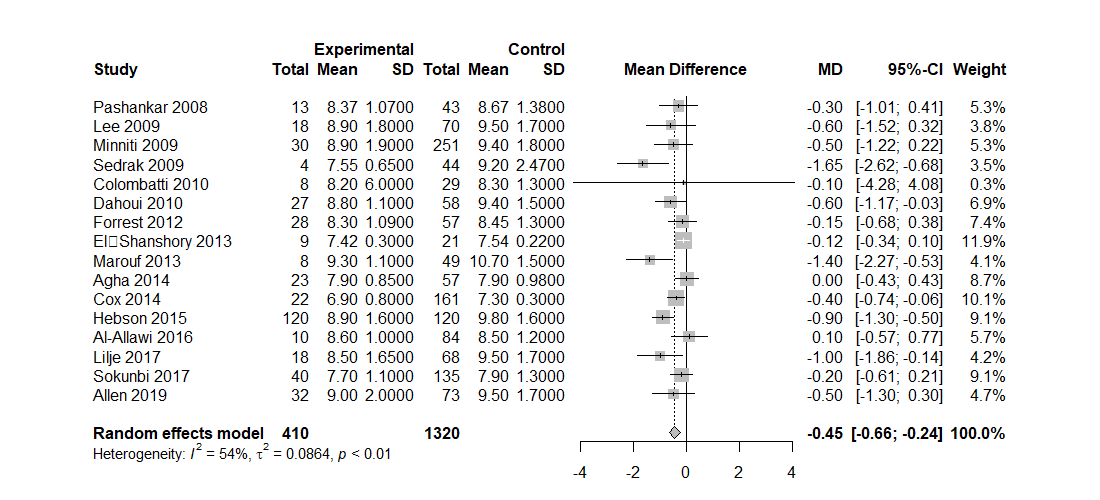


**eFigure 36.** Random-effects meta-analysis of hemoglobin level (mean difference) comparing children with elevated ePASP to those without. Horizontal lines = width of 95% confidence interval, diamond = mean of random effects distribution, diamond width = width of 95% confidence interval, horizontal lines surrounding diamond = 95% predictive interval.


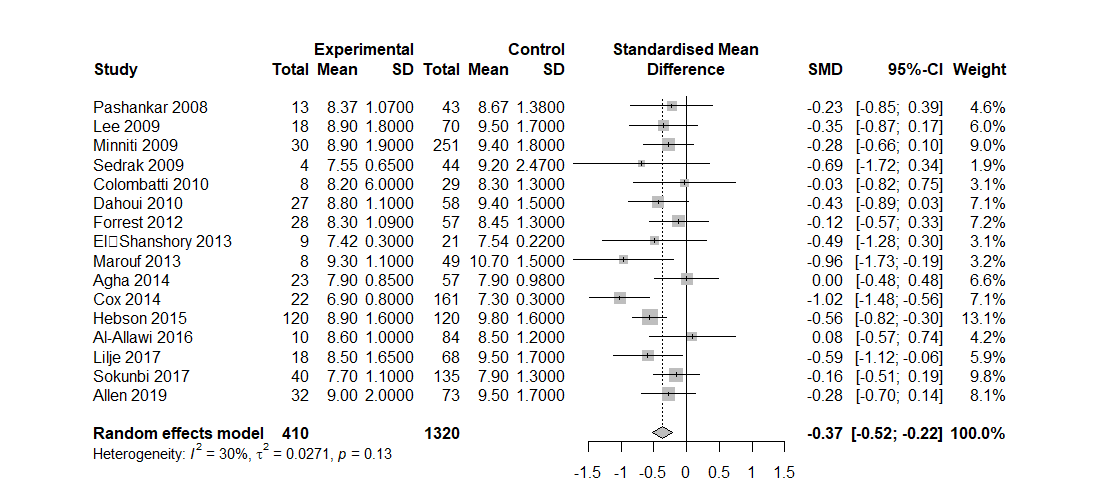


**eFigure 37.** Random-effects meta-analysis of hemoglobin level (standardized mean difference) comparing children with elevated ePASP to those without. Horizontal lines = width of 95% confidence interval, diamond = standardized mean of random effects distribution, diamond width = width of 95% confidence interval, horizontal lines surrounding diamond = 95% predictive interval.


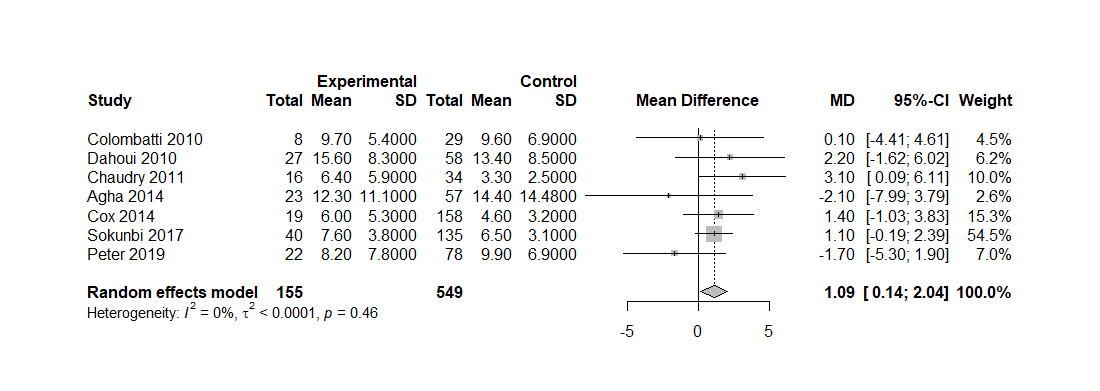


**eFigure 38.** Random-effects meta-analysis of fetal hemoglobin level (mean difference) comparing children with elevated ePASP to those without. Horizontal lines = width of 95% confidence interval, diamond = mean of random effects distribution, diamond width = width of 95% confidence interval, horizontal lines surrounding diamond = 95% predictive interval.


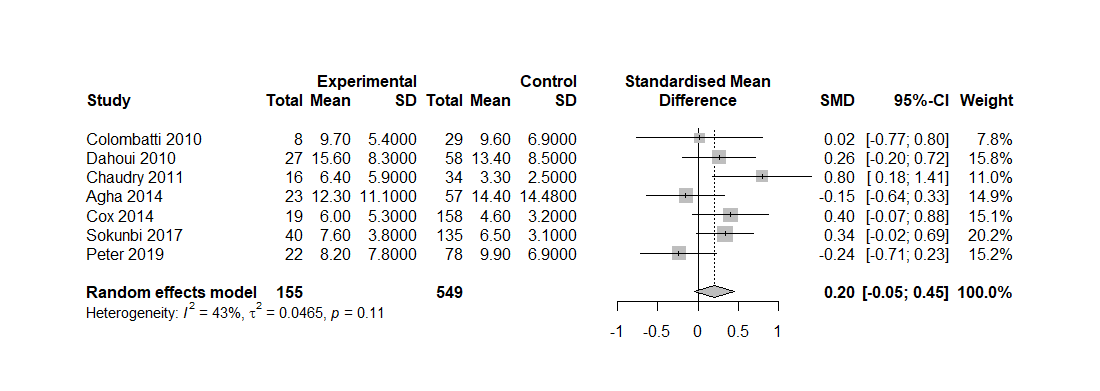


**eFigure 39.** Random-effects meta-analysis of fetal hemoglobin level (standardized mean difference) comparing children with elevated ePASP to those without. Horizontal lines = width of 95% confidence interval, diamond = standardized mean of random effects distribution, diamond width = width of 95% confidence interval, horizontal lines surrounding diamond = 95% predictive interval.


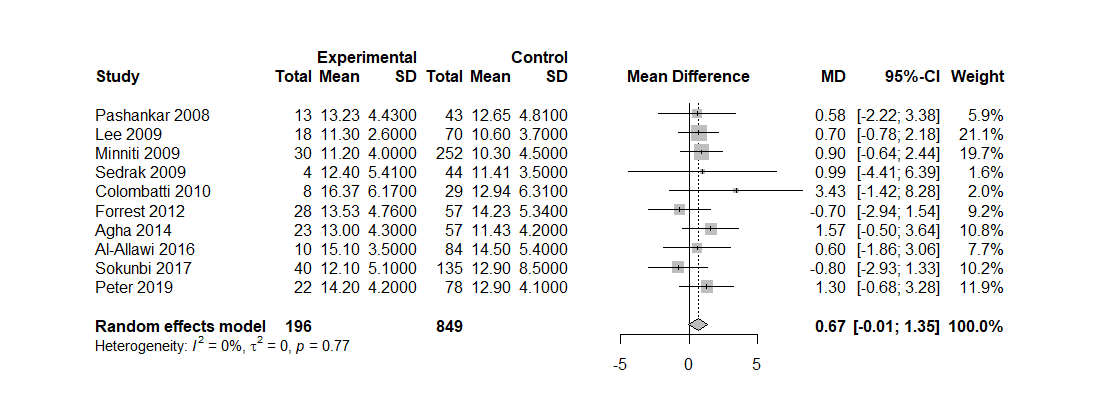


**eFigure 40.** Random-effects meta-analysis of white blood cell level (mean difference) comparing children with elevated ePASP to those without. Horizontal lines = width of 95% confidence interval, diamond = mean of random effects distribution, diamond width = width of 95% confidence interval, horizontal lines surrounding diamond = 95% predictive interval.


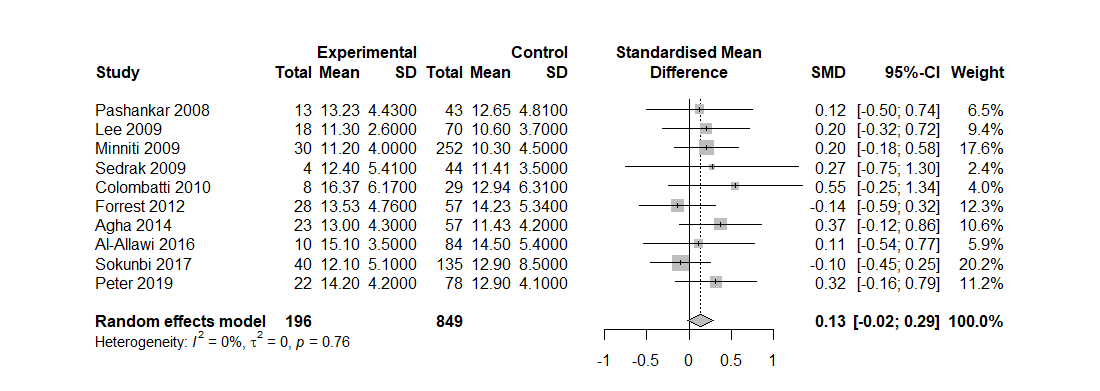


**eFigure 41.** Random-effects meta-analysis of white blood cell level (standardized mean difference) comparing children with elevated ePASP to those without. Horizontal lines = width of 95% confidence interval, diamond = standardized mean of random effects distribution, diamond width = width of 95% confidence interval, horizontal lines surrounding diamond = 95% predictive interval.


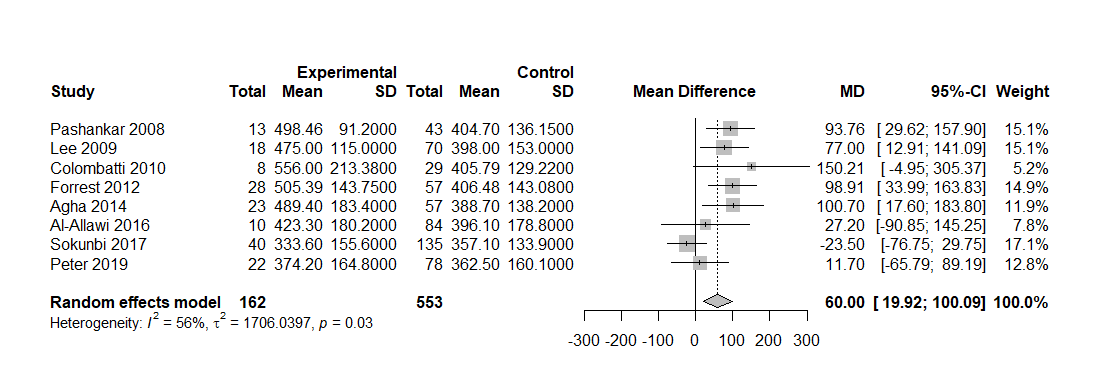


**eFigure 42.** Random-effects meta-analysis of platelet level (mean difference) comparing children with elevated ePASP to those without. Horizontal lines = width of 95% confidence interval, diamond = mean of random effects distribution, diamond width = width of 95% confidence interval, horizontal lines surrounding diamond = 95% predictive interval.


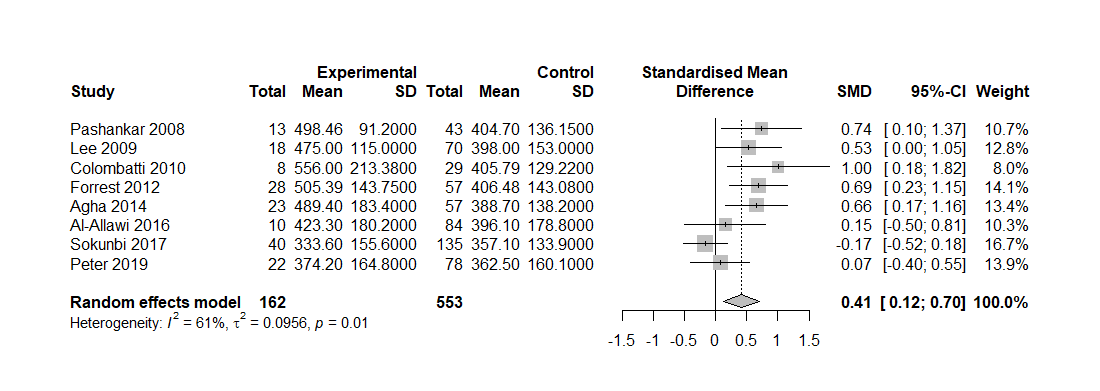


**eFigure 43.** Random-effects meta-analysis of platelet level (standardized mean difference) comparing children with elevated ePASP to those without. Horizontal lines = width of 95% confidence interval, diamond = standardized mean of random effects distribution, diamond width = width of 95% confidence interval, horizontal lines surrounding diamond = 95% predictive interval.


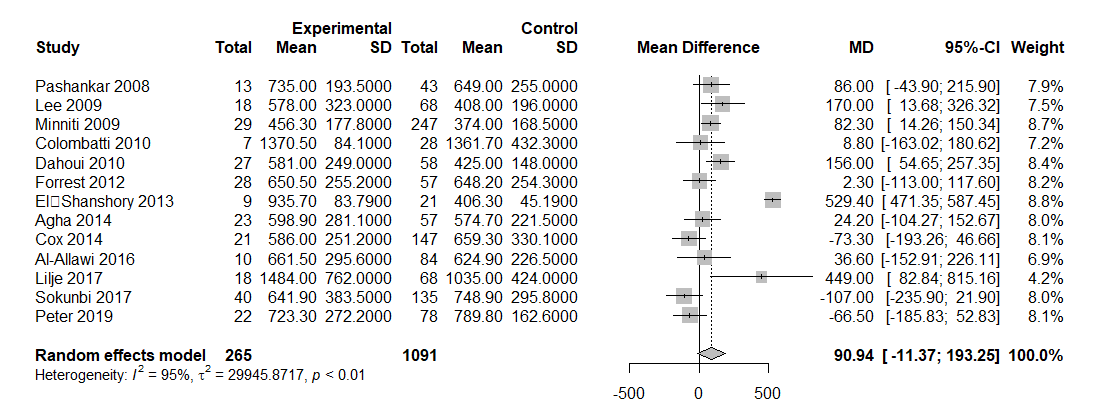


**eFigure 44.** Random-effects meta-analysis of lactate dehydrogenase level (mean difference) comparing children with elevated ePASP to those without. Horizontal lines = width of 95% confidence interval, diamond = mean of random effects distribution, diamond width = width of 95% confidence interval, horizontal lines surrounding diamond = 95% predictive interval.


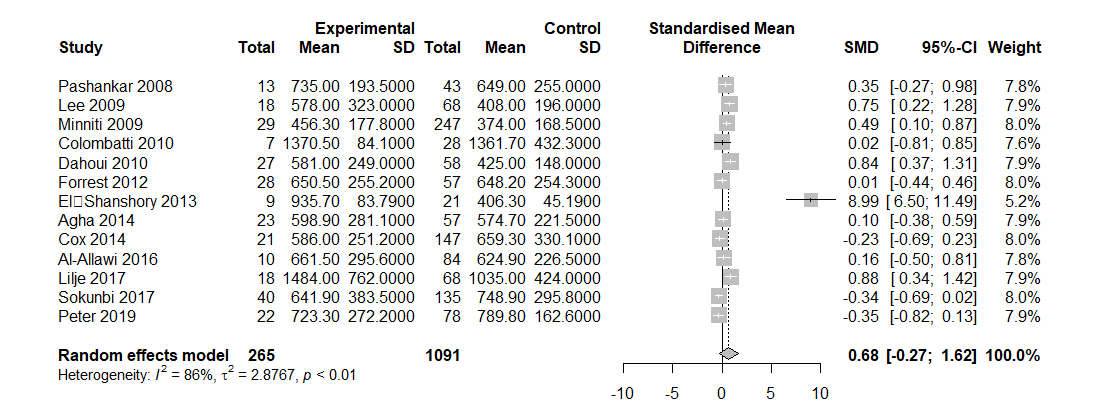


**eFigure 45.** Random-effects meta-analysis of lactate dehydrogenase level (standardized mean difference) comparing children with elevated ePASP to those without. Horizontal lines = width of 95% confidence interval, diamond = standardized mean of random effects distribution, diamond width = width of 95% confidence interval, horizontal lines surrounding diamond = 95% predictive interval.


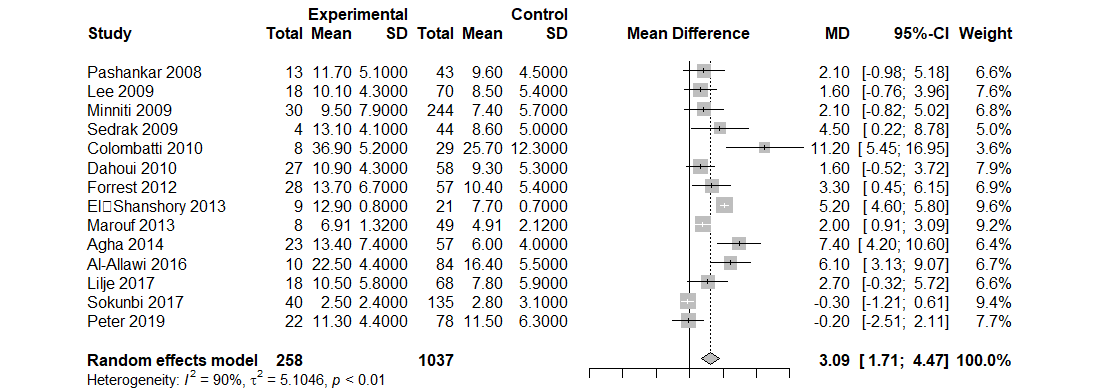


**eFigure 46.** Random-effects meta-analysis of reticulocyte count (mean difference) comparing children with elevated ePASP to those without. Horizontal lines = width of 95% confidence interval, diamond = mean of random effects distribution, diamond width = width of 95% confidence interval, horizontal lines surrounding diamond = 95% predictive interval.


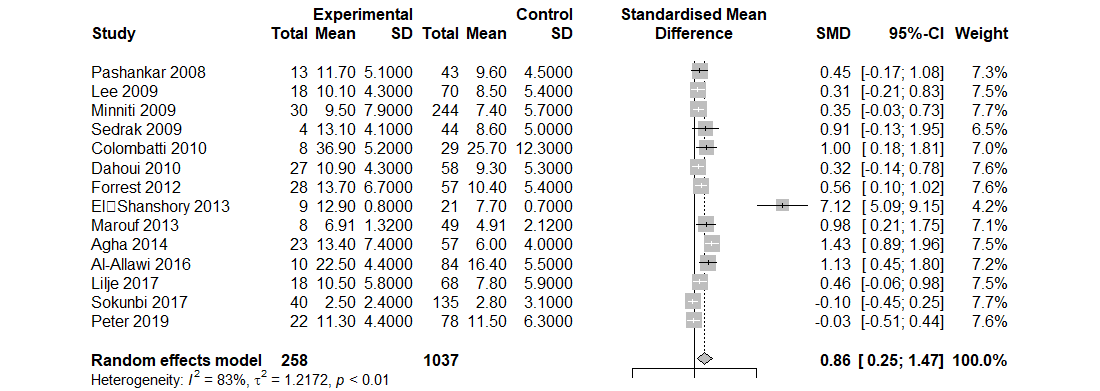


**eFigure 47.** Random-effects meta-analysis of reticulocyte count (standardized mean difference) comparing children with elevated ePASP to those without. Horizontal lines = width of 95% confidence interval, diamond = standardized mean of random effects distribution, diamond width = width of 95% confidence interval, horizontal lines surrounding diamond = 95% predictive interval.
