# Supplementary material for: Global prevalence of elevated estimated pulmonary artery systolic pressure in clinically stable children and adults with sickle cell disease: A systematic review and meta-analysis
Source: PLoS One. 2025 Feb 13;20(2):e0318751. doi: 10.1371/journal.pone.0318751 (PMC11825009; doi:10.1371/journal.pone.0318751)
Supplement: Attachment — Submitted filename: Response to Reviewers.docx [file pone.0318751.s008.docx]

**Date:** 2024-11-21

**Subject**: Response to Reviewer's Comments

**Title:** PLOS ONE

**ID:** PONE-D-24-41837

**Dear Editor**

I sincerely appreciate the editor and reviewers. We believe that the manuscript has been improved. We hope that the revised manuscript has achieved the journal's standards. We submitted the revised manuscript with the reply to the editor and reviewers' comments as point-to-point.

**Editor Comments to Author:**

The reviewers found merit in your study but also identified several areas that need more clarification.  For example, the sickle cell disease genotype and definitions of severity that you have used are not consistent with the literature and should be revised accordingly.  Further discussion is also needed to better understand changes before 2014 and after 2015, particularly since hydroxyurea was not recommended until after this time period.

**Response:** Thank you so much for your comment and attention. I would like to address this misunderatanding that caused for the editor and all the reviewers. The decision for considering the subgroups in the current study was based on the definition of each subgroup. The subgroups including “type of patients” and “severe genotype (HbSS and HbS/β^0^)” was made in order to assess these specific SCD populations accurately. Some use the term ‘‘sickle cell anemia’’ to include both the HbSS and HbS/β^0^ genotypes, however, **based on the definition in the literature,** HbS/β0 thalassemia can be distinguished from sickle cell anemia by analyzing the morphology of red blood cells and the results of hemoglobin electrophoresis [1-3]. So, we considered the subgroup based on the definition in the literature. Also, we considered the subgroup severe genotypes (HbSS and HbS/β^0^) in order to specifically present the prevalence of elevated ePASP in these genotypes and we did not include the studies that did not define the genotypes through hemoglobin electrophoresis.

In order to resolve this misunderstanding that can also cause for the readers, we decided to delete the subgroup “Type of disease” from the entire of the study [we corrected the order of the eFigures in the manuscript] and supplementary file (eTables and eFigures) and we specifically focused on the studies clearly defined the most severe forms of SCD, HbSS and HbS/β^0^ through genotyping.

The definitions of all subgroups were explained in the Method section (Lines 162-176).

We discussed the changes before 2014 and after 2015 in the Discussion section (Lines 474-488 and 469-473).

We revised the severity of elevated ePASP in adults (Table 1).

1. When submitting your revision, we need you to address these additional requirements.

Please ensure that your manuscript meets PLOS ONE's style requirements, including those for file naming. The PLOS ONE style templates can be found at

<https://journals.plos.org/plosone/s/file?id=wjVg/PLOSOne_formatting_sample_main_body.pdf> and

<https://journals.plos.org/plosone/s/file?id=ba62/PLOSOne_formatting_sample_title_authors_affiliations.pdf>

**Response:** Thank you so much for your comment. We modified our manuscript based on these templates.

2. Thank you for stating the following in your Competing Interests section: "None"

Please complete your Competing Interests on the online submission form to state any Competing Interests. If you have no competing interests, please state "The authors have declared that no competing interests exist", as detailed online in our guide for authors at <http://journals.plos.org/plosone/s/submit-now>

This information should be included in your cover letter; we will change the online submission form on your behalf.

**Response:** Thank you so much for your comment. We completed our Competing Interests on the online submission form. We stated th competing interest both in the manuscript (Lines 600-601) and cover letter.

3. We note that there is identifying data in the Supporting Information file <eTables and S2 Supplementary file>. Due to the inclusion of these potentially identifying data, we have removed this file from your file inventory. Prior to sharing human research participant data, authors should consult with an ethics committee to ensure data are shared in accordance with participant consent and all applicable local laws.

Data sharing should never compromise participant privacy. It is therefore not appropriate to publicly share personally identifiable data on human research participants. The following are examples of data that should not be shared:

-Name, initials, physical address

-Ages more specific than whole numbers

-Internet protocol (IP) address

-Specific dates (birth dates, death dates, examination dates, etc.)

-Contact information such as phone number or email address

-Location data

-ID numbers that seem specific (long numbers, include initials, titled “Hospital ID”) rather than random (small numbers in numerical order)

Data that are not directly identifying may also be inappropriate to share, as in combination they can become identifying. For example, data collected from a small group of participants, vulnerable populations, or private groups should not be shared if they involve indirect identifiers (such as sex, ethnicity, location, etc.) that may risk the identification of study participants.

Additional guidance on preparing raw data for publication can be found in our Data Policy (<https://journals.plos.org/plosone/s/data-availability#loc-human-research-participant-data-and-other-sensitive-data>) and in the following article: <http://www.bmj.com/content/340/bmj.c181.long>.

Please remove or anonymize all personal information (<specific identifying information in file to be removed>), ensure that the data shared are in accordance with participant consent, and re-upload a fully anonymized data set. Please note that spreadsheet columns with personal information must be removed and not hidden as all hidden columns will appear in the published file.

**Response:** Thank you so much for your comment. As you stated, we removed the columns with personal information from the supplenetary files (including eTables and eFigures files) and modified our files based on the mentioned changes. Notably, the current S4 file (including a list of excluded studies at full text review with brief report) did not contain any identifying data.

4. As required by our policy on Data Availability, please ensure your manuscript or supplementary information includes the following:

A numbered table of all studies identified in the literature search, including those that were excluded from the analyses.

For every excluded study, the table should list the reason(s) for exclusion.

If any of the included studies are unpublished, include a link (URL) to the primary source or detailed information about how the content can be accessed.

A table of all data extracted from the primary research sources for the systematic review and/or meta-analysis. The table must include the following information for each study:

Name of data extractors and date of data extraction

Confirmation that the study was eligible to be included in the review.

All data extracted from each study for the reported systematic review and/or meta-analysis that would be needed to replicate your analyses.

If data or supporting information were obtained from another source (e.g. correspondence with the author of the original research article), please provide the source of data and dates on which the data/information were obtained by your research group.

If applicable for your analysis, a table showing the completed risk of bias and quality/certainty assessments for each study or outcome.  Please ensure this is provided for each domain or parameter assessed. For example, if you used the Cochrane risk-of-bias tool for randomized trials, provide answers to each of the signalling questions for each study. If you used GRADE to assess certainty of evidence, provide judgements about each of the quality of evidence factor. This should be provided for each outcome.

An explanation of how missing data were handled.

This information can be included in the main text, supplementary information, or relevant data repository. Please note that providing these underlying data is a requirement for publication in this journal, and if these data are not provided your manuscript might be rejected.

**Response:** Thank you so much for your comment. We provided numbered table of all studies identified in the literature search. Also, we provided a table for the excluded studies with the reason for exclusion in Supporting information file, “S3 File. List of excluded studies”. All the studies were published. We provided tables of all data extracted from the primary research sources in Supporting information file, “S6 File. eTables (eTables 1 - 6)”. We mentioned the data extractors, date of data extraction, the eligibility of the included studies in the manuscript. All data or supporting information were obtained from the included studies. We provided the completed risk of bias and quality/certainty assessments for each study in Supporting information file, “S4 File. Risk of bias assessment”. We tried to handle the missing data of the outcome of interest through risk of bias assessment which we devoted two scores of the modified version of the checklist regarding the missing data including item 5 and item 8 of the checklist (Lines 155-156).

5. Please include captions for your Supporting Information files at the end of your manuscript, and update any in-text citations to match accordingly. Please see our Supporting Information guidelines for more information: <http://journals.plos.org/plosone/s/supporting-information>.

**Response:** Thank you so much for your comment. We provided captions for our Supporting Information files at the end of the manuscript, and updateed the in-text citations (Lines 576-583).

Reviewers'comments:

Reviewer's Responses to Questions

Comments to the Author

1. Is the manuscript technically sound, and do the data support the conclusions?

The manuscript must describe a technically sound piece of scientific research with data that supports the conclusions. Experiments must have been conducted rigorously, with appropriate controls, replication, and sample sizes. The conclusions must be drawn appropriately based on the data presented.

Reviewer #1: Partly

Reviewer #2: Yes

Reviewer #3: Yes

**Response:** Thank you so much for your comment and attention. Based on the editor and reviewers’ comments, we did our best to address those comments and we believe that the manuscript has been improved. We have highlighted all the changes in yellow. We hope that the revised manuscript has achieved the journal's standards.

2. Has the statistical analysis been performed appropriately and rigorously?

Reviewer #1: Yes

Reviewer #2: Yes

Reviewer #3: I Don't Know

**Response:** Thank you so much for your attention.

3. Have the authors made all data underlying the findings in their manuscript fully available?

The [PLOS Data policy](http://c05y1x9s.r.us-east-2.awstrack.me/L0/http:%2F%2Fwww.plosone.org%2Fstatic%2Fpolicies.action%23sharing/1/010f0192b4fdabf2-a7d64a8f-491f-48a4-8285-ef3e58f60d3e-000000/hNwbr1IEmtN0HwXmsd765wbeuoI=182) requires authors to make all data underlying the findings described in their manuscript fully available without restriction, with rare exception (please refer to the Data Availability Statement in the manuscript PDF file). The data should be provided as part of the manuscript or its supporting information, or deposited to a public repository. For example, in addition to summary statistics, the data points behind means, medians and variance measures should be available. If there are restrictions on publicly sharing data—e.g. participant privacy or use of data from a third party—those must be specified.

Reviewer #1: Yes

Reviewer #2: Yes

Reviewer #3: Yes

**Response:** Thank you so much for your comment.

4. Is the manuscript presented in an intelligible fashion and written in standard English?

PLOS ONE does not copyedit accepted manuscripts, so the language in submitted articles must be clear, correct, and unambiguous. Any typographical or grammatical errors should be corrected at revision, so please note any specific errors here.

Reviewer #1: Yes

Reviewer #2: Yes

Reviewer #3: Yes

**Response:** Thank you so much for your consideration.

**Reviewer Comments to Author:**

Please use the space provided to explain your answers to the questions above. You may also include additional comments for the author, including concerns about dual publication, research ethics, or publication ethics. (Please upload your review as an attachment if it exceeds 20,000 characters)

**Reviewer 1:**

Comment: Thank you for your efforts to clarify an unclear topic of real significance to the morbidity and mortality of sickle cell disease. Overall, your analysis is well-done, but the groupings do not fully make sense. Specifically sickle cell disease is the overarching diagnosis that includes severe genotypes (HbSS and HbS/beta-zero thalassemia) and these severe genotypes are referred to as "sickle cell anemia." These patients make up the majority of patients with the disease. I have stated this further and with additional comments below.

**Response:** Thank you so much for your comment and attention. I would like to address this misunderatanding that caused for the editor and all the reviewers. The decision for considering the subgroups in the current study was based on the definition of each subgroup. The subgroups including “type of patients” and “severe genotype (HbSS and HbS/β^0^)” was made in order to assess these specific SCD populations accurately. Some use the term ‘‘sickle cell anemia’’ to include both the HbSS and HbS/β^0^ genotypes, however, **based on the definition in the literature,** HbS/β0 thalassemia can be distinguished from sickle cell anemia by analyzing the morphology of red blood cells and the results of hemoglobin electrophoresis [1-3]. So, we considered the subgroup based on the definition in the literature. Also, we considered the subgroup severe genotypes (HbSS and HbS/β^0^) in order to specifically present the prevalence of elevated ePASP in these genotypes and we did not include the studies that did not define the genotypes through hemoglobin electrophoresis.

In order to resolve this misunderstanding that can also cause for the readers, we decided to delete the subgroup “Type of disease” from the entire of the study [we corrected the order of the eFigures in the manuscript] and supplementary file (eTables and eFigures) and we specifically focused on the studies clearly defined the most severe forms of SCD, HbSS and HbS/β^0^ through genotyping.

The definitions of all subgroups were explained in the Method section (162-176).

Comment: Abstract
Background: Suggest rephrasing "stable pediatric and adult" as "stable children and adults"
Results/methods: pediatric patients and patients under 18 years old are usually considered to be the same. I don't see this explained in the methods or presented in the results in the text with this same duplicative description

**Response:** Thank you so much for your attention. "stable pediatric and adult" substituted by "stable children and adults" in the Background subsection (Lines 26-27) and throughout the manuscript.

We also mentioned the definition of children and adults in the Abstract (Line 34) and Method sections (Lines 163-164).

Comment: Introduction
- Both sentences mentioning homozygous vs compound heterozygous sickle cell disease are redundant

**Response:** Thank you so much for your attention. We deleted these sentences.

Comment:

- Regarding the statement about the importance of screening and early detection on page 3, the sickle cell guidelines are mixed regarding the utility of screening ECHOs. The American Society of Hematology does not recommend screening ECHO's for asymptomatic patients. Because of this, even though you include "stable patients" it is not possible to know from your analysis if they are asymptomatic. Screening symptomatic patients would yield higher rates of ePASP, and this difference in testing may explain some of the heterogeneity. If the authors are unable to clarify how many patients were symptomatic, please add this as a limitation.

**Response:** Thank you so much for your attention. We conducted our study based on the previous systematic review and meta-analysis and on patients in clinically stable conditions that the characteristics in detail was that we omitted studies of patients with vaso-occlusive pain crisis or acute chest syndrome, studies of hospitalized patients, patients presenting with cardiopulmonary symptoms during echocardiography to avoid overestimation of elevated pulmonary pressures. Actually, we limited and focused our analysis to SCD patients at steady state during echocardiography. We added this sentence to the limitation section (Lines 512-513).

Comment:

- Why did you include "sickle cell trait" in your search? I would think this should be excluded

**Response:** Thank you so much for your consideration and attention. We included "sickle cell trait" keyword based on the results of the previous studies. Aliyu et al. conducted a study in 2008 with 208 hydroxyurea naive Nigerian SCD patients at steady state and 94 healthy controls (patients with documented haemoglobin AA and AS genotypes). They discovered that 7% of controls had elevated systolic pulmonary artery pressure, defined as jet velocity ≥2.5 m/second (estimated systolic pulmonary artery pressure ≥30 mm Hg) [4].

Literature indicates that individuals with sickle cell trait (SCT) typically do not show symptoms, but recent studies on large U.S. groups have shown that SCT raises the risk of venous thromboembolism, especially pulmonary embolism, by 1.5-2.0 times in African Americans compared to those without SCT [5, 6]. Moreover, blood viscosity and estimated RBC rigidity were found to be very high compared to the values of the general population. Furthermore, SCT could exacerbate multiple factors that increase the risk of developing type 2 diabetes related vascular complications, including arterial stiffness, blood hyperviscosity, the loss of vasodilatory capacity, advanced glycation end products accumulation, and increased E-selectin expression [7, 8].

Comment:

- There may be a typo in the description of the study dichotomy by dates "before 2015 and after 2014"?

**Response:** Thank you so much for your attention. In order to resolve this issue, we decided to change the name of this subgroup to the previous meta-analysis (studies published from 2000 to 2014) and current meta-analysis (studies published from 2015 to the end of 2023) and we defined this subgroup in the Method section (Lines 175-176).

Comment:

- The two different groups of "type of disease (SCA and SCD)" vs "genotype severity of SCD" does not make sense to me. Sickle cell anemia typically is used to describe the severe SCD genotypes (HbSS and HbS/beta zero thalassemia). These seem redundant.

**Response:** Thank you so much for your comment. We used to discuss it in the first comment. In order to resolve this misunderstanding that can also cause for the readers, we decided to delete the subgroup “Type of disease” from the entire of the study [we corrected the order of the eFigures in the manuscript] and supplementary file (eTables and eFigures) and we specifically focus on the studies clearly defined the most severe forms of SCD, HbSS and HbS/β^0^ through genotyping. The definitions of all subgroups were explained in the Method section (Lines 162-176).

Comment:

- Additionally, patients with SCA (aka with more severe genotypes) are also patients with SCD (which includes all genotypes)

**Response:** Thank you so much for your attention. We used to discuss it in the first comment. We decided to delete the subgroup “Type of disease” from the entire of the manuscript [we corrected the order of the eFigures in the manuscript] and supplementary file (eTables and eFigures) and we just specifically focused on the studies clearly defined the most severe forms of SCD, HbSS and HbS/β0 through genotyping. The definitions of all subgroups were explained in the Method section (162-176).

Comment:

- I would suggest the authors revisit their analysis and either redefine their groups appropriately or remove the attempt to stratify studies based on genotype/disease severity, since patients with severe genotype SCD (aka with SCA) represent the majority of patients with sickle cell disease

**Response:** Thank you so much for your attention. We used to discussed it in the first comment. We decided to delete the subgroup “Type of disease” from the entire of the manuscript [we corrected the order of the eFigures in the manuscript] and supplementary file (eTables and eFigures) and we just specifically focused on the studies clearly defined the most severe forms of SCD, HbSS and HbS/β0 through genotyping. The definitions of all subgroups were explained in the Method section (162-176).

Comment:

Results
- "western" and "non-western" countries does not provide much meaning to readers. Consider stating the countries or continents.

**Response:** Thank you so much for your attention.

Constantly stating the names of the countries and comparing them with each other takes up a lot of space in the manuscript and also becomes boring for the readers. Moreover, the expression of the countries by the name of the continents can make readers make mistakes while interpreting and generalizing the results. Therefore, we defined this subgroup in the Method section, Definitions subsection. Studies with country of origin in United States and Europe considered as Western countries and with country of origin in Africa, Middle East, South America and Caribbean considered as Non-Western countries (Lines 173-174).

Comment:

- 3.3 "prevalence of elevated ePASP in adults and pediatrics." Pediatric is an adjective. Consider changing pediatrics to children or adding the word patients to the end

**Response:** Thank you so much for your attention. We changed “pediatrics” to “children” throughout the manuscript and supplementary files.

**Reviewer 2:**

Comment:

Summary:
This manuscript by Ghazaiean et al is a thorough and extensive systematic review and meta-analysis evaluating the prevalence of elevated estimated pulmonary artery systolic pressure (ePASP) in pediatrics and adults with sickle cell disease (SCD) worldwide. The authors included 79 primary studies comprising 6,256 pediatric and 6,582 adult patients with SCD from 22 countries. They found that the prevalence of elevated ePASP was 21.8% in the pediatric population and 30.6% in adults. There were differences in the prevalence of elevated ePASP in subgroup analyses with age, sex, severity of SCD genotype, and year (before 2015 and after 2014). Those with elevated ePASP had significant differences in clinical characteristics and laboratory values compared to those without elevated ePASP. The authors concluded that there is a high prevalence of elevated ePASP in patients with SCD.
Elevated ePASP on a TTE is an important approach to early detection/screening of pulmonary hypertension, a major risk factor for morbidity and mortality in SCD. There was a systematic review on this in 2015 (Caughey et al, BJH), and this updated version 9 years later features the addition of 34 more studies (2,925 more adults & 3,804 more pediatric patients). This is a much needed updated review given there have been various improvements in the management of SCD including in treatment and technology. Given that there is no consensus on optimal screening practices for PH, it is important to first establish the scope of elevated ePASP in the SCD population. Thus, it is critical in the field of SCD to publish this data given the implications for health policy and updated guidelines. The strengths of this paper are its thoroughness, great tables and figures, and interesting subgroup analyses. In the discussion section, there was also a comprehensive review on the pathophysiology of pulmonary hypertension. However, there are some areas where this manuscript could be strengthened, to further enhance and convey the importance of elevated ePASP screening.

**Response:** Thank you so much for your attention. I appreciate your consideration.

Major issues:

Comment:
• Please cite the previous meta-analysis on ePASP and sickle cell disease (Caughey MC, Poole C, Ataga KI, Hinderliter AL. Estimated pulmonary artery systolic pressure and sickle cell disease: a meta-analysis and systematic review. Br J Haematol. 2015 Aug;170(3):416-24. doi: 10.1111/bjh.13447. Epub 2015 Apr 9. PMID: 25854714.) The authors mention this paper in the introduction and it is a crucial cutoff point in the analysis (before 2015, after 2014), so it should be acknowledged. This paper can also be referenced to highlight in the discussion any new findings from the analysis, and possible explanations. It is great that this work builds on work done previously.

**Response:** Thank you so much for your attention. We cited the previous meta-analysis both in the Introduction and Discussion sections and also highlighted in the discussion the new findings from the analysis.

Comment:

• The definitions for sickle cell anemia (SCA) versus sickle cell disease (SCD) and mild/moderate/severe TRV, should be clearly defined as it is not entirely apparent who is included in these groups, which makes the paper confusing to read. Does SCA just include HbSS or did it also include HbSbeta-0-thalassemia? For severity of ePASP, what were the cutoffs? These definitions are important as the authors later make recommendations on screening practices.

**Response:** Thank you so much for your attention. I would like to address this misunderatanding that caused for the editor and all the reviewers. The decision for considering the subgroups in the current study was based on the definition of each subgroup. The subgroups including “type of patients” and “severe genotype (HbSS and HbS/β^0^)” was made in order to assess these specific SCD populations accurately. Some use the term ‘‘sickle cell anemia’’ to include both the HbSS and HbS/β^0^ genotypes, however, **based on the definition in the literature,** HbS/β0 thalassemia can be distinguished from sickle cell anemia by analyzing the morphology of red blood cells and the results of hemoglobin electrophoresis [1-3]. So, we considered the subgroup based on the definition in the literature. Also, we considered the subgroup severe genotypes (HbSS and HbS/β^0^) in order to specifically present the prevalence of elevated ePASP in these genotypes and we did not include the studies that did not define the genotypes through hemoglobin electrophoresis.

In order to resolve this misunderstanding that can also cause for the readers, we decided to delete the subgroup “Type of disease” from the entire of the study [we corrected the order of the eFigures in the manuscript] and supplementary file (eTables and eFigures) and we specifically focused on the studies clearly defined the most severe forms of SCD, HbSS and HbS/β^0^ through genotyping.

The definitions of all subgroups were explained in the Method section (Lines 162-176).

Comment:
• The authors have many interesting findings such as the decrease in prevalence of ePASP in pediatrics after 2014 compared to before 2015, lab correlations, sex differences, and non-Western countries versus Western countries – the manuscript could be enhanced with further discussion of at least a few of these findings if not all of them. TTE may be more accessible in non-Western countries, which may be an important factor as to why this is an important screening tool.

**Response:** Thank you so much for your comment. We discussed the mentioned findings in the Discussion section (Lines 414-419, Lines 427-440, Lines 452-461, Lines 462-473, Lines 474-488, and Lines 489-502).

Minor issues:

Comment:
• In the introduction paragraph, the following sentence is ambiguous to me as it makes right heart catheterizations seem like the initial and sole method of diagnosing pulmonary hypertension for some cases, but it is typically done after a screening TTE: “This underscores the importance of screening and early detection of PASP elevations through non-invasive methods, such as trans-thoracic echocardiography, rather than relying solely on invasive right heart catheterization (RHC) for diagnosis.”

**Response:** Thank you so much for your attention. We improved the Introduction section and we modified this sentence (Lines 63-81).

Comment:

• In the introduction, when mentioning the compound heterozygous state, you should clarify that HbSC is an example of a compound heterozygous state, as the way it is written know suggests it is the only heterozygous state.

**Response:** Thank you so much for your time and attention. According to the one of the reviewer’s comment, we used to delete this sentence from the Introduction section.

Comment:

• In the introduction, would clarify that a RHC is required for diagnosis of PH. TRV > 2.5 is a great screening tool but is not always indicative of pulmonary hypertension (sensitivity increases with higher TRV), however, given that elevated TRV is associated with increased mortality, it is still an important clinical feature to assess, particularly since it is noninvasive.

**Response:** Thank you so much for your attention. We improved the Introduction section and we modified this sentence (Lines 63-81).

Comment:

• It feels as though the author of sections 3.3.1-3.3.2 was different from the author of 3.3.3- 3.3.7. The use of the phrase, “meticulously assessed” in the latter sections do not appear consistent with the writing style of previous sections. Would recommend being more consistent and possibly removing a few of the “meticulously” phrases as it is repetitive.

**Response:** Thank you so much for your attention. We edited the mentioned subsections in the Result section.

Comment:

• In section 3.4, please highlight some important takeaways from table 3.

**Response:** Thank you so much for your attention. We created two subsections for the MD and SMD of the clinical and laboratory findings in the section 3.4. We highlighted some important findings in these sections (Lines 360-365 and Lines 367-373).

Comment:

• In the discussion, you mention, “Thankfully, there has been a slightly meaningful decrease in the prevalence of elevated ePASP in children since 2015, as compared to the previous rate.” Why is this 2015 date important? The meta-analysis had been published at that time, but those results would not change clinical practice. What do you think could explain this finding?

**Response:** Thank you so much for your attention. This research builds on work done previously and comparing our results with previous findings was one of the subgroups of our study. In the previous meta-analysis that published in 2015, most of the subgroups that were selected were often in the form of cut-off due to the included studies limitations, for example proportion of females [≤51% and >51%], proportion with HbSS or HbSb^0^ [≤84% and >84%], mean haemoglobin [≤88 g/l and >88 g/l], proportion treated by hydroxycarbamide [≤36% and >36%], and even 6MWT.

However in our study, we tried to use specific subgroups and without cut-off point as much as possible. We also tried to compare the mean ± SD values of the clinical and laboratory findings in patients with elevated ePASP than without elevated ePASP in order to present precise and real difference among these patients in different age groups. As a result, based on the subgroups of this study and the previous study, discussing and comparing our results with previous meta-analysis results can confuse readers and cause misinterpretation of the findings. However, we discussed our findings and the previous meta-analysis results as much as possible.

Comment:

• In the discussion, you write “The prevalence of elevated ePASP is notably higher in pediatric males compared to females. However, this trend reverses in adults, where females exhibit a greater prevalence than males.” Please discuss why this is important or what could explain this.

**Response:** Thank you so much for your attention. We discussed the potential reasons of these differences in the Discussion section (Lines 452-461).

Comment:

• In the discussion, you write, “Clinical and laboratory analyses of pediatric and adult patients with SCD revealed significant mean differences in various characteristics, including age, oxygen saturation, Hb, HbF, WBC count, and reticulocyte levels between those with elevated ePASP and those without.” Please discuss why this is important.

**Response:** Thank you so much for your attention. We discussed the importance of clinical and laboratory analyses of children and adult patients with SCD in the Discussion section (Lines 414-419 and Lines 438-440) and also we mentioned them throughout the manuscript while we discussed each subgroup.

Comment:

• On the last sentence of page 26: “Our study proposes a noninvasive screening protocol for PH in children with SCD, which may facilitate the identification of a specific subgroup of patients who would benefit from further evaluation via catheterization. Implementing a strategy to recognize children at risk for hemolysis-associated vasculopathy through a straightforward biomarker, such as elevated TRV, followed by preventive interventions like hydroxycarbamide, transfusions, or other methods to mitigate hemolysis, could significantly enhance survival outcomes in SCD.” – what is your screening protocol? Screen every pediatric patient with TTE? What age? How often? Is there a TRV cutoff? Should we follow the ATS guidelines or ASH guidelines? It would be important to acknowledge there is no consensus. TRV is not a straightforward biomarker, as you mention later in the limitation section – TTEs are highly tech and reader dependent. It would also be important to mention the next steps if the TRV is elevated, including obtaining a pro-NT-BNP, assessing 6 minute walk distance, and a RHC. It would be interesting to discuss any differences in the approach of non-Western versus Western countries given the amount of resources in each setting. It was thoughtful to include potential treatment strategies.

**Response:** Thank you so much for your attention. We modified those sentences (Lines 427-440, Lines 462-473 and Lines 474-488).

Comment:

• In the limitations section, I would not recommend listing each limitation with “firstly,” “secondly,” “thirdly,” … “twelfthly” as this makes the paragraph choppy and less interesting to read.

**Response:** Thank you so much for your attention. We edited listing each limitation.

Comment:

• Section 2.7 Risk of bias assessment – please write in past tense, as you did for the other paragraphs in this section.

**Response:** Thank you so much for your attention. We rewrote this section in past tense (Line 158).

Comment:

• Table 3 is great. Why is “No of studies/total listed” twice?

**Response:** Thank you so much for your comment. We edited this list both in Table 3 and Table 4.

Comment:

• I am surprised that there were no differences observed between patients from Western and non-Western countries? Why do you think that is?

**Response:** Thank you so much for your attention. We discussed this finding in the Discussion section (Lines 462-473).

**Reviewer 3:**

Comment: Abstract
1. In the results section, can the authors clarify the definition of SCA and severe genotype? SCA and severe genotype both typically refer to HbSS and HbSbeta zero thalassemia but both have different prevalence rates and confidence intervals. The statement below is from the paper…
“When stratified by disease type, the prevalence rates for patients with sickle cell anemia (SCA) were 16.55% (95% CI: 12.49 to 20.61) in pediatric patients and 36.06% (95% CI: 31.53 to 40.6) in adults. The prevalence of elevated ePASP among studies with severe SCD genotype was found to be 19.45% (95% CI: 14.95 to 23.95) in pediatric patients and 29.55% (95% CI: 24.21 to 34.89) in adults.”

**Response:** Thank you so much for your comment and attention. I would like to address this misunderatanding that caused for the editor and all the reviewers. The decision for considering the subgroups in the current study was based on the definition of each subgroup. The subgroups including “type of patients” and “severe genotype (HbSS and HbS/β^0^)” was made in order to assess these specific SCD populations accurately. Some use the term ‘‘sickle cell anemia’’ to include both the HbSS and HbS/β^0^ genotypes, however, **based on the definition in the literature,** HbS/β0 thalassemia can be distinguished from sickle cell anemia by analyzing the morphology of red blood cells and the results of hemoglobin electrophoresis [1-3]. So, we considered the subgroup based on the definition in the literature. Also, we considered the subgroup severe genotypes (HbSS and HbS/β^0^) in order to specifically present the prevalence of elevated ePASP in these genotypes and we did not include the studies that did not define the genotypes through hemoglobin electrophoresis.

In order to resolve this misunderstanding that can also cause for the readers, we decided to delete the subgroup “Type of disease” from the entire of the study [we corrected the order of the eFigures in the manuscript] and supplementary file (eTables and eFigures) and we specifically focus on the studies clearly defined the most severe forms of SCD, HbSS and HbS/β^0^ through genotyping.

The definitions of all subgroups were explained in the Method section (Lines 162-176).

Comment:

Introduction
1. Can the authors elaborate more on the importance of elevated PASP in the SCD population in the introduction? For example, multiple studies exist linking elevated TRV with increased morbidity in children and mortality in adults. This was more clearly explained in the discussion but would have been helpful in the introduction to carry the reader along.

**Response:** Thank you so much for your attention. We explained the importance of elevated PASP in the SCD population in the introduction section (Lines 63-81).

Comment:

2. Sickle cell anemia (SCA) conventionally refers to HbSS and HbSbeta zero thalassemia. Also referred to as “severe genotype.” However, the authors report different values for SCA and “severe genotype.” Please clarify definition.

**Response:** Thank you so much for your attention. As we previously explanied in the first comment, in order to resolve this misunderstanding that can also cause for the readers, we decided to delete the subgroup “Type of disease” from the entire of the study [we corrected the order of the eFigures in the manuscript] and supplementary file (eTables and eFigures) and we specifically focus on the studies clearly defined the most severe forms of SCD, HbSS and HbS/β^0^ through genotyping. We also created a subsection in the Method section for all the definitions. The definitions of all subgroups were explained in the Method section (Lines 162-176).

Comment:

Results
1. Since the study involved such robust statistical analysis, was a biostatistician involved in this study? I do not see a biostatistician listed among the authors.

**Response:** Thank you so much for your comment. Dr. Hadi Darvishi-Khezri was the biostatistician of the current study. He works as a PhD by Research at Mazandaran University of Medical Sciences. Here’s the profile page of Dr. Hadi Darvishi-Khezri (https://www.researchgate.net/profile/Hadi-Darvishi-Khezri).

Comment:

2. Were there any echo complications seen in those patients with elevated PASP like diastolic dysfunction? Or were studies with these complications completely excluded during the review process?

**Response:** Thank you so much for your attention and comment.

Diastolic dysfunction of the left ventricle was found to be an independent factor in the development of pulmonary hypertension in SCD [9]. Diastolic dysfunction is frequently seen in patients with sickle cell disease and is probably a consequence of the disease's pathophysiology and severity [9, 10].

Some studies mentioned echo complications, including diastolic dysfunction in their studies and some studies did not mention and most studies did not determine echo complications, including diastolic dysfunction in those patients with elevated PASP. It is not possible from the included studies to draw precise assessment through considering the echo complications. Therefore, due to this difference in the included studies that existed both in the studies published in the previous meta-analysis and in the studies published after 2015, we did not consider it in the protocol of our study due to the fact that it was not possible to present an accurate assessment. That's why we mentioned this issue in the Limitation section (Lines 519-522).

Comment:

- Were other parameters such as normal ejection fraction, left ventricular wall thickness, etc evaluated?

**Response:** Thank you so much for your time and attention. Unfortunately, as we mentioned in the protocol of our study, we just evaluated the findings that were presented in Table 1-4.

Comment:

3. Did any cohort have right heart catheterization data? It will be helpful to include this data to demonstrate a correlation between elevated PASP confirmed by right heart catheterization.

**Response:** Thank you so much for your attention. As we mentioned in the protocol of our study, we just evaluated the findings that were represented in Table 1 to Table 4. However, according to this comment, we mentioned the prevalence of PH confirmed by right heart catheterization among patients with elevated PASP (four studies in adults age group) in the Discussion section (only one study evaluated the prevalence of PH confirmed by right heart catheterization in children with elevated PASP) (Lines 421-423).

Comment:

4. I found it interesting that there was no statistically significant difference in the prevalence rates of elevated PASP across Western and non-Western countries. Can the authors provide a possible reason for this?

**Response:** Thank you so much for your attention. We discussed this finding in the Discussion section (Lines 462-473).

Comment:

5. Why were the date ranges selected for analysis? “Studies published prior to 2014” and “prior to 2015” were specifically selected as the cut-off year in the selection.

**Response:** Thank you so much for your comment. We selected the cut-off year in order to clarify the charateristics of the studies published between two periods and compare the prevalence of elevated ePASP between the results of the previous meta-analysis and the studies published after that both in children and adults. We mentioned the definitions of all subgroups in the Method section (Lines 162-176).

Comment:

Discussion
1. Can the authors put forward a hypothesis why there has been a “slightly meaningful decrease” in the prevalence of elevated PASP in children since 2015? It is interesting because hydroxyurea did not become very widely adopted until FDA approved hydroxyurea for use in children in 2017. Other factors may be in play.

**Response:** Thank you so much for your attention. We discussed this finding in the Discussion section (Lines 474-488 and Lines 469-472).

Comment:

2. The paper ostensibly sought to estimate the global prevalence of elevated estimated pulmonary artery systolic pressure in children and adults with SCD, but the discussion devotes large sections to tricuspid regurgitation velocity (TRV). Is the argument that PASP may be superior to TRV in estimating pulmonary hypertension or are they equally acceptable alternatives? Does one measurement parameter have any advantage over the other specifically for estimating PH in SCD?

**Response:** Thank you so much for your attention and comment. We discussed the mentioned issues and addressed the advantages and drawbacks in the discussion section (Lines 489-502).

Comment:

4. Instead of using Firstly to twelfthly to highlight limitations, perhaps the authors can combine some limitations and use other introductory words such as “Furthermore” to introduce the other limitations.

**Response:** Thank you so much for your attention. We combined some limitations and we edited listing each limitation.

6. PLOS authors have the option to publish the peer review history of their article. If published, this will include your full peer review and any attached files.

If you choose “no”, your identity will remain anonymous but your review may still be made public.

**Response:** Thank you so much. According to your comment, we agree to publish the peer review history.

** I have a lot of respect for the time and attention of the editor and the reviewers have given to this manuscript and I wish all of them the best.

Sincerely,

Corresponding author: dr.mobin.gh@gmail.com

Address: Mazandaran University of Medical Science, Bou Ali Sina Hospital, Pasdaran Boulevard, Sari, Mazandaran Province, Iran.

PO BOX: 481675793

Tel & Fax: +98(0)11-33347837, +98(0)11-33349672

**References**

1.	Pincus, M.R., *Henry's clinical diagnosis and management by laboratory methods*. 2021: Elsevier.

2.	Vichaka Fanestil BS, M. and M. Carleen Van Siclen MS, *Differentiation Between Sickle Cell Anemia and S/[Beta]^ sup 0^ Thalassemia.* Labmedicine, 2015. **46**(3): p. E79.

3.	Quinn, C.T., *Minireview: clinical severity in sickle cell disease: the challenges of definition and prognostication.* Experimental biology and medicine, 2016. **241**(7): p. 679-688.

4.	Aliyu, Z.Y., et al., *Prevalence and risk factors for pulmonary artery systolic hypertension among sickle cell disease patients in Nigeria.* American journal of hematology, 2008. **83**(6): p. 485-490.

5.	Austin, H., et al., *Sickle cell trait and the risk of venous thromboembolism among blacks.* Blood, The Journal of the American Society of Hematology, 2007. **110**(3): p. 908-912.

6.	Folsom, A.R., et al., *Prospective study of sickle cell trait and venous thromboembolism incidence.* Journal of Thrombosis and Haemostasis, 2015. **13**(1): p. 2-9.

7.	Diaw, M., et al., *Sickle cell trait worsens oxidative stress, abnormal blood rheology, and vascular dysfunction in type 2 diabetes.* Diabetes Care, 2015. **38**(11): p. 2120-2127.

8.	Connes, P., *Blood rheology and vascular function in sickle cell trait and sickle cell disease: From pathophysiological mechanisms to clinical usefulness.* Clinical Hemorheology and Microcirculation, 2023(Preprint): p. 1-19.

9.	Sachdev, V., et al., *Diastolic dysfunction is an independent risk factor for death in patients with sickle cell disease.* Journal of the American College of Cardiology, 2007. **49**(4): p. 472-479.

10.	Hankins, J.S., et al., *Ventricular diastolic dysfunction in sickle cell anemia is common but not associated with myocardial iron deposition.* Pediatric blood & cancer, 2010. **55**(3): p. 495-500.
