# Supplementary material for: Global prevalence of elevated estimated pulmonary artery systolic pressure in clinically stable children and adults with sickle cell disease: A systematic review and meta-analysis
Source: PLoS One. 2025 Feb 13;20(2):e0318751. doi: 10.1371/journal.pone.0318751 (PMC11825009; doi:10.1371/journal.pone.0318751)
Supplement: Attachment — Submitted filename: Response_to_Reviewers_auresp_2.docx [file pone.0318751.s009.docx]

**Date:** 2025-01-16

**Subject**: Response to Reviewer's Comments

**Title:** PLOS ONE

**ID:** PONE-D-24-41837R1

**Dear Editor**

I sincerely appreciate the editor and reviewers. We believe that the manuscript has been improved. We hope that the revised manuscript has achieved the journal's standards. We submitted the revised manuscript with the reply to the editor and reviewers' comments as point-to-point.

Journal Requirements:

Please review your reference list to ensure that it is complete and correct. If you have cited papers that have been retracted, please include the rationale for doing so in the manuscript text, or remove these references and replace them with relevant current references. Any changes to the reference list should be mentioned in the rebuttal letter that accompanies your revised manuscript. If you need to cite a retracted article, indicate the article’s retracted status in the References list and also include a citation and full reference for the retraction notice.

**Response:** Thank you so much for your comment and attention. We reviewed the reference list that was complete and correct. No cited papers have been retracted. According to the reviewer’s comment regarding the improvement in some parts of the Discussion section, some references added to the manuscript which have been highlighted in yellow in the “References section”. (Lines 767 to 805 and Lines 821 to 832 and Lines 874 to 896 and Lines 899 to 900 and Lines 902 to 906 and Lines 909 to 917)

Reviewers' comments:

Reviewer's Responses to Questions

**Comments to the Author**

1. If the authors have adequately addressed your comments raised in a previous round of review and you feel that this manuscript is now acceptable for publication, you may indicate that here to bypass the “Comments to the Author” section, enter your conflict of interest statement in the “Confidential to Editor” section, and submit your "Accept" recommendation.

Reviewer #2: (No Response)

Reviewer #3: All comments have been addressed

**Response:** Thank you so much for your comment. We hope that the revised manuscript has achieved the journal's standards.

2. Is the manuscript technically sound, and do the data support the conclusions?

The manuscript must describe a technically sound piece of scientific research with data that supports the conclusions. Experiments must have been conducted rigorously, with appropriate controls, replication, and sample sizes. The conclusions must be drawn appropriately based on the data presented.

Reviewer #2: Yes

Reviewer #3: Yes

**Response:** Thank you so much for your comment.

3. Has the statistical analysis been performed appropriately and rigorously?

Reviewer #2: Yes

Reviewer #3: Yes

**Response:** Thank you so much for your comment.

4. Have the authors made all data underlying the findings in their manuscript fully available?

The [PLOS Data policy](http://track.editorialmanager.com/CL0/http:%2F%2Fwww.plosone.org%2Fstatic%2Fpolicies.action%23sharing/1/010f019448f6cb1d-246c11b0-dd28-460f-98ef-089bd149f2df-000000/DHFguwN2kP9swGPZd9x1byYjfTmLX_mSRrlNIckl4u8=193) requires authors to make all data underlying the findings described in their manuscript fully available without restriction, with rare exception (please refer to the Data Availability Statement in the manuscript PDF file). The data should be provided as part of the manuscript or its supporting information, or deposited to a public repository. For example, in addition to summary statistics, the data points behind means, medians and variance measures should be available. If there are restrictions on publicly sharing data—e.g. participant privacy or use of data from a third party—those must be specified.

Reviewer #2: Yes

Reviewer #3: Yes

**Response:** Thank you so much for your comment.

5. Is the manuscript presented in an intelligible fashion and written in standard English?

PLOS ONE does not copyedit accepted manuscripts, so the language in submitted articles must be clear, correct, and unambiguous. Any typographical or grammatical errors should be corrected at revision, so please note any specific errors here.

Reviewer #2: Yes

Reviewer #3: Yes

**Response:** Thank you so much for your comment.

6. Review Comments to the Author

Please use the space provided to explain your answers to the questions above. You may also include additional comments for the author, including concerns about dual publication, research ethics, or publication ethics. (Please upload your review as an attachment if it exceeds 20,000 characters)

Reviewer #2: The revised manuscript is greatly improved although there are a few small points that should be addressed prior to being published. Here are my comments below.

1. In the abstract, lines 39-41 and the second conclusion (“There has been a slightly meaningful reduction in the prevalence of elevated ePASP among children since 2014) is an overstatement, so I would recommend removing these, particularly since it was not explained in the discussion.

**Response:** Thank you so much for your comment and attention. We removed these sentences.

2. In the abstract, lines 37-38, “Age specific analysis… in adults” does not add to the results section and can be removed.

**Response:** Thank you so much for your attention. We agree and we removed this sentence from the result subsection of the Abstract section.

3. In the introduction, lines 64-72, the authors wrote: “The TRV cutoff of >=2.5m/s seems inappropriate… (It is important to note that NT-pro-BNP levels can be deceptive in a condition of renal insufficiency)” should not be in the introduction. Discussing the ambiguity of TRV measurements and cutoffs in the introduction weakens the premise of this meta-analysis (that people use TRV measurements to estimate ePASP), and should instead be in the limitations section.

**Response:** Thank you so much for your comment and consideration. We removed these sentences from the introduction section and added them to the Limitation section. (Lines 603 to 607)

4. In the introduction and the rest of the manuscript, please be consistent with “TRV” and “TRJV” – are these the same? Only TRJV was defined in line 64 as tricuspid regurgitant jet velocity. Also please define the abbreviation ePASP in the introduction as well – PASP was defined in line 62, but ePASP appears in 87 and the reader may not know what the “e” stands for.

**Response:** Thank you so much for your consideration. We resolved this misunderstanding through the whole manuscript which is highlighted in yellow. We also defined the abbreviation of ePASP in the introduction. We added the abbreviation to the Abbreviation section. (Line 75 and Line 632)

5. In the definition section, line 167, what is SPAP? Please define.

**Response:** Thank you so much for your comment and attention. We defined SPAP (systolic pulmonary artery pressure) in the definition section. We also added the abbreviation to the Abbreviation section. (Line 155 and Line 633)

6. For tables 1 and 2, they would be strengthened by including the number of patients used to calculate the prevalence rate. You mention using thousands of patients in the meta-analysis, so it would strengthen the validity of the table to show these numbers here as well.

**Response:** Thank you so much for your attention. We added new columns to the both Tables 1 and 2 called “No of patients/total”. We also added this footnote “This column represents the number of patients used to calculate the prevalence rate. Detailed information regarding the calculation of the prevalence values of each study is mentioned in S7 File. eFigures” to the both Tables.” (Line 228, Lines 233 to 234, Line 245, and Lines 250 to 251)

7. For the results section, I am a little perplexed by the sex-specific analysis. Are the authors saying that among male children, the rate of elevated ePASP is 60.35% (because that is quite alarming) or are the authors saying that among children with elevated ePASP, the percentage of males is 60.35%? Similarly, for the adults, is the prevalence of elevated ePASP among male adults with SCD 45.6%? - because that is what is implied by the table. The discussion on sex (lines 452-461) may have to be adjusted if the prevalence rates change upon further re-analysis.

**Response:** Thank you so much for your attention. We used to mention on the heading related to this finding “Prevalence of elevated ePASP based on the age and sex of the patients with elevated ePASP in children and adults” in the Result section (Lines 255 to 256), so we provided the prevalence values related to the sex among SCD patients with elevated ePASP not among the SCD patients. We modified the abstract section. We also added this footnote “The prevalence values based on the sex were calculated among SCD patients with elevated ePASP.” to the both Tables 1 and 2 and also modified the sentence in the Discussion section in order to avoid any misunderstanding. (Lines 37 to 38, Line 232, Line 249, Line 264, Line 270, Line 498, and Line 505)

8. Please be consistent in what is statistically significant. If your cutoff is p<=0.05, please do not write that p=0.06 is marginally significant, as this can be misleading. Instead, for the analyses where p=0.06 (variation in heterogeneity indices, children’s ePASP prevalence before and after 2015), you can say that numerically there was a trend towards some specific direction, but it was not statistically significant.

**Response:** Thank you so much for your attention. We edited the manuscript based on this comment. (Lines 264 to 265, Line 268, and Lines 345 to 346)

9. In table 3, why are the P-values of the mean difference and standardized mean difference columns separated by the columns “heterogeneity/ I-square and p-value”? If the model is the same for all of the studies, this column can be removed.

**Response:** Thank you so much for your consideration and attention. The p-value mentioned in the tables 3 and 4 under the column of “Heterogeneity” relates to the “Test of heterogeneity”. To determine whether significant heterogeneity exists, readers should look for the P value of the test of heterogeneity. A high P value is good news because it suggests that the heterogeneity is insignificant and it can guide readers while interpreting the results. We also added footnotes to the Tables 3 and 4. (Lines 384 to 385 and Lines 400 to 401)

10. In the discussion, lines 422-424, the authors note that 22-46% of patients with elevated PASP actually had PH. This should be highlighted as a limitation of using ePASP from TTEs – that TTEs are a good screening tool but do not necessarily equate to pulmonary hypertension. However, there are studies, as the author mentioned that elevated TRVs do portend worse outcomes, so this can be highlighted as well.

**Response:** Thank you so much for your attention. We added this sentence to the limitation section (Lines 612 to 614). We also discussed the studies that elevated TRVs do portend worse outcomes. (Lines 459 to 473)

11. In the discussion, lines 474-488: this paragraph is confusing and hard to follow. The first sentence states that the prevalence of elevated ePASP in children decreased after 2015, so I was expecting a paragraph about why the authors thought this finding could be valid and true. However, it is unclear how HbF and other factors that affect disease severity in SCD relate to a lower prevalence of ePASP in children, specifically.

**Response:** Thank you so much for your comment. We modified the mentioned paragraph and we discussed the potential factors as well. (Lines 523 to 568)

12. The discussion should also highlight the interesting findings from the subgroup analyses and how these factors could confound the TTE findings (severe anemia, low O2, etc) or how these could potentially help figure out who to screen (elevated WBC, low Hgb, low HbF, etc). These markers are all indicators of severe disease, and given that the authors consolidated multiple studies, the fact that these all are associated with elevated ePASP is validating.

**Response:** Thank you so much for your attention. According to your comment, certain researchers have indicated that TRV is probably elevated in patients with severe anemia who have increased cardiac output and high stroke volume [1, 2]. However, the patients of the included studies were in steady state and not to be severely anemic.

We discussed the interesting findings from the subgroup analyses and provided a summary of findings that how these factors could confound the TTE findings. (Lines 414 to 451)

Reviewer #3: (No Response)

**Response:** Thank you so much for your time and attention.

7. PLOS authors have the option to publish the peer review history of their article ([what does this mean?](https://track.editorialmanager.com/CL0/https:%2F%2Fjournals.plos.org%2Fplosone%2Fs%2Feditorial-and-peer-review-process%23loc-peer-review-history/1/010f019448f6cb1d-246c11b0-dd28-460f-98ef-089bd149f2df-000000/FIlkWOOgeGPZ-fIzqOAB5VxZQdLa3Ql4YtG8cryFZgU=193)). If published, this will include your full peer review and any attached files.


If you choose “no”, your identity will remain anonymous but your review may still be made public.

**Response:** Thank you so much. According to your comment, we agree to publish the peer review history.

**Do you want your identity to be public for this peer review?** For information about this choice, including consent withdrawal, please see our [Privacy Policy](https://track.editorialmanager.com/CL0/https:%2F%2Fwww.plos.org%2Fprivacy-policy/1/010f019448f6cb1d-246c11b0-dd28-460f-98ef-089bd149f2df-000000/RxRx-gfjqhYVNRsTVp0uYO2BvrLAbiJG5Re54iWeBec=193).

Reviewer #2: No

Reviewer #3: **Yes:** Chibuzo Ilonze

**Response:** Thank you so much. I have a lot of respect for the time and attention of the editor and the reviewers have given to this manuscript and I wish all of them the best.

Sincerely,

Corresponding author: dr.mobin.gh@gmail.com

Address: Mazandaran University of Medical Science, Bou Ali Sina Hospital, Pasdaran Boulevard, Sari, Mazandaran Province, Iran.

PO BOX: 481675793

Tel & Fax: +98(0)11-33347837, +98(0)11-33349672

Scopus: 57726509000

Researcher ID: JUV-2911-2023

**References**

1.	Bunn, H.F., et al., *Pulmonary hypertension and nitric oxide depletion in sickle cell disease.* Blood, The Journal of the American Society of Hematology, 2010. **116**(5): p. 687-692.

2.	Caughey, M.C., et al., *Hemodynamic characteristics and predictors of pulmonary hypertension in patients with sickle cell disease.* The American journal of cardiology, 2012. **109**(9): p. 1353-1357.
